# Chemo- and regio-selective differential modification of native cysteines on an antibody *via* the use of dehydroalanine forming reagents†

**DOI:** 10.1039/d4sc00392f

**Published:** 2024-05-02

**Authors:** Steven Y. Yap, Tobias Butcher, Richard J. Spears, Clíona McMahon, Ioanna A. Thanasi, James R. Baker, Vijay Chudasama

**Affiliations:** a Department of Chemistry, University College London 20 Gordon Street London WC1H 0AJ UK v.chudasama@ucl.ac.uk j.r.baker@ucl.ac.uk

## Abstract

Protein modification has garnered increasing interest over the past few decades and has become an important tool in many aspects of chemical biology. In recent years, much effort has focused on site-selective modification strategies that generate more homogenous bioconjugates, and this is particularly so in the antibody modification space. Modifying native antibodies by targeting solvent-accessible cysteines liberated by interchain disulfide reduction is, perhaps, the predominant strategy for achieving more site-selectivity on an antibody scaffold. This is evidenced by numerous approved antibody therapeutics that have utilised cysteine-directed conjugation reagents and the plethora of methods/strategies focused on antibody cysteine modification. However, all of these methods have a common feature in that after the reduction of native solvent-accessible cystines, the liberated cysteines are all reacted in the same manner. Herein, we report the discovery and application of dehydroalanine forming reagents (including novel reagents) capable of regio- and chemo-selectively modifying these cysteines (differentially) on a clinically relevant antibody fragment and a full antibody. We discovered that these reagents could enable differential reactivity between light chain C-terminal cysteines, heavy chain hinge region cysteines (cysteines with an adjacent proline residue, Cys–Pro), and other heavy chain internal cysteines. This differential reactivity was also showcased on small molecules and on the peptide somatostatin. The application of these dehydroalanine forming reagents was exemplified in the preparation of a dually modified antibody fragment and full antibody. Additionally, we discovered that readily available amide coupling agents can be repurposed as dehydroalanine forming reagents, which could be of interest to the broader field of chemical biology.

## Abstract 2

Use of dehydroalanine-forming reagents to enable the first example of differentially modifying the native solvent accessible cysteines on an antibody.

## Graphical abstract

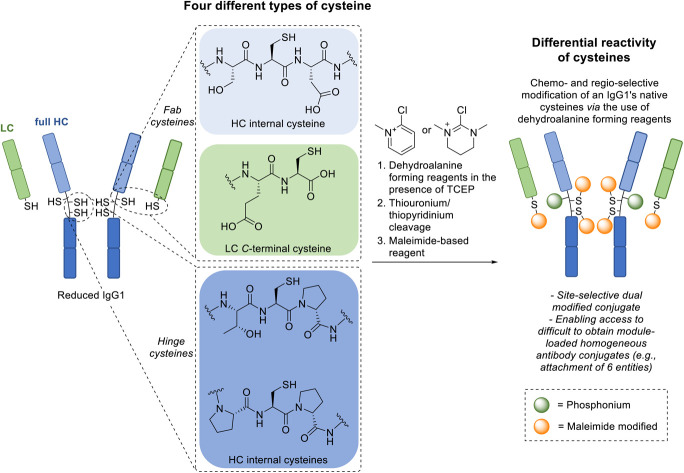

## Introduction

**Fig. 1:**
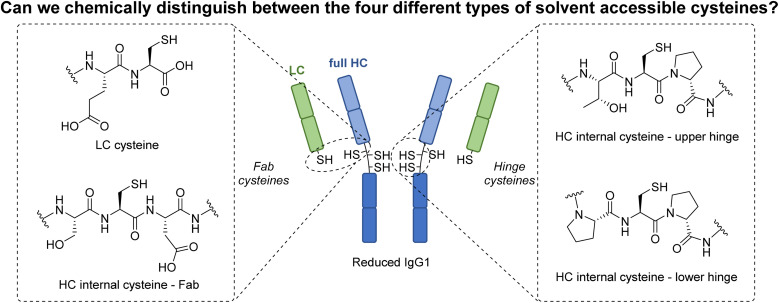
This work seeks to explore the possibility of distinguishing between the four different types of solvent accessible cysteines found on a typical IgG1 antibody as a conduit to exploring a fundamentally novel way to modify antibodies.

Over the past few decades, protein modification has become a fundamentally important tool in many aspects of chemical biology.^1–4^ In particular, and of most relevance to this manuscript, the modification of antibodies has enabled key advances in therapy, drug delivery, imaging, diagnostics, *etc.*^5–9^ Classically, native antibody modification has been carried out *via* stochastic lysine modification, which leads to a highly heterogeneous mixture of antibody conjugates, consequently limiting their potential.^10,11^ To circumvent this, efforts were made to site-selectively modify engineered antibody scaffolds (*e.g.*, chemical modification of cysteine-engineered antibodies,^12–15^ enzyme-directed modification of specifically “tagged” antibodies,^10,16–24^*etc.*). Whilst providing access to homogeneous antibody conjugates, the use of engineered antibodies comes with well-known caveats (*e.g.* time, cost, *etc.*).^25,26^ In more recent years, methods to site-selectively modify native antibodies have been developed, *e.g.* affinity ligand-based modification,^27–31^ glycan modification,^32–34^*etc.*, with the modification of cysteines liberated by the reduction of native (solvent accessible) disulfide bonds perhaps the most popular/common method for site-selective modification.^35–52^ Interestingly, despite a multitude of strategies being developed to modify these reduced disulfide bonds, all these methods have a common feature in that the liberated cysteines are reacted in the same manner. As a conduit to exploring a fundamentally novel way to modify antibodies, we have recently been intrigued by the possibility of trying to distinguish between the reactivity of the four different classes of solvent accessible cysteines liberated from the reduction of the interchain disulfides on a typical IgG1 antibody (Fig. 1). The four types of cysteine are a C-terminal cysteine on a light chain (LC) and three different internal cysteines on the heavy chain (HC). In a previous study, we showed that we can distinguish the reactivity of C-terminal cysteine and internal cysteines on a model peptide, *i.e.*, formation of a highly stable C-terminal thiophosphonium and dehydroalanine (Dha) respectively.^53^ However, as that core method relies on the formation of thiol unstable alkyl-aryl disulfides, it is not compatible with antibody disulfide systems (or any protein disulfide bond containing system) due to rapidly competing native disulfide bond formation. Nonetheless, this previous work showed that a Dha forming pathway could be a viable way to enable the first example of chemo- and regio-specific reactivity on the solvent accessible cysteines of an antibody. It is well known that Dha can act as an electrophilic centre upon reaction with various nucleophiles,^54^ and many chemical methods to site-selectively incorporate Dha onto proteins have been described, especially *via* the conversion of cysteine to Dha.^54–60^ Whilst numerous proteins have been successfully modified using these methods, the only work that is related to antibody modification pertains to antibody cysteine mutants generated by site-directed mutagenesis,^55,58,61^*i.e.*, there are no examples of exploiting Dha formation on a native antibody scaffold.

In this manuscript, we realise the goal of chemo- and regio-specific differential native cysteine modification on an antibody fragment and a full antibody *via* the use of reagents and strategies for Dha formation. Overall, we unveil that: (i) a cysteine's alpha proton acidity/protein microenvironment on a native IgG1 antibody can be exploited to enable selective Dha formation; (ii) novel Dha forming reagents can be tuned to prevent protein fragmentation (a general issue that is reported for Dha formation on proteins);^54,56,62,63^ (iii) how Dha forming reagents can be effectively applied to reduced disulfides; and (iv) how classically used (and readily available) coupling agents can be repurposed as effective Dha forming reagents.

## Results and discussion

### Reaction of reduced Fab with known Dha forming reagents

Our study began with the appraisal of the reaction of various known Dha forming reagents on the fragment antigen-binding (Fab) region of a full antibody. A Fab bears a single solvent accessible disulfide bridge, which upon disulfide reduction yields a LC C-terminal cysteine and a HC internal cysteine. Trastuzumab was chosen as a model antibody due to its clinical relevance (as antibody alone (*e.g.*, as a HER2-targeting cancer drug)^64,65^ and in the form of ADCs (*e.g.*, Kadcyla®, Enhertu®)^6,8,11,66^), availability, and as it is a humanised IgG1 (the vast majority of clinical ADCs are of this form).^11^

**Scheme 1:**
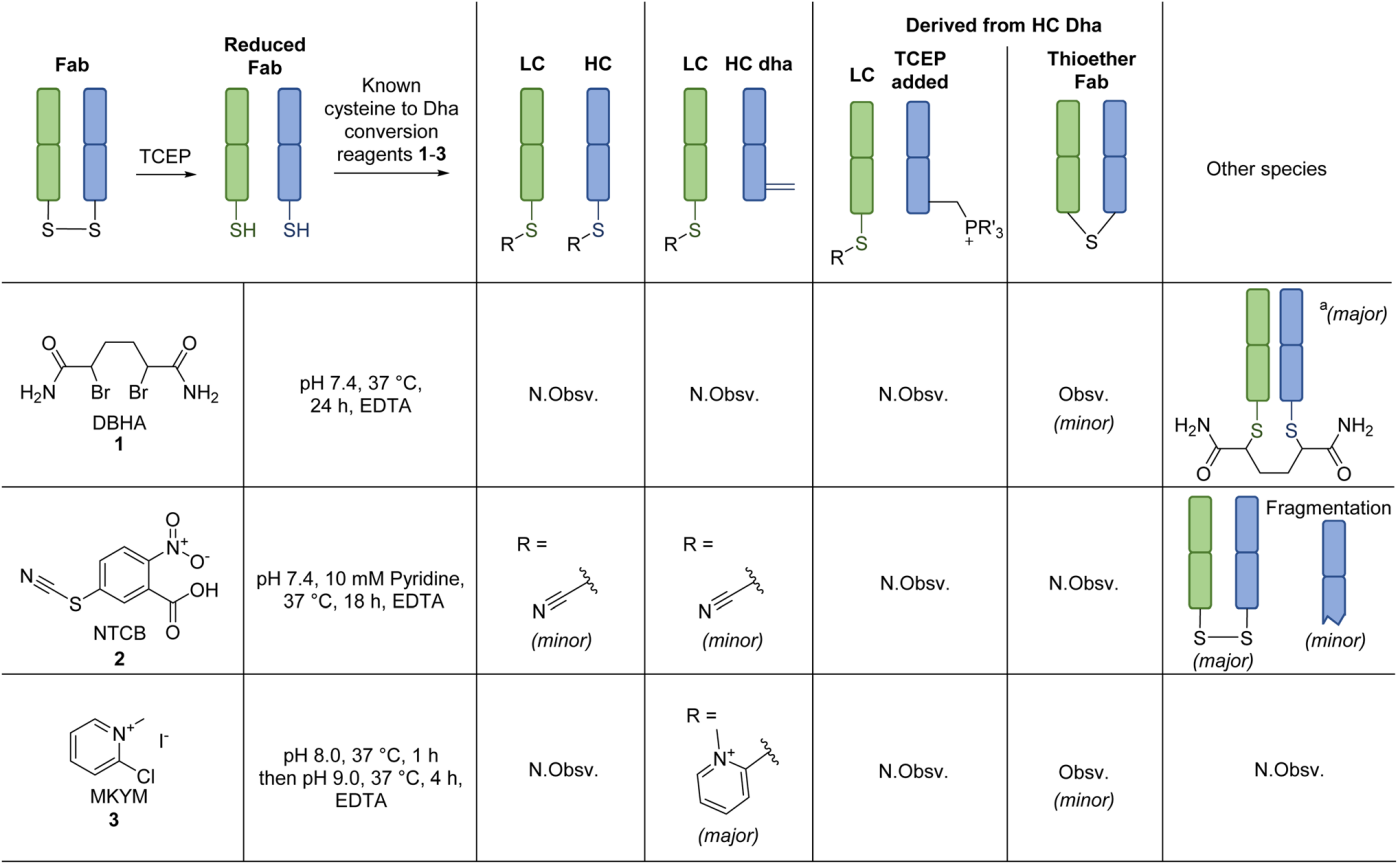
Reaction of known dehydroalanine forming reagents with reduced Fab (prepared *via* TCEP reduction at pH 8.0, 37 °C, 1.5 h). All reactions proceeded with complete conversion of the starting material Fab. N.Obsv. = Not Observed, Obsv. = Observed. ^a^Non-selective addition of DBHA 1 also observed on rebridged Fab.

Previously described Dha forming reagents, namely 2,5-dibromohexanediamide (DBHA) 1 (used most commonly), 2-nitro-5-thiocyanatobenzoic acid (NTCB) 2, and lesser utilised Mukaiyama reagent (MKYM) 3 (more often used as a coupling reagent), were incubated with reduced Fab.‡‡Note: We did not appraise the use of *O*-mesitylenesulfonylhydroxylamine (MSH) as we were unable to obtain local safety clearance for its synthesis (explosion risk) and all our efforts to purchase the reagent commercially were unsuccessful (despite exploring multiple avenues).,^56,58^ As somewhat expected, use of DBHA 1 primarily resulted in disulfide rebridging and, to a lesser extent, non-selective addition onto other nucleophilic amino acid residues (Scheme 1). It also resulted in the formation of a Fab-like mass with a loss of 32 Da as a minor product, which presumably formed *via* the reaction of Dha on one antibody chain and a cysteine on the other, thus generating a thioether bond. Following a literature protocol,^56^ use of NTCB 2 resulted in rapid cyano transfer onto thiols and subsequent partial Dha formation was observed only on HC (Scheme 1). However, competing disulfide formation back to native Fab, promoted by the reagent, was a major issue. Furthermore, fragmentation of the HC was observed, with cleavage of the backbone *N*-amide bond of the HC cysteine. This phenomenon was reported by Qiao *et al.*, whereby cyano cysteine and cysteine *N*-amide undergoes intramolecular cyclisation and subsequent nucleophilic substitution with hydroxide/amine nucleophiles (Scheme 3A).^56^

Out of the reagents assessed, MKYM 3 was the most effective, *i.e.*, capable of full Dha conversion on HC whilst forming thiopyridinium on LC, albeit with the formation of a small amount of thioether Fab (Scheme 1). Literature has described MKYM 3 as being poor at converting cysteine to Dha, capable of Dha formation only at high pH ∼11–12.^58^ Nevertheless, our attempt to react reduced Fab with MKYM 3 led to the efficient chemo-selective addition of one methylpyridinium onto the LC and HC cysteines, followed by regio-selective formation of Dha on the HC when the pH was increased to pH 9. Overall, these observations showcased that the adjacent carboxylate inferred stability to the methylpyridinium at the C-terminal cysteine position and prevented Dha formation from thiopyridinium and/or that the HC cysteine is particularly susceptible to Dha formation, possibly due to its local microenvironment. The presence of thioether Fab, presumably formed *via* a LC cysteine thiol reacting with HC Dha, implied that hydrolysis of thiopyridinium is a competing reaction that can introduce an unfavourable reaction pathway. To further confirm the site of addition and elimination on reduced Fab, *N*-ethylmaleimide (a thiol reactive reagent) was added post reaction of MKYM 3 with reduced Fab, and no maleimide addition was observed. Together, these results indicated that the site of addition (and subsequent elimination for HC) was at cysteine.

### Reaction of Fab with thiouronium forming reagents

Inspired by the positive results obtained on reaction of MKYM 3 with reduced Fab, we investigated other reagents that could generate a thiopyridinium-like structure. Naturally, we gravitated toward using commercially available reagents, namely carbodiimides and thiouronium-based reagents, *i.e.*, *N*-ethyl-*N*′-(3-dimethylaminopropyl)carbodiimide hydrochloride (EDC) 4, *S*-benzylisothiourea (SBTU) 5, and *S*-(1-oxido-2-pyridyl)-1,1,3,3-tetramethylthiuronium hexafluorophosphate (HOTT) 6. We also note that these reagents are also commonly used amide coupling reagents.

**Scheme 2:**
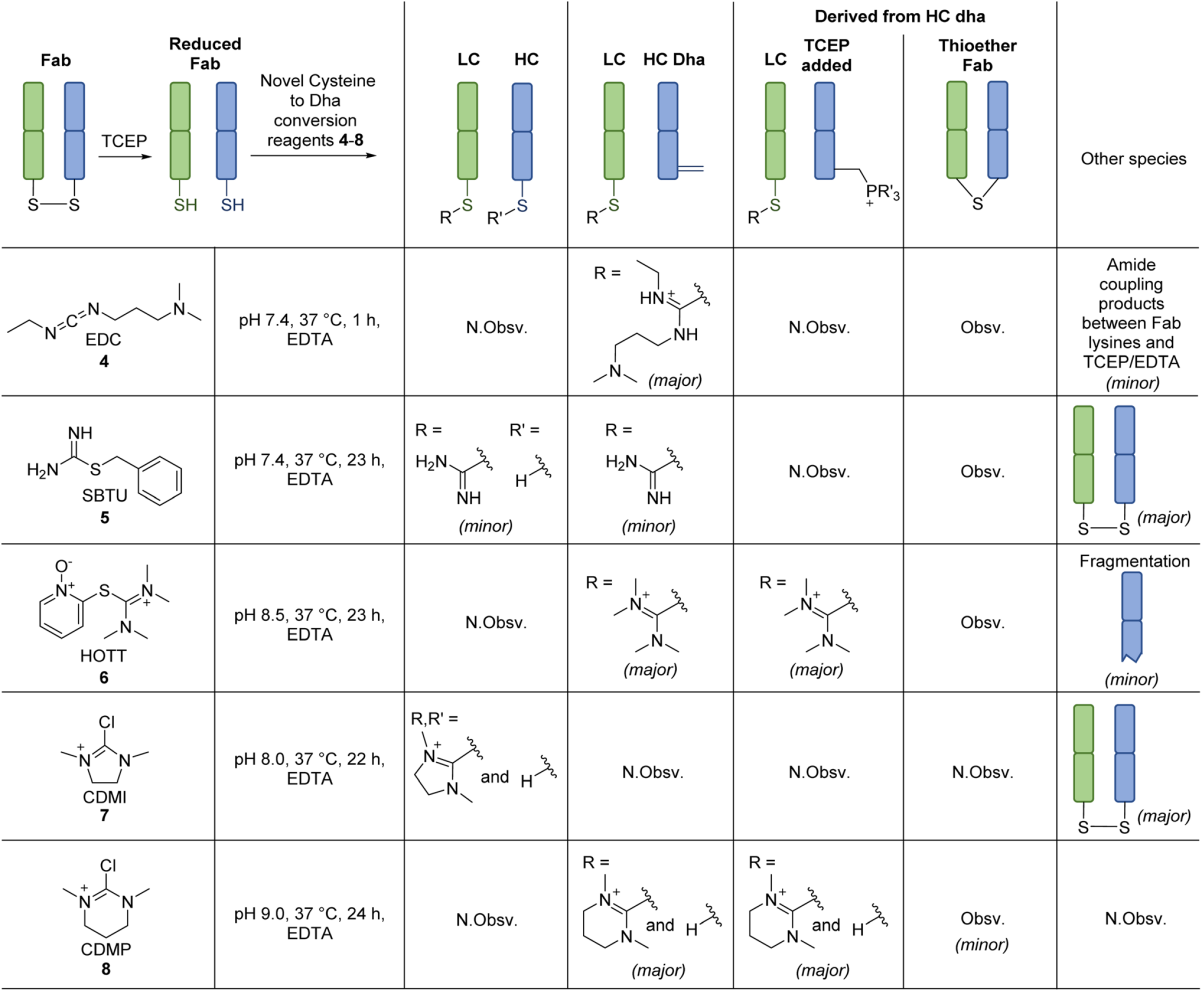
Reaction of reagents capable of forming thiouronium with reduced Fab. Reactions were monitored by LC-MS. All reactions proceeded with complete conversion of the starting material Fab. N.Obsv. = Not Observed, Obsv. = Observed.

**Scheme 3:**
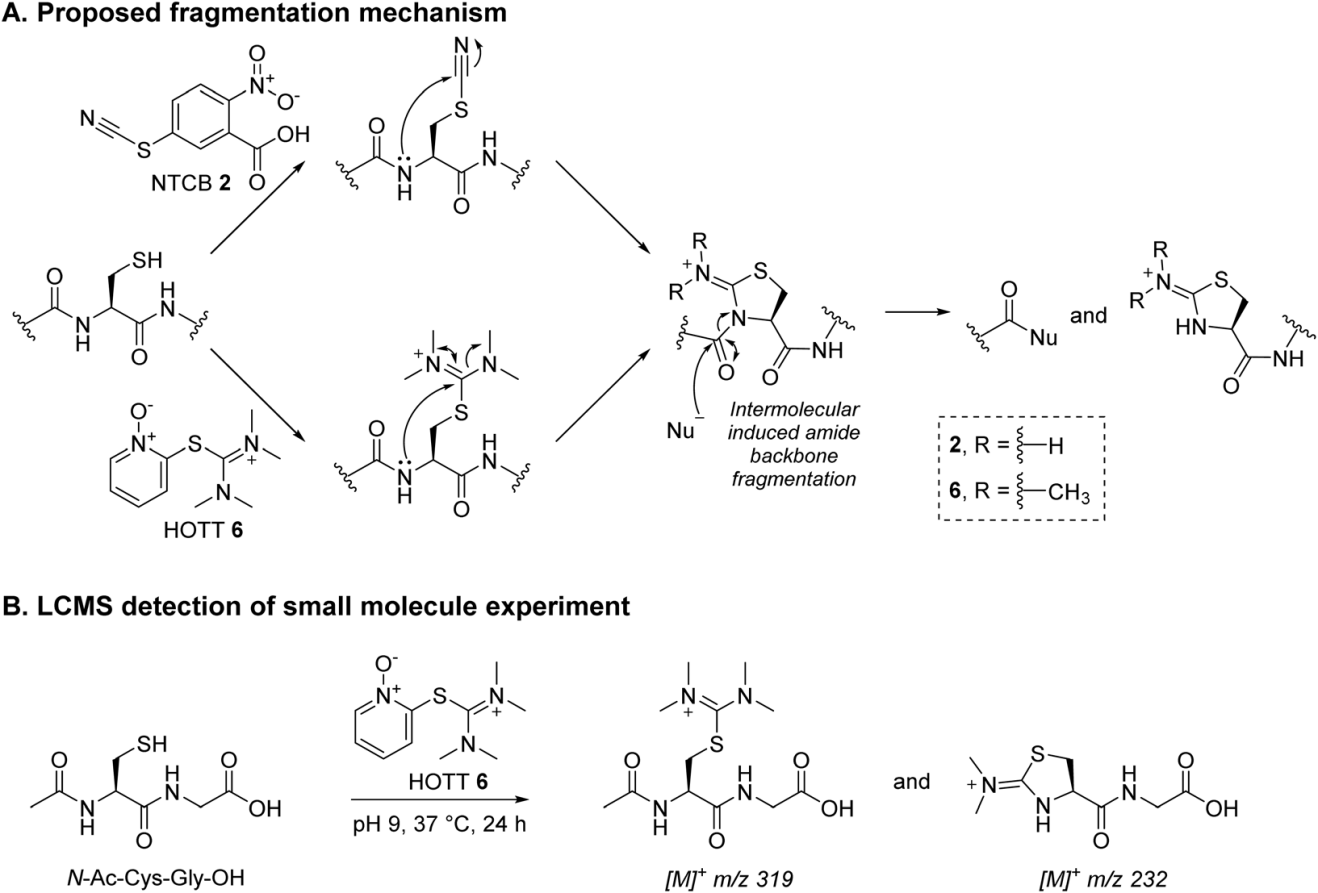
(A) Proposed fragmentation mechanism by NTCB 2 and HOTT 6.^56^ (B) Reaction between *N*-Ac-Cys-Gly-OH and HOTT 6 was analysed using LC-MS; the data obtained supported the proposed mechanism.

EDC 4, when reacted with reduced Fab, showed rapid addition onto both LC and HC cysteines to initially form thiouroniums followed by rapid and selective HC cysteine elimination to Dha at pH 7.4 at 37 °C in 1 h (Scheme 2). However, although minor, EDC 4 was also capable of promoting amide formation between the carboxylic acids on ethylenediaminetetraacetic acid (EDTA) (and to some extent tris(2-carboxyethyl)phosphine (TCEP)) with lysine residues on the Fab, thus limiting its use, especially as EDTA is needed in the buffer to prevent the reduced disulfide from being oxidised back to native Fab.^67^ Reaction between reduced Fab and SBTU 5 resulted in incomplete thiouronium addition onto the Fab cysteines after 1 h and leaving the reaction on for longer resulted in varying mixtures of LC thiouronium, several HC species (thiouronium, Dha, *etc.*), thioether Fab, and re-oxidation back to native Fab (Scheme 2). The varying mixture of species observed was presumably due to the highly hydrolysable nature of the thiouronium formed with SBTU 5. This would subsequently lead to incomplete formation of HC Dha, formation of thioether Fab, and re-formation of Fab from free LC and HC. HOTT 6, on the other hand, was capable of regio-selective Dha conversion upon reaction with reduced Fab. When incubated at pH 8.5 and 37 °C overnight, this resulted in full conversion of HC thiouronium to Dha (Scheme 2). We observed that incomplete removal of TCEP from the initial reduction step could result in phospha-Michael addition onto Dha. Another interesting observation was the presence of a small amount of HC fragmentation, with cleavage of the backbone *N*-amide bond of the HC cysteine, akin to what we observed when employing NTCB 2 (Scheme 3A). Further investigation, in a small molecule study using *N*-Ac-Cys-Gly-OH as a model (Scheme 3B), suggested a similar fragmentation mechanism to that of NTCB 2 as described by Qiao *et al.* was taking place when using HOTT 6.^56^

Overall, we discovered that a thiopyridinium or thiouronium intermediate formed upon reaction of reduced Fab with MKYM 3, EDC 4, and HOTT 6, can enable differential reactivity between a C-terminal LC cysteine and internal HC cysteine on a typical IgG1 Fab *via* the formation of thiopyridium/thiouronium on LC and Dha on HC. Use of MKYM 3 resulted in minimal side-reactions (*i.e.*, formation of thioether Fab); EDC 4 exhibited rapid thiouronium elimination to form Dha on HC, but this was accompanied by unrelated amide coupling of EDTA/TCEP to Fab lysines; use of HOTT 6 overcame the undesired amide coupling observed for EDC 4 but was plagued by a HC fragmentation side-reaction. To try and overcome the shortfall of HOTT 6, reagents capable of generating cyclic thiouronium were explored, *i.e.*, chloro-1,3-dimethylimidazolinium chloride (CDMI) 7 and 2-chloro-1,3-dimethyl-3,4,5,6-tetrahydropyrimidinium (CDMP) 8. It was hypothesised that the cyclic thiouroniums that would be generated on reaction of these reagents with cysteine would no longer be prone to fragmentation, as the amide cyclisation step with amine elimination would be entropically disfavoured by the cyclic nature of the thiouronium and as cyclic thiouroniums would (in general) be more stable. Gratifyingly, CDMP 8 displayed regio-selective Dha conversion on HC internal cysteine with no undesired fragmentation (Scheme 2). Reaction of reduced Fab with CDMI 7, however, had an unfavourable outcome; it resulted in reoxidation of Fab with a small amount of thiouronium formation observed on LC cysteine (see ESI†). It was deduced that the thiouronium formed with CDMI 7 was highly prone to hydrolysis, which led to various side-reactions. CDMP 8, however, appeared to be an appropriate reagent to enable differential reactivity between C-terminal cysteines and internal cysteines and overcame undesired fragmentation observed with HOTT 6.

Despite the success of unveiling several reagents (*i.e.*, MKYM 3, EDC 4, HOTT 6, and CDMP 8) that could enable differential reactivity depending on whether a cysteine was C-terminal or internal, thioether Fab was formed in all such reactions. This was thought to be a consequence of C-terminal thiouronium or thiopyridinium adducts undergoing hydrolysis under the reaction conditions employed, which would liberate free LC cysteine to react with HC Dha. To prevent the formation of thioether Fab, it was hypothesised that nucleophilic Michael addition onto Dha with a surrogate molecule prior to thiouronium or thiopyridinium hydrolysis would prevent this by acting as a trap for any Dha formed.

In view of the above, several nucleophiles reactive to Dha were to be assessed, namely, thiols,^54,57,58,68,69^ amines,^55,60,70,71^ and phosphines^72,73^ – we note that the products of these reactions would likely be formed as diastereomeric mixtures. To test these nucleophiles in the above context, although it was accompanied by unrelated amide coupling reactions to Fab, EDC 4 was selected due to its time efficiency in converting cysteine to Dha (1 h). As such, various nucleophiles were added after 1 h of incubation of reduced Fab with EDC 4. The thiol nucleophiles that were assessed were dithiothreitol (DTT), 2-mercaptoethanol (BME), and 4-mercaptophenylacetic acid (MPAA). These thiols did react with HC Dha, however, they also reacted with LC thiouronium, reverting it back to a free cysteine and thus stimulating the formation of thioether Fab (see ESI†). Nonetheless, this discovery also provided us with knowledge of how to effectively cleave off LC thiouronium adducts. For aza-Michael addition, several amines of different reactivity were selected and tested, including aryl amine (*p*-anisidine), primary alkyl amines (benzyl amine), secondary alkyl amine (piperidine) and “soft” amines (hydrazine and hydroxylamine). Unfortunately, even under harsh conditions, *i.e.*, at high amine molar equivalence (1000 eq.), high pH, and long reaction time (24 h), all amines, except for hydrazine, were unreactive towards HC Dha (see ESI†). Even in the case of hydrazine, incomplete hydrazine addition was observed, even after overnight incubation and this also resulted in numerous side reactions. Therefore, aza-Michael addition was deemed unsuitable. In terms of phosphine nucleophiles, TCEP effectively added to HC Dha to form HC phosphonium and was unreactive towards the LC thiouronium. To prove the stability and the site of TCEP addition, the LC thiouronium and HC phosphonium adduct was incubated with thiol nucleophile (1000 eq. BME, pH 7.4, 37 °C, 1 h). As expected, LC thiouronium was reverted back to native cysteine and, to our delight, the HC phosphonium adduct was shown to be stable to thiols as no further thiol addition was observed on the HC (see ESI†). This breakthrough prompted further investigation into the use of other phosphine reagents, specifically commercially available tris(hydroxypropyl)phosphine (THPP), as well as two TCEP-derivatives, one bearing an *N*-propargyl amide and two methyl ester groups and the other bearing three *N*-propargyl amide groups; the *N*-propargyl amide and ester groups were prepared from the acid moieties present on TCEP *via* amide coupling and esterification, respectively. However, only THPP and the TCEP-derivative bearing three alkyne groups added to HC Dha and they only did so at a very slow rate–the reactions did not even go to completion after overnight; side-reactions were also observed (see ESI†).

Overall, in this study, TCEP was identified as the nucleophile of choice for reaction with HC Dha; TCEP could add effectively and fully react with HC Dha *via* phospha-Michael addition and was inert towards LC thiouronium/thiopyridinium. It was also discovered that thiols were effective reagents to cleave thiouroniums to regenerate native cysteine.

### Application of Dha forming reagents to dually modify a Fab regio-selectively

**Fig. 2:**
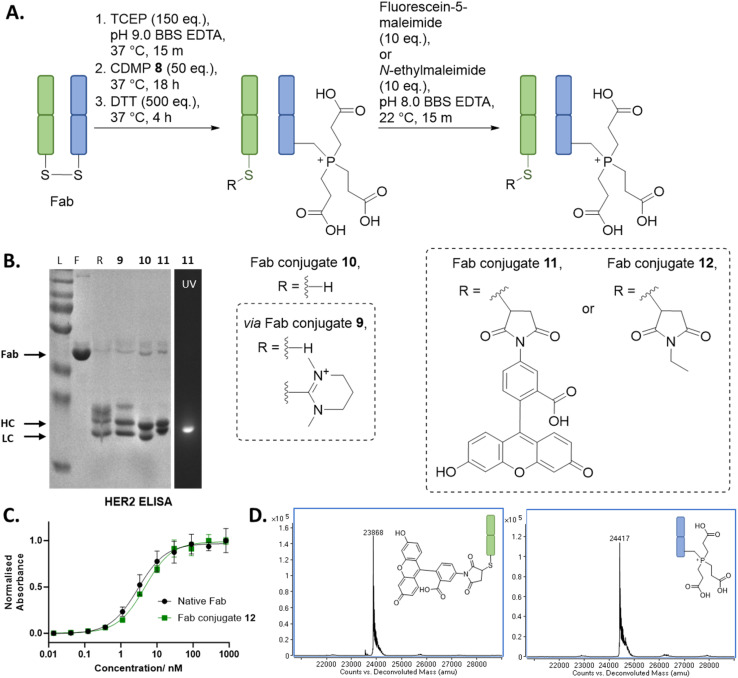
(A) Reaction scheme for the synthesis of a dually functionalised Fab using CDMP 8, TCEP, and maleimide. All reactions proceeded with complete conversion of the starting material Fab. (B) SDS PAGE: L: protein ladder; F: native Fab; R: reduced Fab. (C) ELISA study on Fab conjugate 12. (D) Deconvoluted LC-MS of Fab conjugate 11; Fab conjugate 11 LC, expected mass: 23 867 Da, observed mass: 23 868 Da; Fab conjugate 11 HC, expected mass: 24 418 Da, observed mass: 24 417 Da (see ESI† for full details).

With the above discoveries in mind, we attempted to form a dually modified Fab with the HC bearing a phosphonium and the LC functionalised with a maleimide. To the best of our knowledge, this would be the first example of chemo-specific differential modification of the solvent accessible cysteines on a native antibody fragment. Initially, regio-selective formation of Dha was carried out using MKYM 3 and CDMP 8, followed by TCEP addition onto Dha, cleavage of LC thiopyridinium/thiouronium using an excess of thiol, and then finally reaction of LC cysteine with a maleimide. However, despite the use of TCEP limiting the amount of thioether Fab formed by trapping some of the Dha formed, it was still present as a side-reaction. It was at this point we conceived of the idea of adding excess TCEP at the start of the reaction and not washing out the TCEP prior to addition of reagents (MKYM 3 or CDMP 8). This would result in any Dha that was being generated being effectively trapped with TCEP in an *in situ* phospha-Michael addition. Gratifyingly, this alternative method resulted in very minimal thioether Fab formation, and we also discovered that cleavage of LC thiopyridinium/thiouronium to yield LC cysteine in the following step could be carried out *via* the use of DTT or by hydrolysis induced at pH 9 (see ESI† for cleavage *via* hydrolysis). Modification of the free LC cysteine with fluorescein-5-maleimide yielded dually modified Fab conjugate 11, which was made up of LC fluorescein and HC phosphonium (Fig. 2, data shown for CDMP 8, see ESI† for details on use of MKYM 3), as determined by LC-MS (see ESI† for details). SDS-PAGE analysis corroborated this finding as it showed only the LC was fluorescent, indicating that only the LC was functionalised with fluorescein. HER2 ELISA studies were carried out on an analogue of Fab conjugate 11, *i.e.*, Fab conjugate 12 bearing modification with *N*-ethylmaleimide instead of fluorescein-5-maleimide to prevent UV-vis absorption interference with ELISA analysis. Pleasingly, the ELISA assay indicated no decrease in binding relative to native Fab.

### Application of Dha forming reagents to a full antibody

Spurred on by our results on Fab, we turned our attention to appraise our novel discovery on a more complex system, the full IgG1 of trastuzumab. Trastuzumab was the obvious choice for appraisal on a mAb in view of the above work on its Fab and as it is a clinically validated antibody in various contexts.^64,65^ As mentioned above, trastuzumab mAb bears four solvent accessible disulfides, which upon reduction will liberate four different types of cysteine: three internal cysteines on full HC (referred as full HC in this manuscript to differentiate from the aforementioned HC on Fab) and the same LC that was appraised above bearing a C-terminal cysteine (further details provided in Fig. 1). *Prima facie*, we expected our method to form three Dhas on the HC and a thiouronium on the LC, but we were also cautious in this assumption as the sequence within the hinge region of trastuzumab (–HTCPPCPAP–) shows that both hinge region cysteines had a neighbouring proline residue (–CP–/–Cys–Pro–), which could reduce alpha-proton acidity/accessibility.^63,74,75^

**Fig. 3:**
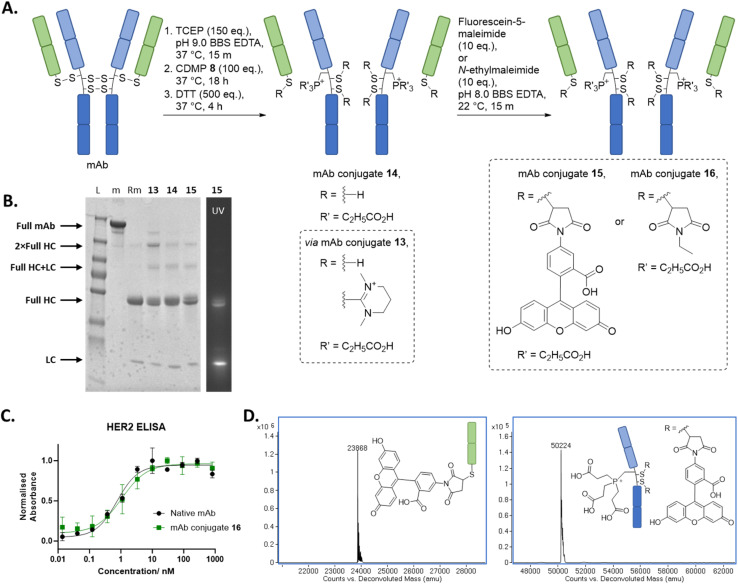
(A) Reaction scheme for the synthesis of functionalised mAb using CDMP 8, TCEP, and maleimide. All reactions proceeded with complete conversion of the starting material mAb. (B) SDS PAGE: L: protein ladder; m: native full mAb; Rm: reduced mAb. (C) ELISA study on mAb conjugate 16. (D) Deconvoluted LC-MS of mAb conjugate 15; mAb conjugate 15 LC, expected mass: 23 867 Da, observed mass: 23 868 Da; mAb conjugate 15 full HC, expected mass: 50 223 Da, observed mass: 50 223 Da (see ESI† for full details).

When a similar method (using CDMP 8), as used to prepare dually functionalise Fab 11, was applied to trastuzumab, this resulted in the formation of mAb conjugate 15, which comprised a LC + fluorescein and full HC + 1 × phosphonium+2 × fluorescein (Fig. 3). We would like to note that the quantification of the fluorescence signal on SDS-PAGE shows a signal ratio of *ca.* 2 : 1 for LC : HC (Fig. 3B), in contrast to the expected *ca.* 1 : 2 ratio based on fluorescein loading. We believe this discrepancy is due to self-quenching of the fluorophores, a well-known phenomenon where fluorophores in close proximity can lead to a decrease in expected fluorescence emission intensity.^76–79^ Our regio-selective method resulted in only one full HC cysteine being converted into Dha, whilst the two other full HC cysteines remained as thiouroniums or as free cysteines (the presence of free cysteines was due to partial hydrolysis of thiouronium) in the initial steps, and LC cysteine (as expected) remained as thiouronium or free cysteine; this was all confirmed by LC-MS analysis (see ESI† for details). Based on Fab experiments described above, we presumed that the thiouronium that was converted to Dha was positioned in the constant CH1 domain instead of the hinge region and that the secondary amide on the proline residue imparted more rigidity and/or reduced alpha proton acidity to the hinge cysteine residues, thus resulting in less favourable conditions for Dha formation for these cysteines.^63,74,75^ To appraise this further, an enzymatic digestion experiment with papain was carried out on *N*-ethylmaleimide analogue of mAb conjugate 15, *i.e.*, mAb conjugate 16, which yielded LC maleimide and HC phosphonium, confirming the location of installed Dha. Fc 2 × maleimide was not observed on LC-MS, suspected due to the low ionisable nature of Fc and the overlapping of the retention time with LC maleimide on LC-MS. Overall, our protocol resulted in, to the best of our knowledge, the first example of native thiol-based regio- and chemo-selective dual modification of a full antibody. Moreover, our protocol enables access to conventionally difficult to access drug-to-antibody ratio (DAR) conjugates of 6. Similar to our study on dually modified Fab, HER2 ELISA was carried out on mAb conjugate 16, which gratifyingly indicated no decrease in binding relative to native trastuzumab.

### Small molecule and peptide studies to further understand reactivity/selectivity

**Scheme 4:**
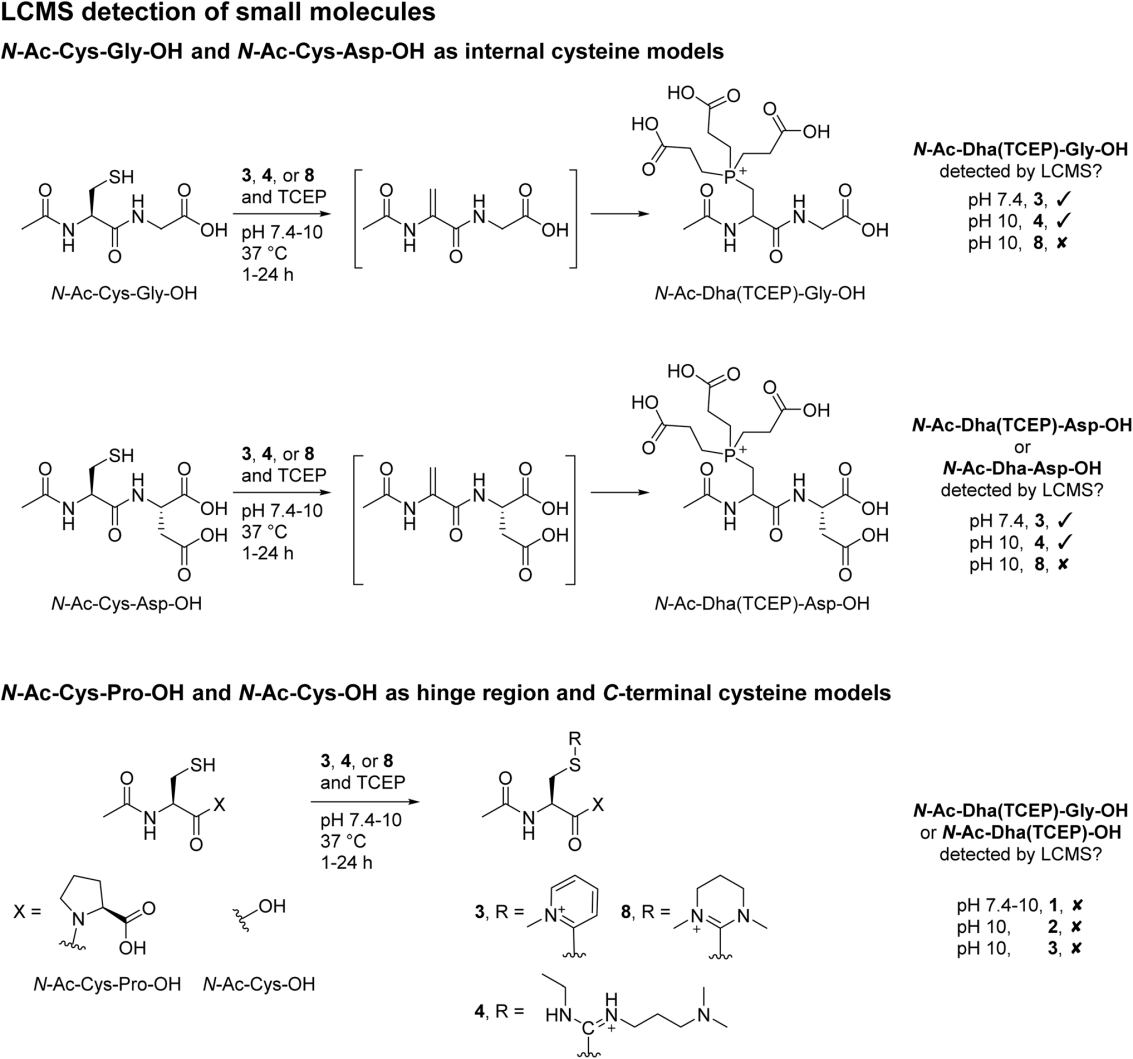
LCMS detection of small molecule studies. (top) Reaction of *N*-Ac-Cys-Gly-OH and *N*-Ac-Cys-Asp-OH, as internal cysteine models, with 3, 4, or 8. (bottom) Reaction of *N*-Ac-Cys-OH and *N*-Ac-Cys-Pro-OH, as C-terminal cysteine and hinge region cysteine models, respectively, with 3, 4, or 8, in which no Dha formation was observed.

To appraise our findings further, small molecule models were carried out using *N*-Ac-Cys-Gly-OH as a generic internal cysteine, *N*-Ac-Cys-Asp-OH as a model for Fab HC internal cysteine, *N*-Ac-Cys-OH as a model for LC C-terminal cysteine, and *N*-Ac-Cys-Pro-OH to represent hinge region cysteines. MKYM 3, EDC 4, and CDMP 8 were investigated in terms of reaction with these model small molecules (Scheme 4); EDC 4 was chosen due to its high efficiency in converting cysteine to Dha; and MKYM 3 and CDMP 8 as they performed best for dually modifying Fab.

First, the reaction of MKYM 3, EDC 4, and CDMP 8 with *N*-Ac-Cys-Gly-OH (internal cysteine) was explored. *N*-Ac-Dha(TCEP)-Gly-OH was detected on LC-MS upon reaction of MKYM 3 with *N*-Ac-Cys-Gly-OH at pH 10 and 37 °C for 24 h, albeit with some starting *N*-Ac-Cys-Gly-OH still observed after this time. Incubating EDC 4 and *N*-Ac-Cys-Gly-OH in the presence of TCEP at pH 7.4 and 37 °C for 24 h gave rise to complete conversion of *N*-Ac-Cys-Gly-OH and the formation of *N*-Ac-Dha(TCEP)-Gly-OH, as determined by LC-MS analysis. Unfortunately, despite CDMP 8 being capable of forming Dha on Fab and full mAb, it was not able to convert cysteine to Dha as no *N*-Ac-Dha(TCEP)-Gly-OH was detected upon reaction with *N*-Ac-Cys-Gly-OH. Reaction with CDMP 8 did show that thiouronium was formed, however, under these reaction conditions, the generated thiouronium fully hydrolysed overnight. Thus, it was hypothesised that under these conditions competing hydrolysis resulted in low/no Dha formation; this was not observed on protein models as higher equivalents could be used, and a lower pH was required. To probe this further, we also carried out the reaction of MKYM 3, EDC 4, and CDMP 8 with *N*-Ac-Cys-Asp-OH (a mimic of the HC internal cysteine). The results from these reactions indicate that the presence of the extra carboxylic acid, if anything, slows down the rate of conversion to Dha (see ESI† for details). Collectively, these results seem to indicate that the specific alpha proton of the HC Fab cysteine is likely to be particularly acidic or in an usually basic environment due to its 3D protein microenvironment. Next, the incubation of reagents MKYM 3, EDC 4, and CDMP 8 with *N*-Ac-Cys-OH (C-terminal cysteine) and *N*-Ac-Cys-Pro-OH (hinge region-like cysteine) was carried out. This resulted in the formation of respective thiouronium or thiopyridinium with no subsequent elimination to form Dha in all cases. Overall, these small molecule models demonstrated the stability of cysteine thiouronium or thiopyridinium towards elimination for Dha formation at the C-terminus and when adjacent to a proline (Cys–Pro) residue. Finally, to further explore the differential reactivity we unveiled in the manuscript, reaction of reagents MKYM 3, EDC 4, and CDMP 8 with somatostatin (a clinically relevant cyclic peptide containing a C-terminal cysteine and an internal cysteine not adjacent to proline)^80,81^ in the presence of TCEP was carried out. This, to our delight, resulted in the formation of Dha(TCEP) and a thiouronium or thiopyridinium for all reagents (see ESI† for details).

## Conclusion

Despite the modification of an antibody's native solvent accessible cysteines being exploited for many years, we unveil the first examples of regio- and chemo-selective differential modification of these cysteines on a clinically validated antibody fragment and full antibody. This was achieved by developing several novel Dha forming reagents, *e.g.*, MKYM 3 and CDMP 8, that enable differential reactivity between a LC *C*-terminal cysteine, HC hinge region cysteines (cysteine with an adjacent proline residue, –Cys–Pro–), and other internal cysteines. Dually modified Fab and antibody conjugates were prepared by our novel method, and selectivity was determined by LC-MS, SDS-PAGE, and using enzymatic digestion protocols. Whilst this method does result in the absence of a covalent link between the antibody chains, retention of antibody binding was observed by ELISA, it is well-document that many clinically approved ADCs also bear this feature (*e.g.*, Enhertu®, Trodelvy®),^6,8,11,66^ and our method also enables facile access to difficult to obtain (and thus underexplored) 6 module-loaded antibody conjugates. Moreover, this method could open the door to fundamentally novel pathways for Dha-based chemo- and regio-selective modification of various disulfide-containing proteins and peptides. In addition, the discovery of new Dha forming reagents, more generally, could be of significant interest for the broader field of chemical biology, especially as we show that readily available amide coupling agents can now be repurposed in this context.

## Data availability

Synthetic chemistry experimental details, including synthetic procedures and compound characterizations, *i.e.*, NMR, IR & MS spectra. Chemical biology experimental details, including bioconjugation procedures, details on antibody digestion, LC-MS methodology, and full LC-MS spectra including TIC and raw data.

## Author contributions

S. Y. Y. synthesised the small molecules. S. Y. Y. carried out the bioconjugation experiments on the Fab and mAb. S. Y. Y. and T. B. carried out experiments on small molecules and peptides. I. A. T. generated the Fab fragment. S. Y. Y. and C. M. M. conducted the ELISA studies. S. Y. Y., R. J. S., J. R. B., and V. C. conceived and designed the project/experiments. S. Y. Y. and V. C. co-wrote the manuscript. All authors read and approved the final manuscript.

## Conflicts of interest

There are no conflicts to declare, but we highlight that V. C. and J. R. B. are directors of UCL spin-out ThioLogics.

## Supplementary Material

SC-015-D4SC00392F-s001
