# Supplementary material for: Chemo- and regio-selective differential modification of native cysteines on an antibody via the use of dehydroalanine forming reagents
Source: Chem Sci. 2024 May 2;15(22):8557–68. doi: 10.1039/d4sc00392f (PMC11151841; doi:10.1039/d4sc00392f)
Supplement: SC-015-D4SC00392F-s001 [file SC-015-D4SC00392F-s001.pdf]

Electronic Supplementary Material (ESI) for Chemical Science.
This journal is © The Royal Society of Chemistry 2024

Chemo- and regio-selective differential modification of native cysteines on an antibody *via* the use of dehydroalanine forming reagents

Steven Y. Yap,[a] Tobias Butcher,[a] Richard J. Spears,[a] Clíona M. McMahon,[a] Ioanna A. Thanasi,[a] James R. Baker,[a,]* Vijay Chudasama[a,]*

[a] Department of Chemistry, University College London, 20 Gordon Street, London, WC1H 0AJ, UK.

* Correspondence: v.chudasama@ucl.ac.uk, j.r.baker@ucl.ac.uk

Table of contents

General experimental ... 4
LCMS analysis for peptides and small molecules ... 4
Organic synthesis ... 5
Synthesis of *N*-Ac-Cys(trt)-Gly-OtBu S1 ... 5
Synthesis of *N*-Ac-Cys-Gly-OH S2 ... 7
Synthesis of *N*-Ac-Cys(trt)-Pro-OtBu S3 ... 9
Synthesis of *N*-Ac-Cys-Pro-OH S4 ... 11
Synthesis of *N*-Ac-Cys(trt)-Asp-OtBu S105 ... 13
Synthesis of *N*-Ac-Cys-Asp-OH S106 ... 15
Synthesis of 3,3',3''-phosphanetriyltris(*N*-(prop-2-yn-1-yl)propanamide) S5 ... 17
Synthesis of 3-(bis(3-methoxy-3-oxopropyl)phosphaneyl)propanoic acid[1] S6 ... 20
Synthesis of dimethyl 3,3'-((3-oxo-3-(prop-2-yn-1-ylamino)propyl)phosphanediyl)dipropionate S7 ... 23
Synthesis of 2-chloro-1,3-dimethyl-3,4,5,6-tetrahydropyrimidin-1-ium hexafluorophosphate[2] CDMP 8 ... 26
Bioconjugation general experimental ... 28
SDS-PAGE ... 28
Preparation of 5× SDS PAGE loading dye ... 28
Preparation of 10× running buffer ... 28
Preparation of Coomassie blue stain ... 28
LCMS analysis for Fab and full antibodies ... 28
Assessing reagents on Fab (experimental and mass spec data) ... 30
Native Trastuzumab Fab S9 ... 30
Reduced Fab ... 31
Fab reactions ... 32
Reaction of reduced Fab with DBHA 1 ... 32
Reaction of reduced Fab with NTCB 2 ... 33
Reaction of reduced Fab with MKYM 3 ... 35
Control reaction of reduced Fab with MKYM 3 and then *N*-ethylmaleimide ... 37
Reaction of reduced Fab with EDC 4 ... 42

Reaction of reduced Fab with SBTU 5 45
Reaction of reduced Fab with HOTT 6 47
Reaction of reduced Fab with CDMI 7 49
Reaction of reduced Fab with CDMP 8 53
Screening of nucleophiles 55
2-mercaptoethanol (BME) 56
Dithiothreitol (DTT) 58
4-mercaptophenylacetic acid (MPAA) 60
Benzylamine 62
Piperidine 64
p-Anisidine 66
Hydroxylamine 68
Hydrazine 70
TCEP 72
tris(hydroxypropyl)phosphine (THPP) 73
3,3',3''-phosphanetriyltris(N-(prop-2-yn-1-yl)propanamide) S5 74
Dimethyl 3,3'-((3-oxo-3-(prop-2-yn-1-ylamino)propyl)phosphanediyl)dipropionate S7 76
Dually modified Fab conjugate 11 78
SDS-PAGE Fab conjugate 11 80
LCMS Fab conjugate 9 81
LCMS Fab conjugate S99 83
LCMS Fab conjugate 11 85
LCMS Fab conjugate 12 87
Dually modified IgG 89
SDS-PAGE mAb conjugate 15 90
LCMS Native Trastuzumab S100 91
LCMS of reduced Trastuzumab 92
LCMS mAb conjugate 13 94
LCMS mAb conjugate 15 96
LCMS mAb conjugate 16 98
Papain digestion of mAb conjugate 16 100
Small molecule experiments 103
Reaction of HOTT 6 with S2 104
Reaction of MKYM 3 and TCEP with S2 105
Reaction of MKYM 3 and TCEP with S4 106
Reaction of MKYM 3 and TCEP with *N*-acetyl-cysteine 107
Reaction of MKYM 3 with N-Ac-Cys-Asp-OH S106 108
Positive ESI 108

Negative ESI 109
Reaction of EDC 4 with S2 110
Reaction of EDC 4 and TCEP with S4 111
Reaction of EDC 4 and TCEP with *N*-acetyl-cysteine 112
Reaction of EDC 4 with N-Ac-Cys-Asp-OH S106 113
Positive ESI 113
Negative ESI 114
Reaction of CDMP 8 and TCEP with S2 115
1 h timepoint 115
24 h timepoint 116
Reaction of CDMP 8 and TCEP with S4 117
1 h timepoint 117
24 h timepoint 118
Reaction of CDMP 8 and TCEP with *N*-acetyl-cysteine 119
1 h timepoint 119
24 h timepoint 120
Reaction of CDMP 8 with N-Ac-Cys-Asp-OH S106 121
Positive ESI 121
Negative ESI 122
Peptide experiments 123
Reaction of MKYM 3 and TCEP with somatostatin 124
Reaction of EDC 4 and TCEP with somatostatin 125
Reaction of CDMP 8 and TCEP with somatostatin 126
References 127

General experimental

All chemical reagents were purchased from Alfa Aesar, Acros, Fluorochem, Fischer Scientific, Sigma Aldrich, VWR. Compounds and solvents were used as received unless stated otherwise. Reactions were monitored using thin layer chromatography (TLC) on pre-coated silica gel plates (254 µm). Experiments were obtained using Bruker NMR instruments (Avance III 400, Avance 500) at 25 °C; ^{1}H NMR spectra were obtained at 400 MHz or 500 MHz; ^{13}C NMR spectra were obtained at 100 MHz. ^{31}P NMR spectra were obtained at 160 MHz. Chemical shifts (δ) for ^{1}H NMR, ^{13}C NMR, and ^{31}P NMR are quoted in parts per million (ppm) calibrated using residual signals of the solvent. Where amide rotamers are the case, and when possible, only the chemical shift of the major rotamer has been assigned and areas underneath all rotameric peaks have been considered for the integral intensity calculations. Coupling constants (J values) are reported in Hertz (Hz) and are reported as $J_{H\text{-}H}$ couplings between protons. Infrared spectra were obtained on a Bruker FTIR spectrometer operating in ATR mode. High resolution mass spectra were obtained at UCL Chemistry Mass Spectrometry Facility. Optical rotations were measured on Bellingham + Stanley ADP430 Polarimeter at 25 °C. Specific optical rotation, $[\alpha]_D$, values are reported in 10^{-1} ° cm^{2} g^{-1}.

LCMS analysis for peptides and small molecules

LCMS was performed using Waters Acquity UPLC connected to Waters Acquity Single Quad Detector. Separations were performed on Column: XBridge OST C18 Column, 130Å, 2.5 µm, 2.1 mm X 50 mm at a flow rate of 0.6 mL/min; with UV detection wavelength set at 214 nm; with a mobile phase consisting of 0.1% formic acid in water (solvent A) and 0.1% formic acid in acetonitrile (solvent B). Gradient [time/min](solvent A:solvent B): [0-0.5](95:5). [0.5-3.5](95:5-5:95). [3.5-4](5:95). [4-4.5](5:95-95:5). [4.5-5](95:5).

Organic synthesis

Synthesis of *N*-Ac-Cys(trt)-Gly-OtBu S1

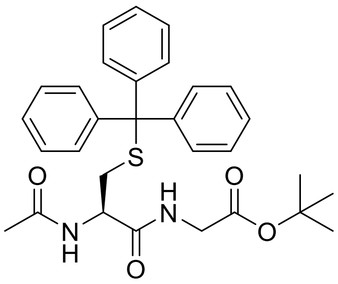

To a flame dried RBF was added *N*-Ac-*S*-trityl-Cys-OH (810 mg, 2 mmol), HOBt (540 mg, 4.0 mmol), H-Gly-OtBu (293 mg, 3.0 mmol) in dry DMF (6 mL), and DIPEA (774 mg, 1.04 mL, 6.0 mmol). EDC (764 mg, 4.0 mmol) was added in one portion. Reaction was stirred at room temperature under argon for 24 h. Reaction mixture was partitioned between 10% (w/v) aqueous LiCl (100 mL) and EtOAc (100 mL). Organic layer was extracted, washed with more 10% (w/v) aqueous LiCl (2×100 mL), dried over $MgSO_4$, and evaporated. Crude was taken up in EtOAc and acetone and adsorbed onto Celite and purified using column chromatography on Biotage (Flashpure Ecoflex 25 g silica, 100:0-50:50 cHex:EtOAc). Appropriate fractions were pooled and solvent was co-evaporated with DCM yielding product as a white solid (570 mg, 1.1 mmol, 55%).

^{1}H NMR (400 MHz, $CDCl_3$) δ 1.44 (s, 9H, $C(CH_3)_3$), 1.89 (s, 3H, *N*-Ac), 2.67 (ddd, *J* = 81.1, 13.1, 6.2 Hz, 2H, *S*-CH_2), 3.83 (qd, *J* = 18.3, 5.0 Hz, 2H, Gly-αCH_2), 4.17 (q, *J* = 6.7 Hz, 1H, Cys-αCH), 5.82 (s, 1H, Cys-NH), 6.53 (s, 1H, Gly-NH), 7.31 (m, 15H, *S*-trt). ^{13}C NMR (100 MHz, $CDCl_3$) δ 23.2 (CH_3), 28.1 (CH_3), 33.2 (CH_2), 42.2 (CH_2), 52.1 (CH), 67.3 (C), 82.4 (C), 127.0 (CH), 128.2 (CH), 129.6 (CH), 144.5 (C), 168.4 (C), 170.1 (C), 170.3 (C). IR (thin film) Vmax/cm^{-1} 3284, 1741, 1650, 1594, 1533. HRMS (ESI+) m/z calculated for $[C_{30}H_{34}O_4N_2{}^{32}S^{23}Na]^+$ 541.2131, observed 541.2115.

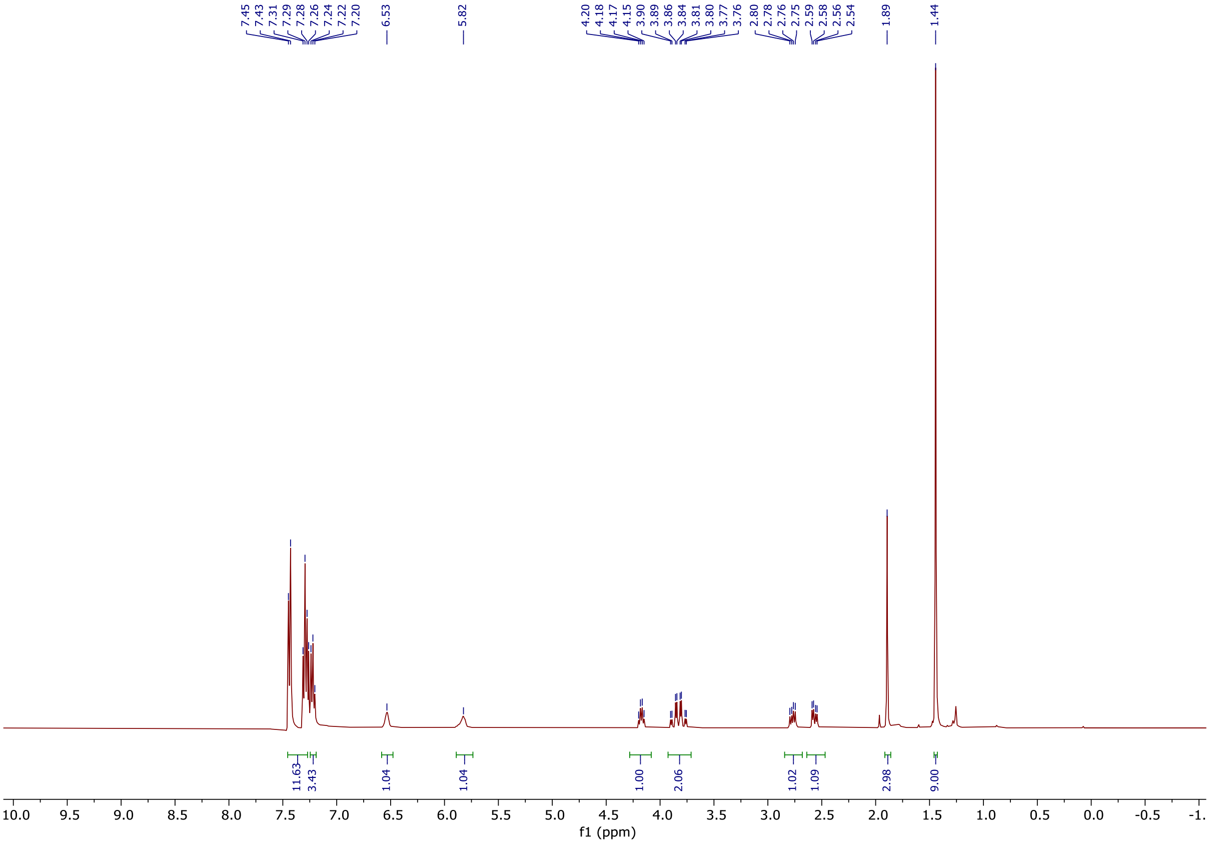

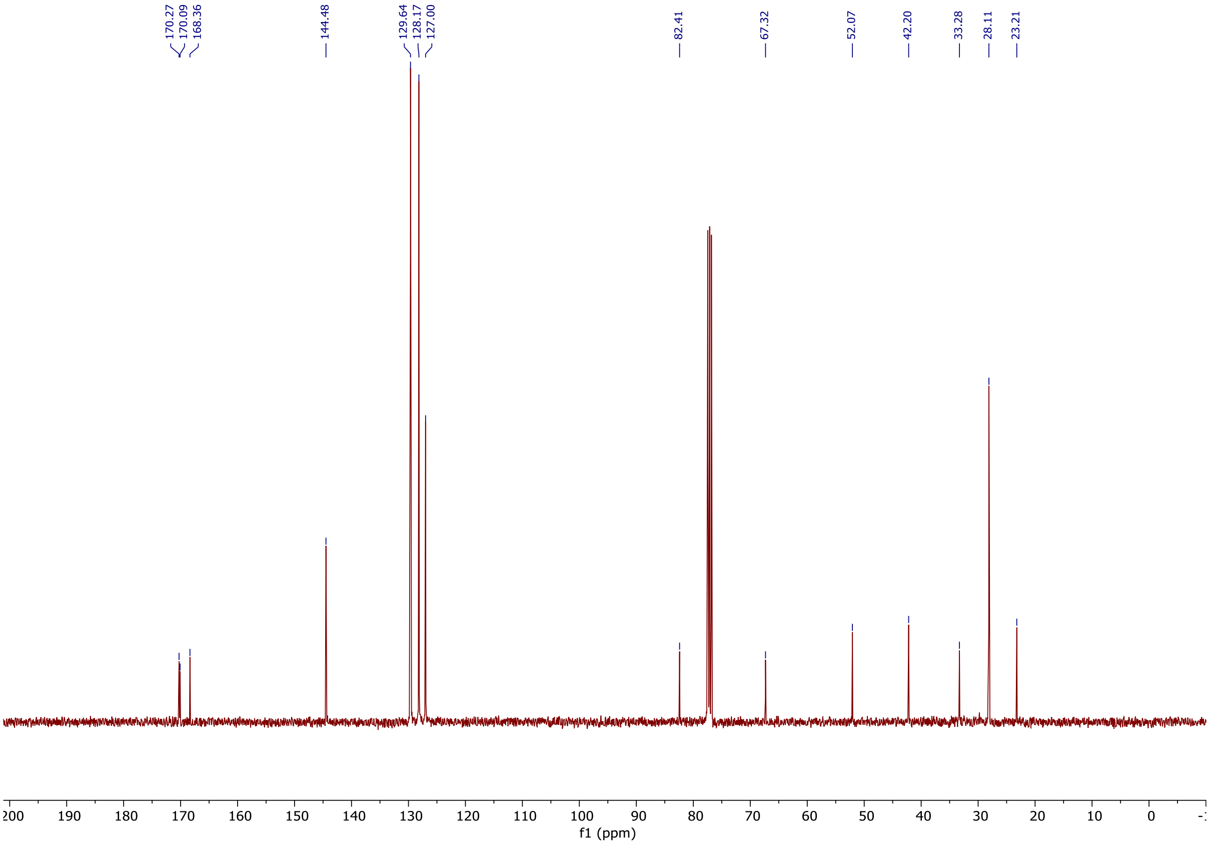

Figure S 1 ^{1}H and ^{13}C NMR for *N*-Ac-Cys(trt)-Gly-OtBu.

Synthesis of *N*-Ac-Cys-Gly-OH S2

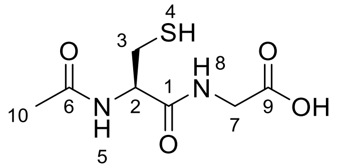

Deprotection cocktail was made containing trifluoroacetic acid (TFA, 1410 µL), H_2O (15 µL), triisopropylsilane (37.5 µL), EDT (37.5 µL), DCM (450 µL). Deprotection cocktail (1.5 mL) was added to *N*-Ac-Cys(trt)-Gly-OtBu **S1** (150 mg, 0.29 mmol). Reaction was stirred at room temperature for 2 h. Solvent was co-evaporated with DCM. Crude was partitioned between DCM (10 mL) and water (2 mL). Water layer was extracted and washed with DCM (2×10 mL). Water layer was loaded directly for reverse phase column chromatography on Biotage (C18 12 g, 100:0 – 97:3 water:MeCN with 0.1% TFA). Fractions containing product were combined and freeze dried to yield the product as a white solid (24 mg, 0.11 mmol, 38%).

^{1}H NMR (400 MHz, D_2O) δ 2.07 (s, 3H, $CH_3$10), 2.92 (d, *J* = 6.0 Hz, 2H, $CH_2$3), 4.00 (s, 2H, $CH_2$7), 4.54 (t, *J* = 6.0 Hz, 1H, CH2). ^{13}C NMR (100 MHz, D_2O) δ 22.3 (CH_3), 25.9 (CH_2), 41.7 (CH), 56.1 (CH), 173.1 (C), 173.7 (C), 175.0 (C). IR (solid) Vmax/cm^{-1} 3311, 1723, 1650, 1619, 1536. HRMS (ESI+) m/z calculated for $[C_7H_{13}O_4N_2{}^{32}S]^+$ 221.0590, observed 221.0588. $[\alpha]_D$ = -18.2 (c 0.55 mg mL^{-1} in H_2O).

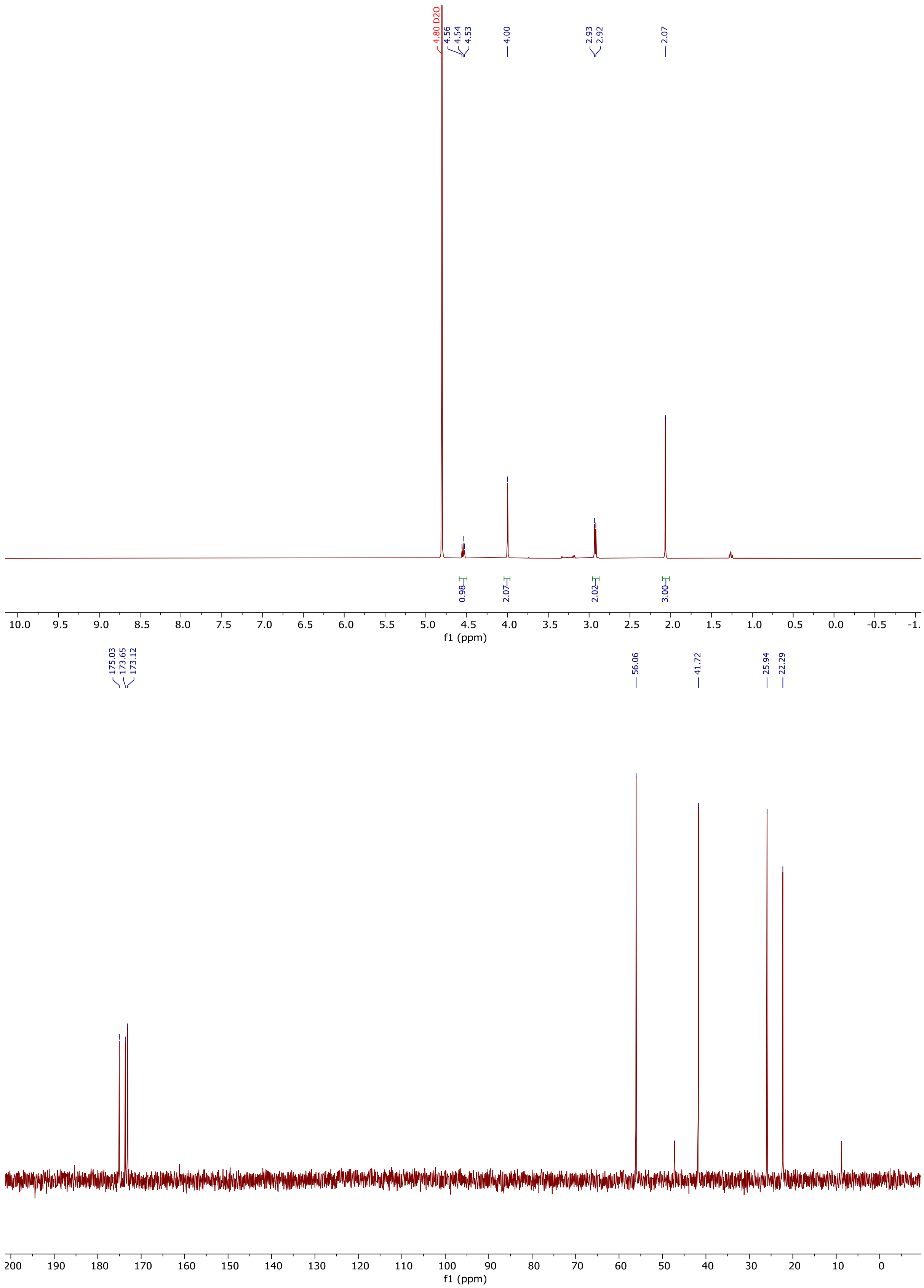

Figure S 2 ^{1}H and ^{13}C NMR for *N*-Ac-Cys-Gly-OH.

Synthesis of *N*-Ac-Cys(trt)-Pro-OtBu S3

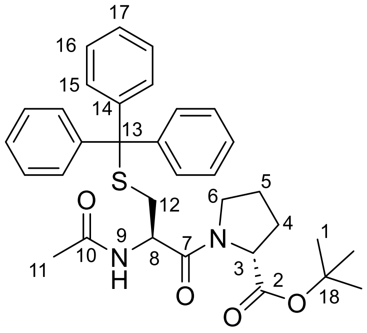

To a flame dried round bottom flask was added *N*-Ac-*S*-trityl-Cys-OH (810 mg, 2.0 mmol), EDC.HCl (382 mg, 2.0 mmol), HOBt (270 mg, 2.0 mmol), H-Pro-OtBu (513 mg, 3 mmol) in dry DMF (6 mL), and DIPEA (516 mg, 695 µL, 4 mmol). Reaction was stirred at room temperature under argon for 18 h. Reaction mixture was partitioned between 10% (w/v) aqueous LiCl (100 mL) and EtOAc (100 mL). Organic layer was extracted, washed with more 10% (w/v) aqueous LiCl (2×100 mL), dried over $MgSO_4$, and evaporated. Crude was taken up in DCM and purified using column chromatography on Biotage (Flashpure Ecoflex 25 g silica, 100:0-50:50 cHex:EtOAc). Appropriate fractions were pooled and solvent was evaporated under reduced pressure to yield product as a waxy solid (390 mg, 0.67 mmol, 35%).

^{1}H NMR (400 MHz, $CDCl_3$, rotamers) δ 1.40 (m, 9H, $(CH_3)_3$1), 1.92 (m, 6H, $CH_2$4, $CH_2$5, $CH_3$11), 2.09 (m, 1H, $CH_2$4), 2.55 (m, 2H, $CH_2$12), 3.05 (m, 1H, $CH_2$6), 3.46 (m, 1H, $CH_2$6), 4.25 (m, 1H, CH3), 4.60 (m, 1H, CH8), 5.95 (d, *J* = 8.7 Hz, 1H, NH9), 7.30 (m, 15H, CH14-17). ^{13}C NMR (100 MHz, $CDCl_3$, rotamers) δ 23.3 ($CH_3$11), 24.8 ($CH_2$5), 28.0 ($CH_3$1), 29.1 ($CH_2$4), 34.0 ($CH_2$12), 46.8 ($CH_2$6), 50.0 (CH8), 60.0 (CH3), 67.3 (C13), 81.3 (C18), 126.9 (Ph-CH), 128.1 (Ph-CH), 129.8 (Ph-CH), 144.6 (Ph-C14), 169.0 (C), 169.8 (C), 170.6 (C). IR (thin film) Vmax/cm^{-1} 3286, 2975, 1736, 1629, 1596, 1532, 1442. HRMS (ESI+) m/z calculated for $[C_{33}H_{38}O_4N_2{}^{32}S^{23}Na]^+$ 581.2444, observed 581.2428.

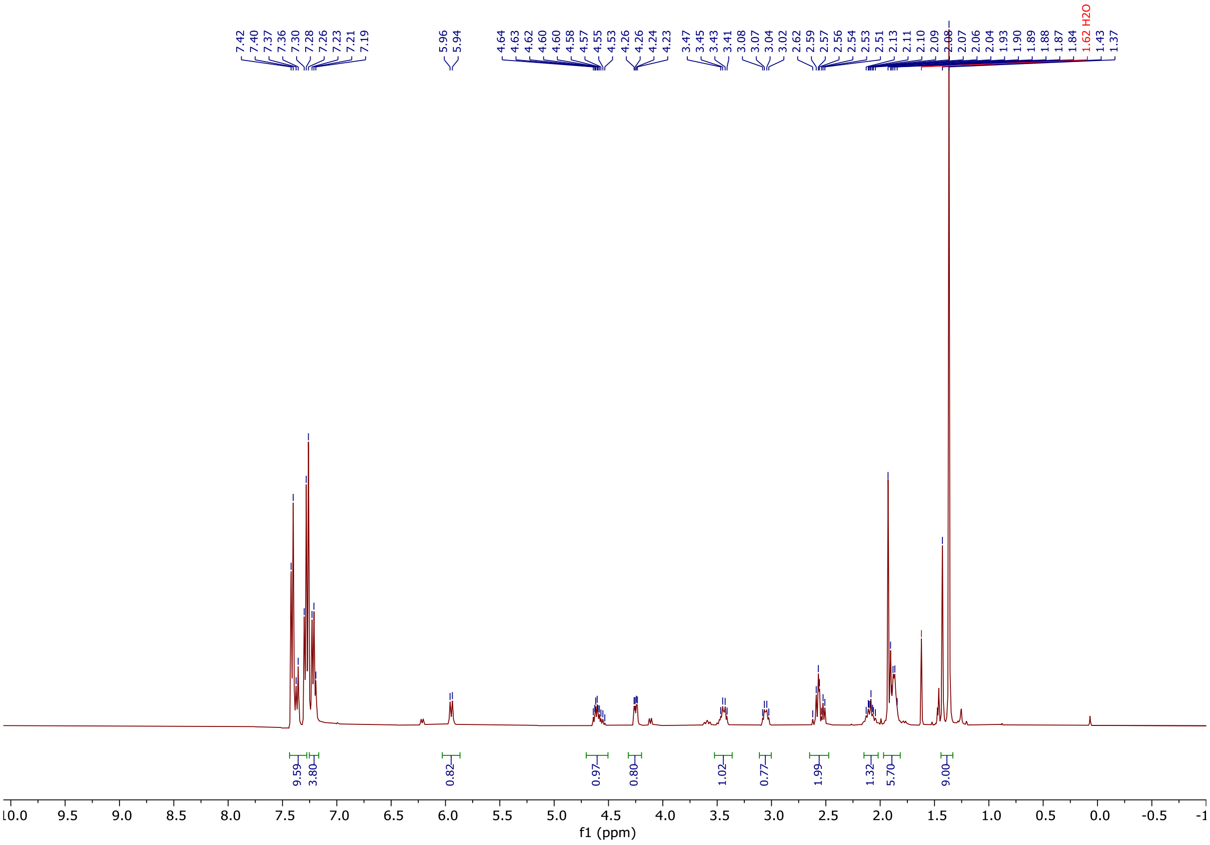

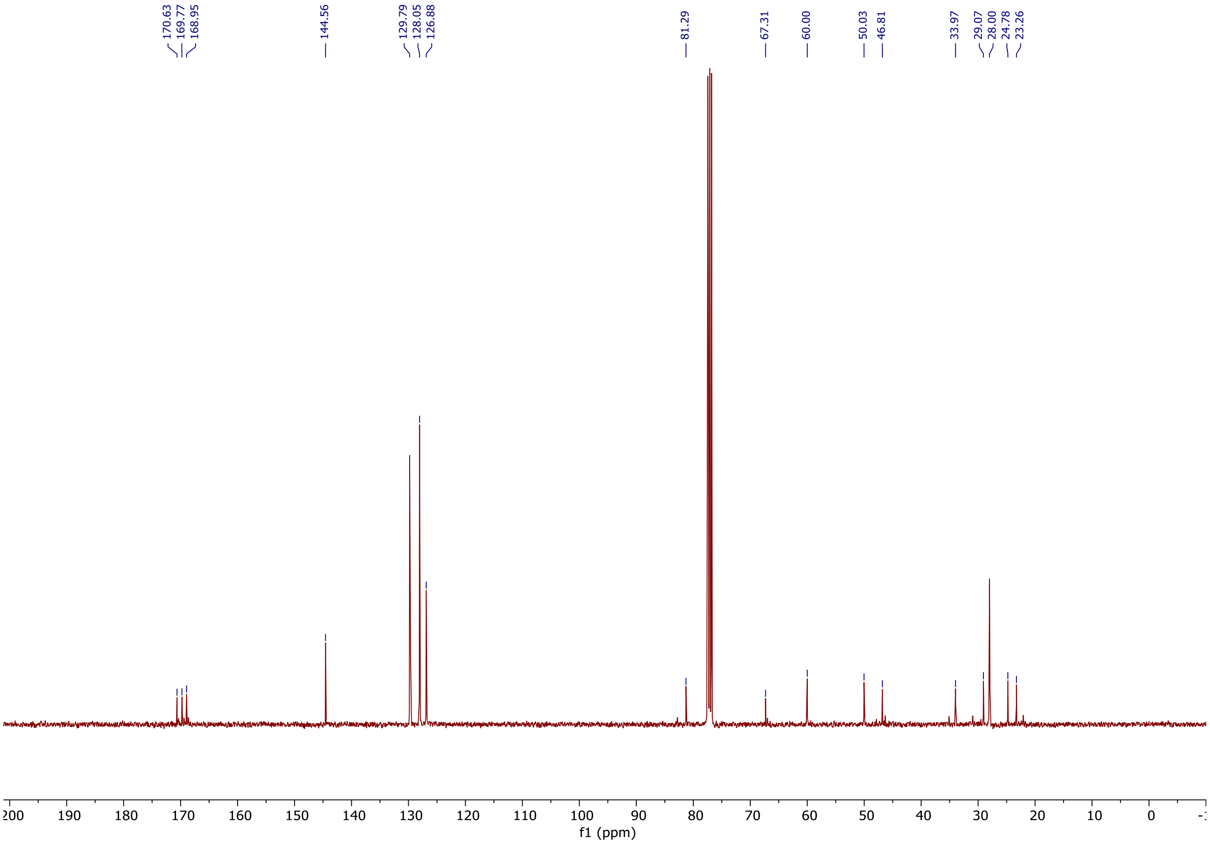

Figure S 3 ^{1}H and ^{13}C NMR for *N*-Ac-Cys(trt)-Pro-OtBu

Synthesis of *N*-Ac-Cys-Pro-OH S4

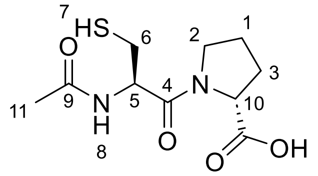

Deprotection cocktail was made containing trifluoroacetic acid (TFA, 1410 µL), H_2O (15 µL), TIS (37.5 µL), EDT (37.5 µL), DCM (650 µL). Deprotection cocktail (2.15 mL) was added to *N*-Ac-Cys(trt)-Pro-OtBu **S3** (140 mg, 0.25 mmol). Reaction mixture was stirred at room temperature for 3 h. Solvent was removed under reduced pressure. Crude was partitioned between DCM (5 mL) and water (3 mL). Water layer was extracted and washed with more DCM (2 ×5 mL). Water layer was loaded directly for reverse phase column chromatography on Biotage (C18 12 g, 100:0 – 80:20 water:MeCN with 0.1% TFA). Fractions containing product were combined and freeze dried to yield the product as a white solid (14 mg, 5.3 µmol, 22%).

^{1}H NMR (400 MHz, D_2O) δ 2.00 (m, 6H, $CH_3$11, $CH_2$2, $CH_2$1), 2.32 (m, 1H, $CH_2$2), 2.86 (m, 2H, $CH_2$3), 3.79 (m, 2H, $CH_2$6), 4.44 (dd, *J* = 8.3, 4.5 Hz, 1H, CH5), 4.76 (t, *J* = 6.6 Hz, 1H, CH10). ^{13}C NMR (100 MHz, D_2O, rotamers) δ 22.0 (CH_3), 25.0 (CH_2), 25.1 (CH_2), 29.4 (CH_2), 48.4 (CH_2), 54.2 (CH), 60.2 (CH), 171.0 (C), 174.6 (C), 176.3 (C). IR (solid) Vmax/cm^{-1} 3329, 2543, 1733, 1640, 1592. HRMS (ESI+) m/z calculated for $[C_{10}H_{17}O_4N_2{}^{32}S]^+$ 261.0903, observed 261.0895. $[\alpha]_D$ = -82.1 (c 0.95 mg mL^{-1} in H_2O).

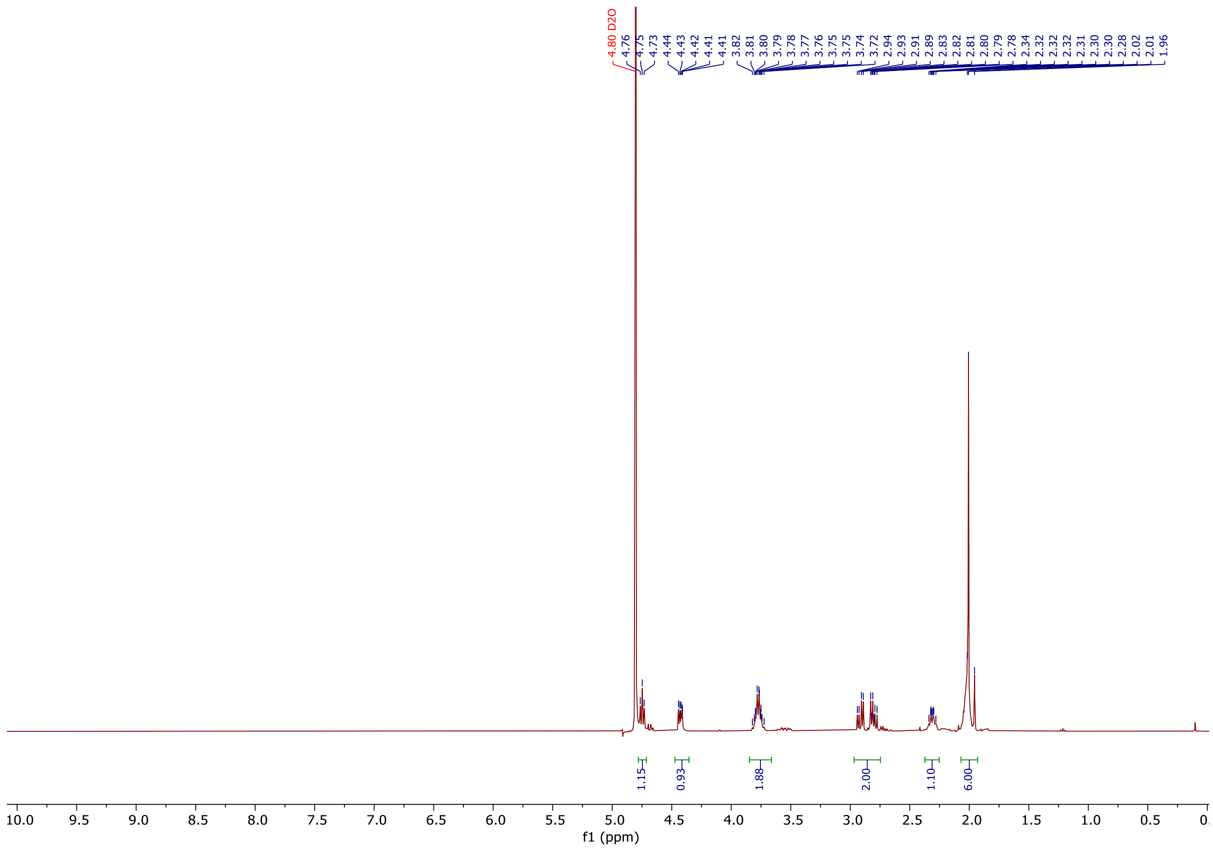

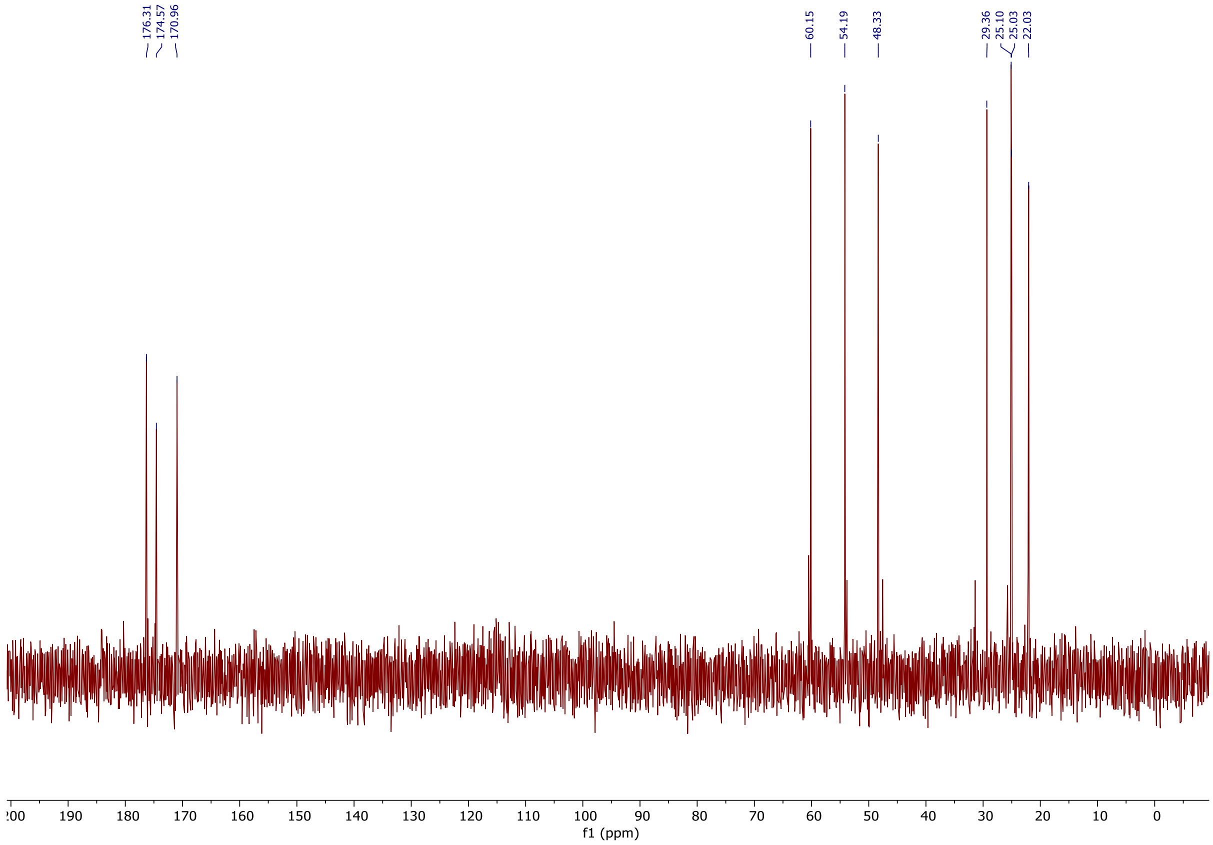

Figure S 4 ^{1}H and ^{13}C NMR for *N*-Ac-Cys-Pro-OH

Synthesis of *N*-Ac-Cys(trt)-Asp-OtBu S105

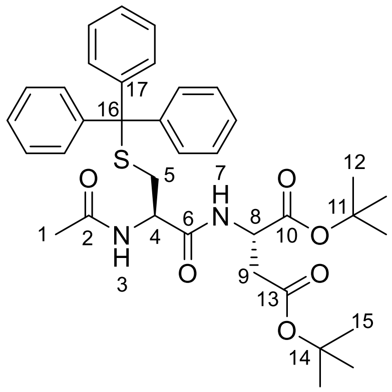

To a flame dried round bottom flask was added *N*-Ac-*S*-trityl-Cys-OH (810 mg, 2.0 mmol), EDC.HCl (382 mg, 2.0 mmol), HOBt (270 mg, 2.0 mmol), H-Asp(OtBu)-OtBu (564 mg, 2.0mmol) in dry DMF (6 mL), and DIPEA (516 mg, 695 µL, 4 mmol). Reaction was stirred at room temperature under argon for 18 h. Reaction mixture was partitioned between 10% (w/v) aqueous LiCl (100 mL) and EtOAc (100 mL). Organic layer was extracted, washed with more 10% (w/v) aqueous LiCl (2×100 mL), dried over $MgSO_4$, evaporated, and purified using column chromatography on Biotage (Flashpure Ecoflex 25 g silica, 50:50 cHex:EtOAc). Appropriate fractions were pooled and solvent was evaporated under reduced pressure to yield product as a white solid (730 mg, 1.2 mmol, 58%).

^{1}H NMR (400 MHz, $CDCl_3$, rotamers) δ 1.42 (m, 18H, $C(CH_3)_3$12), 1.89 (s, 3H,$CH_3$1), 2.69 (m, 4H, $CH_2$5, $CH_2$9), 4.14 (m, 1H, CH4), 4.56 (m, 1H, CH8), 5.67 (d, *J* = 7.8 Hz, 1H, NH3), 6.82 (d, *J* = 8.0 Hz, 1H, NH7), 7.32 (m, 15H, ArCH).^{13}C NMR (100 MHz, $CDCl_3$, rotamers) δ 23.2 (C1), 28.0 (C12/15), 28.1 (C12/15), 33.7 (C5/9), 37.3 (C5/9), 49.4 (C8), 52.1 (C4), 67.3 (C16), 81.6 (C11/14), 82.4 (C11/14), 127.0 (ArCH), 128.2 (ArCH), 129.7 (ArCH), 144.5 (C17), 169.2, 169.6, 169.8, 170.0. IR (solid) Vmax/cm^{-1} 3287, 2978, 2930,1729, 1647, 1520, 1444. HRMS (ESI+) m/z calculated for $[C_{36}H_{45}O_6N_2{}^{32}S]^+$ 633.2993, observed 633.3017.

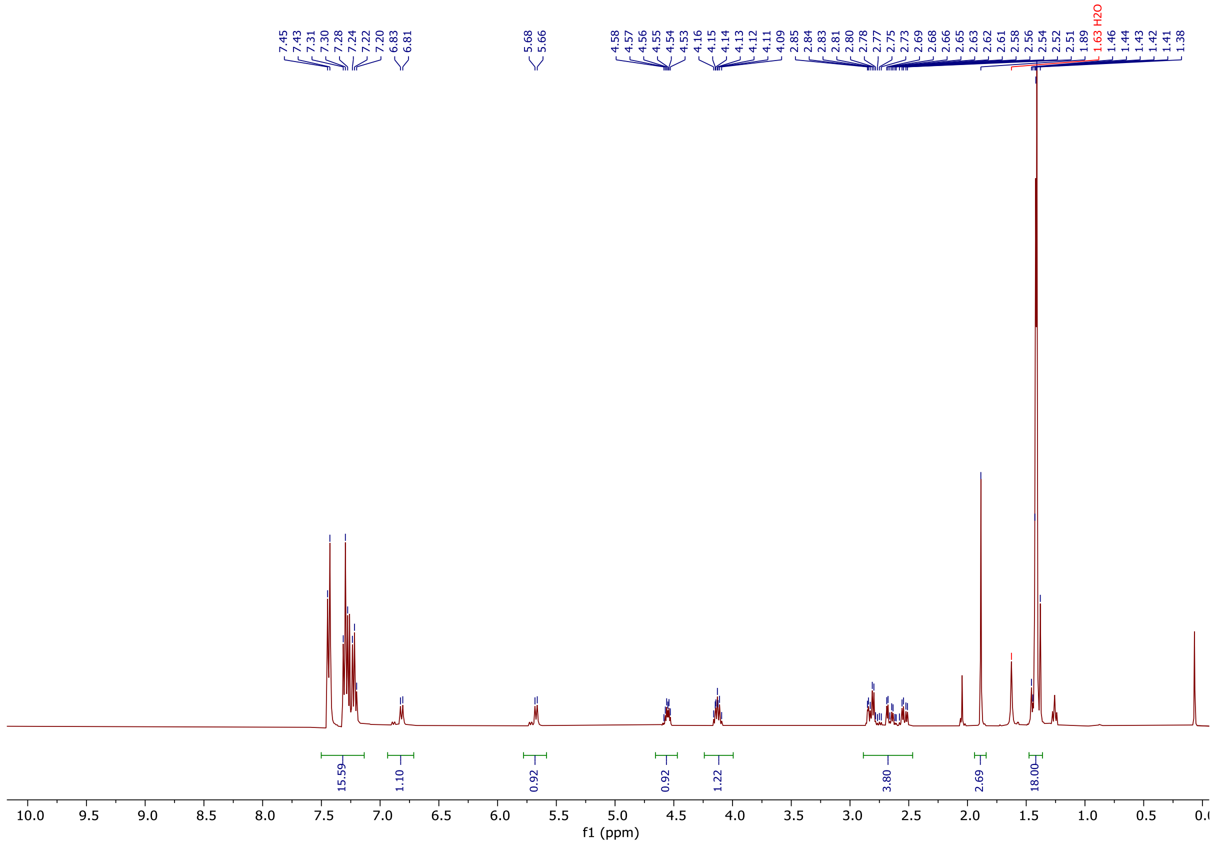

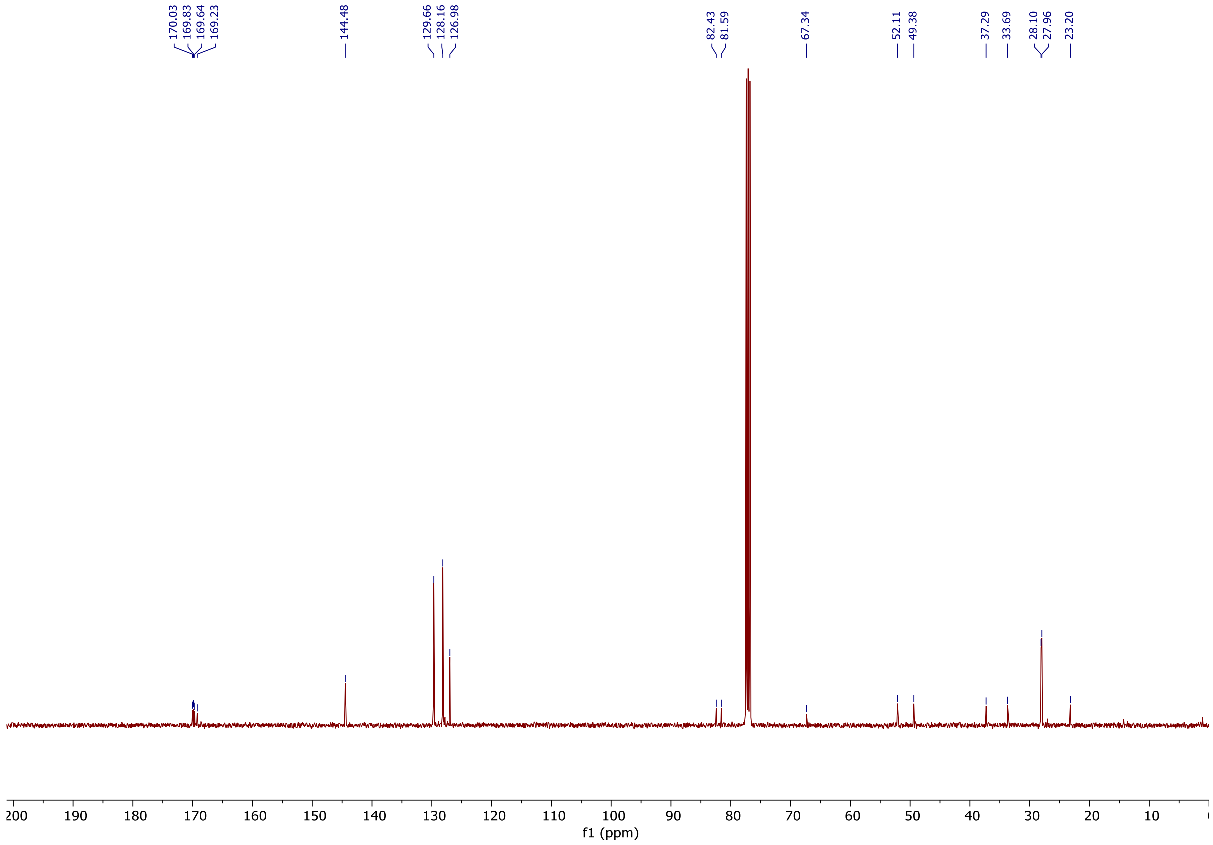

Figure S 5 ^{1}H and ^{13}C NMR for *N*-Ac-Cys(trt)-Asp-OtBu.

Synthesis of *N*-Ac-Cys-Asp-OH S106

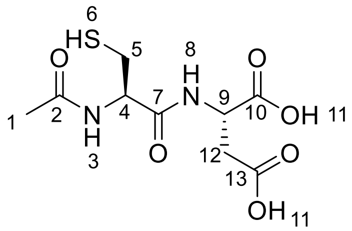

Deprotection cocktail was made containing trifluoroacetic acid (TFA, 2.8 mL), H_2O (30 µL), TIS (613 µL, 479 mg, 3 mmol), EDT (75 µL, 84 mg, 90 mmol), DCM (1.3 mL). Deprotection cocktail was added to *N*-Ac-Cys(trt)-Asp-OtBu **S105** (379 mg, 0.6 mmol). Reaction mixture was stirred at room temperature for 2 h. Solvent co-evaporated with DCM (3×10 mL) under reduced pressure. Resulting white solid was triturated with diethyl ether, precipitate collected *via* filtration, and purified via column chromatography (C18 12 g, 100:0-95:5 water:MeCN with 0.1% TFA). Fractions containing product were combined and freeze dried to yield the product as a white solid (80 mg, 0.29 mmol, 48%).

^{1}H NMR (400 MHz, DMSO, rotamers) δ 1.86 (s, 3H, $CH_3$1), 2.27 (t, J = 8.5 Hz, 1H, SH6), 2.66 (m, 4H, $CH_2$12, $CH_2$5), 4.40 (m, 1H, CH4), 4.51 (q, J = 6.7 Hz, 1H, CH9), 8.12 (d, J = 8.4 Hz, 1H, NH3), 8.36 (d, J = 8.0 Hz, 1H, NH8), 9.90 (bs, 2H, CO_2H11). ^{13}C NMR (100 MHz, DMSO, rotamers) δ 22.5 (C1), 26.4, 35.8, 48.7 (C9), 54.7 (C4), 169.4, 169.9, 171.6, 172.2. IR (solid) Vmax/cm^{-1} 2934, 2560, 1717,1646, 1533. HRMS (ESI+) m/z calculated for $[C_9H_{15}O_6N_2{}^{32}S]^+$ 279.0645, observed 279.0642. $[\alpha]_D$ = -18.0 (c 1.00 mg mL^{-1} in H_2O).

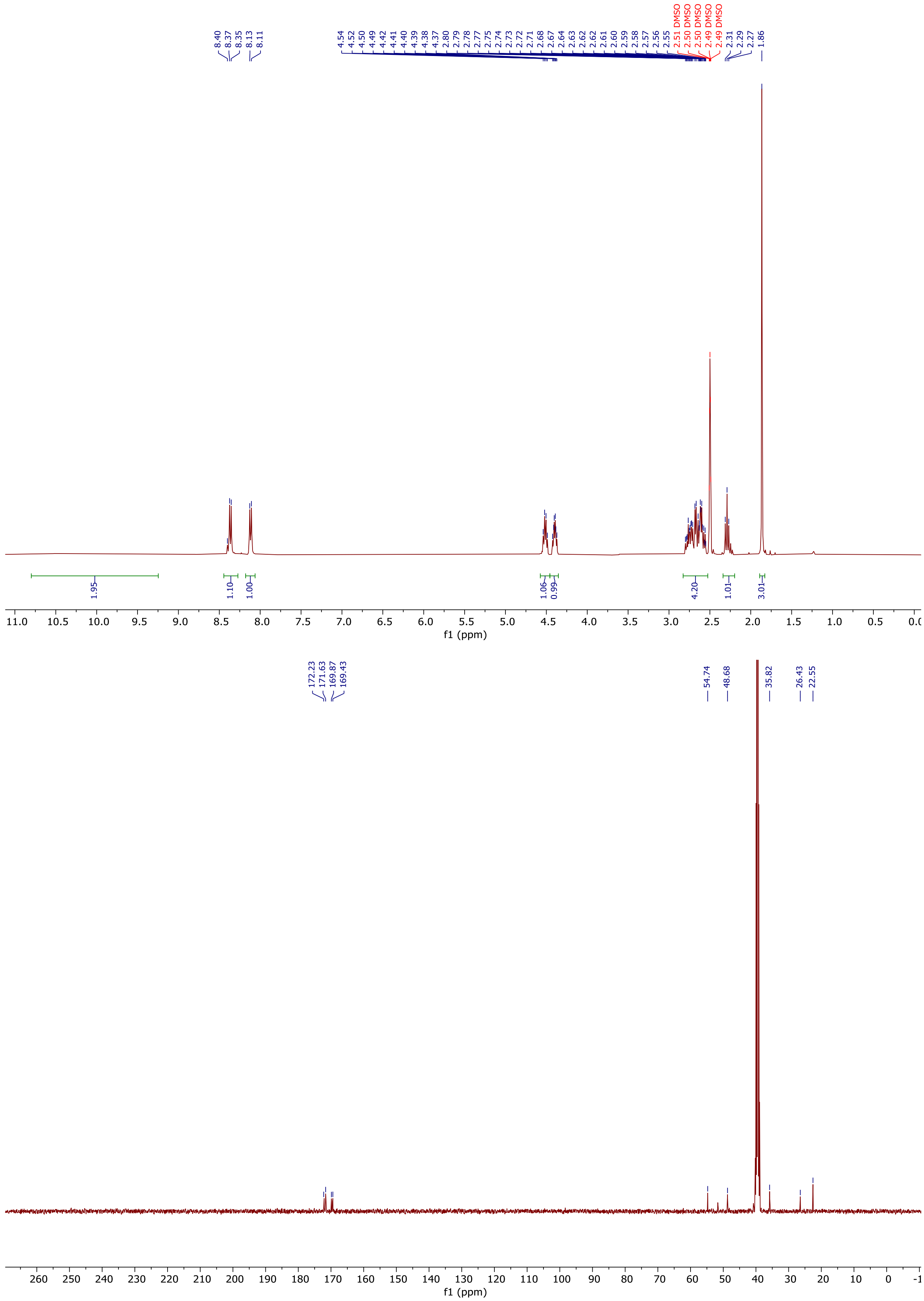

Figure S 6 ^{1}H and ^{13}C NMR for *N*-Ac-Cys-Asp-OH.

Synthesis of 3,3',3''-phosphanetriyltris(*N*-(prop-2-yn-1-yl)propanamide) S5

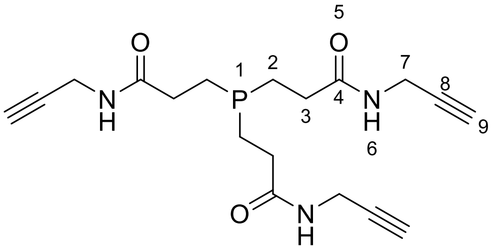

To a stirred reaction vial was added TCEP (72 mg, 0.25 mmol), HATU (342 mg, 0.90 mmol), and DCM (5 mL). To the reaction mixture was added propargyl amine (55 mg, 64 µL, 1.0 mmol) and DIPEA (232 mg, 313 µL, 1.8 mmol). Reaction mixture was stirred for 3 h at room temperature. Reaction was filtered and the residue was washed with DCM (10 mL) and then MeCN (10 mL). The residue was dried under vacuum to yield the product as a white solid (72 mg, 0.20 mmol, 80%).

^{1}H NMR (400 MHz, DMSO) δ 1.58 (t, *J* = 8.2 Hz, 6H, $CH_2$2), 2.16 (q, *J* = 8.3 Hz, 6H, $CH_2$3), 3.09 (d, *J* = 2.7 Hz, 3H, CH9), 3.84 (dd, *J* = 5.5, 2.5 Hz, 6H, $CH_2$7), 8.28 (t, *J* = 5.6 Hz, 3H, NH6). ^{13}C NMR (100 MHz, DMSO) δ 21.5 ($CH_2$2), 27.9 ($CH_2$7), 31.3 ($CH_2$3), 73.0 (CH9), 81.2 (C8), 171.7 (C4). ^{31}P NMR (160 MHz, DMSO) δ -27.1. IR (solid) Vmax/cm^{-1} 3283, 1639, 1539. HRMS (ESI+) m/z calculated for $[C_{18}H_{25}O_3N_3P]^+$ 362.1628, observed 362.1628.

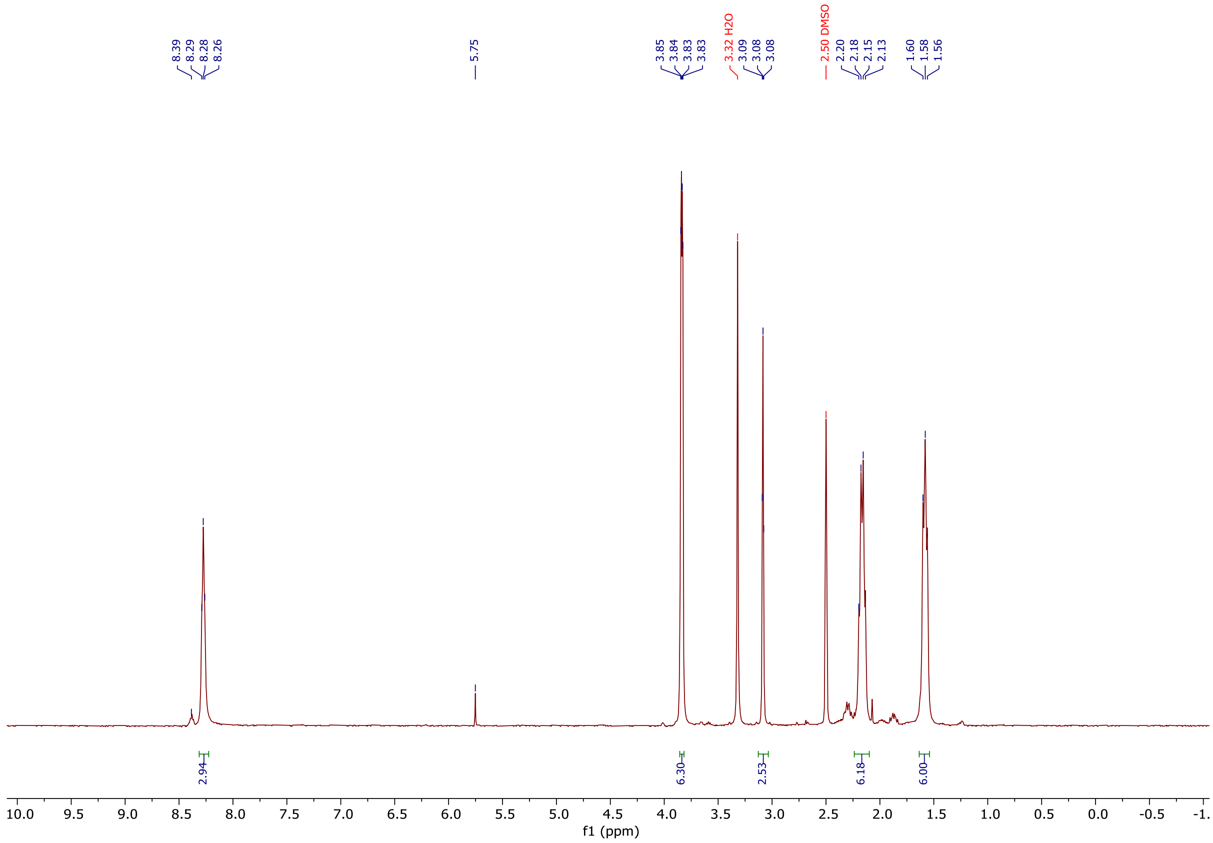
8.39
8.29
8.28
8.26
5.75
3.85
3.84
3.83
3.83
3.32 H2O
3.09
3.08
3.08
2.50 DMSO
2.20
2.18
2.15
2.13
1.60
1.58
1.56
2.94
6.30
2.53
6.18
6.00
f1 (ppm)

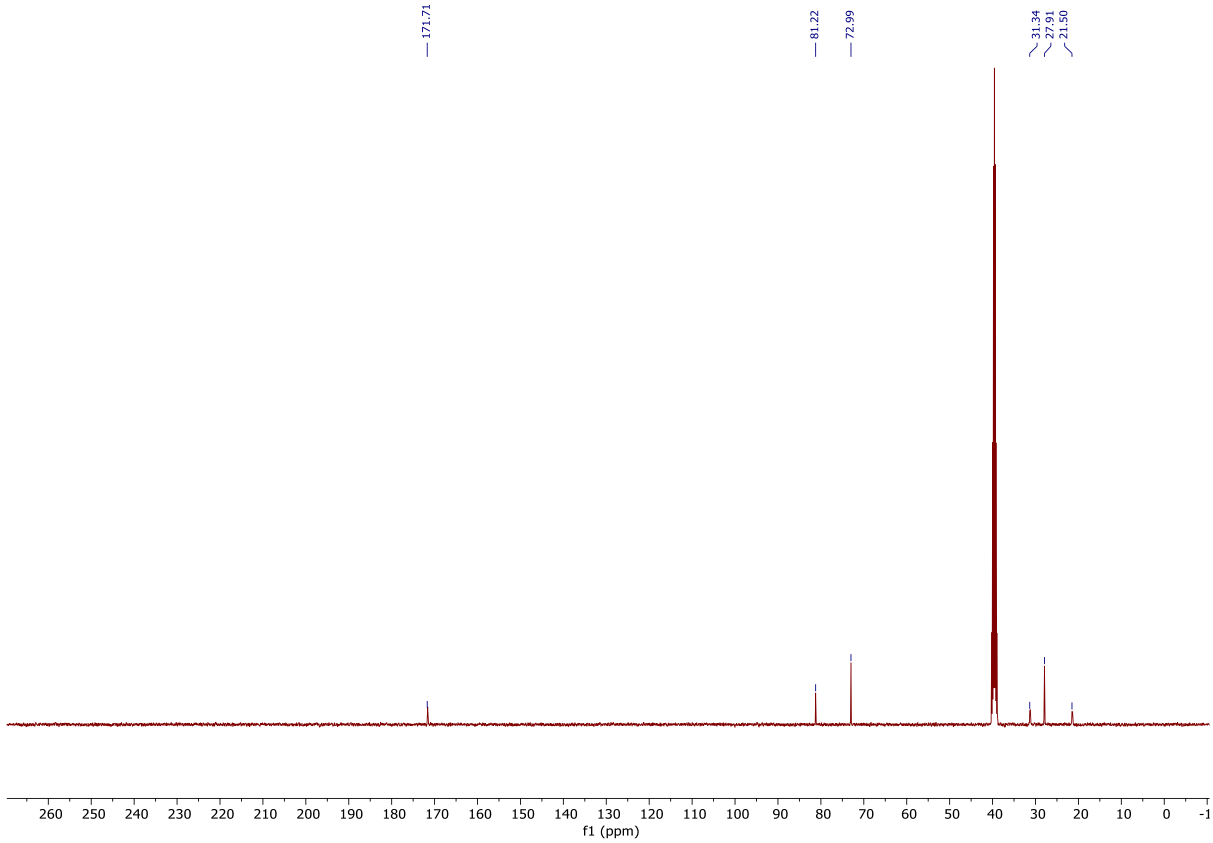
171.71
81.22
72.99
31.34
27.91
21.50
f1 (ppm)

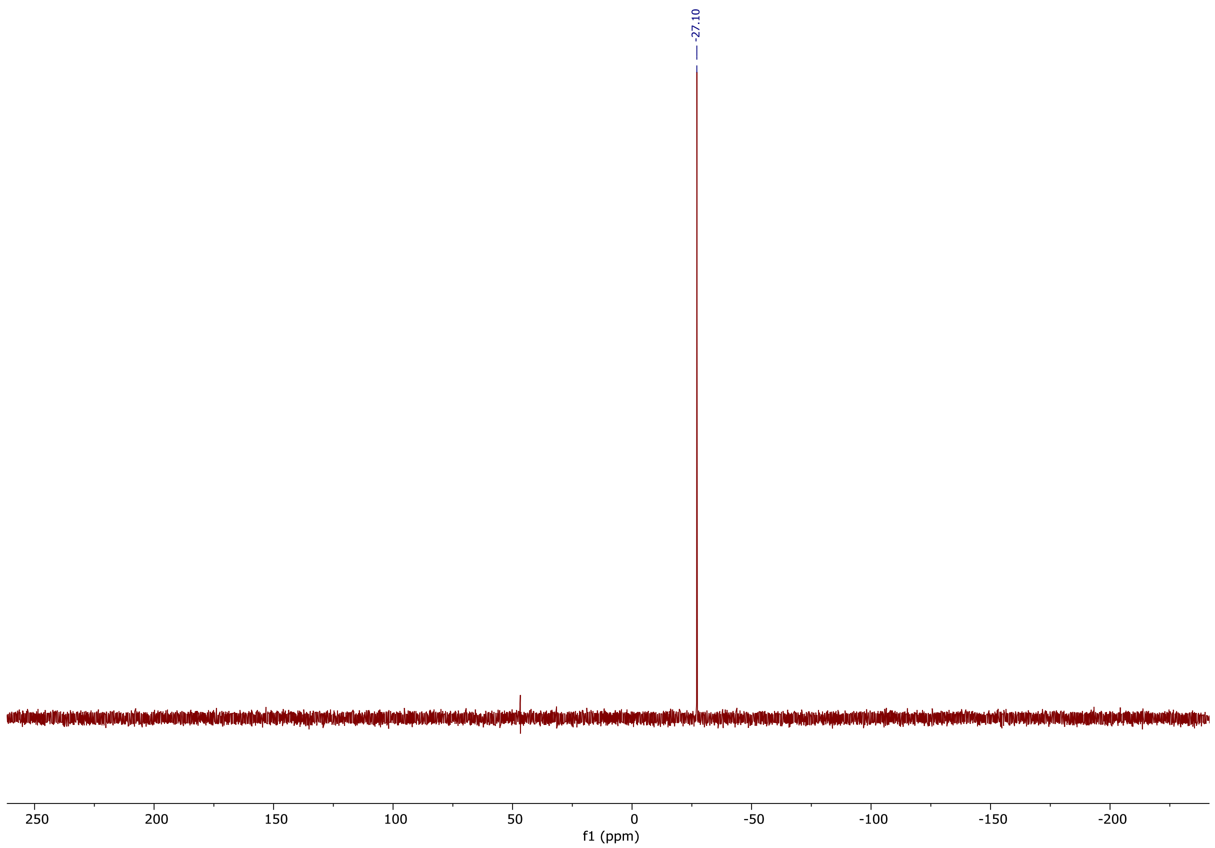
-27.10
250
200
150
100
50
0
-50
-100
-150
-200
f1 (ppm)

Synthesis of 3-(bis(3-methoxy-3-oxopropyl)phosphaneyl)propanoic acid[1] S6

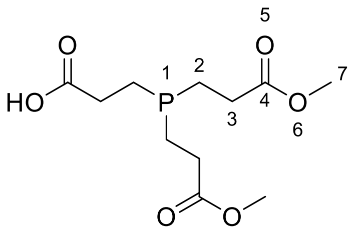

To a reaction vial was added Amberlyst® 15 Hydrogen form (575 mg, dry) and MeOH (10 mL). This mixture was stirred at room temperature for 0.5 h. Amberlyst® was filtered and washed with MeOH. To a separate vial was added TCEP (575 mg, 2.0 mmol), MeOH (10 mL), and the pretreated Amberlyst®. The reaction mixture was stirred at room temperature for 2 h. Amberlyst® was filtered off, and the filtrate was concentrated under reduced pressure. Crude was taken up in DCM and purified using column chromatography on Biotage (Flashpure EcoFlex 12 g, 100:0-96:4 DCM:MeOH). Appropriate fractions were pooled and solvent was evaporated under reduced pressure to yield the product as a clear oil (120 mg, 0.43 mmol, 22%).

^{1}H NMR (400 MHz, MeOD) δ 2.59 (tt, *J* = 12.9, 6.9 Hz, 6H, $CH_2$2), 2.91 (dq, *J* = 19.6, 7.5 Hz, 6H, $CH_2$3), 3.75 (s, 6H, $CH_3$7). ^{13}C NMR (100 MHz, MeOD) δ 16.6 (CH_2), 22.0 (CH_2), 28.5 (CH_2), 53.0 (CH_3), 174.3 (C). ^{31}P NMR (160 MHz, MeOD) δ 18.3. LRMS (ESI+) m/z 279 $[M+H]^+$.

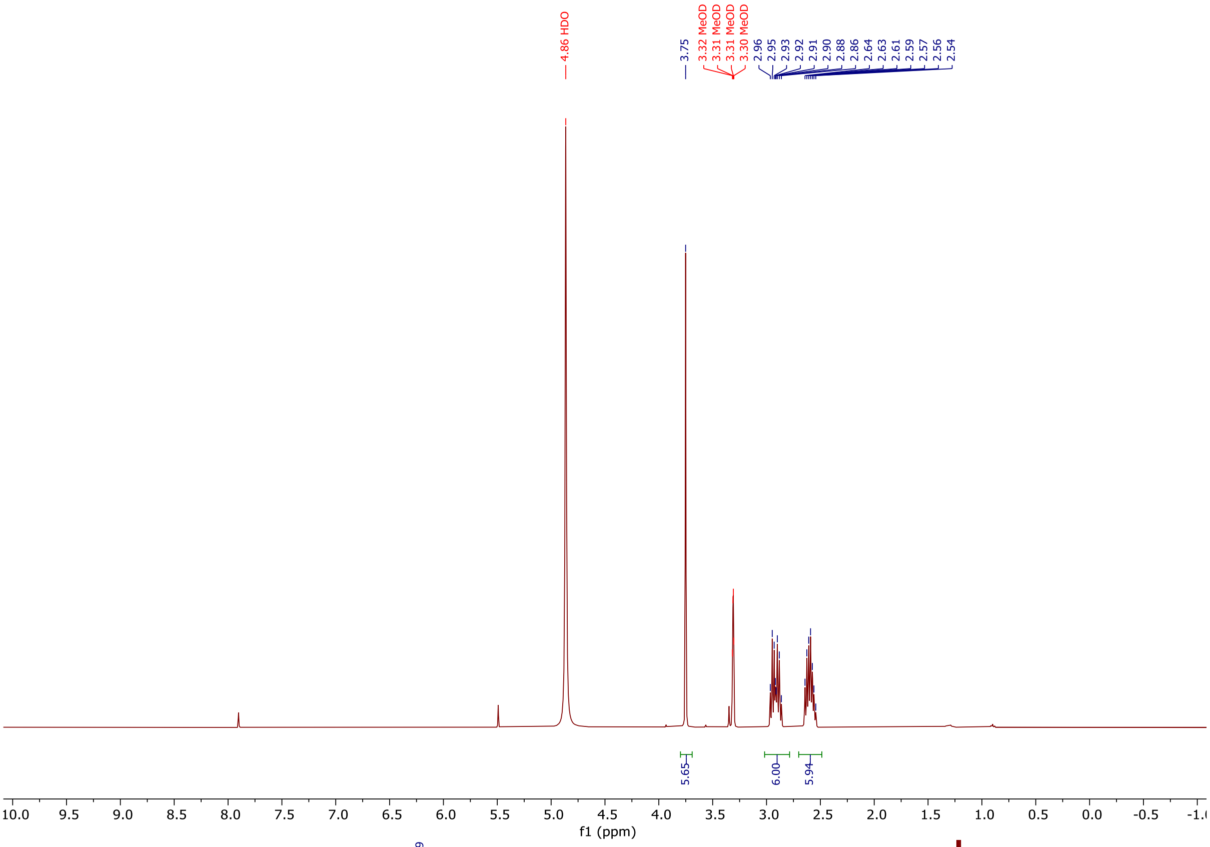
4.86 HDO
3.75
3.32 MeOD
3.31 MeOD
3.31 MeOD
3.30 MeOD
2.96
2.95
2.93
2.92
2.91
2.90
2.88
2.86
2.64
2.63
2.61
2.59
2.57
2.56
2.54
5.65
6.00
5.94
f1 (ppm)

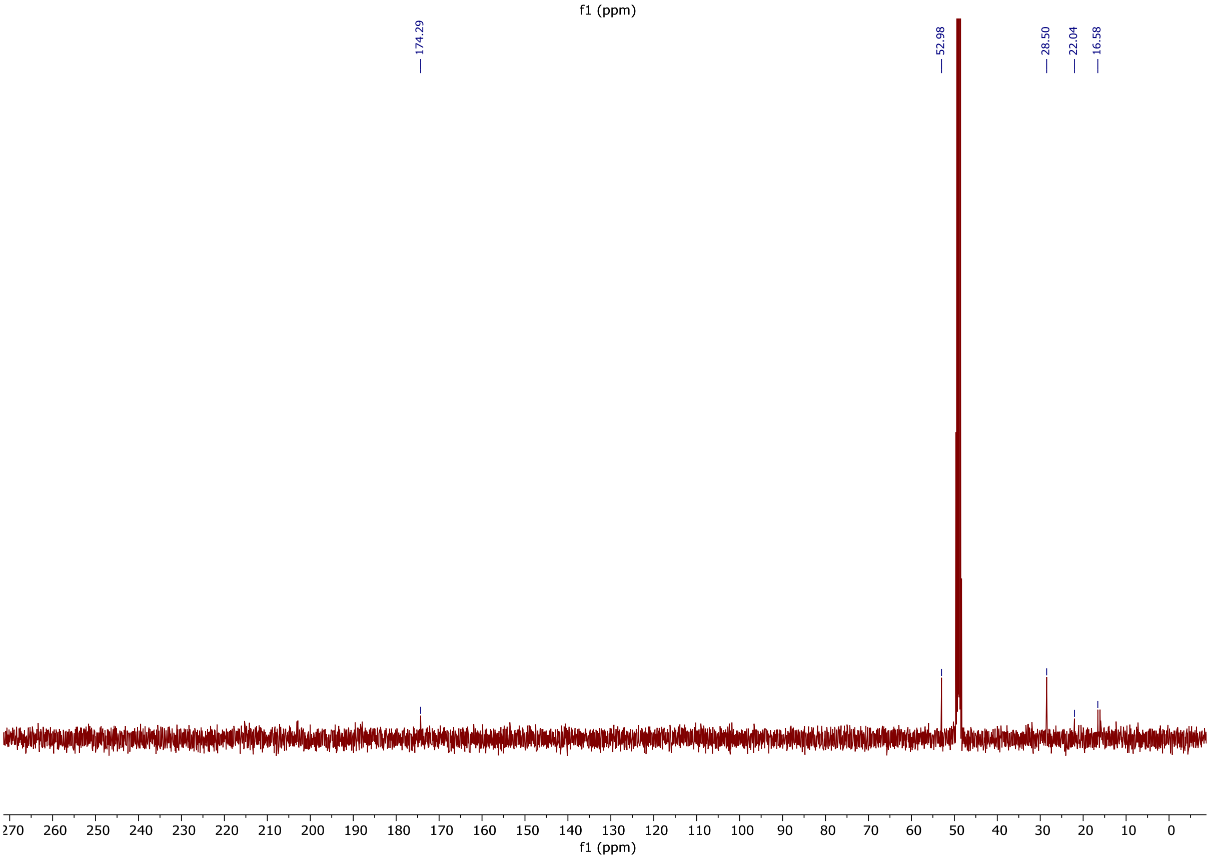
174.29
52.98
28.50
22.04
16.58
f1 (ppm)

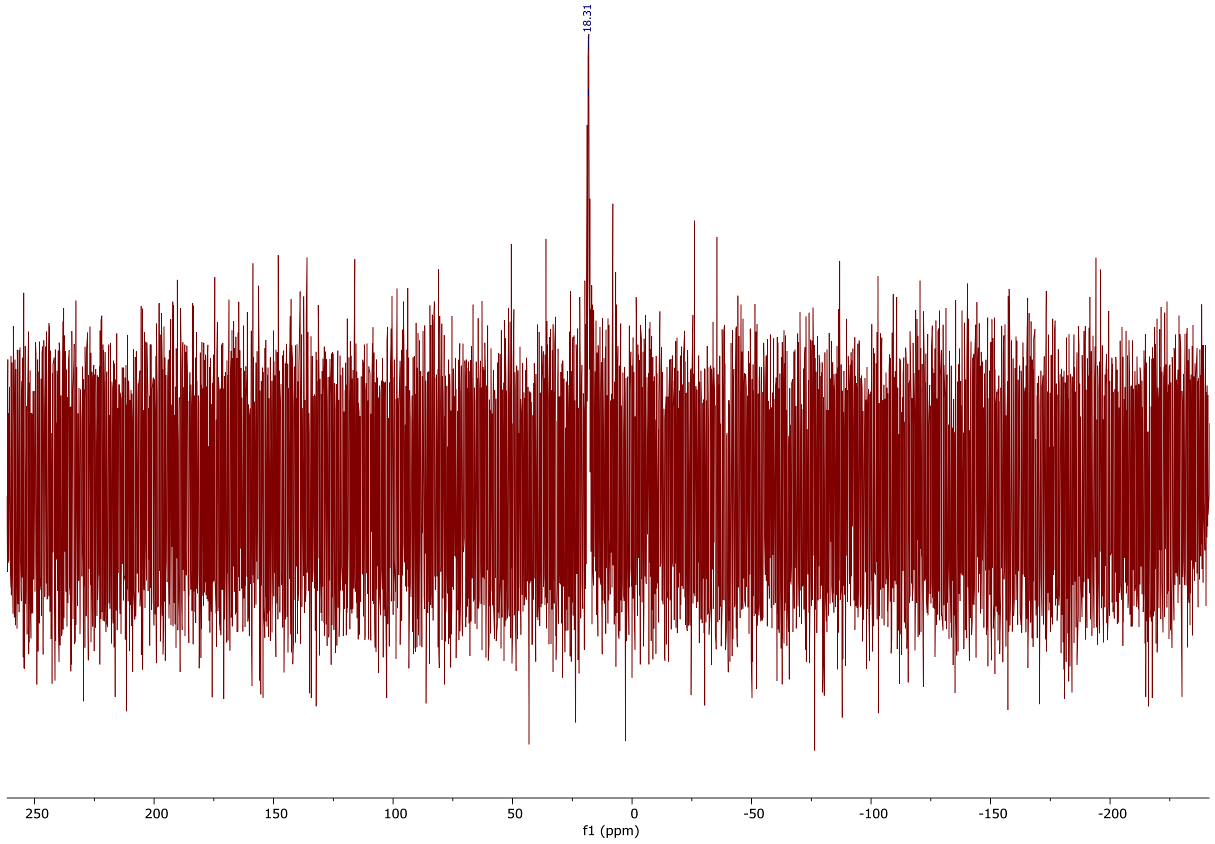
18.31
250
200
150
100
50
0
-50
-100
-150
-200
f1 (ppm)

Synthesis of dimethyl 3,3'-((3-oxo-3-(prop-2-yn-1-ylamino)propyl)phosphanediyl)dipropionate S7

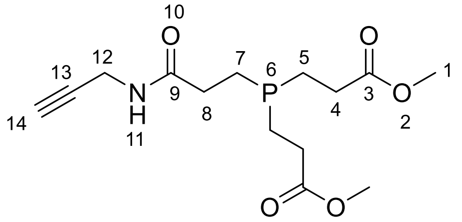

To a solution of 3-(bis(3-methoxy-3-oxopropyl)phosphaneyl)propanoic acid **S6** (29 mg, 0.10 mmol) in dry MeCN (3 mL) was added HATU (76 mg, 0.20 mmol). Reaction mixture was stirred at room temperature for 15 min and was added propargyl amine (12 mg, 14 µL, 0.22 mmol) and DIPEA (28 mg, 38 µL, 0.22 mmol). Reaction mixture was stirred at room temperature for 6 h. Excess solvent was evaporated under reduced pressure. Crude was taken up in DCM and purified using column chromatography on Biotage (Flashpure EcoFlex 4 g, 100:0-98:2 DCM:MeOH). Appropriate fractions were pooled and solvent was evaporated under reduced pressure to yield product as a clear oil (5 mg, 16 µmol, 16%).

^{1}H NMR (400 MHz, $CDCl_3$) δ 1.95 (m, 6H, $CH_2$5, $CH_2$7), 2.23 (q, *J* = 3.2 Hz, 1H, CH14), 2.53 (m, 6H, $CH_2$4, $CH_2$8), 3.70 (m, 6H, $CH_3$1), 4.04 (dd, *J* = 5.3, 2.5 Hz, 2H, $CH_2$12). ^{13}C NMR (100 MHz, $CDCl_3$, rotamers) δ 21.3 ($CH_2$5), 24.1 ($CH_2$7), 26.2 ($CH_2$8), 29.4 ($CH_2$12), 30.3 ($CH_2$4), 52.0 ($CH_3$1), 71.7 (CH14), 79.5 (C13), 173.7 (C), 173.77 (C). ^{31}P NMR (160 MHz, $CDCl_3$) δ 47.6, -25.5. IR (thin film) Vmax/cm^{-1} 3284, 1735, 1655. HRMS (ESI+) m/z calculated for $[C_{14}H_{23}O_5NP]^+$ 316.1308, observed 316.1303.

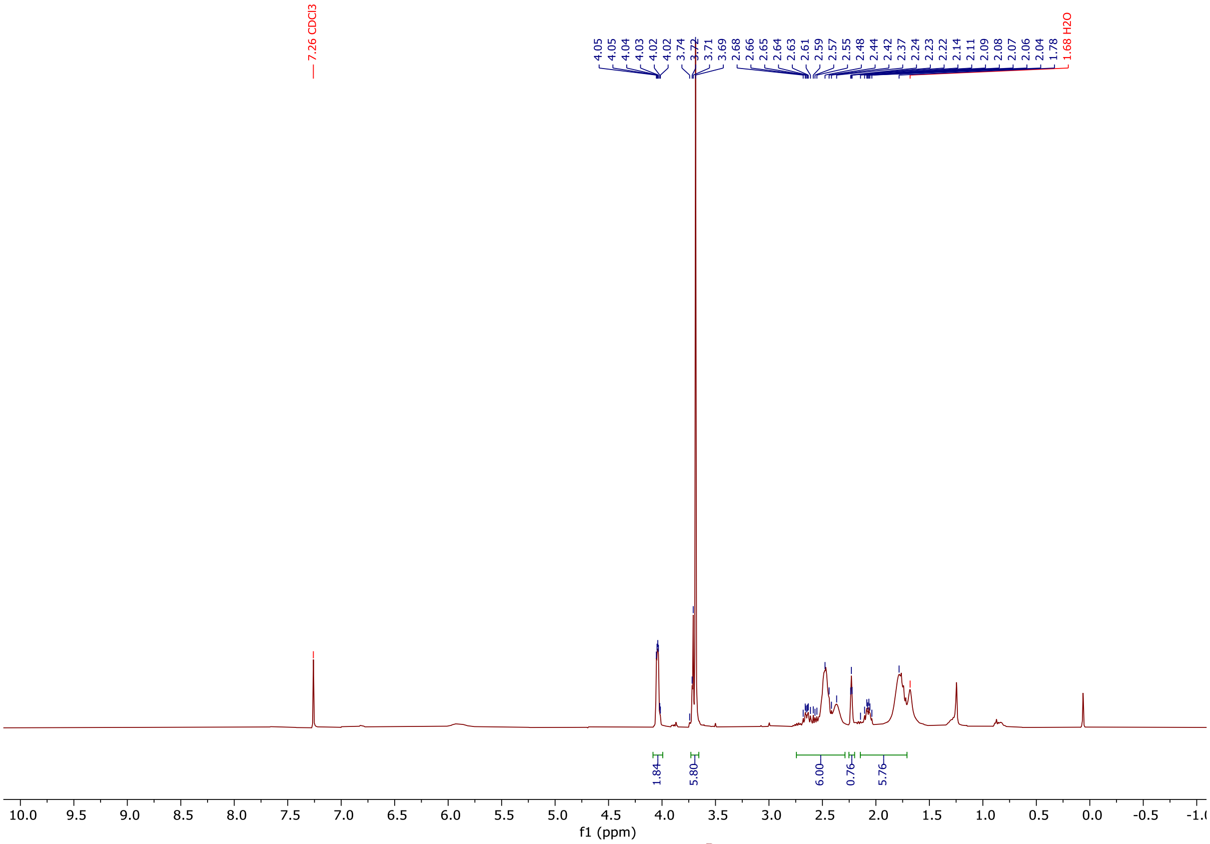

7.26 CDCl3
4.05
4.05
4.04
4.03
4.02
4.02
3.74
3.72
3.71
3.69
2.68
2.66
2.65
2.64
2.63
2.61
2.59
2.57
2.55
2.48
2.44
2.42
2.37
2.24
2.23
2.22
2.14
2.11
2.09
2.08
2.07
2.06
2.04
1.78
1.68 H2O
1.84
5.80
6.00
0.76
5.76
f1 (ppm)

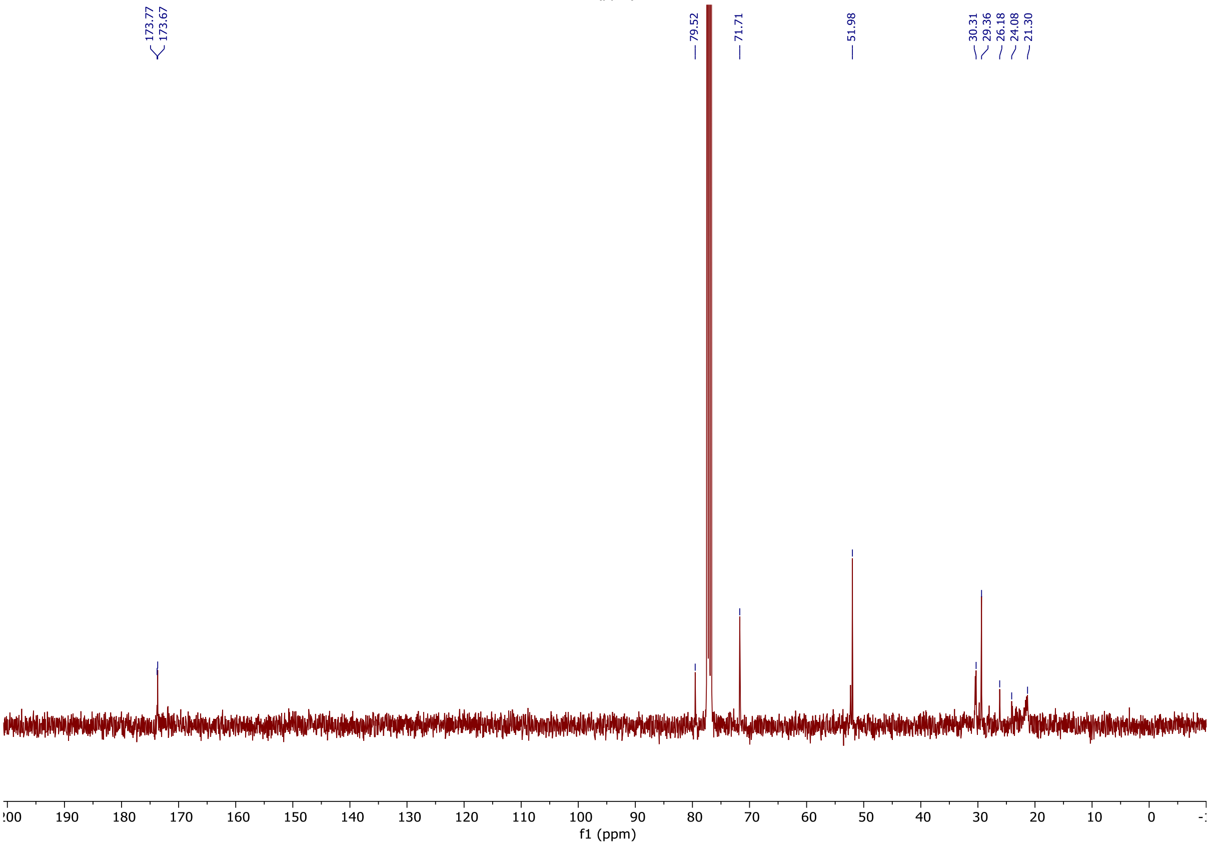

173.77
173.67
79.52
71.71
51.98
30.31
29.36
26.18
24.08
21.30
f1 (ppm)

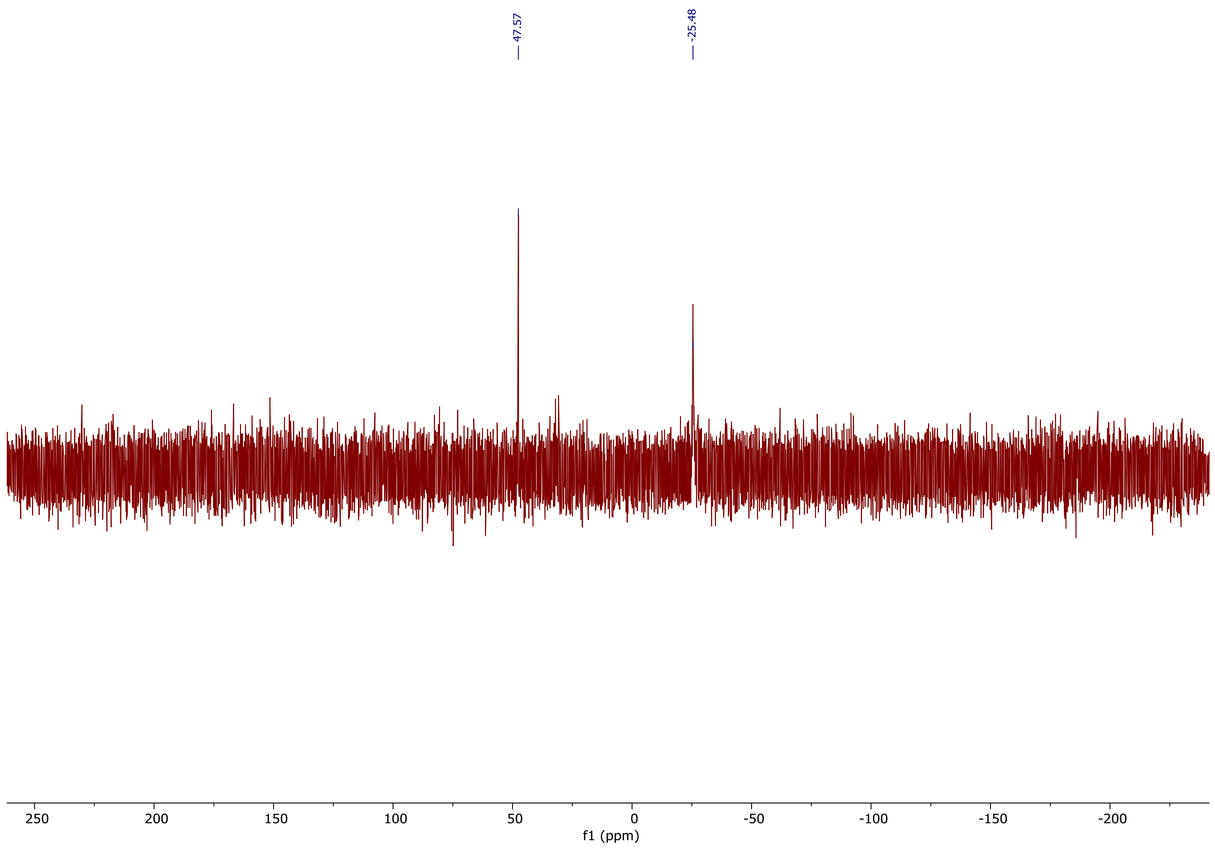
47.57
-25.48
250
200
150
100
50
0
-50
-100
-150
-200
f1 (ppm)

Synthesis of 2-chloro-1,3-dimethyl-3,4,5,6-tetrahydropyrimidin-1-ium hexafluorophosphate[2] CDMP 8

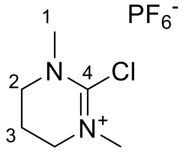

To a stirred solution of 1,3-dimethyltetrahydropyrimidin-2(1*H*)-one (256 mg, 242 µL, 2.0 mmol) in anhydrous DCM (2.8 ml) was added dry DMF (30 µL) and oxalyl chloride (2 M in DCM, 1.2 mL, 2.4 mmol). Reaction mixture was stirred under argon at 50 °C overnight. Solvent was co-evaporated with DCM (2×5 mL). The crude was taken up in dry DCM (3 mL), added KPF_6 (442 mg, 2.4 mmol), and the resulting suspension was stirred at room temperature under argon overnight. The reaction suspension was filtered through a pad of celite and the filtrate was concentrated under reduced pressure. The resultant crude was precipitated from diethyl ether over DCM. The precipitate was collected via filtration, and washed sequentially with diethyl ether (5 mL), $CHCl_3$ (1 mL), and diethyl ether (5 mL) to yield the product as a white solid (160 mg, 0.55 mmol, 27%).

^{1}H NMR (400 MHz, $CDCl_3$) δ 2.26 (p, *J* = 6.1 Hz, 2H, $CH_2$3), 3.43 (s, 6H, $CH_3$1), 3.75 (t, *J* = 6.0 Hz, 4H, $CH_2$2). ^{13}C NMR (100 MHz, $CDCl_3$) δ 19.2 (CH_2), 43.2 (CH_2), 50.5 (CH_3), 152.0 (C4). IR (solid) Vmax/cm^{-1} 1727, 1649. HRMS (ESI+) m/z calculated for $[C_6H_{12}N_2{}^{35}Cl]^+$ 147.0683, observed 147.0681.

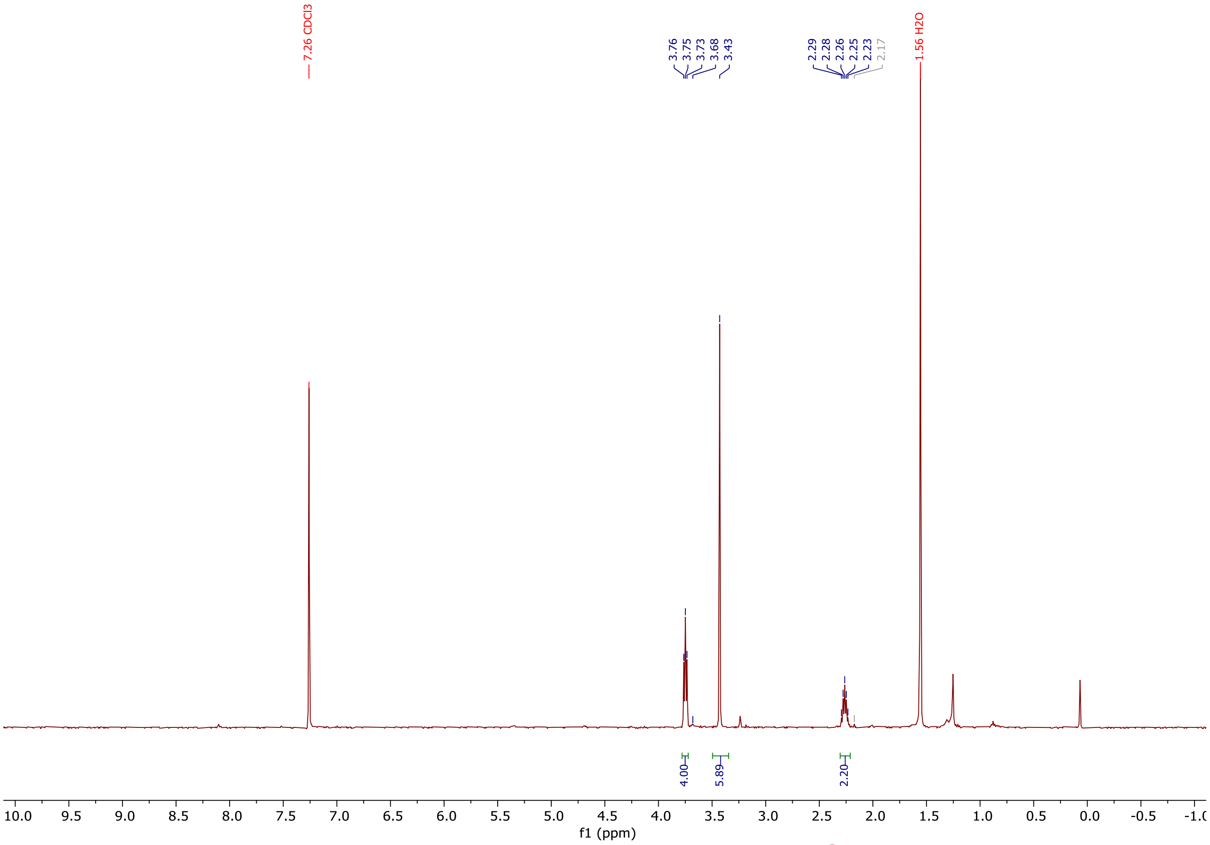

7.26 CDCl3
3.76
3.75
3.73
3.68
3.43
2.29
2.28
2.26
2.25
2.23
2.17
1.56 H2O
4.00
5.89
2.20
f1 (ppm)

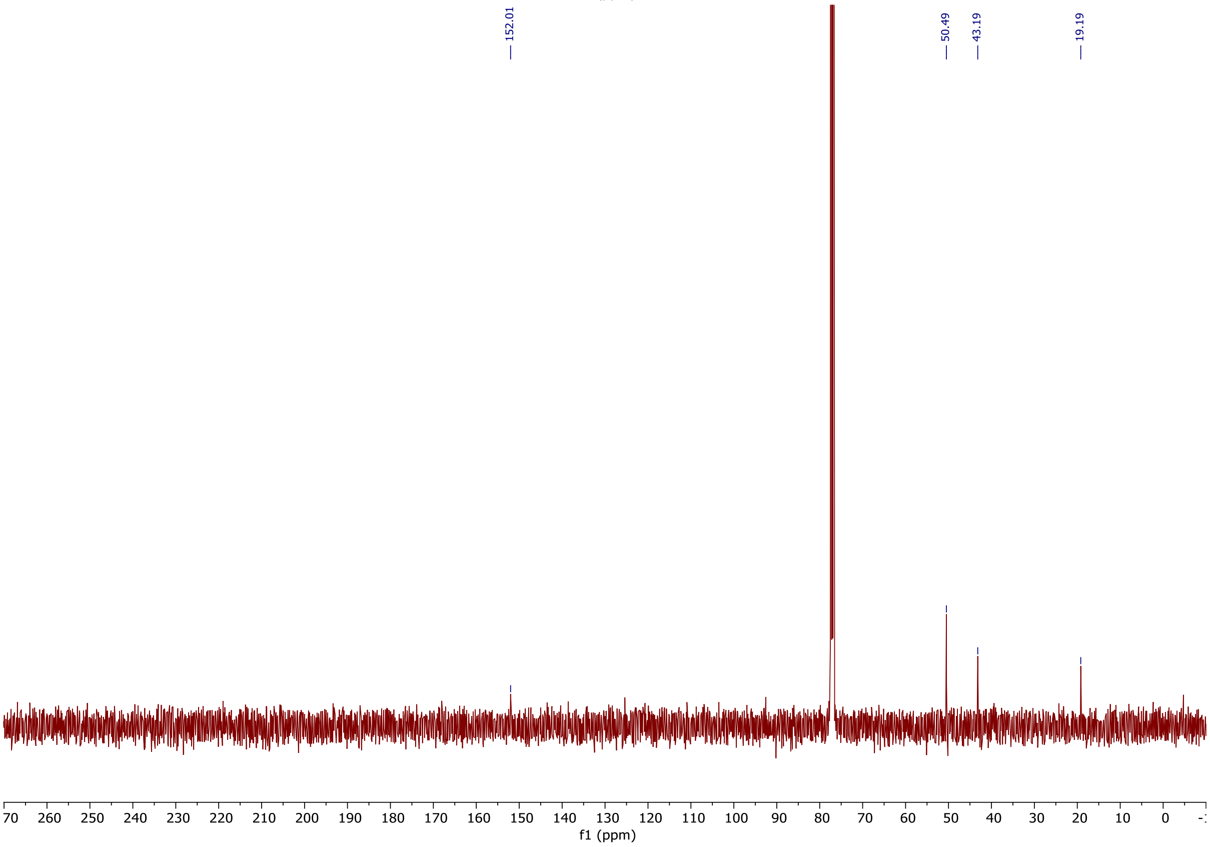

152.01
50.49
43.19
19.19
f1 (ppm)

Bioconjugation general experimental

This section is in addition to general experimental. All conjugation experiments were performed in standard polypropylene Eppendorf safe-lock tubes (0.6, 1.5, or 2.0 mL) at atmospheric pressure with mixing (300 rpm) at the temperature stated. Protein purification, buffer swapping, and desalting was carried out *via* ultrafiltration using Vivaspin 500 centrifugal concentrators (Sartorius) with molecular weight cut-off (MWCO) of 10 kDa or size exclusion filtration using Zebaspin (ThermoFisher Scientific) with MWCO of 7 kDa. Preconditioning of Vivaspin 500 centrifugal concentrators (Sartorius) and Zebaspin (ThermoFisher Scientific) were carried out according to manufacturer instructions. Centrifugation was performed using Eppendorf 5415R fixed angle bench rotor. Trastuzumab (Ontruzant^{TM}) was purchased from UCLH in its clinical formulation (Samsung Bioepis, lyophilised). Trastuzumab Fab was prepared by a sequential enzymatic digest of the full antibody with pepsin and papain, following a literature procedure.[1] UV-Vis spectroscopy was used to determine protein concentration using NanoDrop One/One Microvolume UV-Vis Spectrophotometer (Thermo Fisher) operating at room temperature. Sample buffer was used as blank for baseline correction with extinction coefficients: ε_{280} = 68590 M^{-1} cm^{-1} for Trastuzumab Fab, ε_{280} = 215380 M^{-1} cm^{-1} for trastuzumab. Antibody conjugate concentration was determined using the same extinction coefficient as for native trastuzumab Fab.

SDS-PAGE

Fab bioconjugation reactions were monitored by 15% glycine-SDS-PAGE with 6% stacking gel under nonreducing conditions. Full antibody bioconjugation reactions were monitored by 4–20% Mini-PROTEAN® TGX™ Precast Protein Gels 15 wells (Bio-rad Laboratories) under nonreducing conditions. Protein samples (3 µg) were mixed 1:1 with SDS non-reducing loading dye and heated at 85 °C for 5 min before loaded to the gel. Page Ruler Plus Pre-Stained Protein Ladder (Thermo Scientific) was used. Samples were run at 200 V, 50 min at 1× running buffer. Gels were stained in Coomassie Blue Stain (1 h) and subsequently de-stained with water (18-24 h).

Preparation of 5× SDS PAGE loading dye

Glycerol (10 g, 8 mL), dH_2O (4 mL), of 10 % (w/v) SDS (1.6 mL), of 0.5 M Tris-HCl pH 6.8 (1 mL), and Coomassie Brilliant Blue R-250 (0.025 g).

Preparation of 10× running buffer

Tris base (30 g), glycine (144 g), SDS (10 g), and dH_2O (1 L). Final pH adjusted to 8.3.

Preparation of Coomassie blue stain

10% Ammonium Sulfate (100 g), 0.1% Coomassie Brilliant Blue R-250 (500 mg), 3% Phosphoric Acid (30 mL), ethanol (200 mL), and dH_2O (1 L)

LCMS analysis for Fab and full antibodies

Molecular masses of native and modified proteins were measured using liquid chromatography mass spectrometry (LCMS) on an Agilent 1100/1200 LC system, with an Agilent 6510 QTOF or Agilent 6530 QTOF mass spectrometer. All protein samples were desalted using Zebaspin (7kDa MWCO, ThermoFisher Scientific) prior to LCMS analysis. For Trastuzumab, the antibody (30 µL, 5 µM) was deglycosylated with 0.5 µL PNGase (New England Biolabs) for 16 h at 4 °C prior to LCMS submission. Protein sample (2 µL, 2-5 µM) was separated on Agilent PLRP-S, 1000 Å, 8 µM, 50 mm x 2.1 mm column; with a mobile phase consisting of 0.1% formic acid in water (solvent A) and 0.1% formic acid in acetonitrile (solvent B). For Agilent 6510 QTOF: Gradient flow rate = 0.3 mL min^{-1} [time/min](solvent A:solvent B): [0-2](85:15). [2-3](85:15-68:32).[3-4](68:32). [4-14](68:32-65:35). [14-18](65:35-5:95). [18-20](5:95). [20-22](5:95-85:15). [22-25](-85:15). For Agilent 6530 QTOF: Gradient flow rate = 0.8 mL

min^{-1} [time/min](solvent A:solvent B): [0-1](80:20). [1-6.5](80:20-40:60).[6.5-7.5](40:60). [7.5-7.6](40:60-80:20). [7.6-8.5](80:20). The column temperature was maintained at 60 °C. Agilent 6510/6530 QTOF mass spectrometer was operated in a positive polarity mode, coupled with an ESI ion source. The ion source parameters were set up with a VCap of 3500 V, a gas temperature at 350 °C, a dry gas flow rate at 10 L min^{-1} and a nebulizer of 30 psig. MS TOF was acquired under conditions of a fragmentor at 380 V, a skimmer at 65 V and an acquisition rate at 0.5 spectra s^{-1} in a profile mode, within a scan range between 700 and 7000 m/z. The data was then analysed by deconvoluting a spectrum to a zero charge mass spectra using a maximum entropy deconvolution algorithm within the MassHunter software version B.07.00.

Assessing reagents on Fab (experimental and mass spec data)

Native Trastuzumab Fab S9

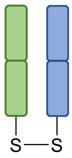

Fab **S9**

Trastuzumab Fab **S9** was obtained through pepsin/papain digestion of trastuzumab as described previously.[1] Fab (5 µM, 130 µL) in pH 8.0 buffer (25 mM BB, 25 mM NaCl, 2 mM EDTA) was desalted (ZebaSpin, 7 kDa MWCO) prior to LCMS analysis.

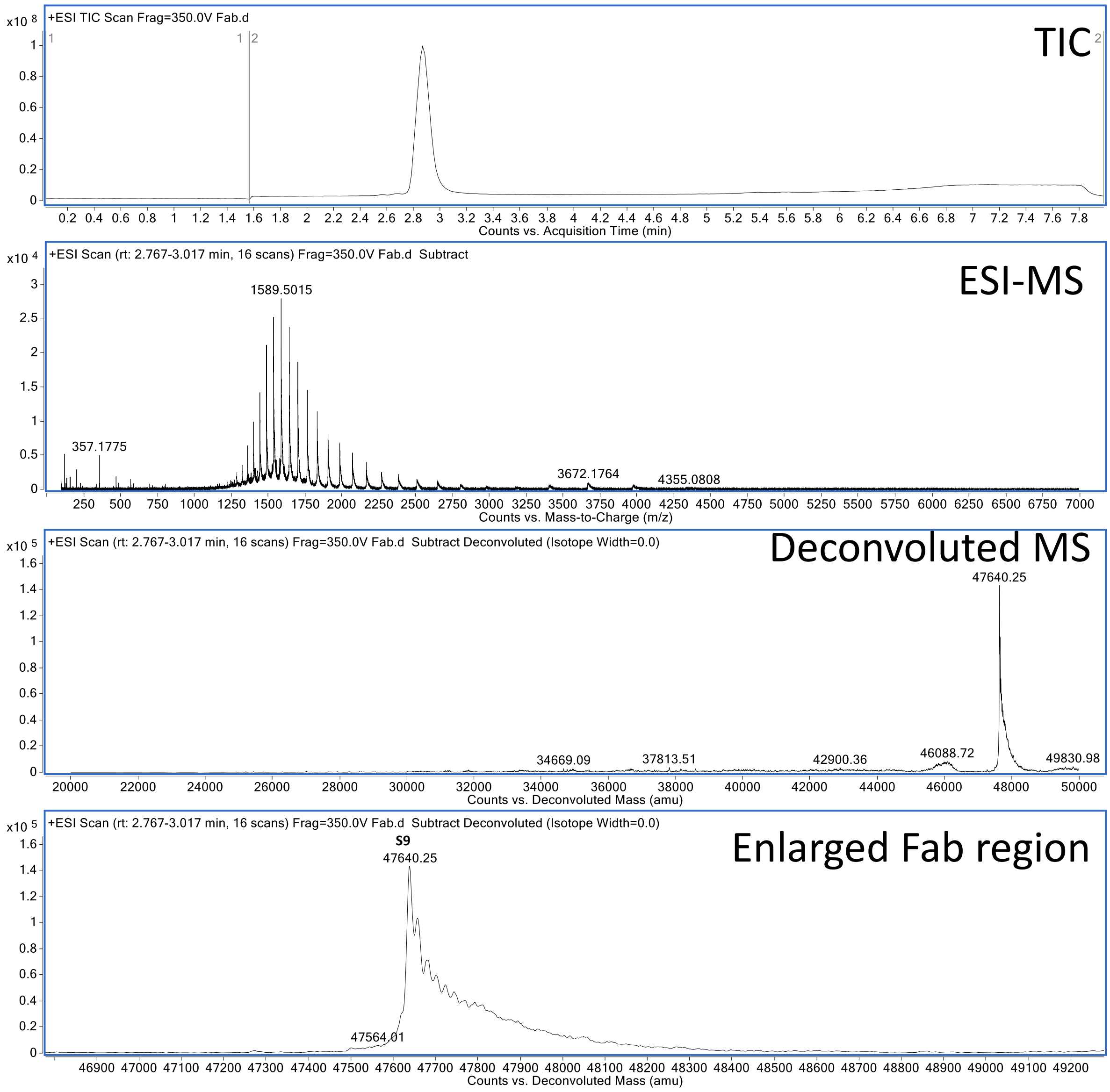

Figure S 7 LCMS of Fab **S9**. Observed mass: 47640 Da.

Reduced Fab

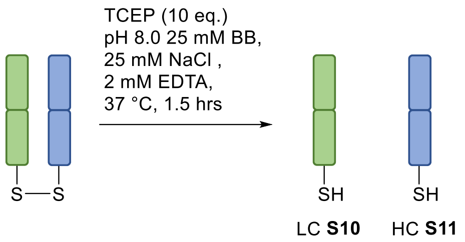

Fab (30 µL, 100 µM, 4.76 mg/mL) in BBS general buffer was reduced with TCEP (2 µL, 20 mM in H_2O, 10 eq.) The mixture was incubated at 37 °C for 1.5 h. Sample was desalted (7 kDa MWCO, ZebaSpin) prior to LCMS analysis.

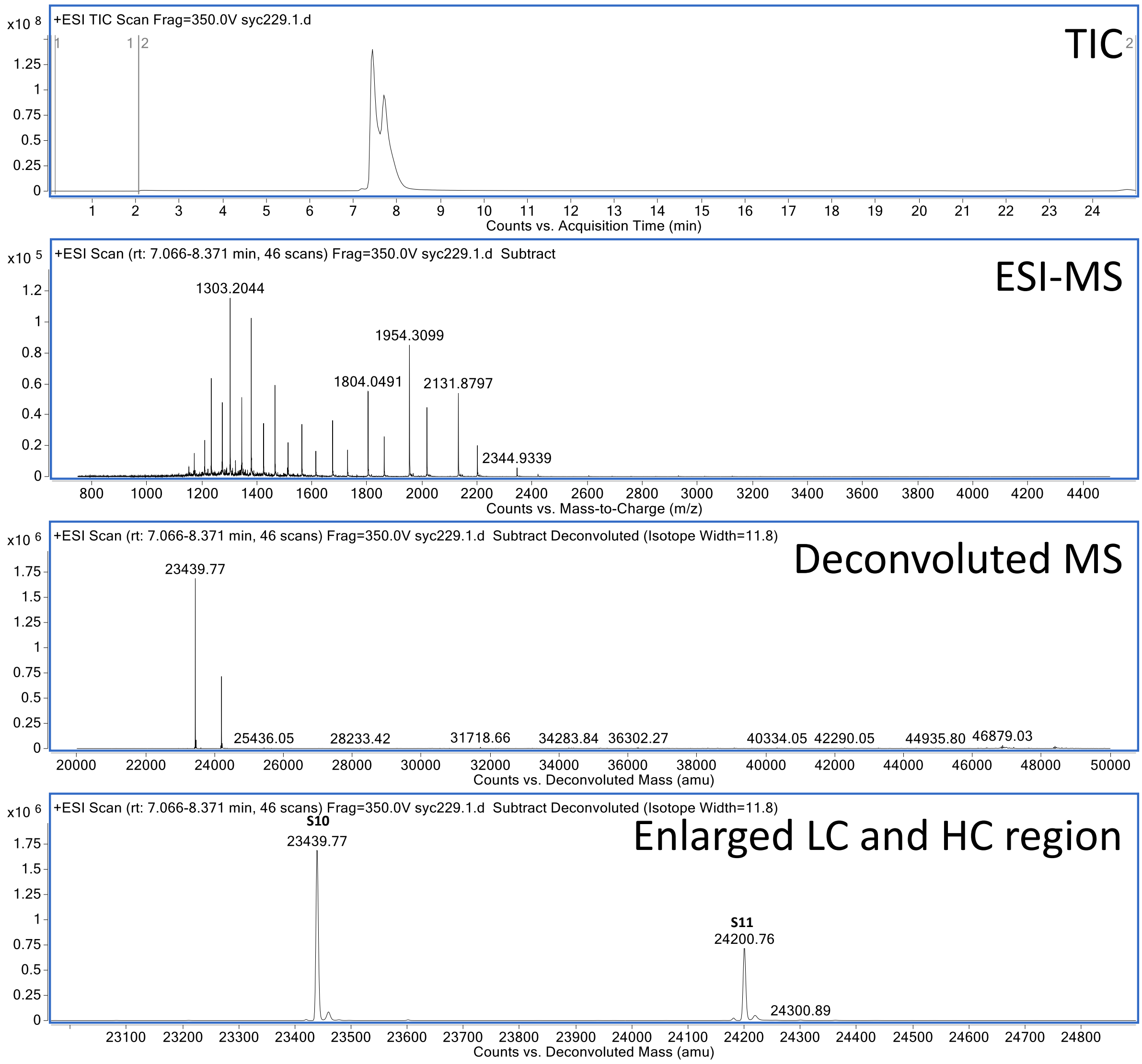

Figure S 8 LCMS of Reduced Fab; Reduced Fab LC **S10**, observed mass 23440 Da; Reduced Fab HC **S11** 24200 Da.

Fab reactions

Reaction of reduced Fab with DBHA 1

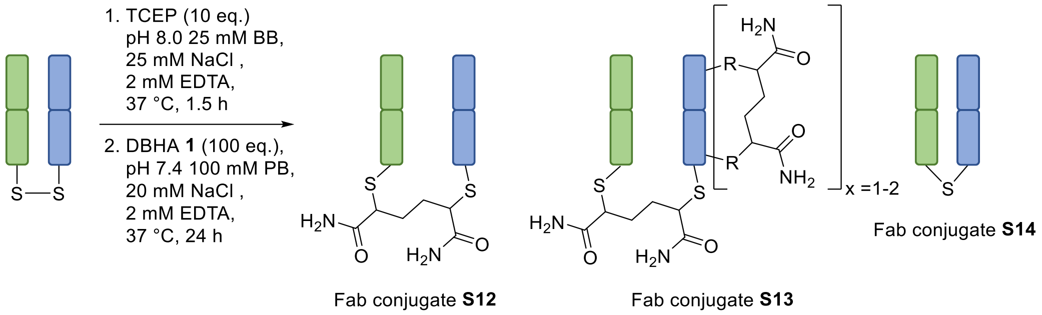

Fab **S9** (25 µL, 100 µM) in buffer (pH 8.0, 25 mM BB, 25 mM NaCl, 2 mM EDTA) was reduced with TCEP (1 µL, 20 mM in H_2O, 10 eq.) The mixture was incubated at 37 °C for 1.5 h. Reaction mixture was diluted and purified (ZebaSpin) into PBS (pH 7.4, 100 mM, 20 mM NaCl, 2 mM EDTA). To the resulting reduced Fab (100 µL, 20 µM) was added DBHA **1** (2 µL, 100 mM in MeCN, 100 eq.) and the reaction was incubated at 37 °C for 24 h. Sample was desalted (ZebaSpin) and analysed using LCMS.

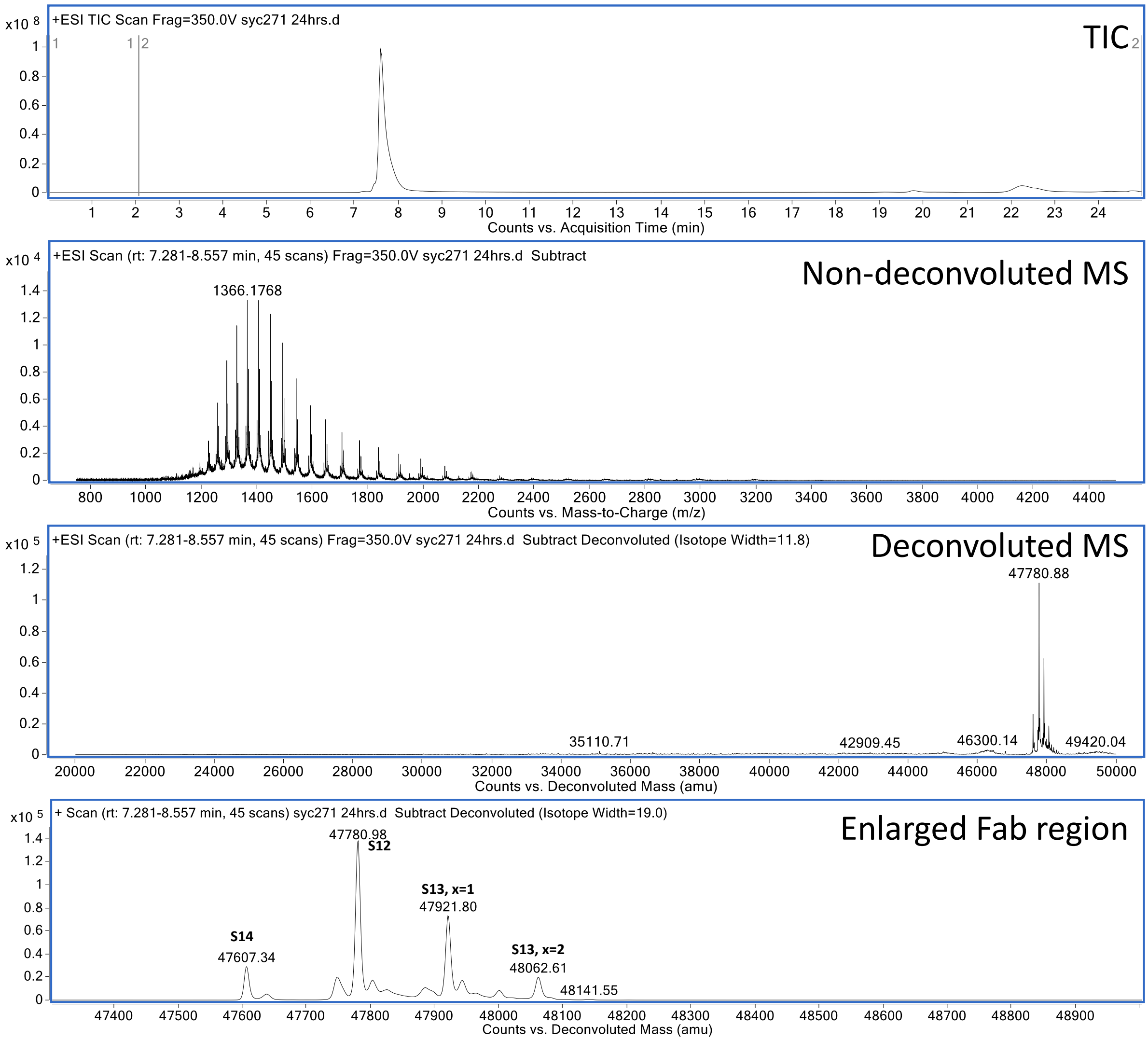

Figure S 9 LCMS of reaction of reduced Fab with DBHA **1**. **S12** expected mass 47780 Da, observed mass 47780 Da; **S13** expected mass: 47920 Da (x =1), 48060 Da (x =2), observed mass: 47921 Da, 48062 Da; **S14** expected mass 47606 Da, observed mass 47607.

Reaction of reduced Fab with NTCB 2

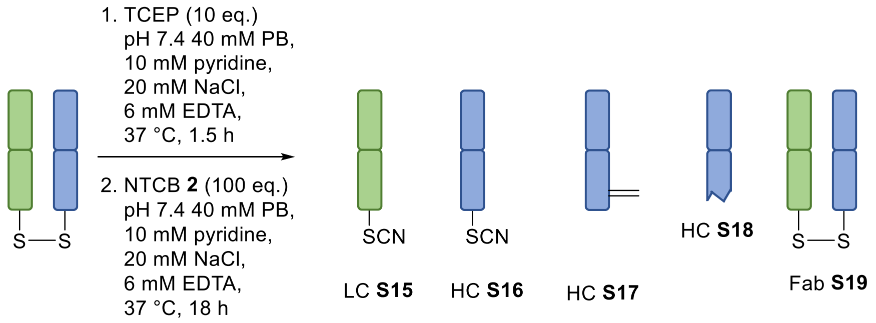

To Fab **S9** (50 µL, 20 µM) in buffer (pH 7.4, 40 mM PB, 20 mM NaCl, 6 mM EDTA, 10 mM pyridine) was added TCEP (0.5 µL, 20 mM in H_2O, 10 eq.) and the reaction mixture was incubated at 37 °C for 1.5 h. To the reaction mixture was added NTCB **2** (1 µL, 100 mM in MeCN, 100 eq.) and the reaction was incubated at 37 °C for 18 h. Sample was desalted (ZebaSpin) and analysed using LCMS.

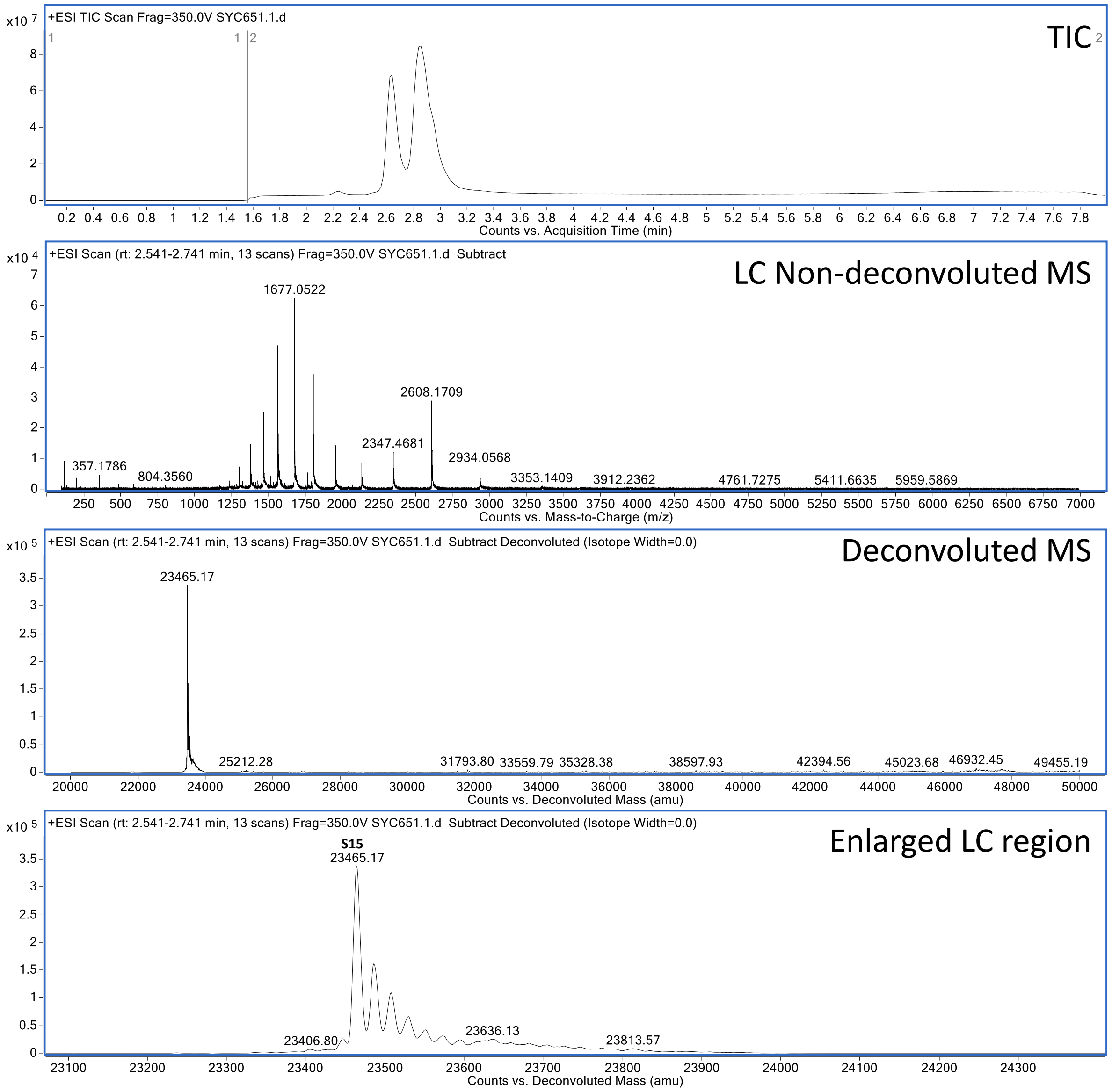

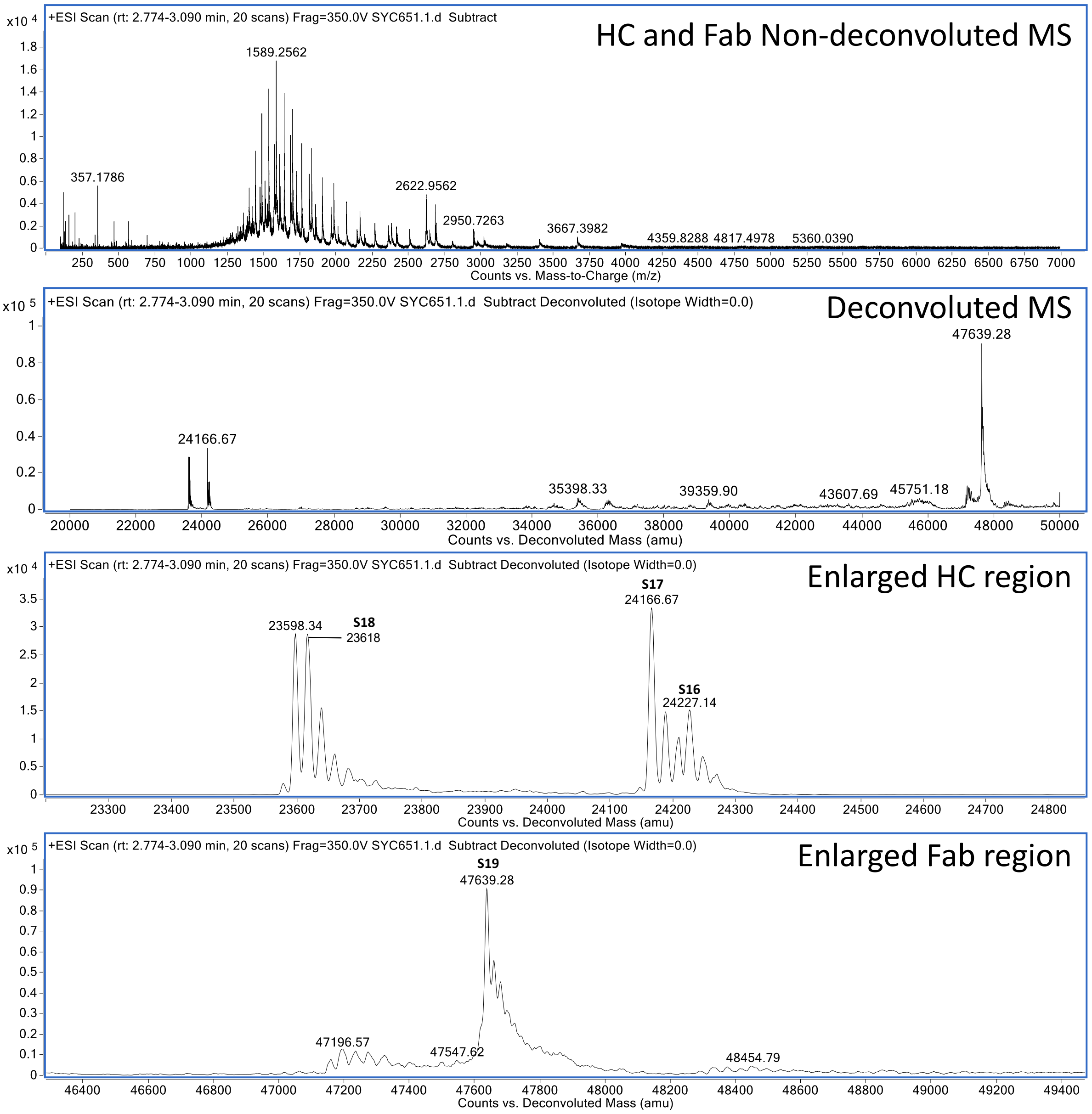

Figure S 10 LCMS of reaction of reduced Fab with NTCB **2**. **S15** expected mass: 23465 Da, observed mass: 23465 Da. **S16** expected mass: 24225 Da, observed mass: 24227 Da; **S17** expected mass: 24166 Da, observed mass: 24167 Da; **S18** expected mass: 23599 Da (intramolecular fragmentation), 23616 Da (intermolecular fragmentation), observed mass: 23598 Da, 23618 Da; **S19** expected mass: 47640 Da, observed mass: 47639 Da.

Reaction of reduced Fab with MKYM 3

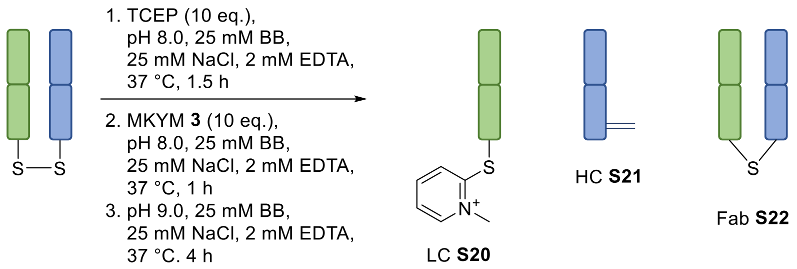

To Fab (30 µL, 120 µM) in pH 8.0 buffer (25 mM BB, 25 mM NaCl, 2 mM EDTA) was added TCEP (1.8 µL, 20 mM in H_2O, 10 eq.) and was incubated at 37 °C for 1.5 h. Reaction mixture was diluted and purified (ZebaSpin) into pH 8.0 buffer (25 mM BB, 25 mM NaCl, 2 mM EDTA). To the resulting reduced Fab (130 µL, 20 µM) was added MKYM **3** (20 mM in MeCN, 10 eq.) and incubated at 37 °C for 1 h. Sample was purified (VivaSpin) into pH 9.0 (25 mM BB, 25 mM NaCl, 2 mM EDTA) and the reaction was incubated at 37 °C for 4 h. Sample was desalted (ZebaSpin) and analysed using LCMS.

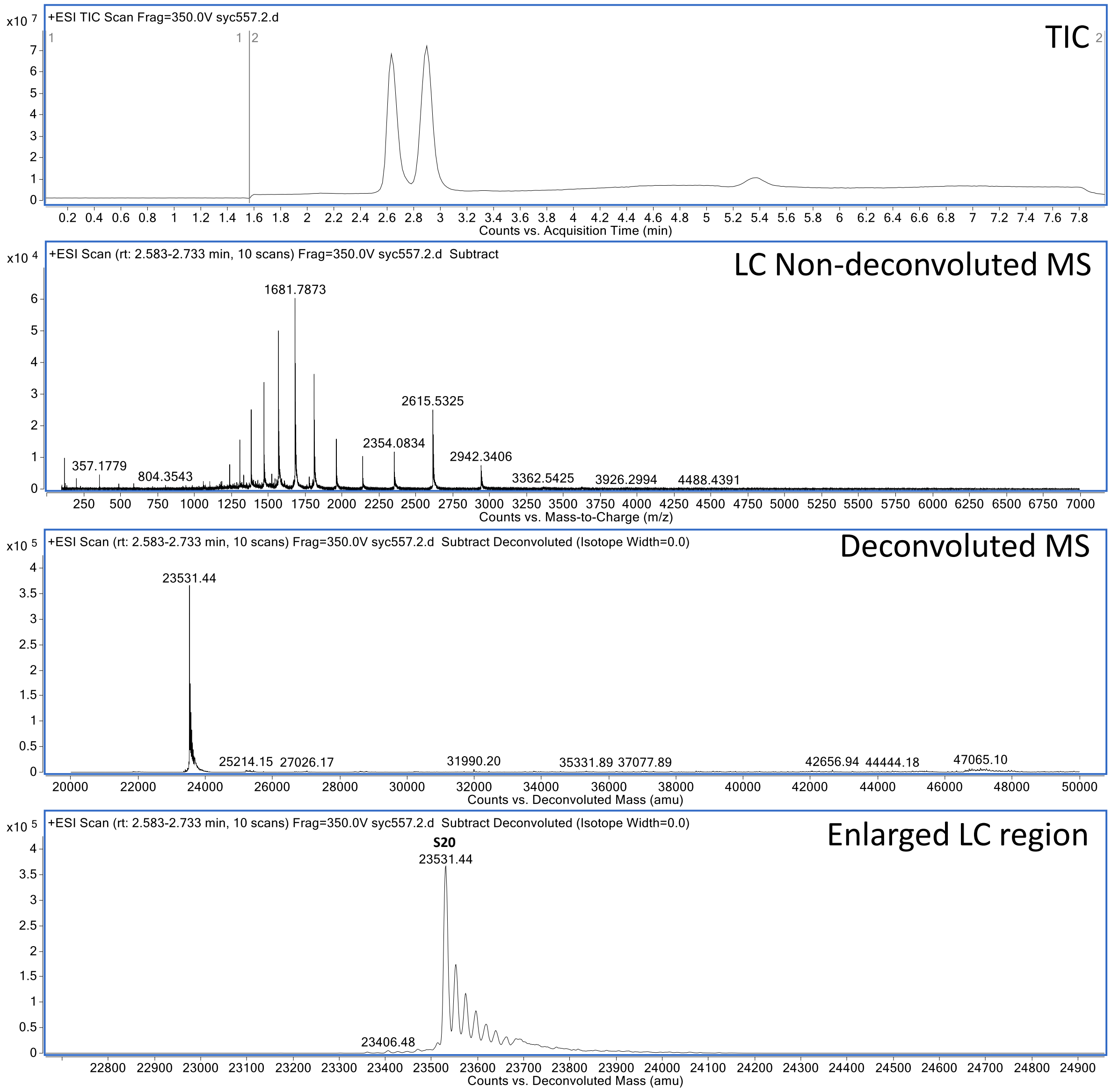

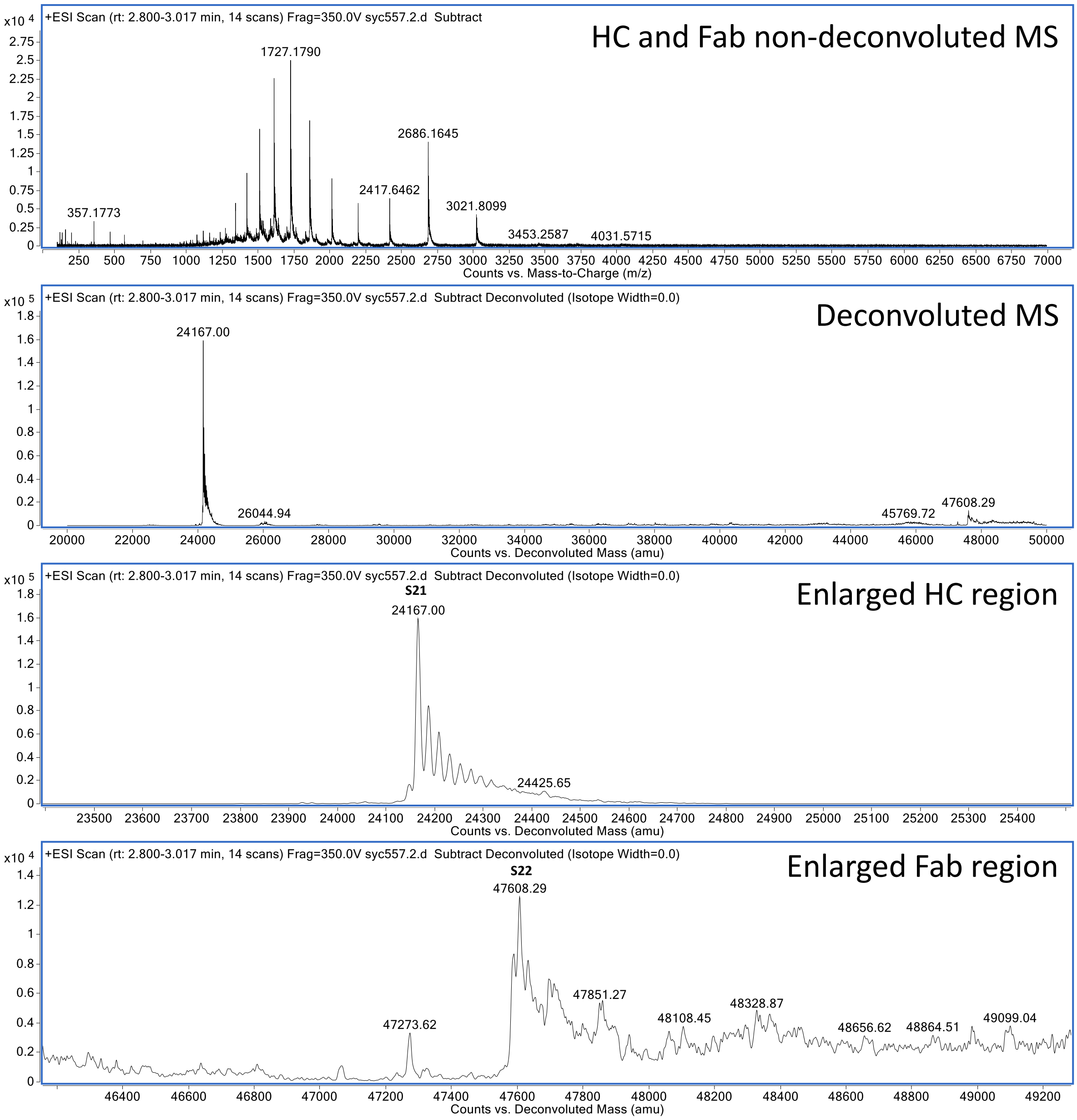

Figure S 11 LCMS of reaction of reduced Fab with MKYM **3**. **S20** expected mass: 23532 Da, observed mass: 23531 Da.**S21** expected mass: 24166 Da, observed mass: 24167 Da; **S22** expected mass: 47606 Da, observed mass: 47608 Da.

Control reaction of reduced Fab with MKYM 3 and then *N*-ethylmaleimide

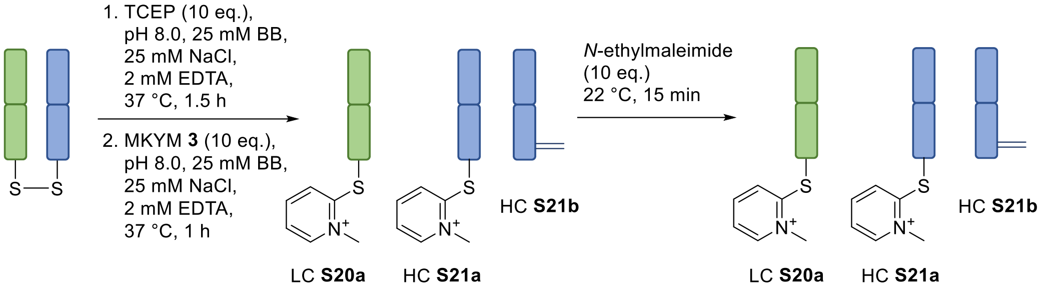

To Fab (30 µL, 120 µM) in pH 8.0 buffer (25 mM BB, 25 mM NaCl, 2 mM EDTA) was added TCEP (1.8 µL, 20 mM in H_2O, 10 eq.) and was incubated at 37 °C for 1.5 h. Reaction mixture was diluted and purified (ZebaSpin) into pH 8.0 buffer (25 mM BB, 25 mM NaCl, 2 mM EDTA). To the resulting reduced Fab (130 µL, 20 µM) was added MKYM **3** (20 mM in MeCN, 10 eq.) and incubated at 37 °C for 1 h. At this stage, an aliquot of the reaction was desalted (ZebaSpin) and analysed using LCMS. After which, *N*-methylmaleimide (5 mM in MeCN, 10 eq.) was added to the reaction mixture and incubated at 22 °C for 15 min. Sample was desalted (ZebaSpin) and analysed using LCMS.

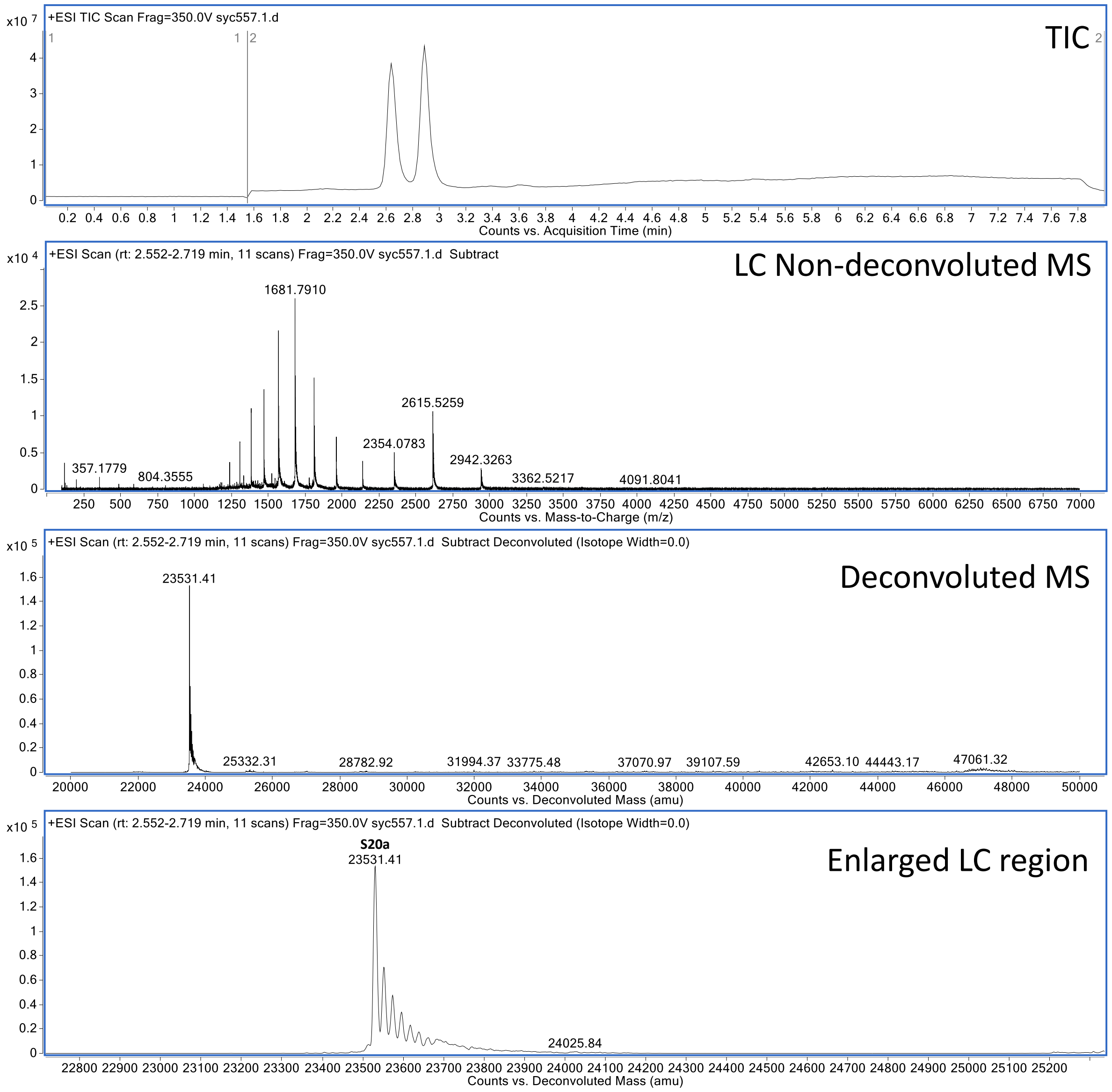
+ESI TIC Scan Frag=350.0V syc557.1.d
TIC
Counts vs. Acquisition Time (min)
+ESI Scan (rt: 2.552-2.719 min, 11 scans) Frag=350.0V syc557.1.d Subtract
LC Non-deconvoluted MS
1681.7910
2615.5259
2354.0783
2942.3263
357.1779
804.3555
3362.5217
4091.8041
Counts vs. Mass-to-Charge (m/z)
+ESI Scan (rt: 2.552-2.719 min, 11 scans) Frag=350.0V syc557.1.d Subtract Deconvoluted (Isotope Width=0.0)
Deconvoluted MS
23531.41
25332.31
28782.92
31994.37
33775.48
37070.97
39107.59
42653.10
44443.17
47061.32
Counts vs. Deconvoluted Mass (amu)
+ESI Scan (rt: 2.552-2.719 min, 11 scans) Frag=350.0V syc557.1.d Subtract Deconvoluted (Isotope Width=0.0)
Enlarged LC region
S20a
23531.41
24025.84
Counts vs. Deconvoluted Mass (amu)

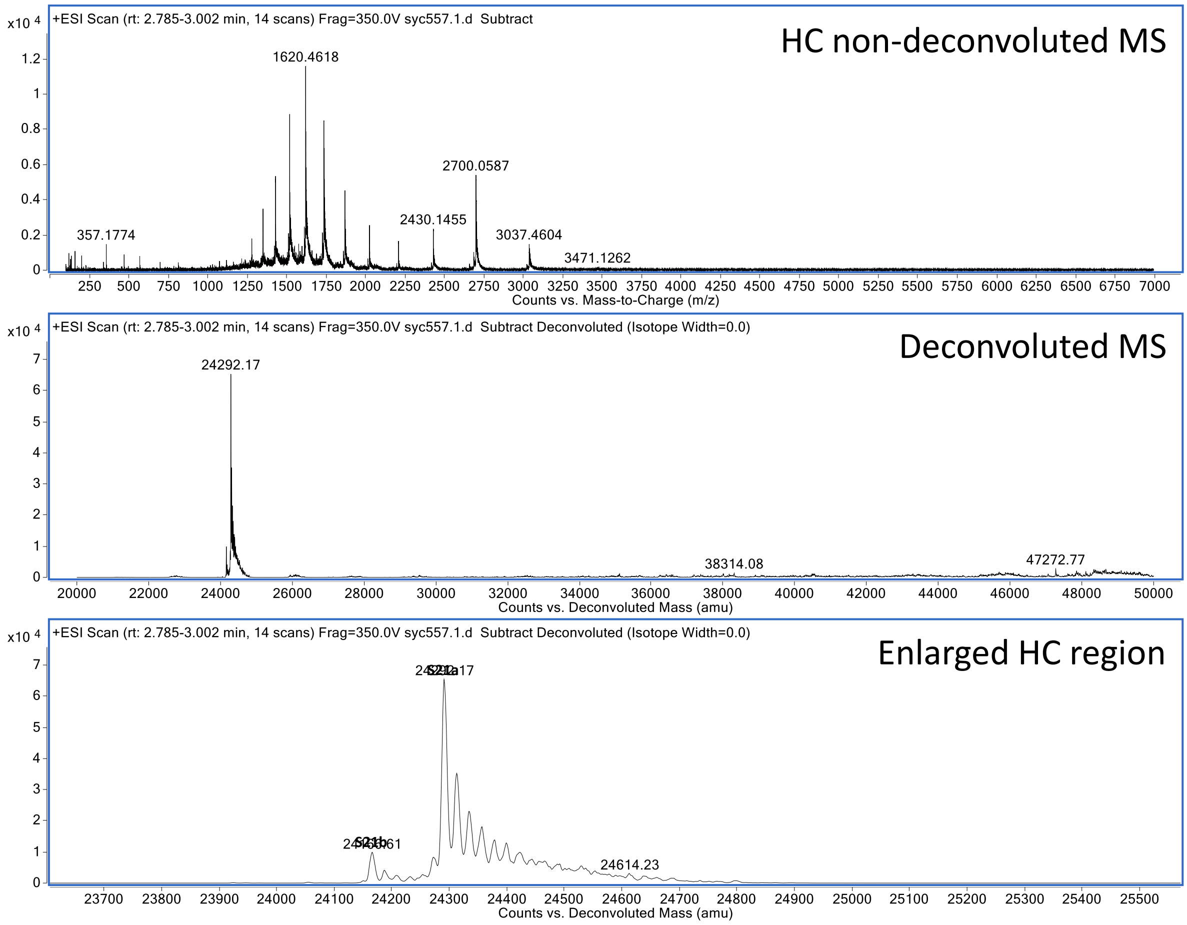

Figure S 12 LCMS of reaction of reduced Fab with MKYM **3** after 1 h incubation at pH 8.0. **S20a** expected mass: 23532 Da, observed mass: 23531 Da. **S21a** expected mass: 24292 Da, observed mass: 24292 Da; **S21b** expected mass: 24166 Da, observed mass: 24167 Da.

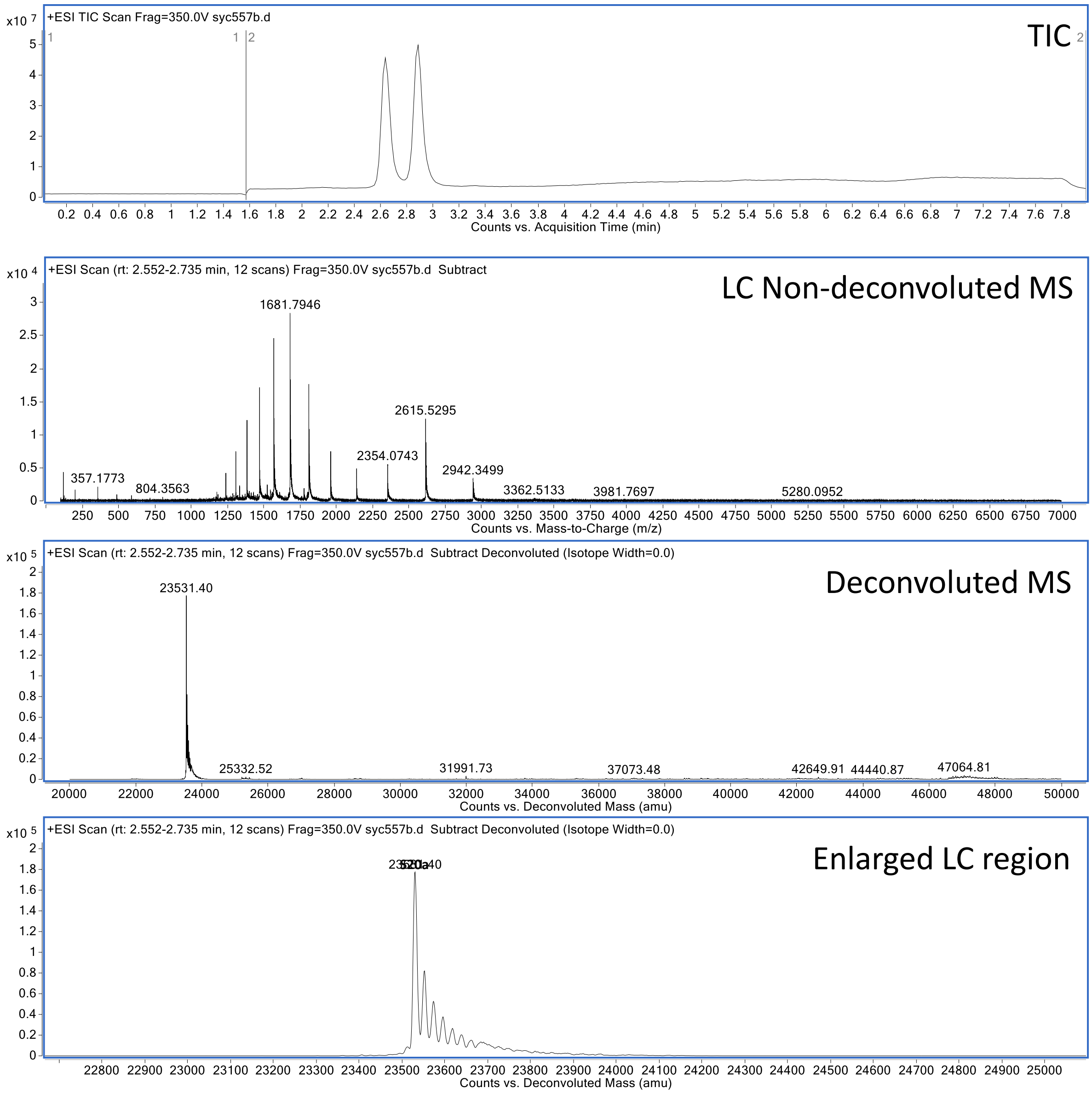

+ESI TIC Scan Frag=350.0V syc557b.d
TIC
Counts vs. Acquisition Time (min)
+ESI Scan (rt: 2.552-2.735 min, 12 scans) Frag=350.0V syc557b.d Subtract
LC Non-deconvoluted MS
1681.7946
2615.5295
2354.0743
2942.3499
357.1773
804.3563
3362.5133
3981.7697
5280.0952
Counts vs. Mass-to-Charge (m/z)
+ESI Scan (rt: 2.552-2.735 min, 12 scans) Frag=350.0V syc557b.d Subtract Deconvoluted (Isotope Width=0.0)
Deconvoluted MS
23531.40
25332.52
31991.73
37073.48
42649.91
44440.87
47064.81
Counts vs. Deconvoluted Mass (amu)
+ESI Scan (rt: 2.552-2.735 min, 12 scans) Frag=350.0V syc557b.d Subtract Deconvoluted (Isotope Width=0.0)
Enlarged LC region
Counts vs. Deconvoluted Mass (amu)

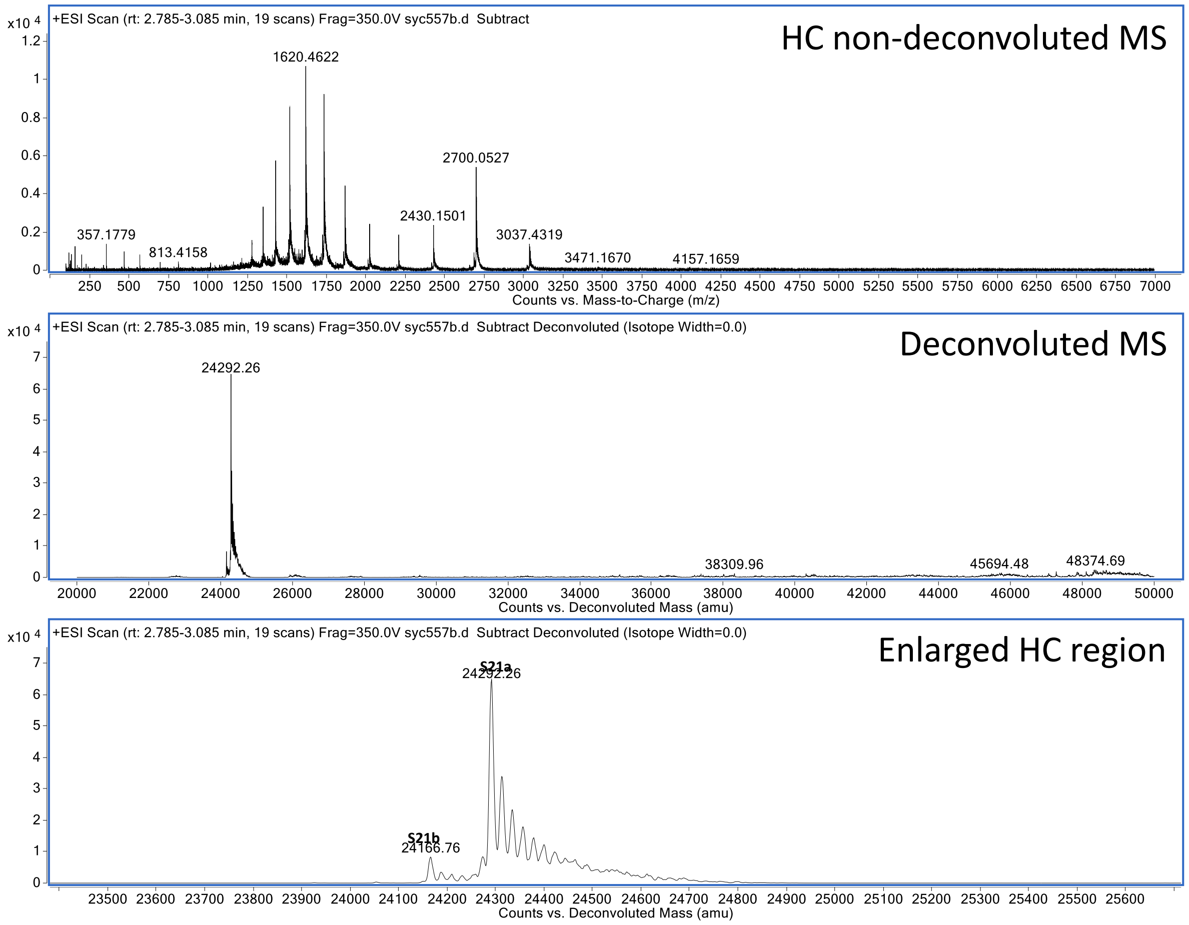

Figure S 13 LCMS of reaction of reduced Fab with MKYM **3** after 1 h incubation at pH 8.0, followed by *N*-ethylmaleimide addition. **S20a** expected mass: 23532 Da, observed mass: 23531 Da. **S21a** expected mass: 24292 Da, observed mass: 24292 Da; **S21b** expected mass: 24166 Da, observed mass: 24167 Da.

Reaction of reduced Fab with EDC 4

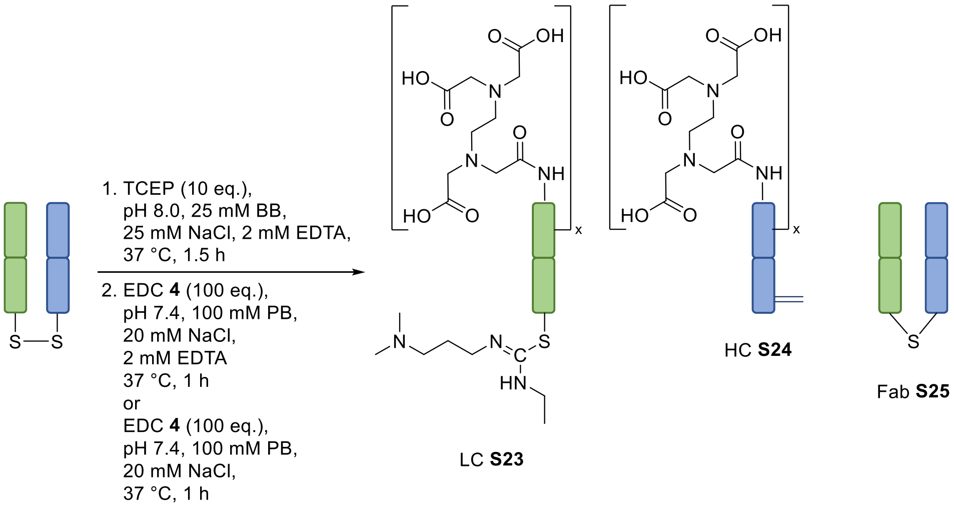

To Fab (25 µL, 100 µM) in pH 8.0 buffer (25 mM BB, 25 mM NaCl, 2 mM EDTA) was added TCEP (1 µL, 20 mM in H_2O, 10 eq.) and incubated at 37 °C for 1.5 h. Reaction mixture was diluted and purified (ZebaSpin) into pH 7.4 buffer (buffer with EDTA: 100 mM PB, 20 mM NaCl, 2 mM EDTA, buffer without EDTA: 100 mM PB, 20 mM NaCl). To the resulting reduced Fab (100 µL, 20 µM) was added EDC **4** (2 µL, 100 mM in H_2O, 100 eq.) and the reaction was incubated at 37 °C for 1 h. Sample was desalted (ZebaSpin) and analysed using LCMS.

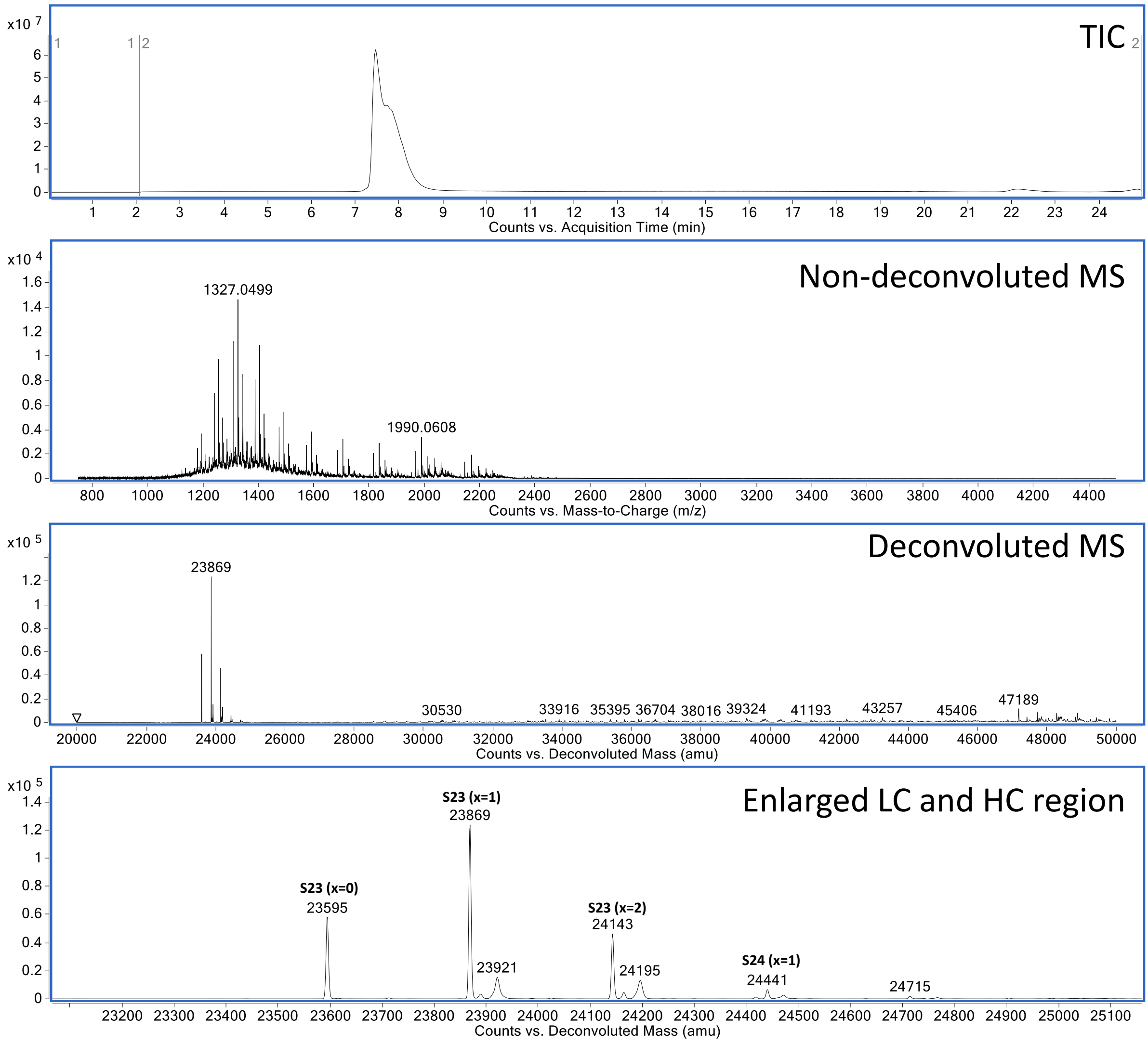

Figure S 14 LCMS of reaction of reduced Fab with EDC **4** (in buffer with EDTA). **S23** expected mass: 23596, 23870 Da (x=0,1), observed mass: 24595, 23869 Da (x=0,1); **S24** expected mass: 24440 Da (x=1), observed mass: 24440 Da.

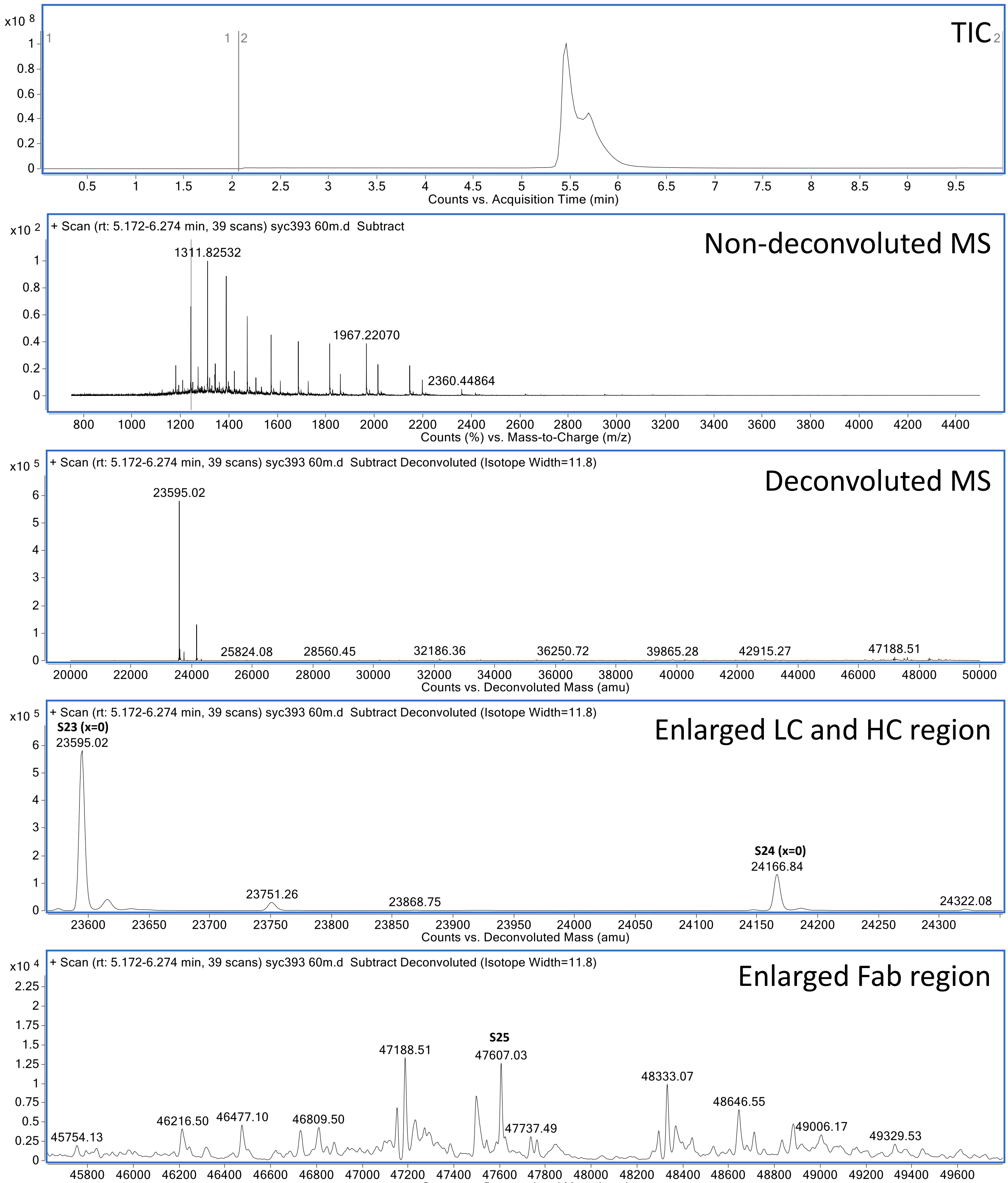

Figure S 15 LCMS of reaction of reduced Fab with EDC **4** (in buffer without EDTA). **S23** expected mass: 23596 Da, observed mass: 24595 Da; **S24** expected mass: 24166 Da, observed mass: 24167 Da; **S25** expected mass: 47606 Da, observed mass: 47607 Da.

Reaction of reduced Fab with SBTU 5

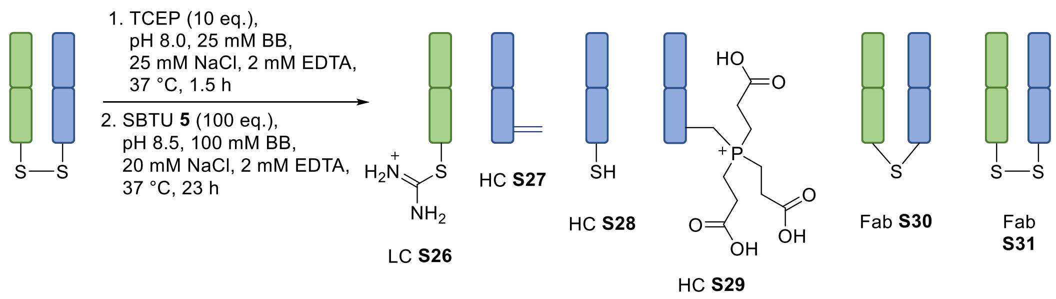

To Fab (25 µL, 100 µM) in pH 8.0 buffer (25 mM BB, 25 mM NaCl, 2 mM EDTA) was added TCEP (1.25 µL, 20 mM in H_2O, 10 eq.) and incubated at 37 °C for 1.5 h. Reaction mixture was diluted and purified (ZebaSpin) into pH 8.5 buffer (100 mM BB, 20 mM NaCl, 2 mM EDTA). To the resulting reduced Fab (100 µL, 20 µM) was added SBTU **5** (2 µL, 100 mM in H_2O, 100 eq.) and the reaction was incubated at 37 °C for 23 h. Sample was desalted (ZebaSpin) and analysed using LCMS.

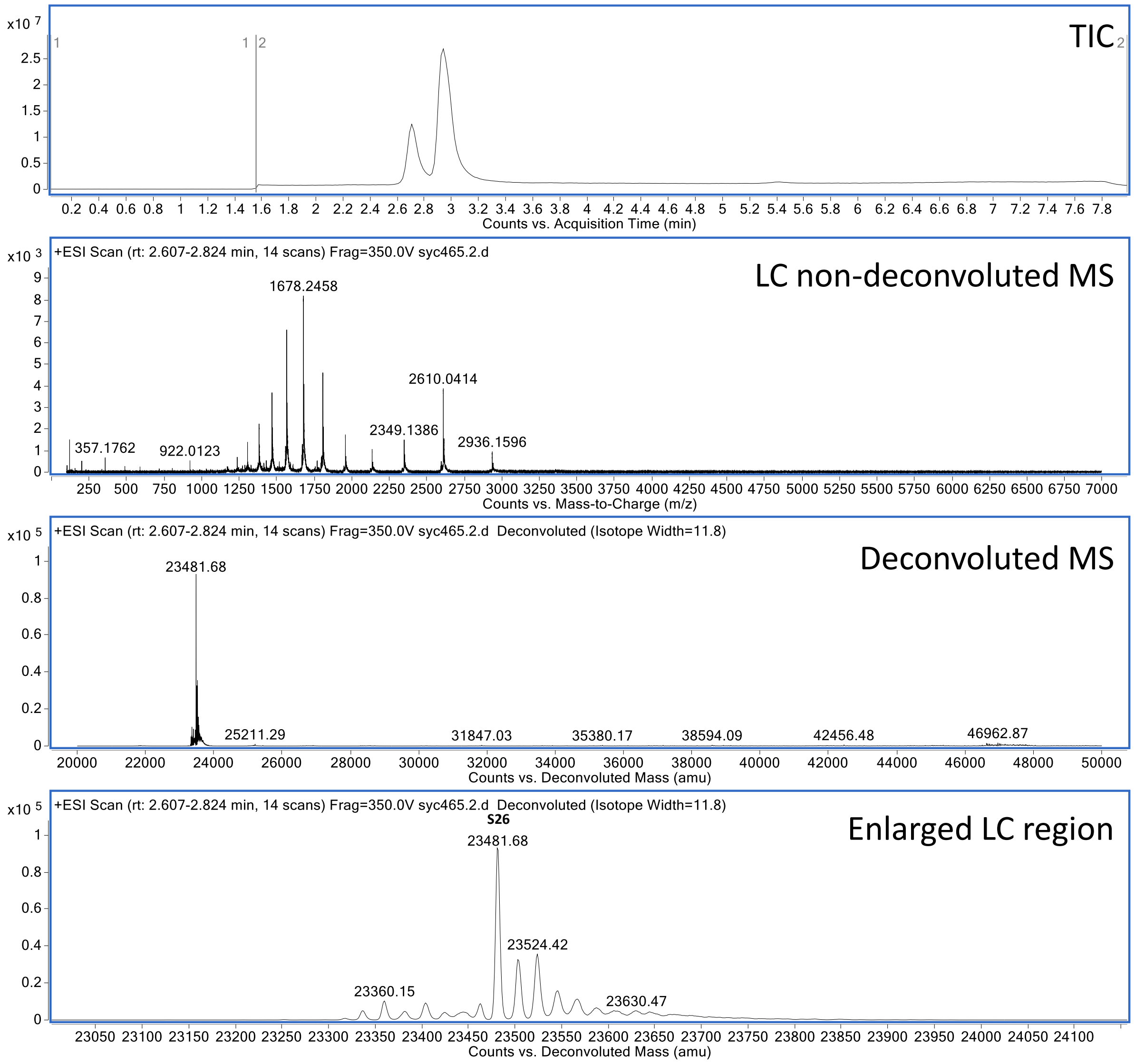

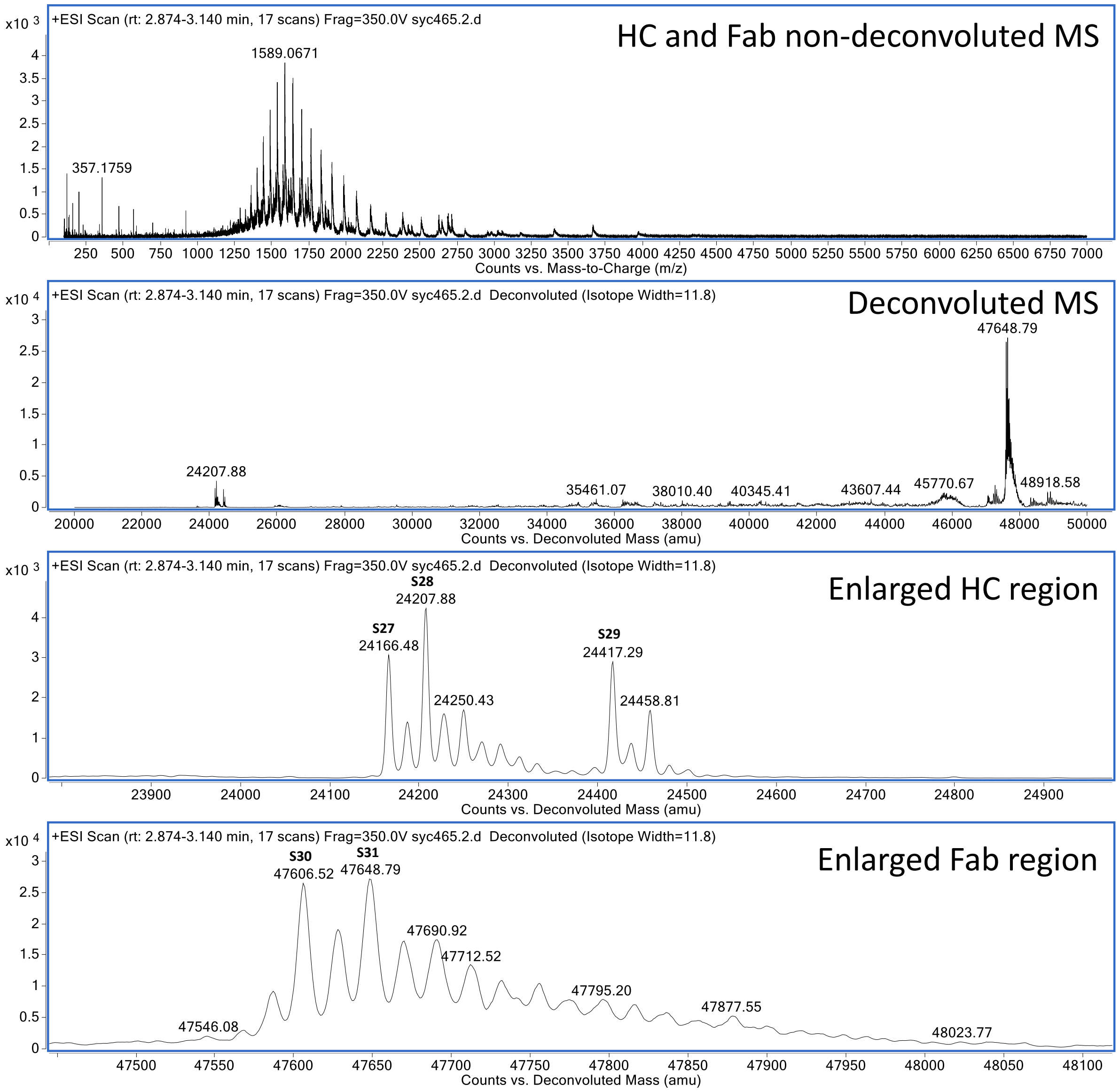

Figure S 16 LCMS of reaction of reduced Fab with SBTU **5**. **S26** expected mass: 23483 Da, observed mass: 24482 Da.**S27** expected mass: 24166 Da, observed mass: 24166 Da; **S28** expected mass: 24200 Da, observed mass: 24208 Da; **S29** expected mass: 24418 Da, observed mass: 24417 Da; **S30** expected mass: 47606 Da, observed mass: 47606 Da; **S31** expected mass: 47638 Da, observed mass: 47648 Da.

Reaction of reduced Fab with HOTT 6

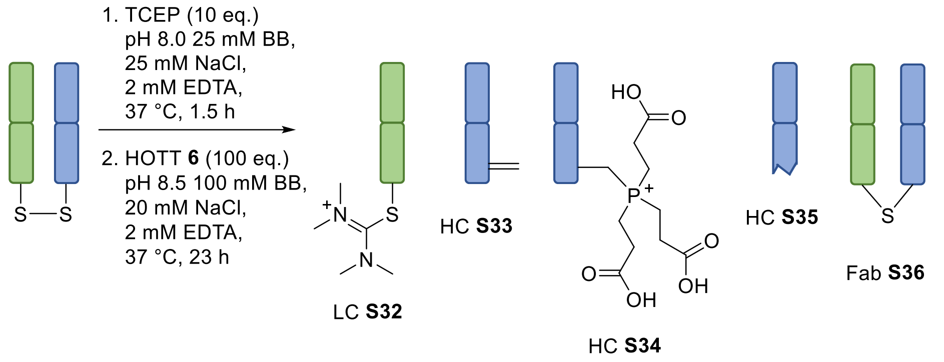

To Fab (25 µL, 100 µM) in pH 8.0 buffer (25 mM BB, 25 mM NaCl, 2 mM EDTA) was added TCEP (1.25 µL, 20 mM in H_2O, 10 eq.) and incubated at 37 °C for 1.5 h. Reaction mixture was diluted and purified (ZebaSpin) into pH 8.5 buffer (100 mM BB, 20 mM NaCl, 2 mM EDTA). To the resulting reduced Fab (100 µL, 25 µM) was added HOTT **6** (2.5 µL, 100 mM in MeCN, 100 eq.) and the reaction was incubated at 37 °C. Sample was desalted (ZebaSpin) and analysed using LCMS.

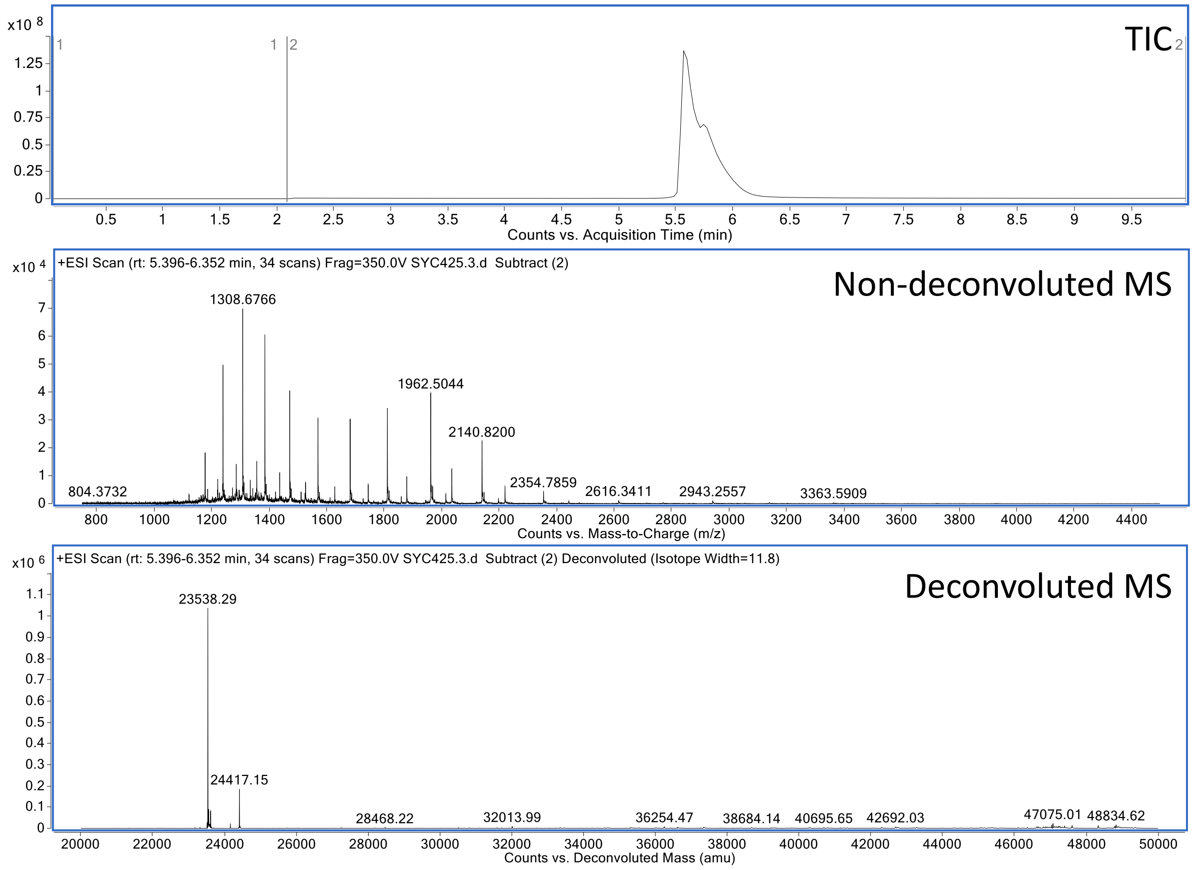

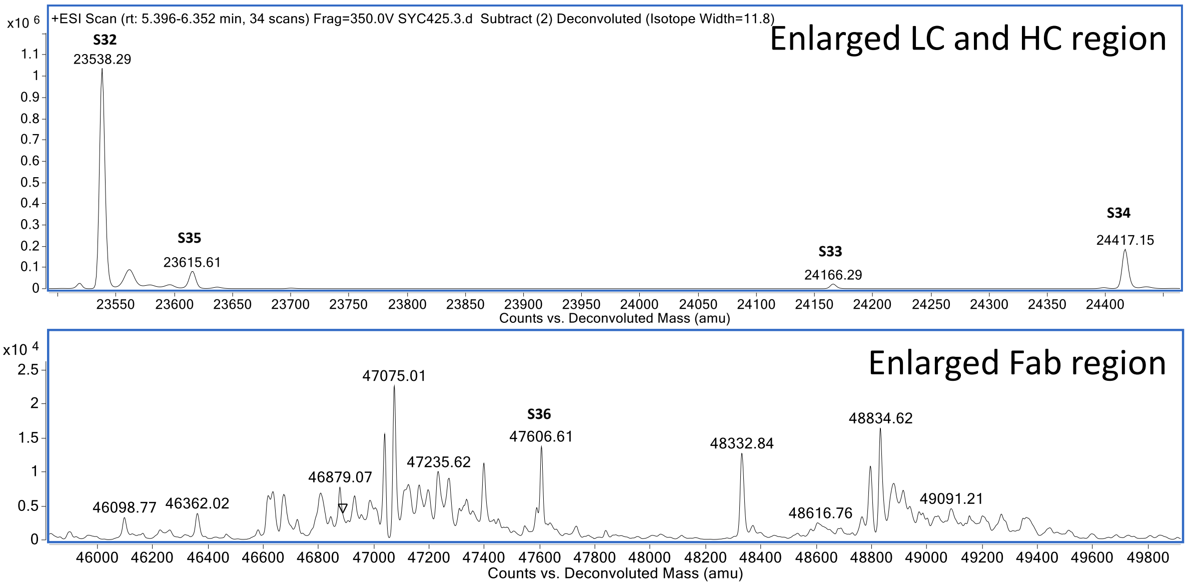

Figure S 17 LCMS of reaction of reduced Fab with HOTT **6**. **S32** expected mass: 23539 Da, observed mass: 23538 Da; **S33** expected mass: 24166 Da, observed mass: 24166 Da; **S34** expected mass: 24418 Da, observed mass: 24417 Da; **S35** expected mass: 23616 Da, observed mass: 23616 Da; **S36** expected mass: 47638 Da, observed mass: 23615 Da.

Reaction of reduced Fab with CDMI 7

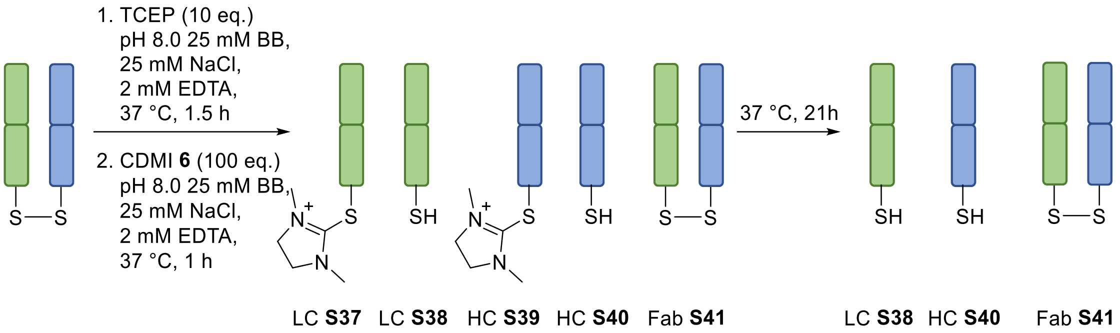

To Fab (20 µL, 100 µM) in pH 8.0 buffer (25 mM BB, 25 mM NaCl, 2 mM EDTA) was added TCEP (1 µL, 20 mM in H_2O, 10 eq.) and incubated at 37 °C for 1.5 h. Reaction mixture was diluted and purified (ZebaSpin) into pH 8.0 buffer (25 mM BB, 25 mM NaCl, 2 mM EDTA). To the resulting reduced Fab (100 µL, 20 µM) was added CDMI **7** (2 µL, 100 mM in MeCN, 100 eq.) and the reaction incubated at 37 °C for 22 h. Sample was desalted (ZebaSpin) and analysed using LCMS.

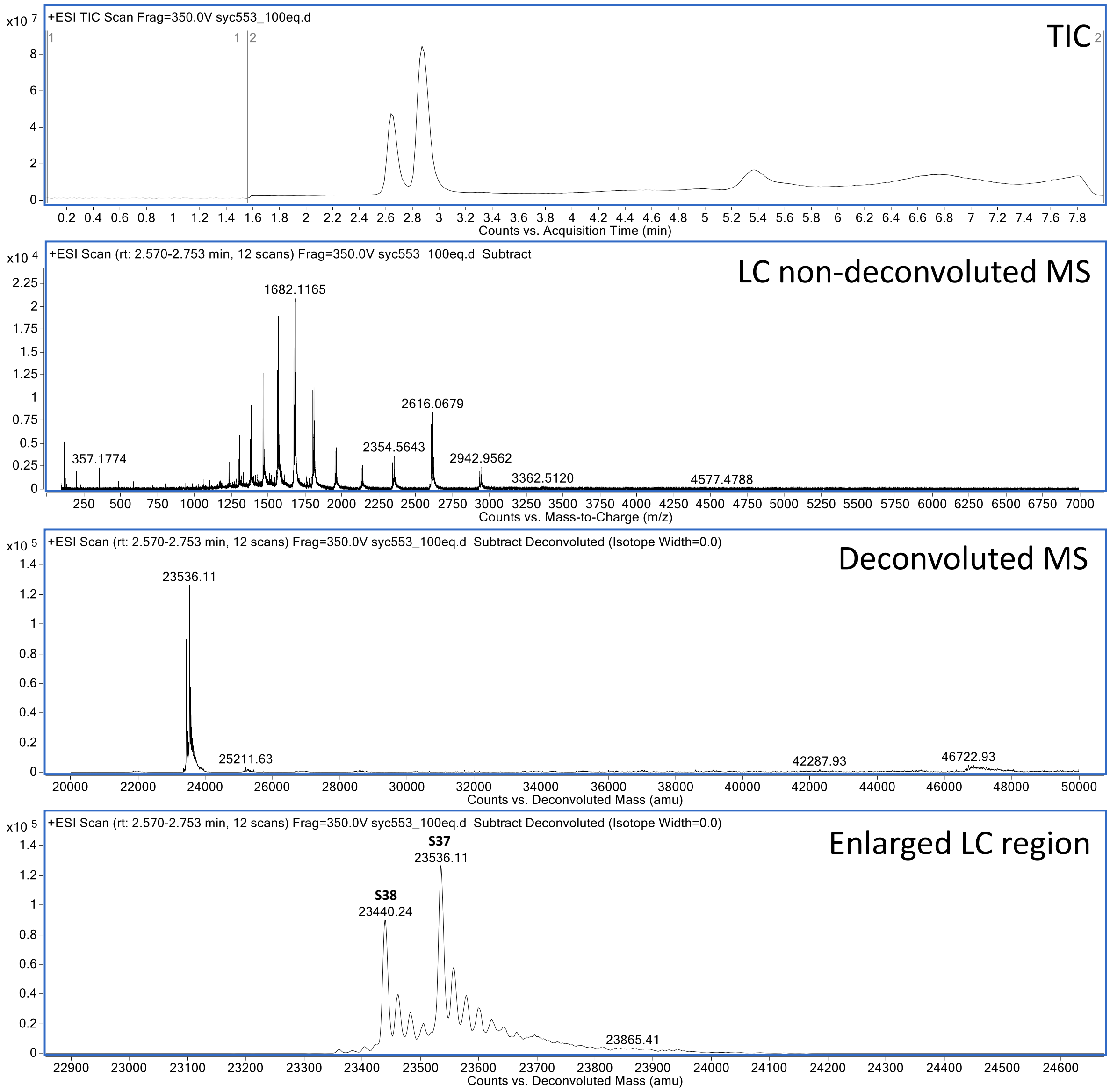

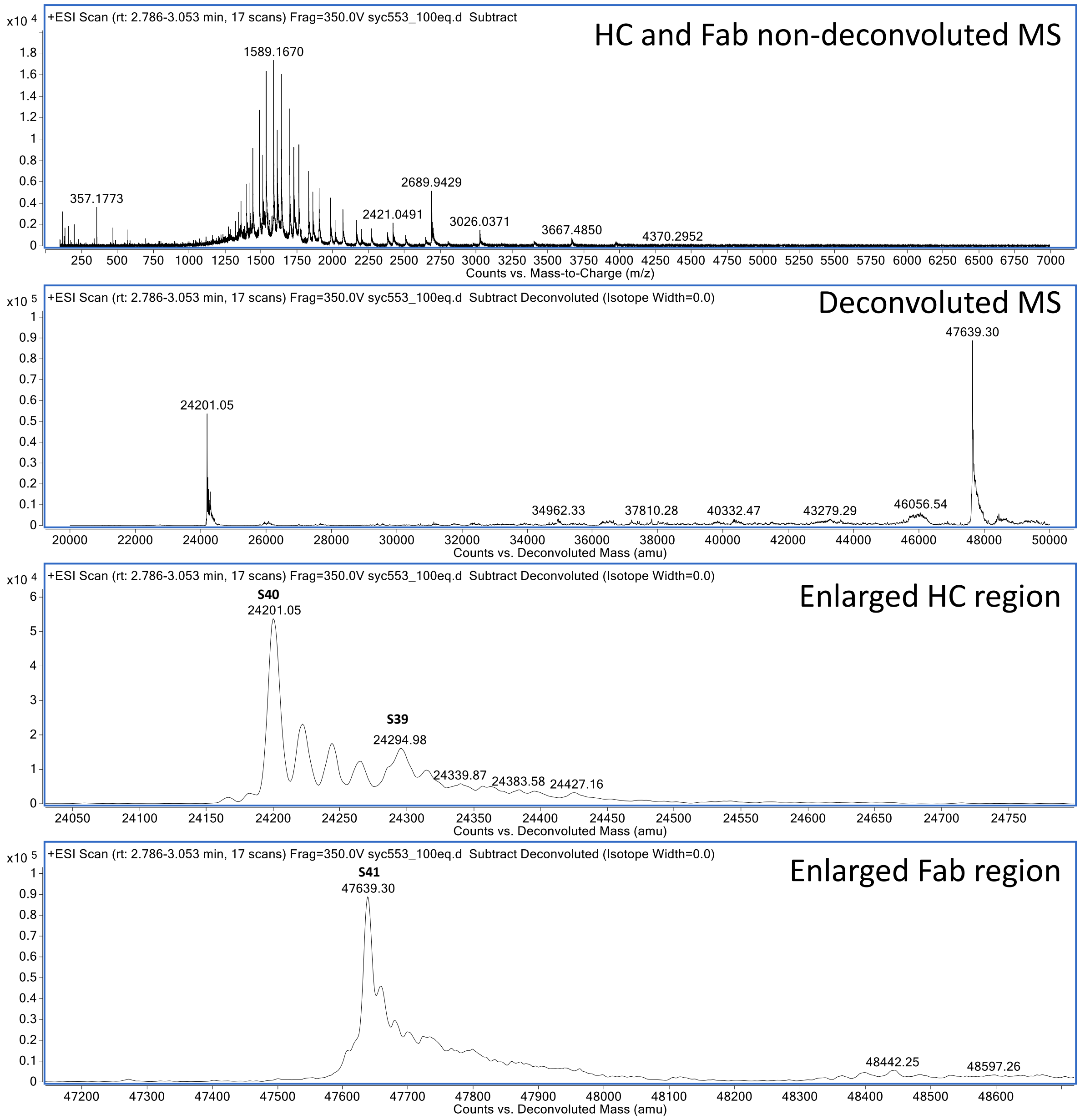

Figure S 18 LCMS of reaction of reduced Fab with CDMI **7** at 1 h timepoint. **S37** expected mass: 23537 Da, observed mass: 23536 Da; **S38** expected mass: 23440 Da, observed mass: 23440 Da. **S39** expected mass: 24297 Da, observed mass: 24294 Da; **S40** expected mass: 24200 Da, observed mass: 24201 Da; **S41** expected mass: 47640 Da, observed mass: 47639 Da.

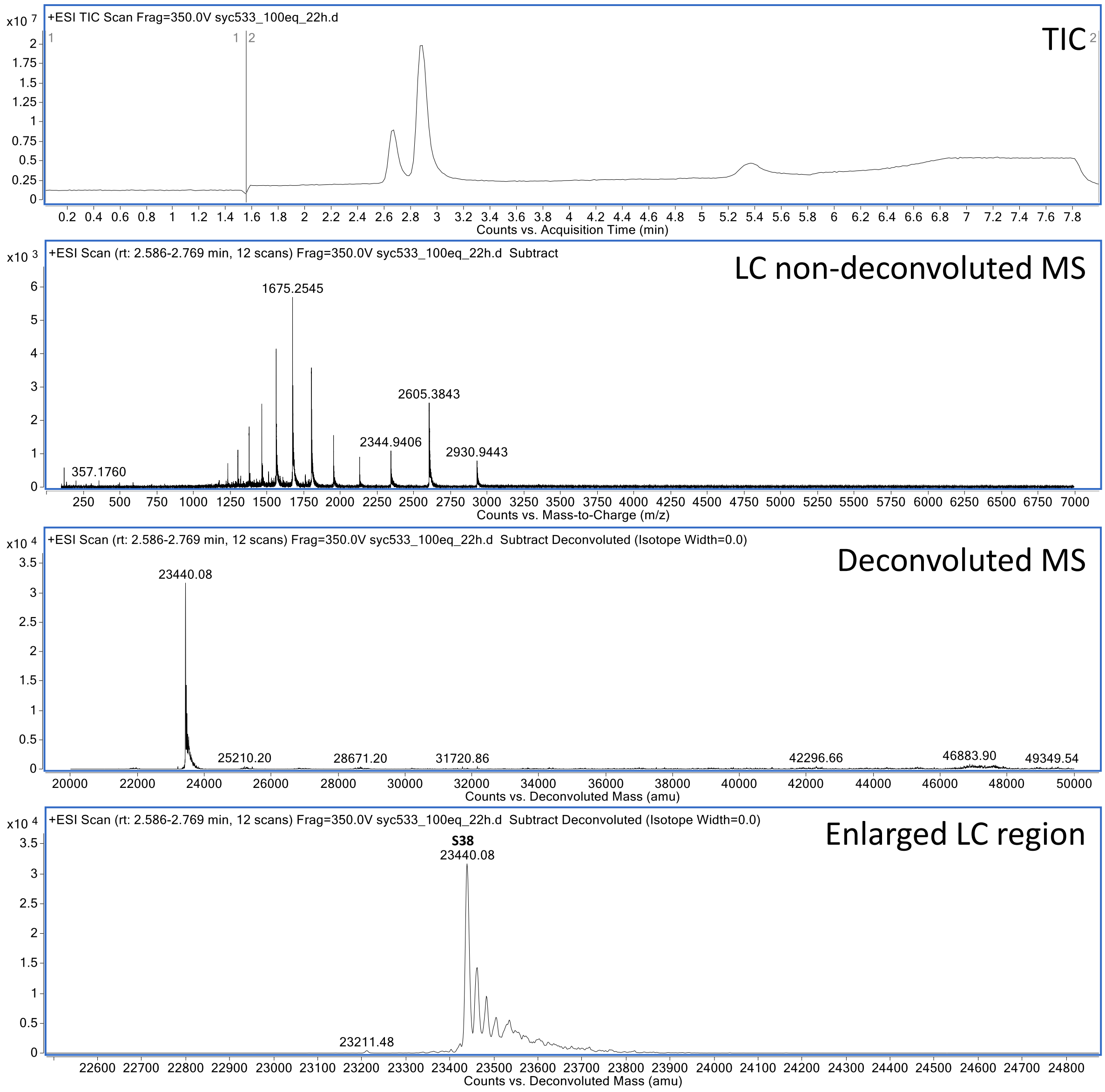
+ESI TIC Scan Frag=350.0V syc533_100eq_22h.d
TIC
Counts vs. Acquisition Time (min)
+ESI Scan (rt: 2.586-2.769 min, 12 scans) Frag=350.0V syc533_100eq_22h.d Subtract
LC non-deconvoluted MS
1675.2545
2605.3843
2344.9406
2930.9443
357.1760
Counts vs. Mass-to-Charge (m/z)
+ESI Scan (rt: 2.586-2.769 min, 12 scans) Frag=350.0V syc533_100eq_22h.d Subtract Deconvoluted (Isotope Width=0.0)
Deconvoluted MS
23440.08
25210.20
28671.20
31720.86
42296.66
46883.90
49349.54
Counts vs. Deconvoluted Mass (amu)
+ESI Scan (rt: 2.586-2.769 min, 12 scans) Frag=350.0V syc533_100eq_22h.d Subtract Deconvoluted (Isotope Width=0.0)
Enlarged LC region
S38
23440.08
23211.48
Counts vs. Deconvoluted Mass (amu)

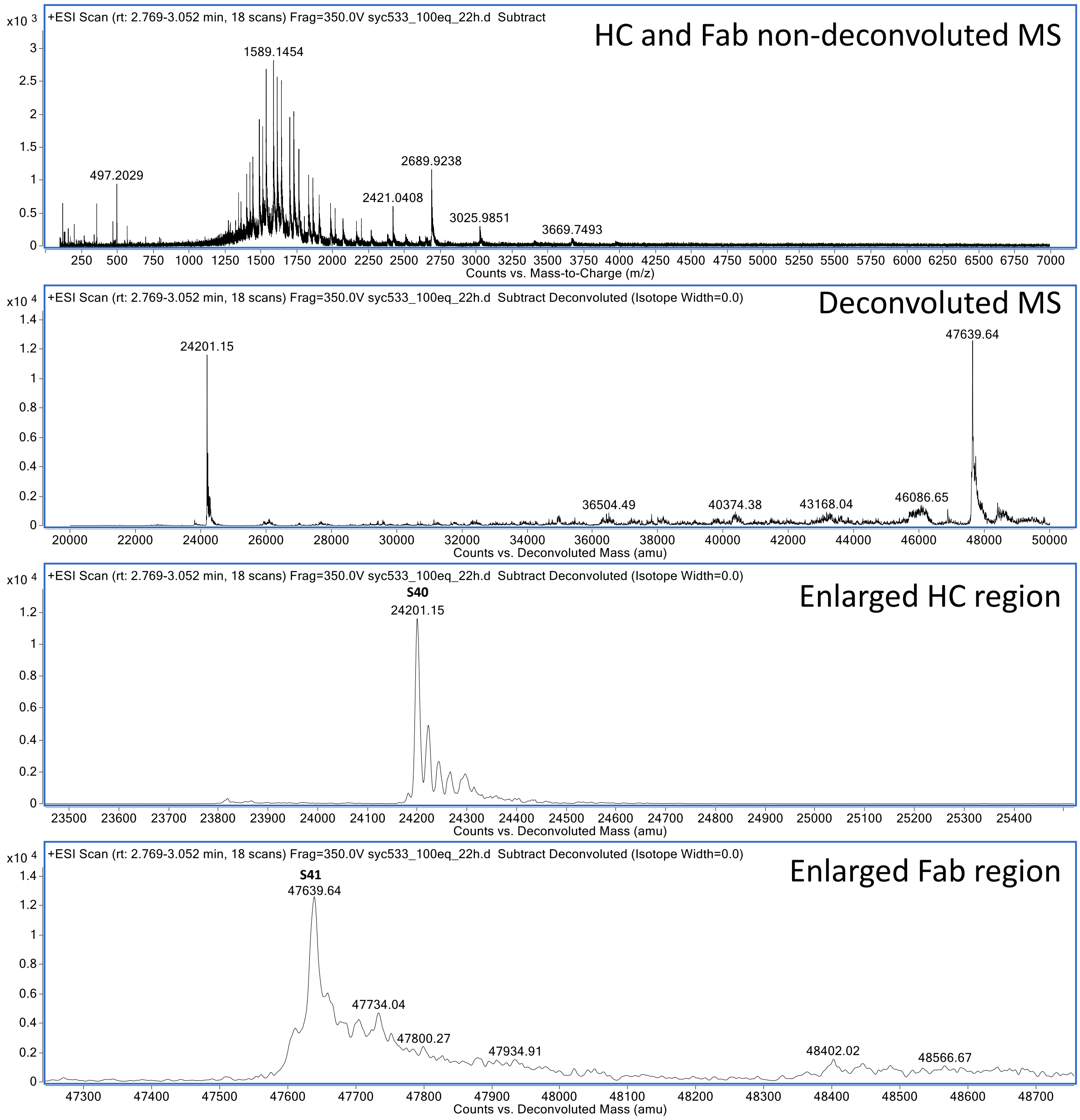

Figure S 19 LCMS of reaction of reduced Fab with CDMI **7** at 22 h timepoint. **S38** expected mass: 23440 Da, observed mass: 23440 Da. **S40** expected mass: 24200 Da, observed mass: 24201 Da; **S41** expected mass: 47640 Da, observed mass: 47639 Da.

Reaction of reduced Fab with CDMP 8

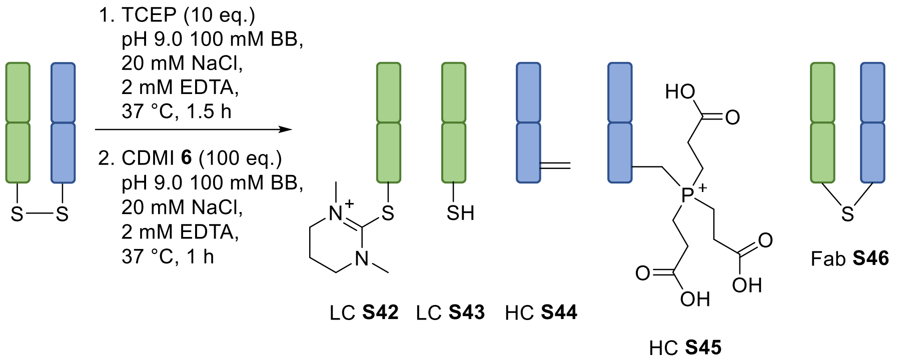

To Fab (20 µL, 100 µM) in pH 8.0 buffer (25 mM BB, 25 mM NaCl, 2 mM EDTA) was added TCEP (1 µL, 20 mM in H_2O, 10 eq.) and incubated at 37 °C for 1.5 h. Reaction mixture was diluted and purified (ZebaSpin) into pH 8.0 buffer (25 mM BB, 25 mM NaCl, 2 mM EDTA). To the resulting reduced Fab (100 µL, 20 µM) was added CDMI **7** (2 µL, 100 mM in MeCN, 100 eq.) and the reaction incubated at 37 °C for 22 h. Sample was desalted (ZebaSpin) and analysed using LCMS.

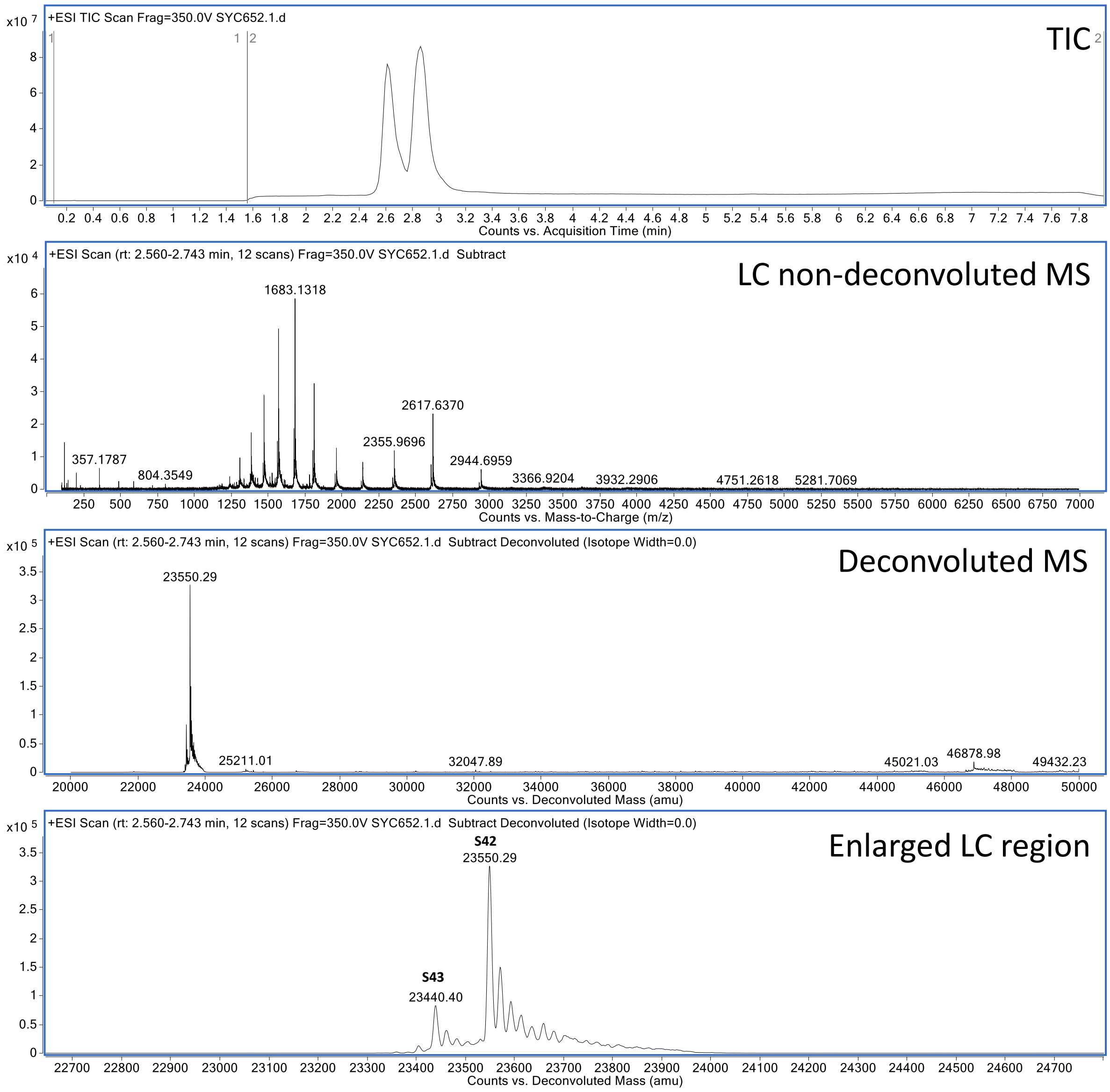

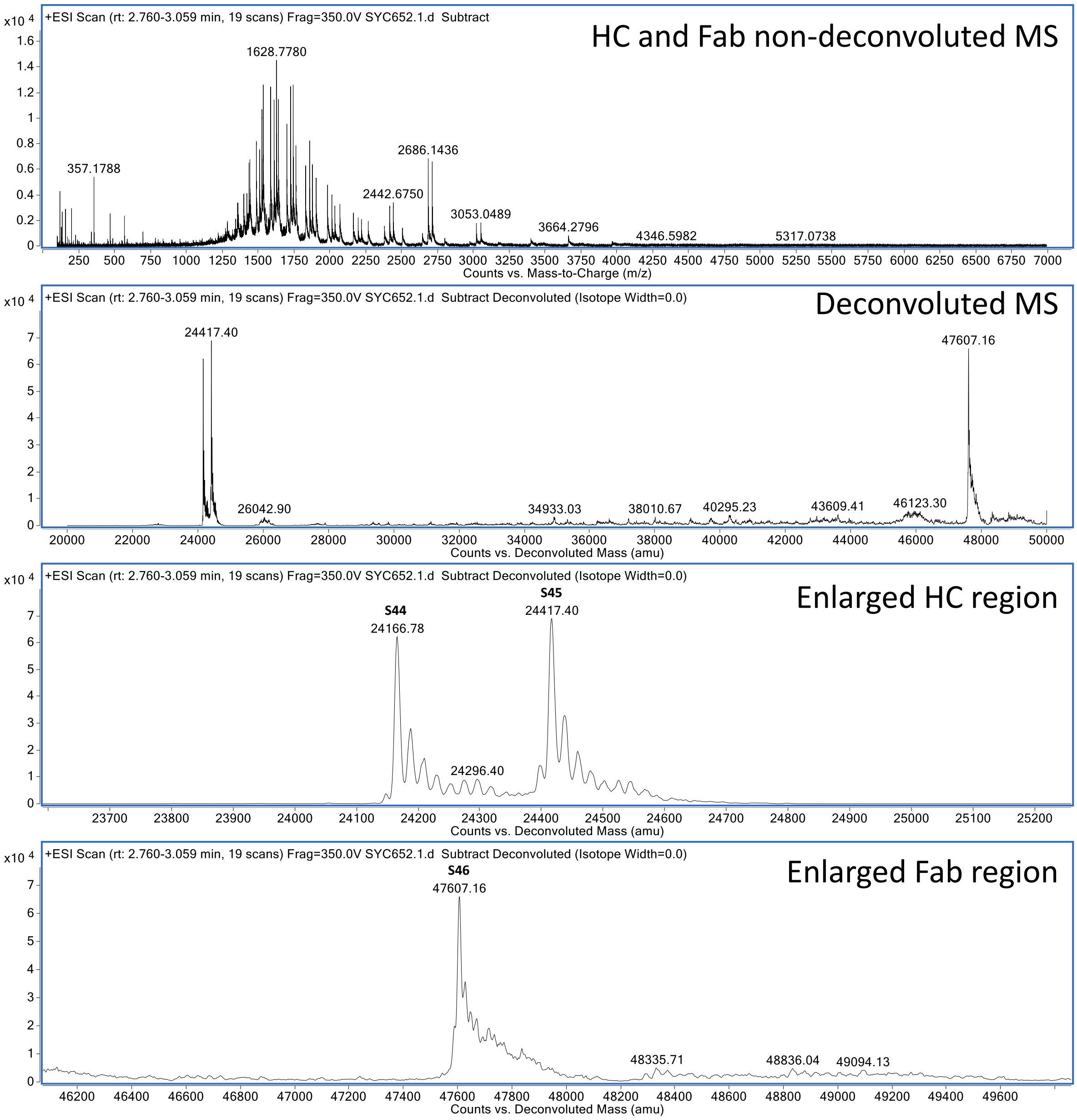

Figure S 20 LCMS of reaction of reduced Fab with CDMP **8**. **S42** expected mass: 23551 Da, observed mass: 23550 Da; **S43** expected mass: 23440 Da, observed mass: 23440 Da. **S44** expected mass: 24166 Da, observed mass: 24167 Da; **S45** expected mass: 23166 Da, observed mass: 23167 Da **S46** expected mass: 47606 Da, observed mass: 47607 Da.

Screening of nucleophiles

General procedure

To Fab (25 µL, 100 µM) in pH 8.0 buffer (25 mM BB, 25 mM NaCl, 2 mM EDTA) was added TCEP (1.25 µL, 20 mM in H_2O, 10 eq.) and incubated at 37 °C for 1.5 h. Reaction mixture was diluted and purified (ZebaSpin) into pH 7.4 PBS (100 mM PB, 20 mM NaCl). To the resulting reduced Fab (100 µL, 20 µM) was added EDC **4** (100 mM in H_2O, 100 eq.) and the reaction was incubated at 37 °C for 1 h, followed by addition of nucleophiles (thiols, amines, or phosphines). Sample was desalted (ZebaSpin) and analysed using LCMS.

2-mercaptoethanol (BME)

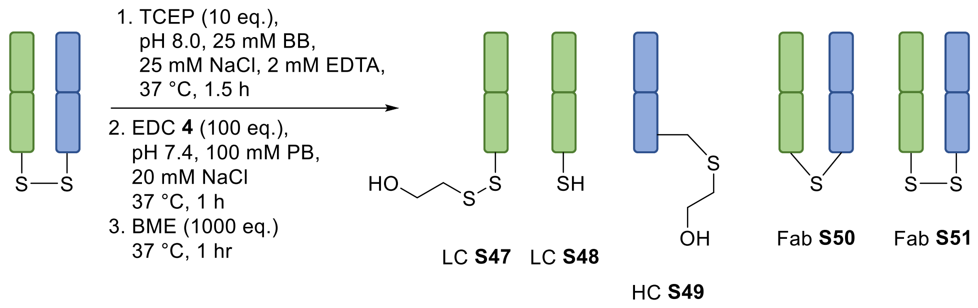

BME (10 µL, 200 mM in MeCN, 1000 eq.) at 37 °C for 1 h.

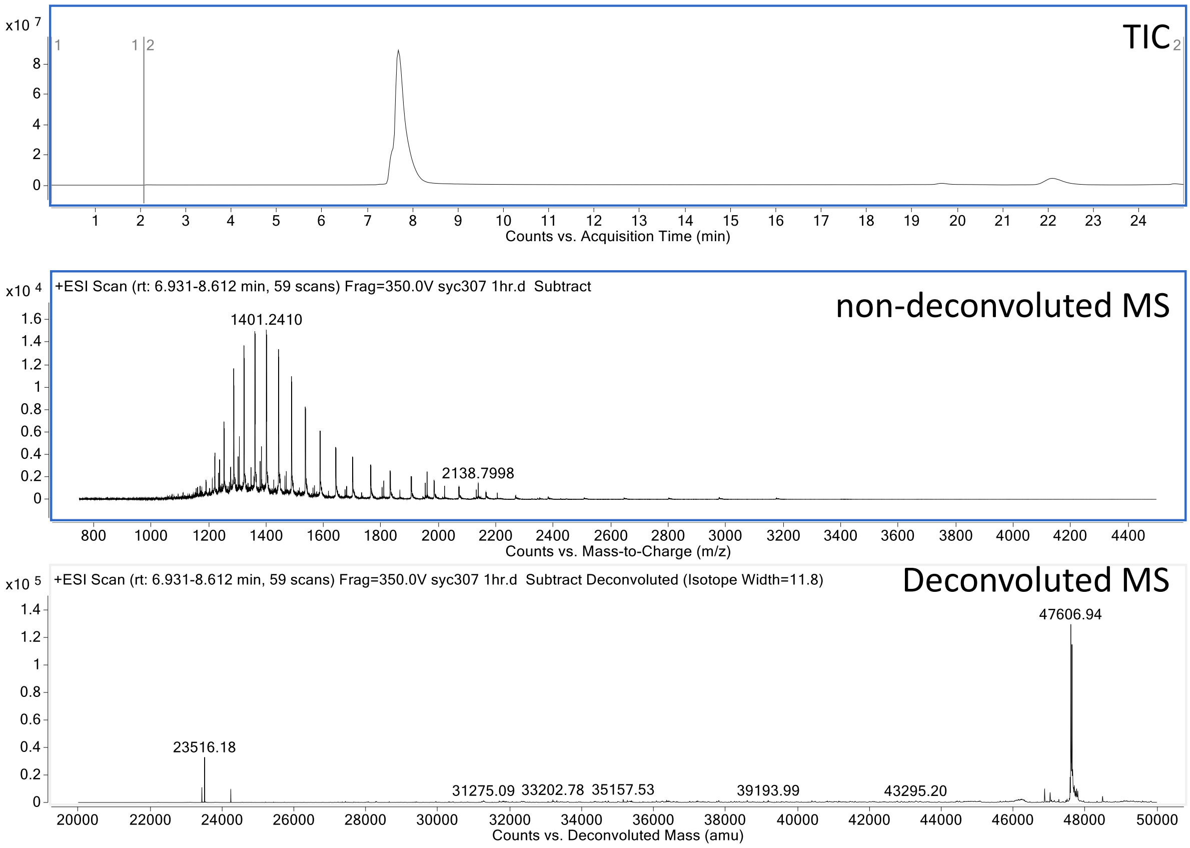

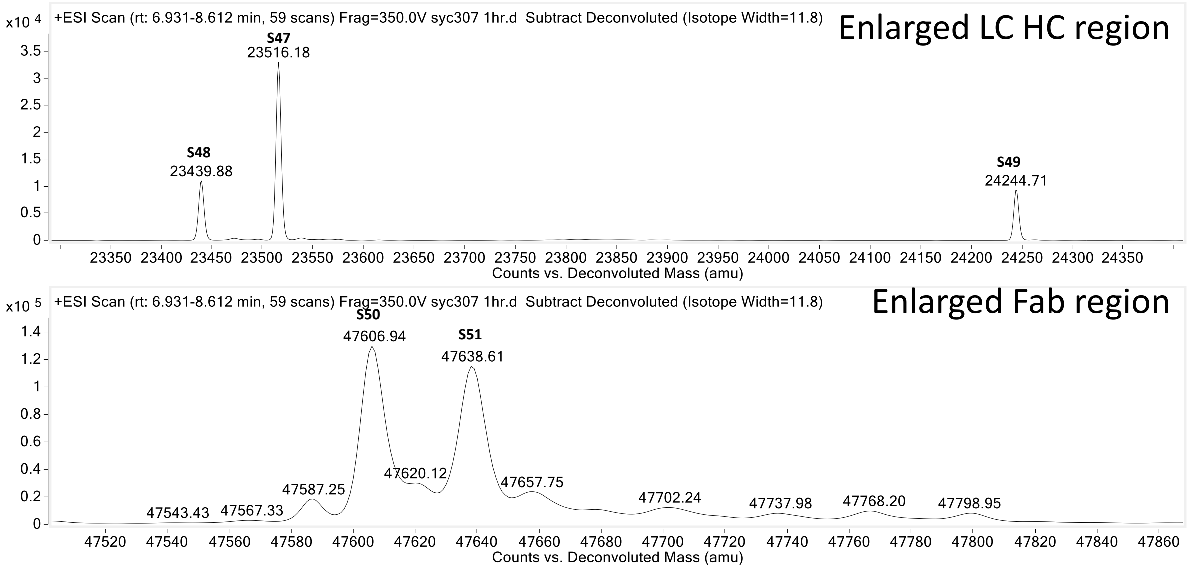

Figure S 21 LCMS of reaction of reduced Fab, EDC **4**, and BME. **S47** expected mass: 23516 Da, observed mass: 23516 Da; **S48** expected mass: 23440 Da, observed mass: 23440 Da. **S49** expected mass: 24244 Da, observed mass: 24245 Da; **S50** expected mass: 47606 Da, observed mass: 47607 Da; **S51** expected mass: 47640 Da, observed mass: 47639 Da.

Dithiothreitol (DTT)

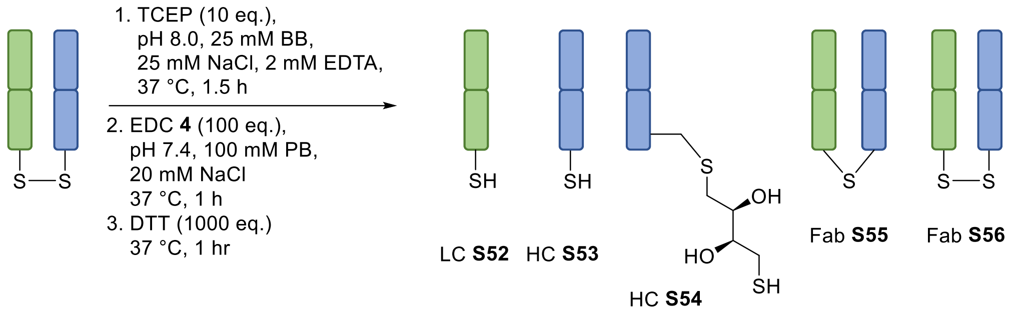

DTT (10 µL, 200 mM in MeCN, 1000 eq.) at 37 °C for 1 h.

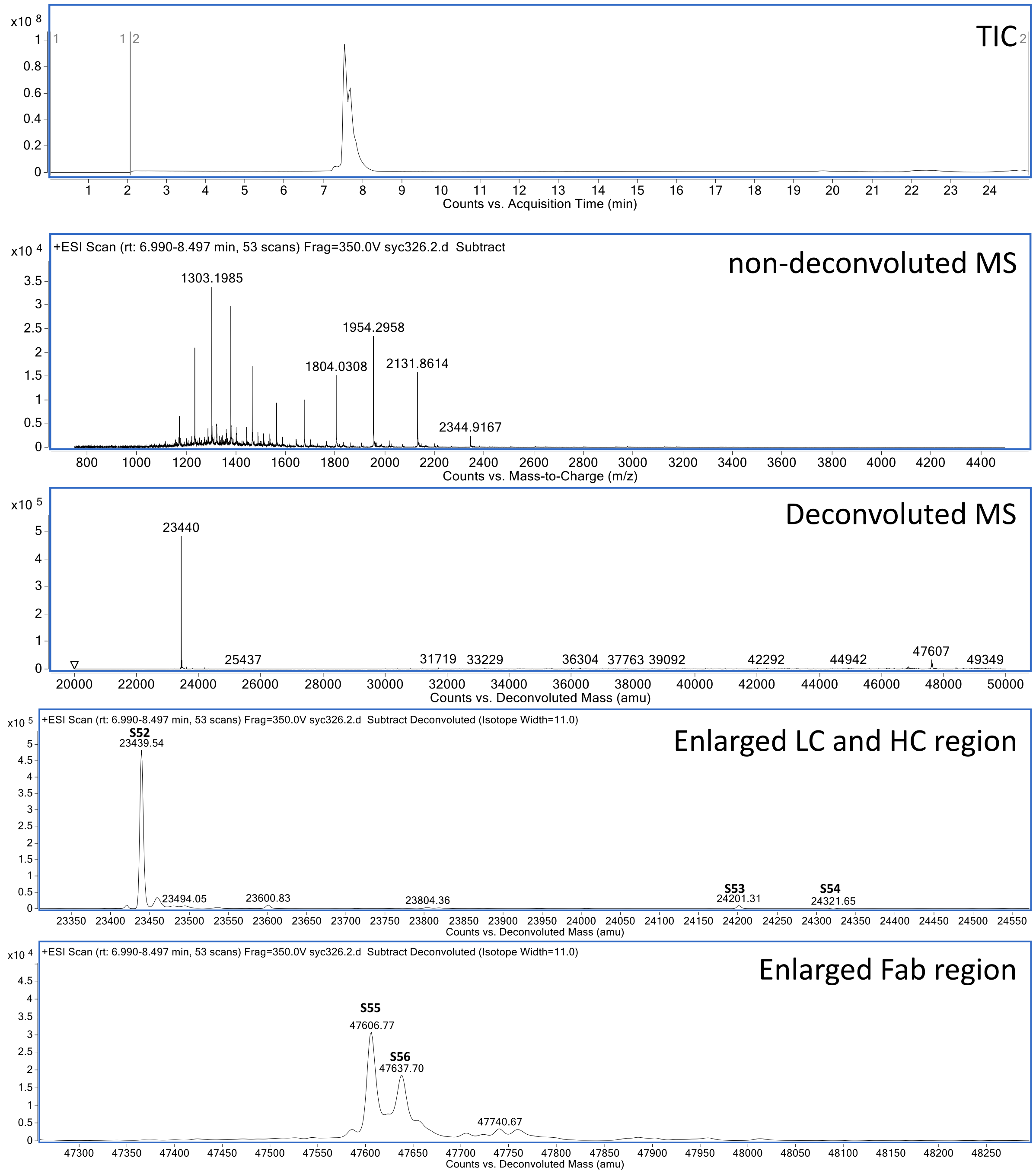

Figure S 22 LCMS of reaction of reduced Fab, EDC **4**, and DTT. **S52** expected mass: 23440 Da, observed mass: 23440 Da; **S53** expected mass: 24200 Da, observed mass: 24201 Da **S54** expected mass: 24320 Da, observed mass: 24322 Da; **S55** expected mass: 47606 Da, observed mass: 47607 Da; **S56** expected mass: 47640 Da, observed mass: 47639 Da.

4-mercaptophenylacetic acid (MPAA)

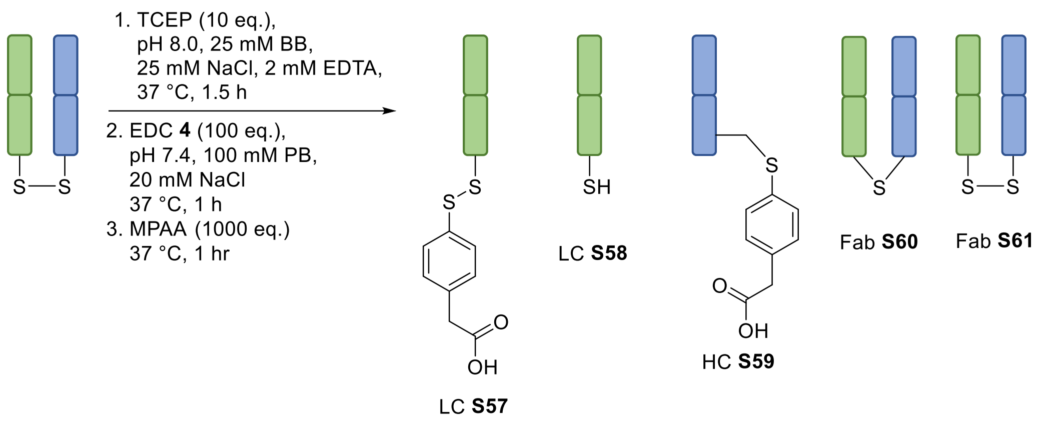

MPAA (10 µL, 200 mM in MeCN, 1000 eq.) at 37 °C for 1 h.

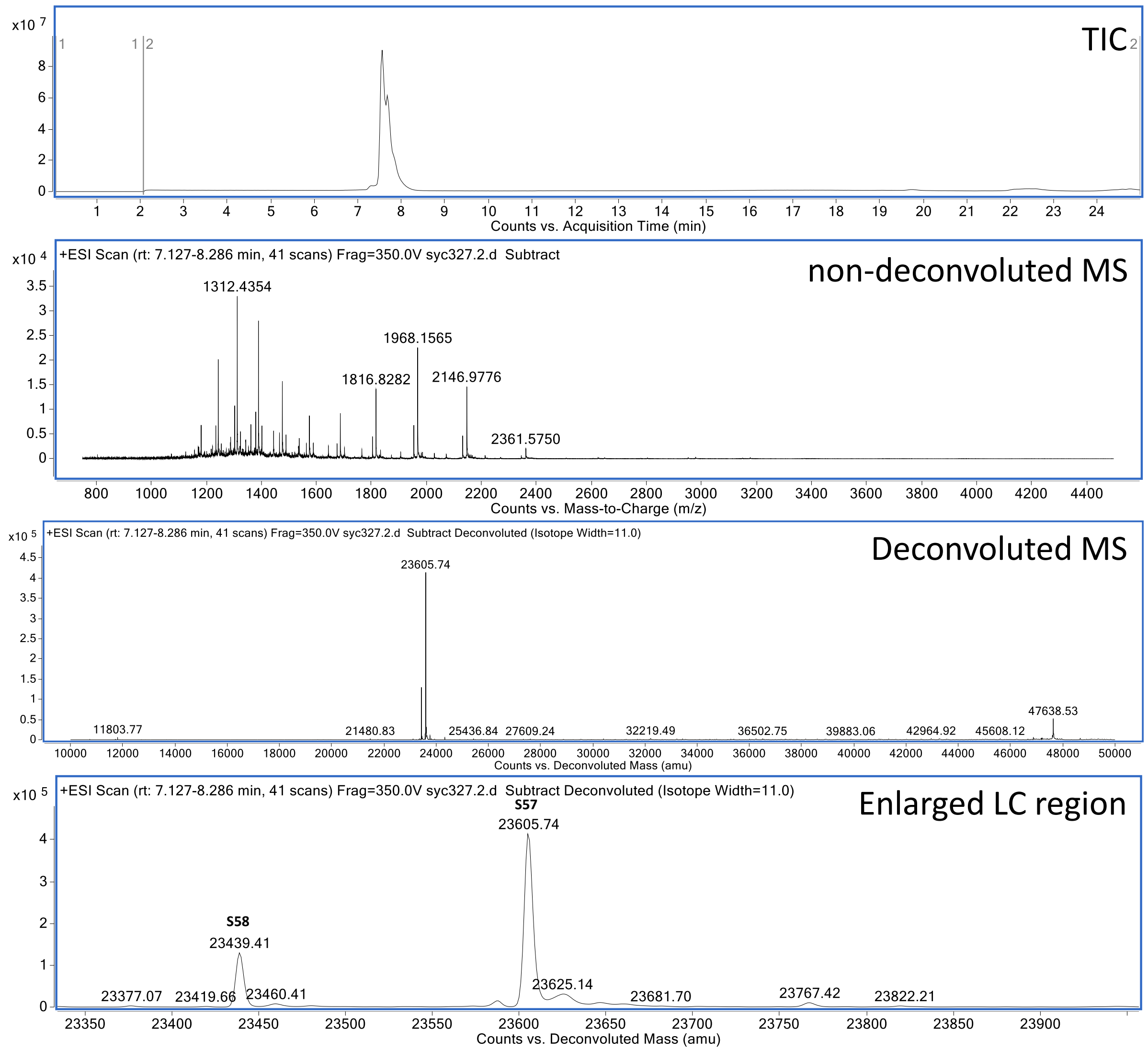

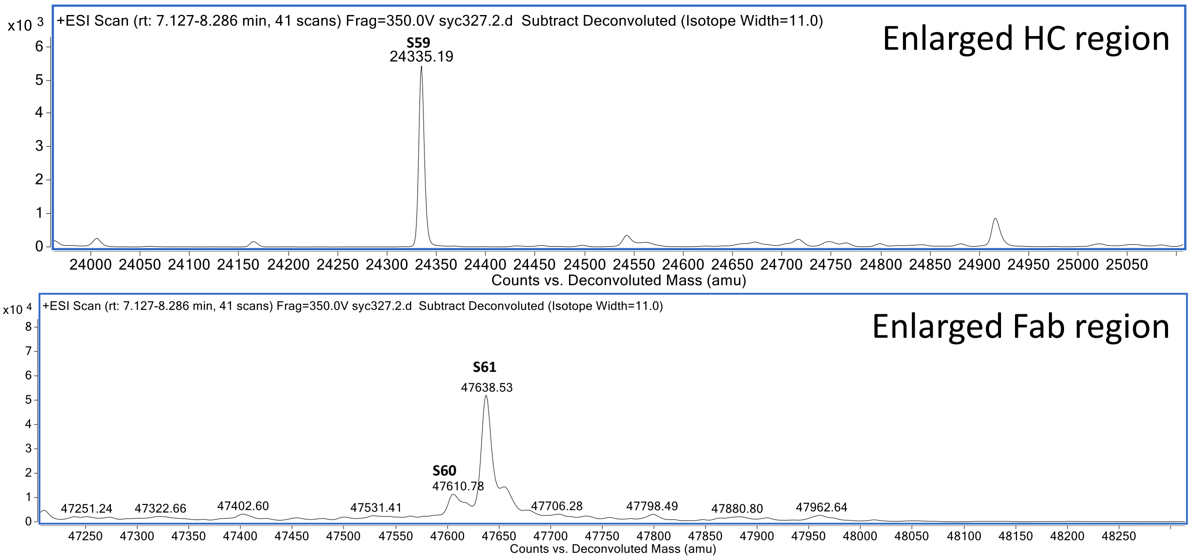

Figure S 23 LCMS of reaction of reduced Fab, EDC **4**, and MPAA. **S57** expected mass: 23606 Da, observed mass: 23605 Da; **S58** expected mass: 23440 Da, observed mass: 23440 Da; **S59** expected mass: 24334 Da, observed mass: 24335 Da; **S60** expected mass: 47606 Da, observed mass: 47610 Da; **S61** expected mass: 47640 Da, observed mass: 47639 Da.

Benzylamine

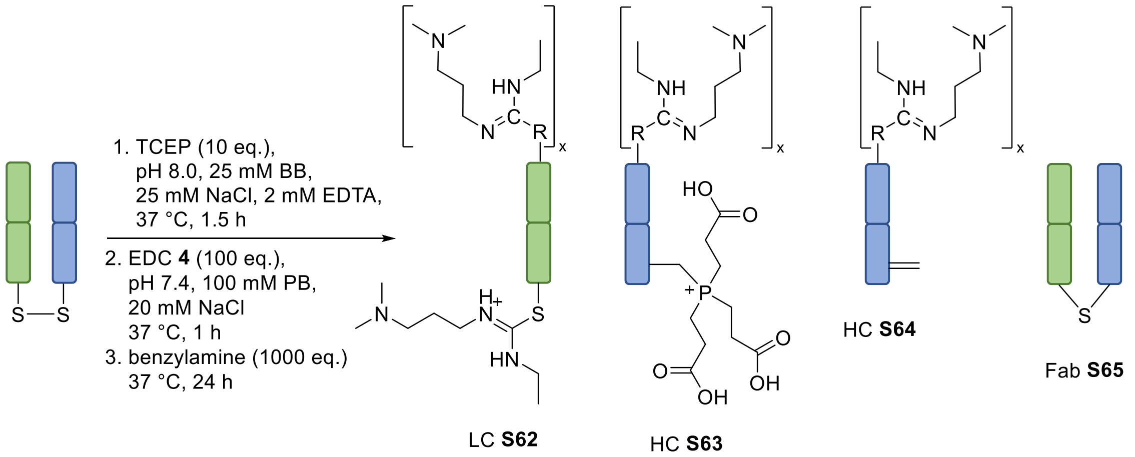

Benzylamine (4 µL, 500 mM in MeCN, 1000 eq.) at 37 °C for 24 h.

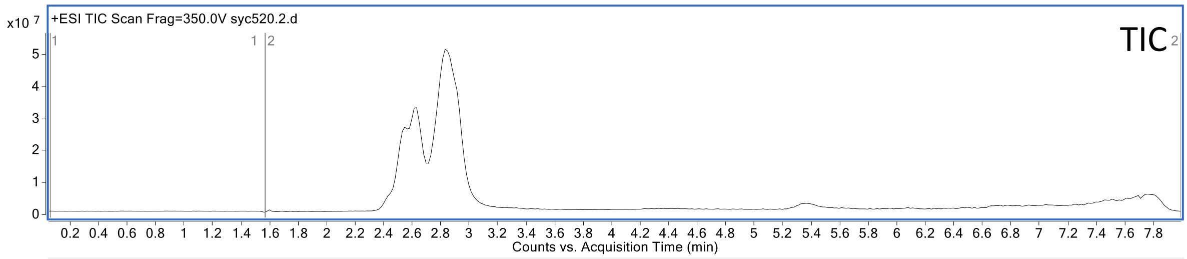

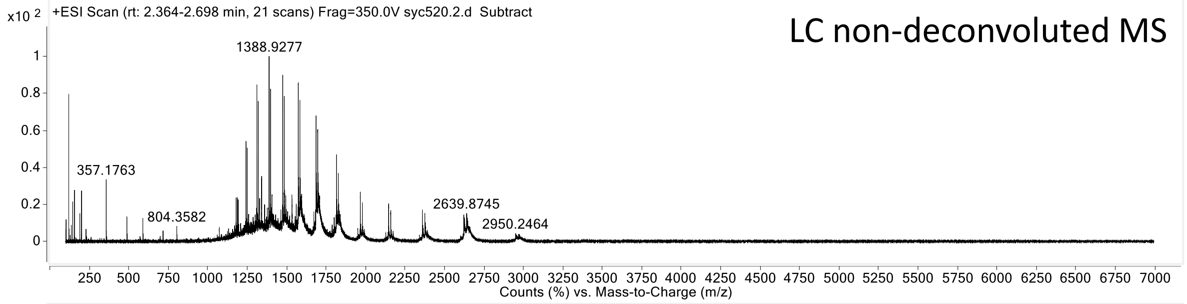

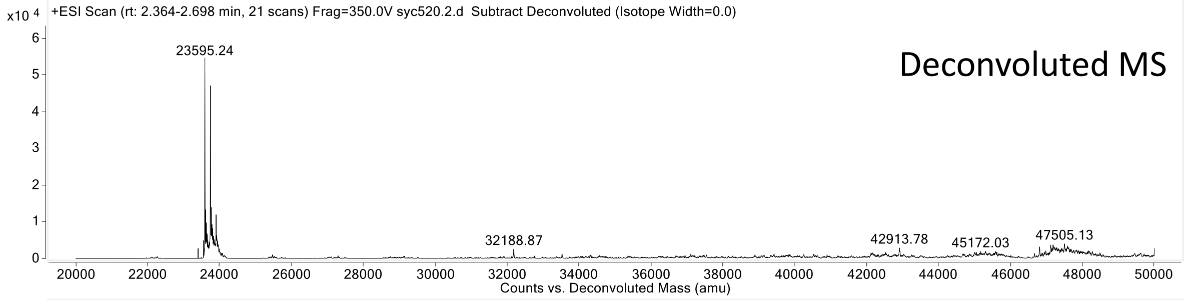

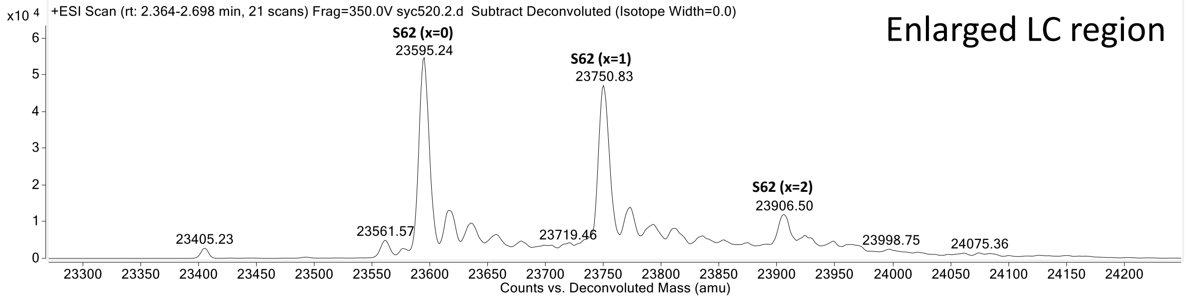

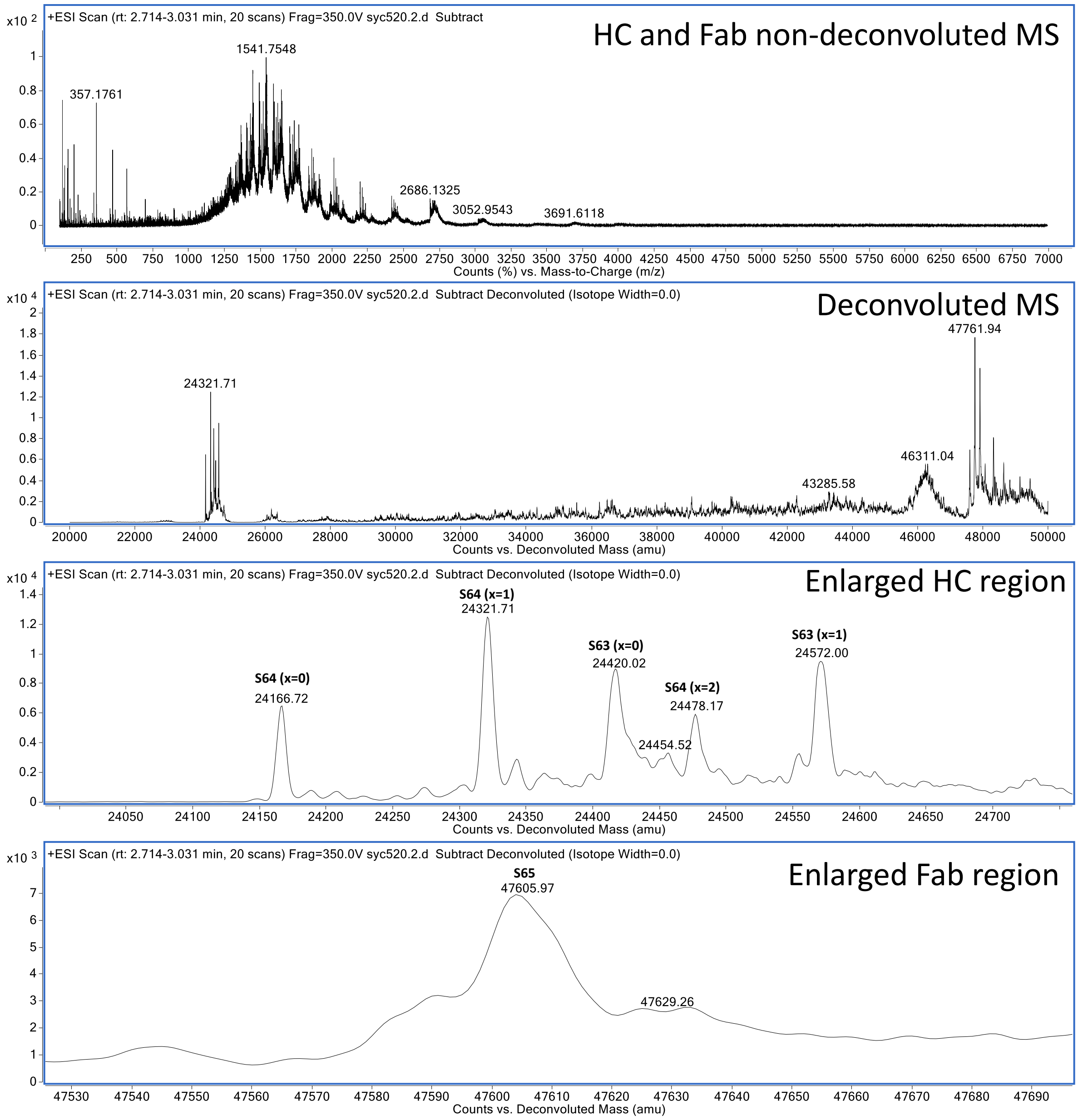

Figure S 24 LCMS of reaction of reduced Fab, EDC **4**, and benzylamine. **S62** expected mass: 23596, 23752, 23908 Da (x=0,1,2), observed mass: 23595, 23751, 23907 Da; **S63** expected mass: 24417, 24573 Da (x=0,1), observed mass: 24420, 24572 Da (x=0,1); **S64** expected mass: 24166, 24322, 23478 Da (x=0,1,2), observed mass: 24167, 24322,24478 Da; **S65** expected mass: 47606 Da, observed mass: 47606 Da.

Piperidine

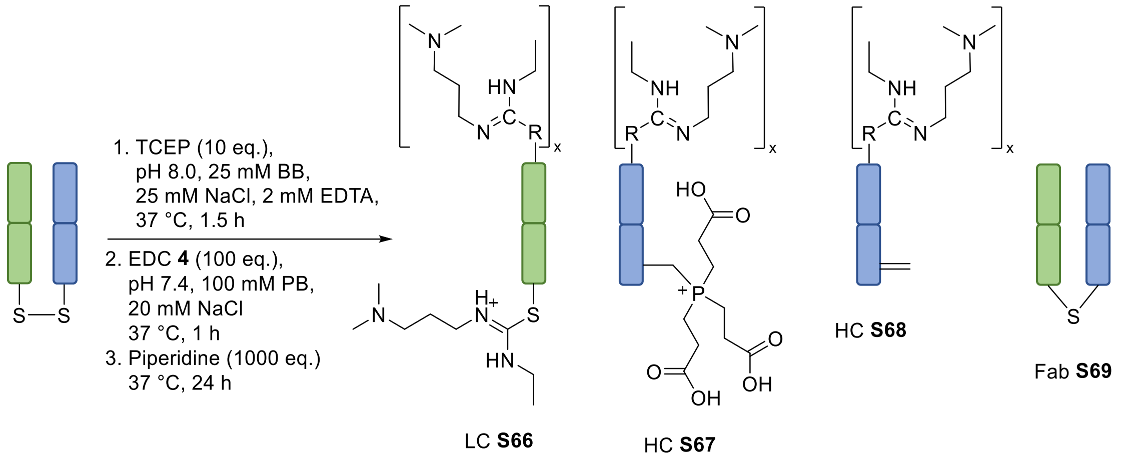

Piperidine (4 µL, 500 mM in MeCN, 1000 eq.) at 37 °C for 24 h.

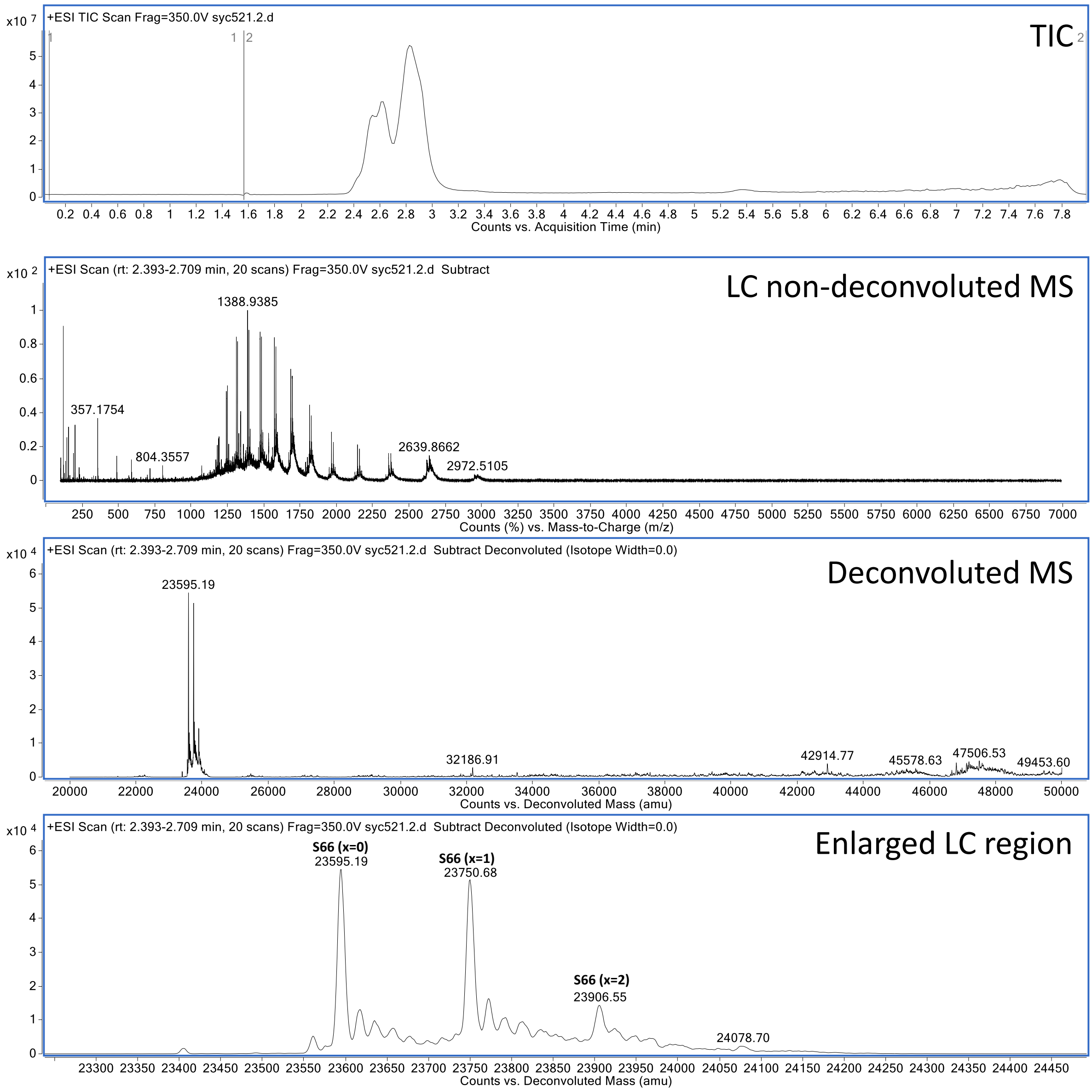

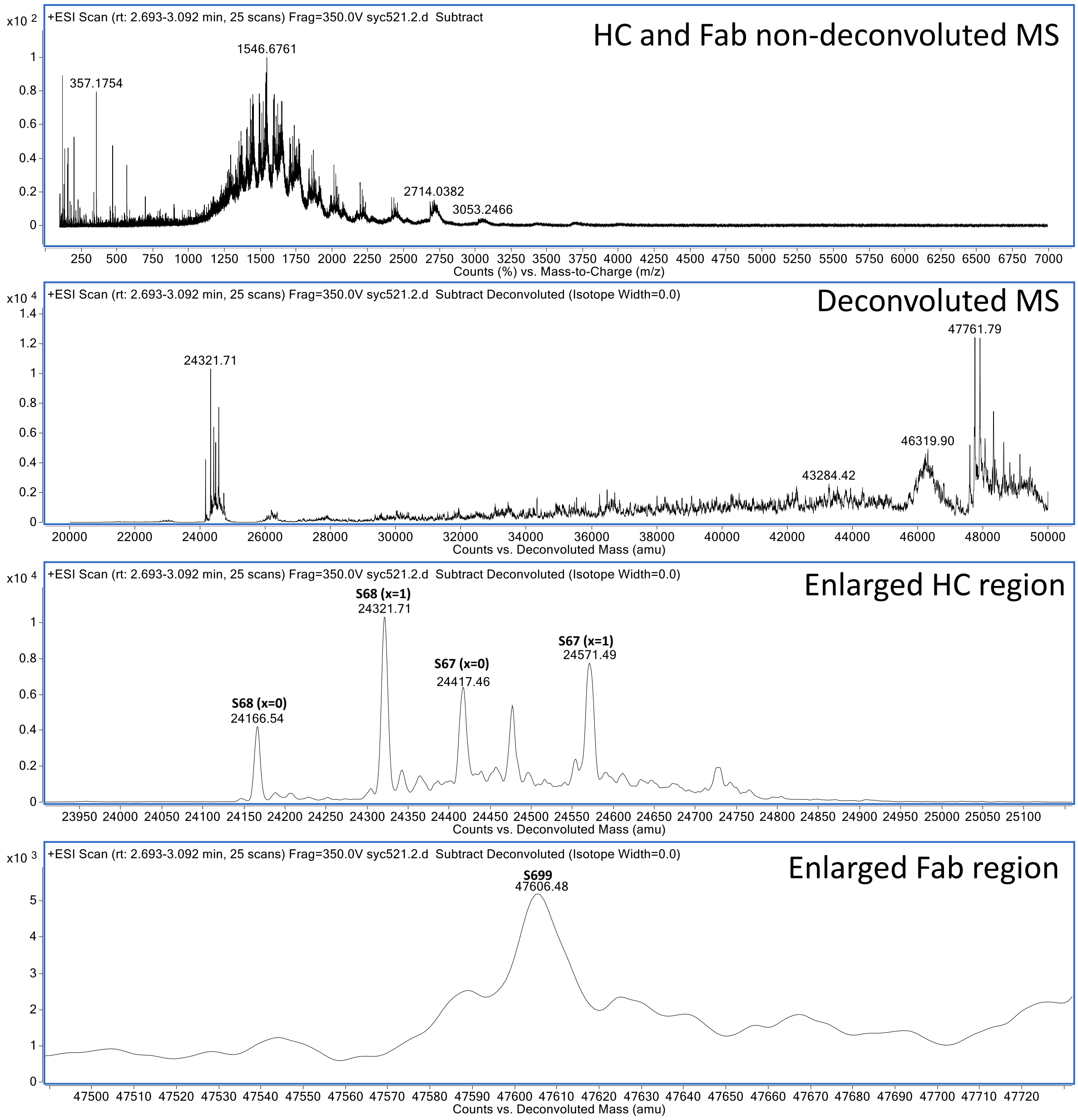

Figure S 25 LCMS of reaction of reduced Fab, EDC **4**, and piperidine. **S66** expected mass: 23596, 23752, 23908 Da (x=0,1,2), observed mass: 23595, 23751, 23907 Da; **S67** expected mass: 24417, 24573 Da (x=0,1), observed mass: 24417, 24571 Da (x=0,1); **S68** expected mass: 24166, 24322 Da (x=0,1), observed mass: 24167, 24322 Da; **S69** expected mass: 47606 Da, observed mass: 47606 Da.

***p*-Anisidine**

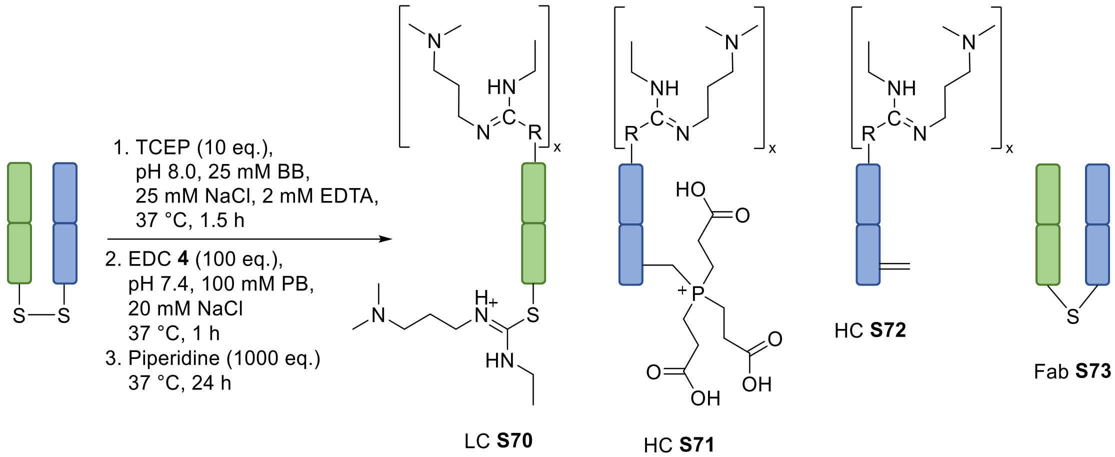

p-Anisidine (4 µL, 500 mM in MeCN, 1000 eq.) at 37 °C for 24 h.

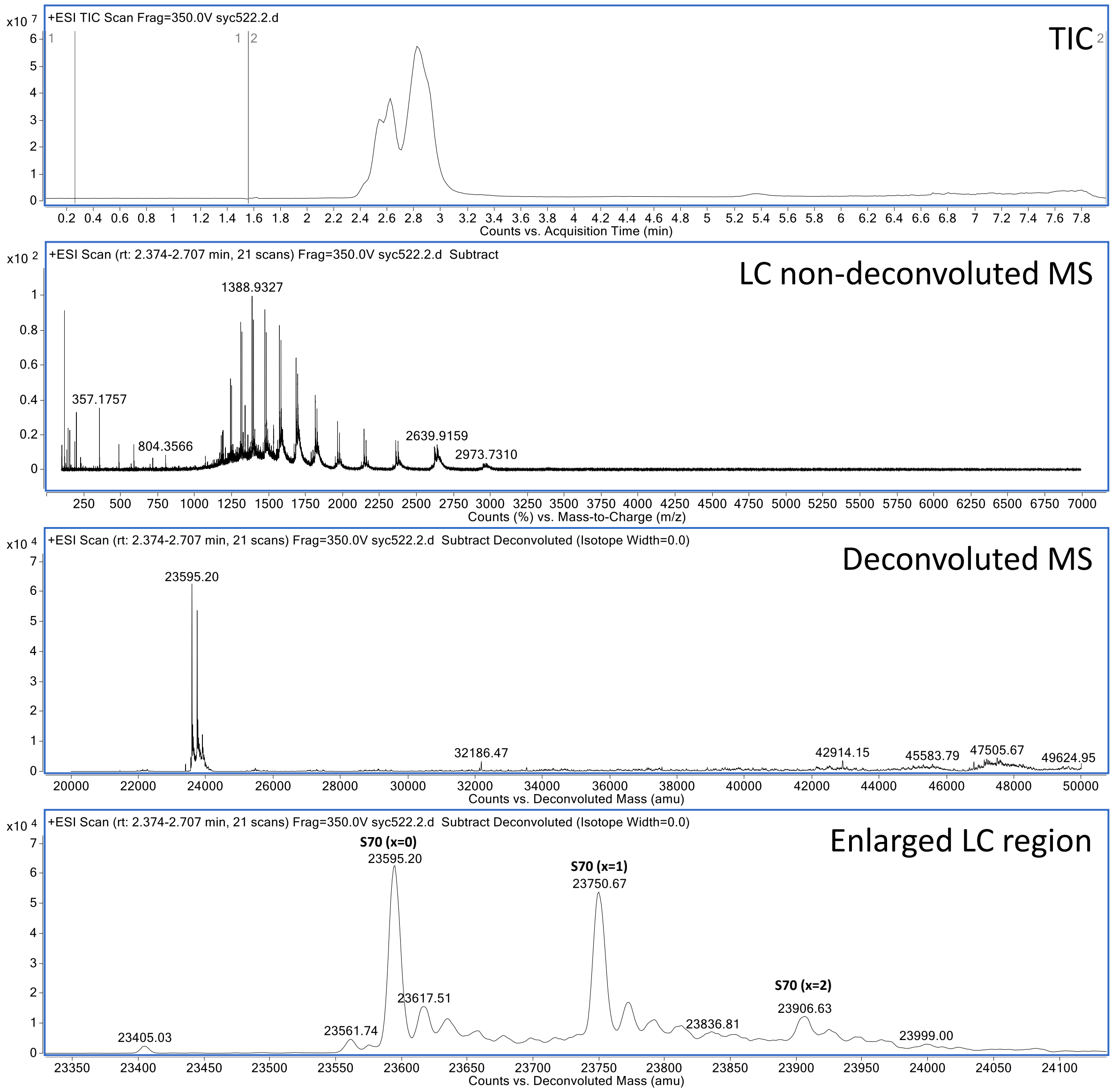

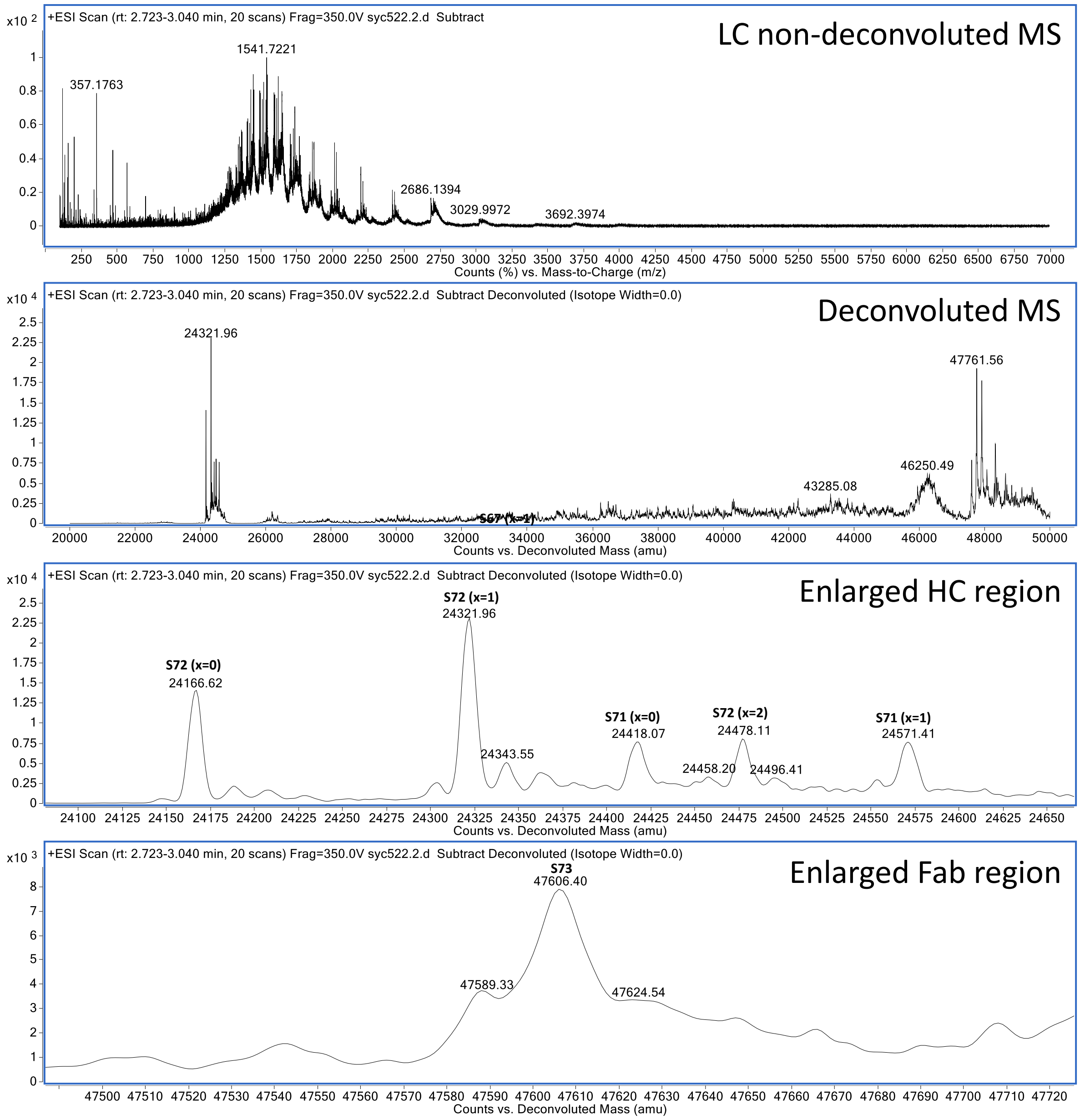

Figure S 26 LCMS of reaction of reduced Fab, EDC **4**, and *p*-anisidine. **S70** expected mass: 23596, 23752, 23908 Da (x=0,1,2), observed mass: 23595, 23751, 23907 Da; **S71** expected mass: 24417, 24573 Da (x=0,1), observed mass: 24418, 24571 Da (x=0,1); **S72** expected mass: 24166, 24322, 23478 Da (x=0,1,2), observed mass: 24167, 24322, 24478 Da; **S73** expected mass: 47606 Da, observed mass: 47606 Da.

Hydroxylamine

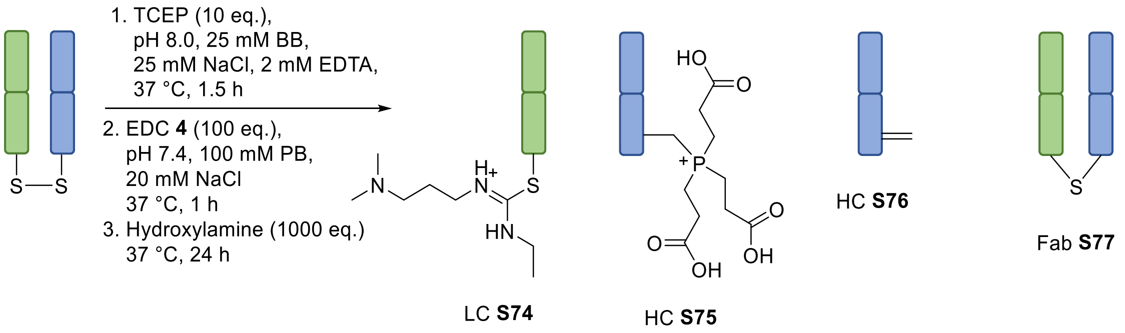

Hydroxylamine (4 µL, 500 mM in water, 1000 eq.) at 37 °C for 24 h.

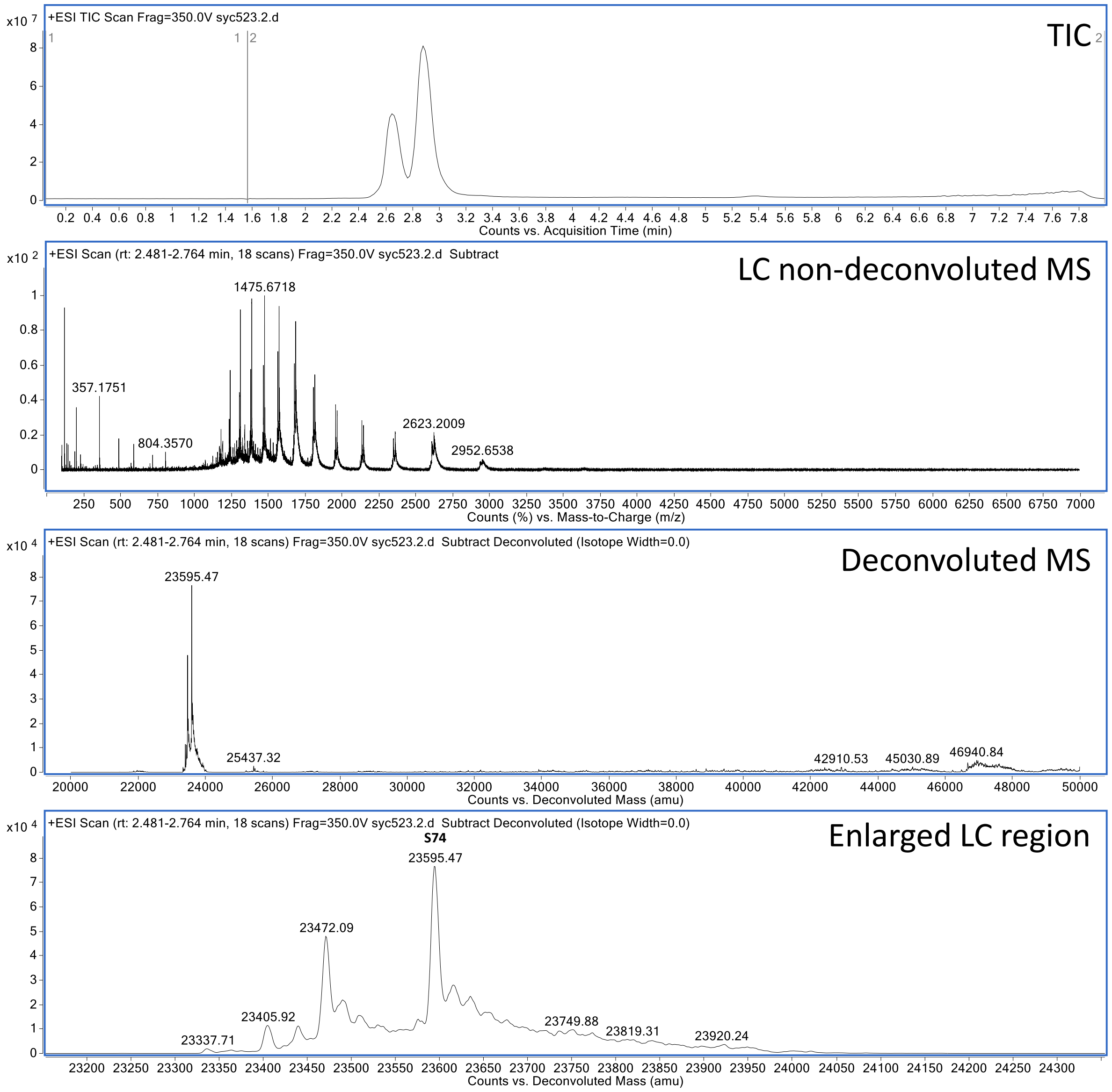

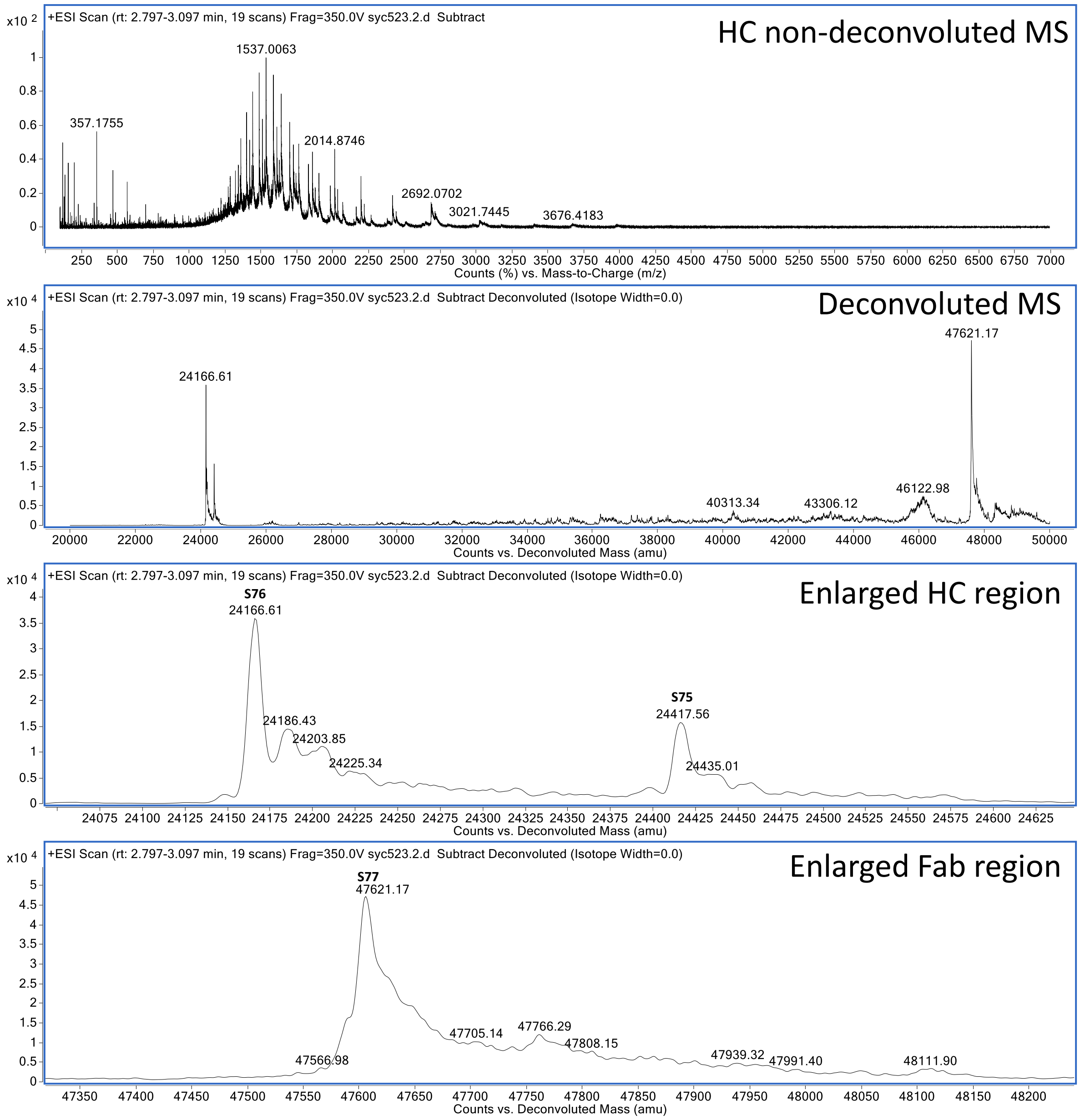

Figure S 27 LCMS of reaction of reduced Fab, EDC **4**, and hydroxylamine. **S74** expected mass: 23596 Da, observed mass: 23595 Da; **S75** expected mass: 24417 Da, observed mass: 24418 Da; **S76** expected mass: 24166 Da, observed mass: 24167 Da; **S77** expected mass: 47606 Da, observed mass: 47621 Da.

Hydrazine

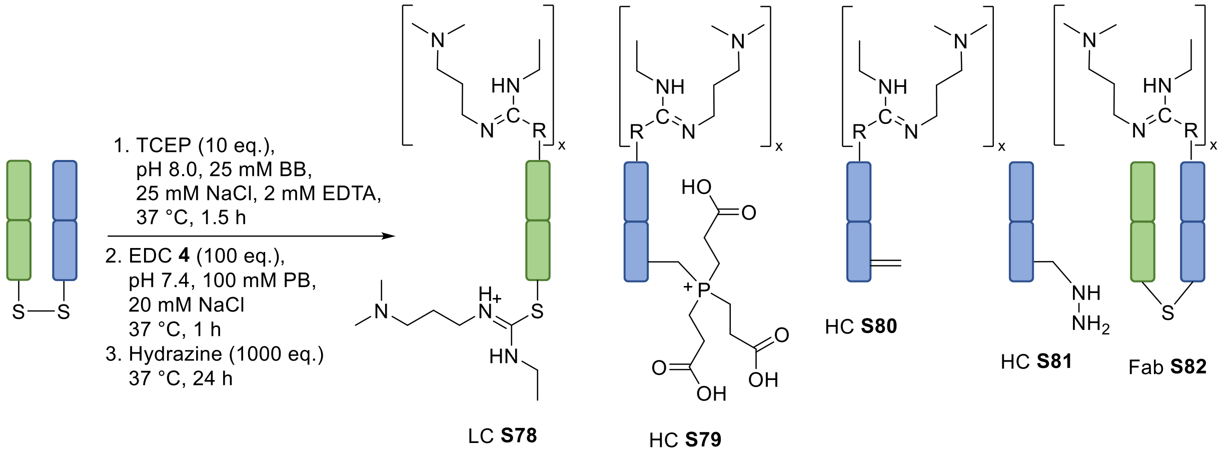

Hydrazine (4 µL, 500 mM in water, 1000 eq.) at 37 °C for 24 h.

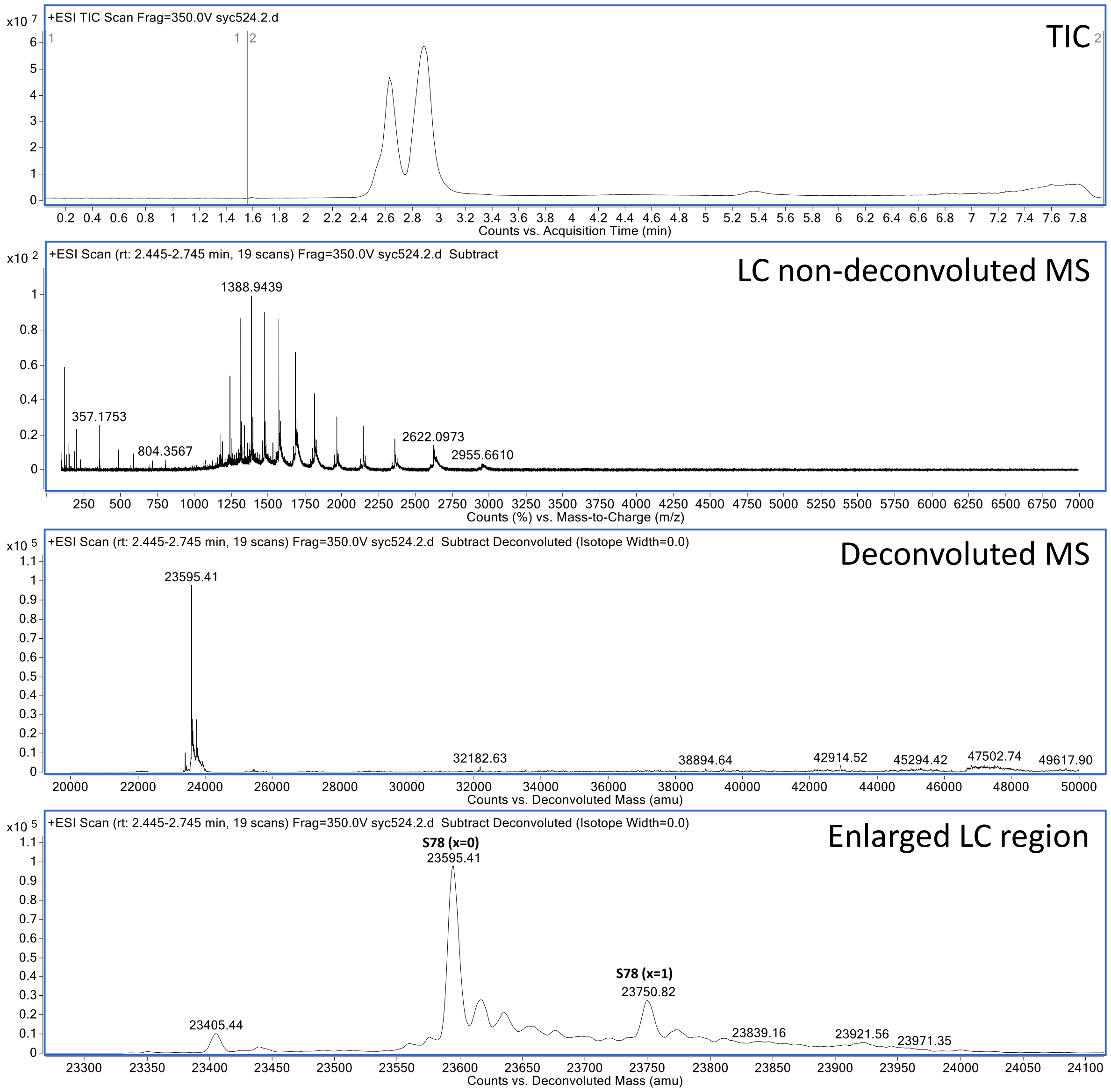

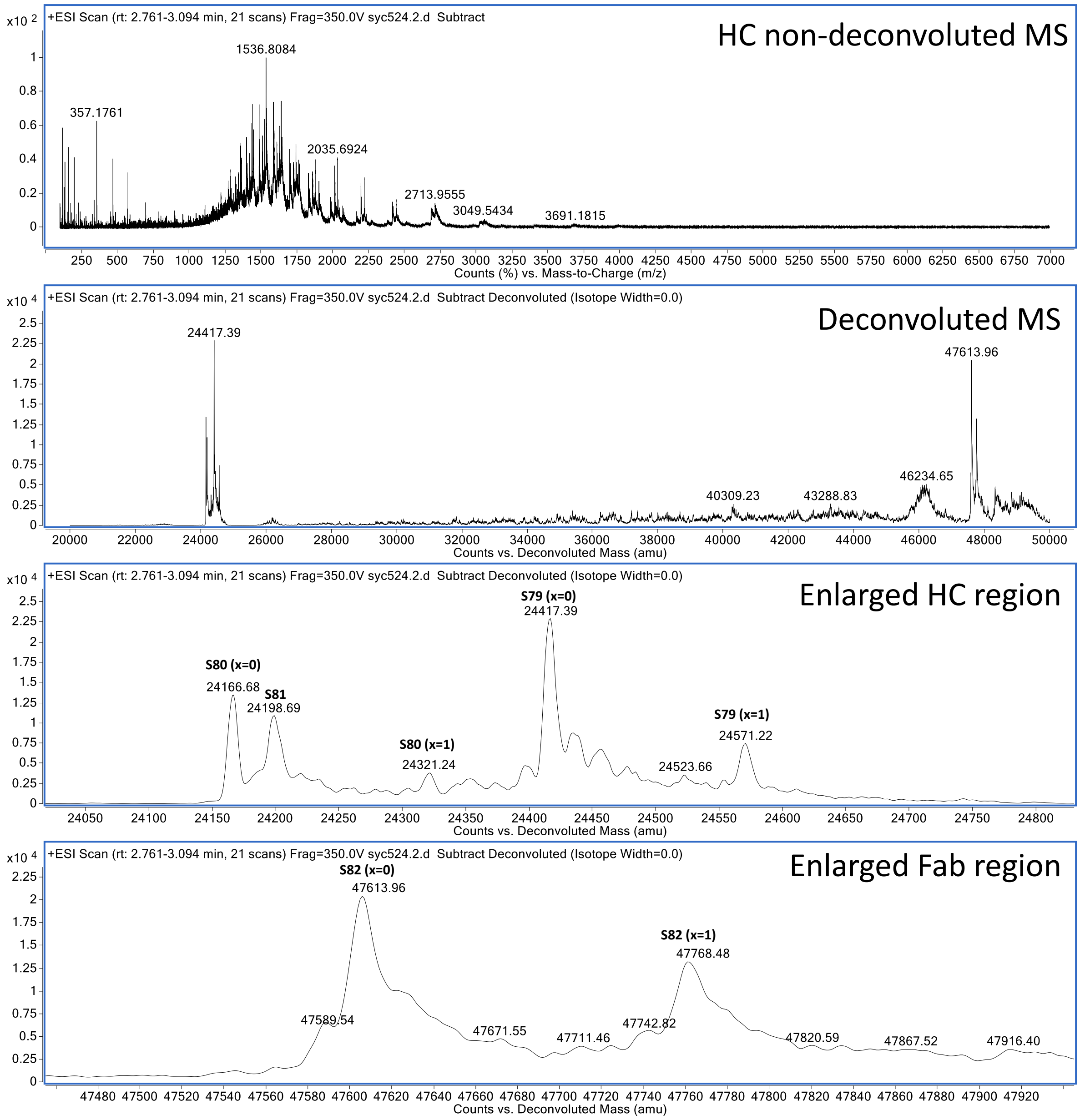

Figure S 28 LCMS of reaction of reduced Fab, EDC **4**, and hydrazine. **S78** expected mass: 23596, 23752 Da (x=0,1), observed mass: 23595, 23751 Da (x=0,1); **S79** expected mass: 24417, 24573 Da (x=0,1), observed mass: 24417, 24571 Da (x=0,1); **S80** expected mass: 24166, 24322 Da (x=0,1), observed mass: 24167, 24321 Da (x=0,1); **S81** expected mass: 24198 Da, observed mass: 24199 Da; **S82** expected mass: 47606, 46762 Da (x=0,1), observed mass: 47606, 47768Da (x=0,1).

TCEP

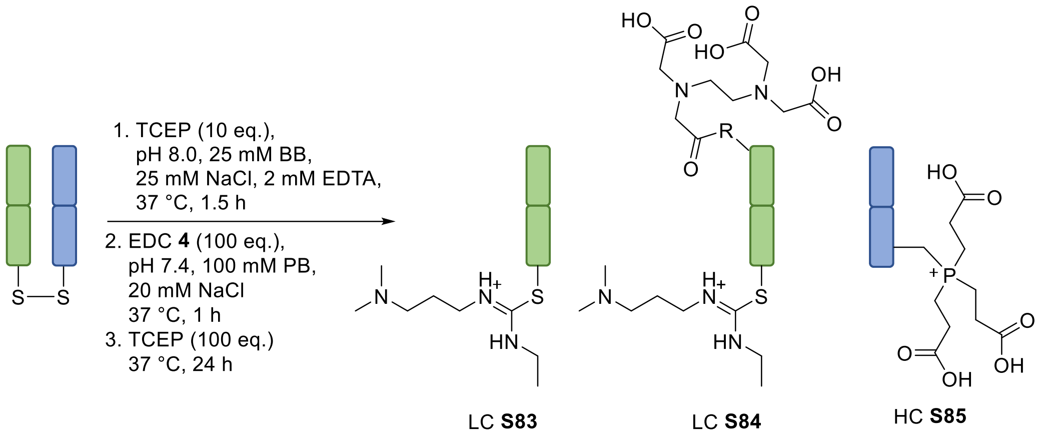

TCEP (10 µL, 20 mM in water, 100 eq.) at 37 °C for 1 h.

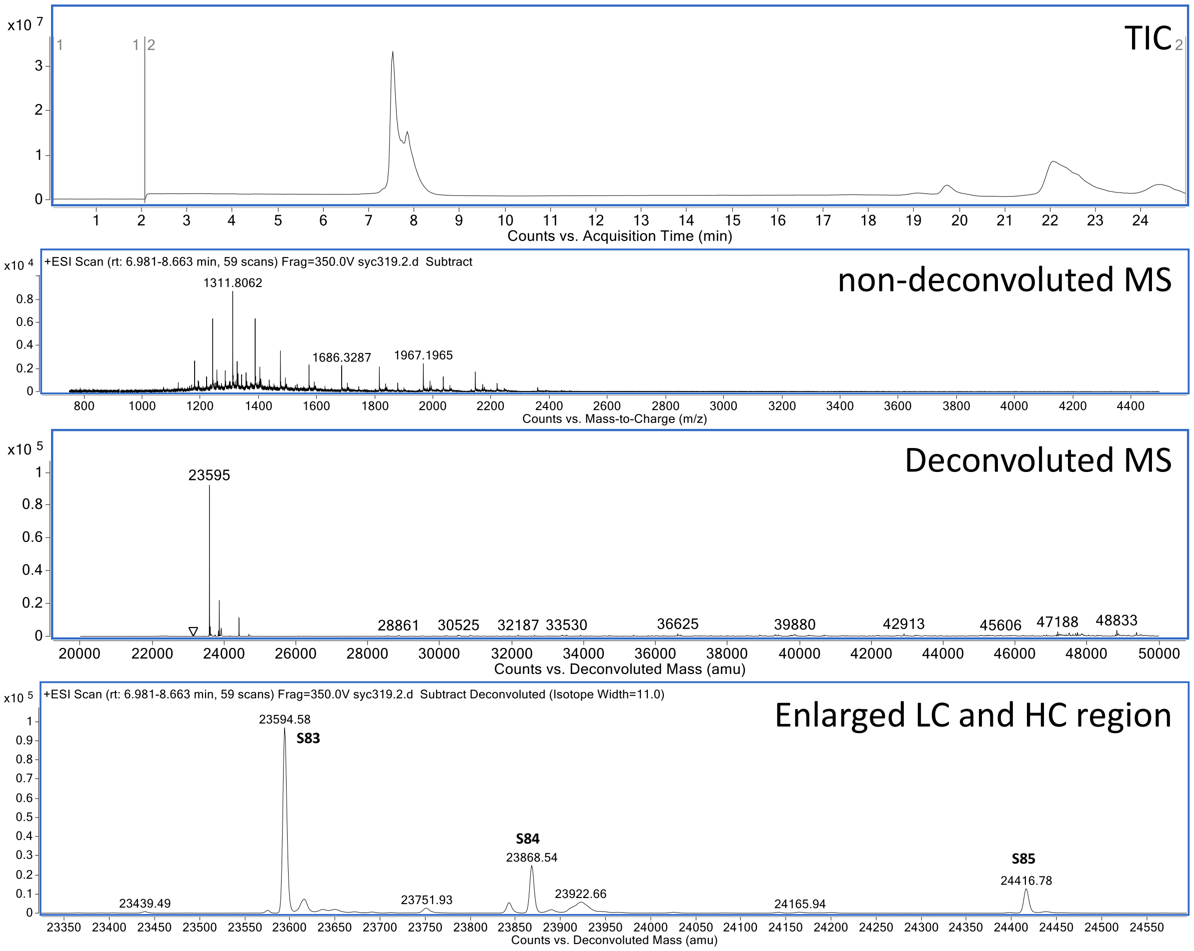

Figure S 29 LCMS of reaction of reduced Fab, EDC **4**, and TCEP. **S83** expected mass: 23596 Da, observed mass: 23595 Da; **S84** expected mass: 23870 Da, observed mass: 23869 Da; **S85** expected mass: 24417 Da, observed mass: 24417 Da; **S80** expected mass: 24166, 24322 Da (x=0,1), observed mass: 24167, 24321 Da (x=0,1); **S73** expected mass: 47606, 46762 Da (x=0,1), observed mass: 47606, 47768Da (x=0,1). Note: presence of **S84**, amide coupled EDTA is due to incomplete removal of EDTA using Zebaspin.

tris(hydroxypropyl)phosphine (THPP)

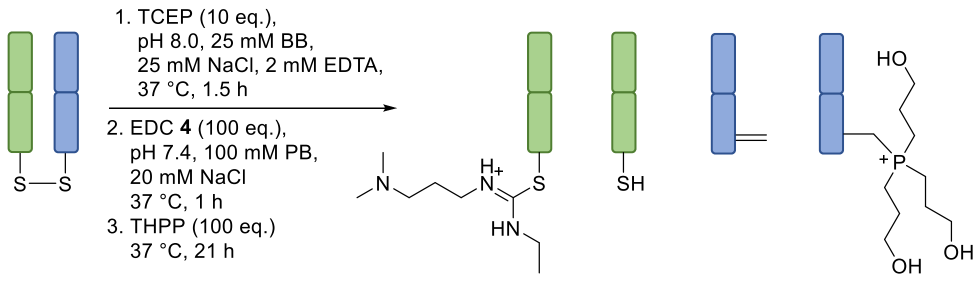

Prior to THPP addition, reaction mixture was purified (Vivaspin) into pH 8.5 (100 mM BB, 20 mM NaCl). THPP (4 µL, 50 mM in DMSO, 100 eq.) was added and reaction was incubated at 37 °C for 21 h.

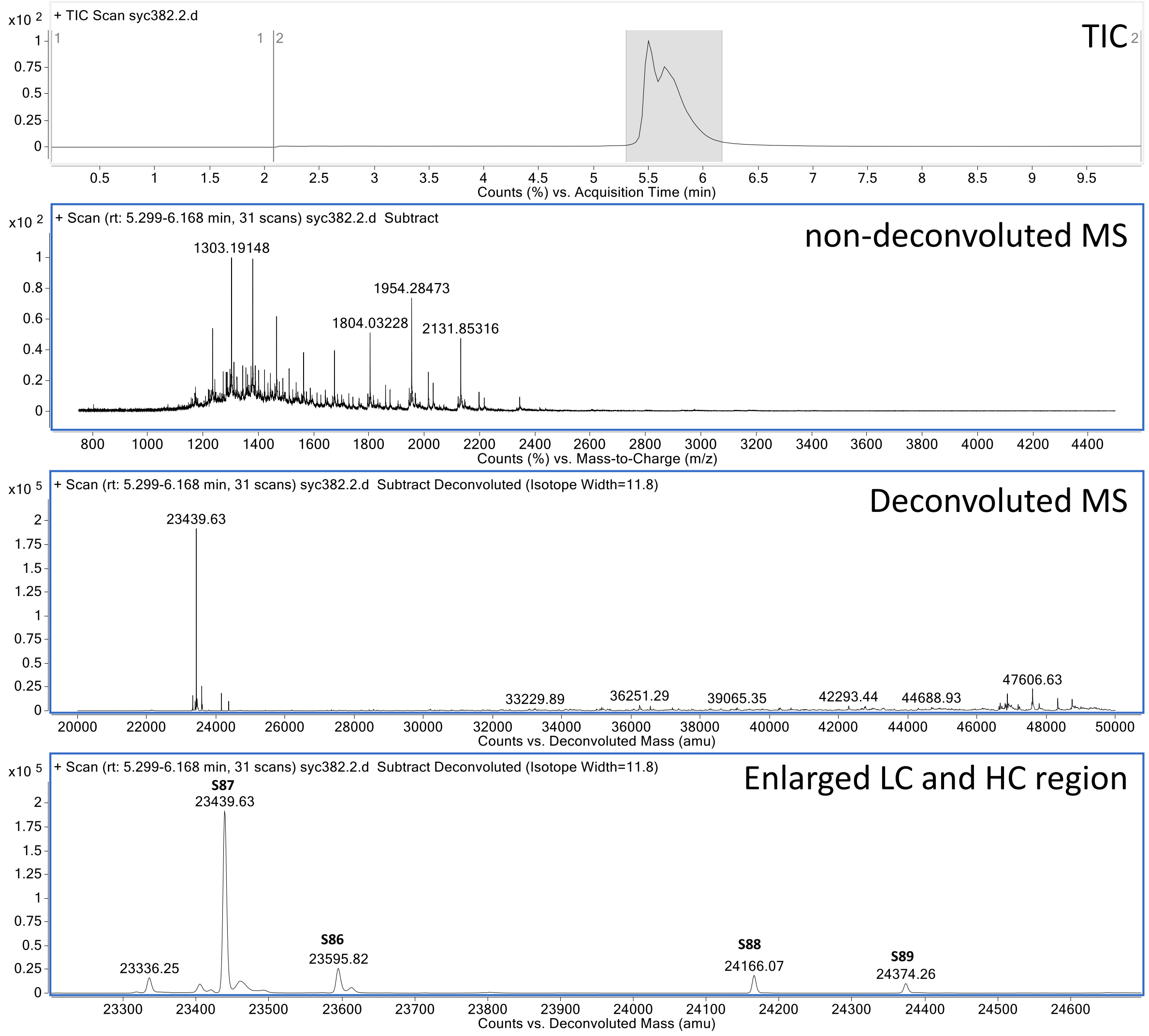

Figure S 30 LCMS of reaction of reduced Fab, EDC **4**, and TCEP. **S86** expected mass: 23596 Da, observed mass: 23596 Da; **S87** expected mass: 23440 Da, observed mass: 23440 Da; **S88** expected mass: 24166 Da, observed mass: 24166 Da; **S89** expected mass: 24375 Da, observed mass: 24374 Da.

3,3',3''-phosphanetriyltris(N-(prop-2-yn-1-yl)propanamide) S5

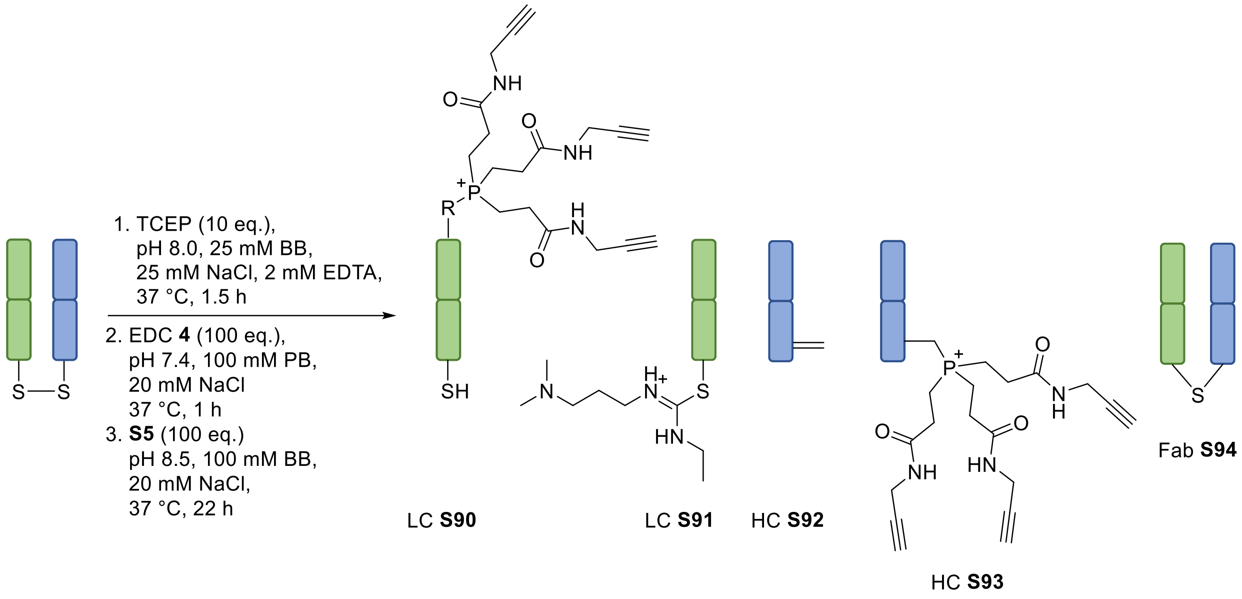

Prior to **S5** addition, reaction mixture was purified (Vivaspin) into pH 8.5 (100 mM BB, 20 mM NaCl). **S5** (4 µL, 50 mM in DMSO, 100 eq.) was added and reaction was incubated at 37 °C for 22 h.

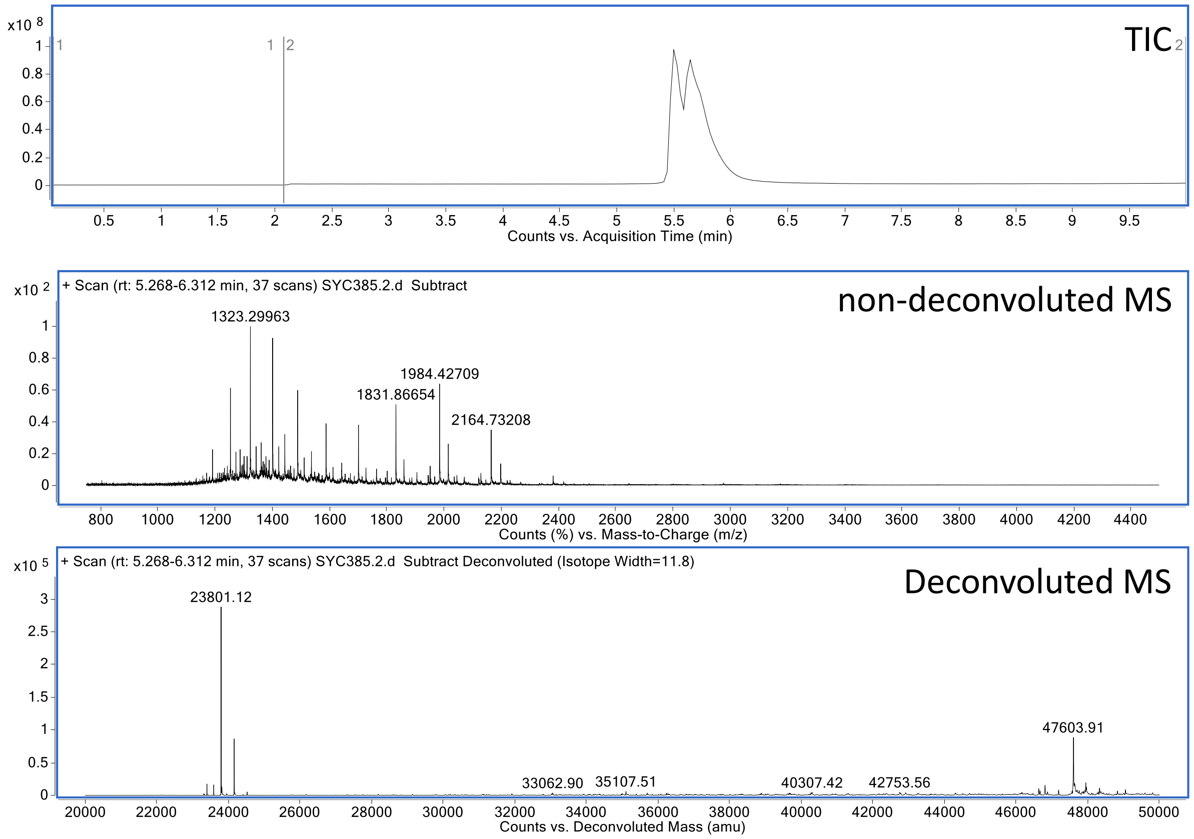

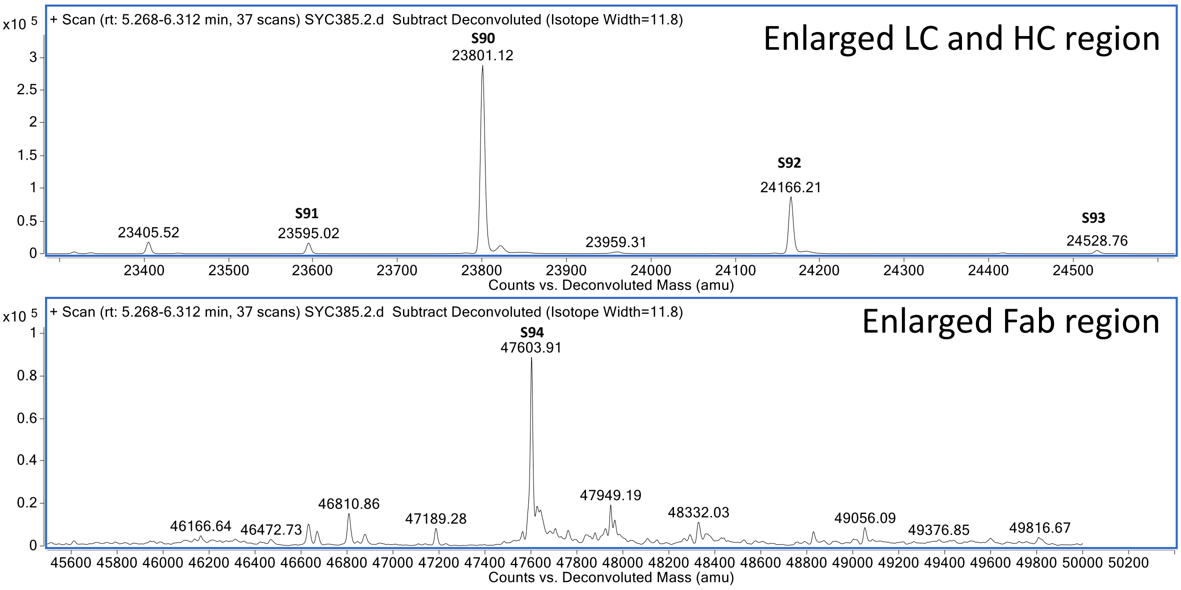

Figure S 31 LCMS of reaction of reduced Fab, EDC **4**, and MPAA. **S90** expected mass: 23800 Da, observed mass: 23801 Da; **S91** expected mass: 23596 Da, observed mass: 23595 Da; **S92** expected mass: 24166 Da, observed mass: 24166 Da; **S93** expected mass: 23528 Da, observed mass: 23529 Da. **S94** expected mass: 47606 Da, observed mass: 47604 Da. Note: site of attachment for **S90** was not determined.

Dimethyl 3,3'-((3-oxo-3-(prop-2-yn-1-ylamino)propyl)phosphanediyl)dipropionate S7

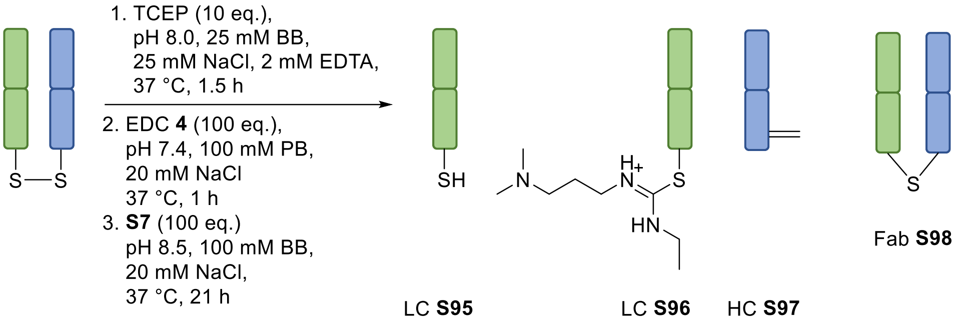

Prior to **S7** addition, reaction mixture was purified (Vivaspin) into pH 8.5 (100 mM BB, 20 mM NaCl). **S7** (8 µL, 25 mM in DMSO, 100 eq.) was added and the reaction was incubated at 37 °C for 21 h.

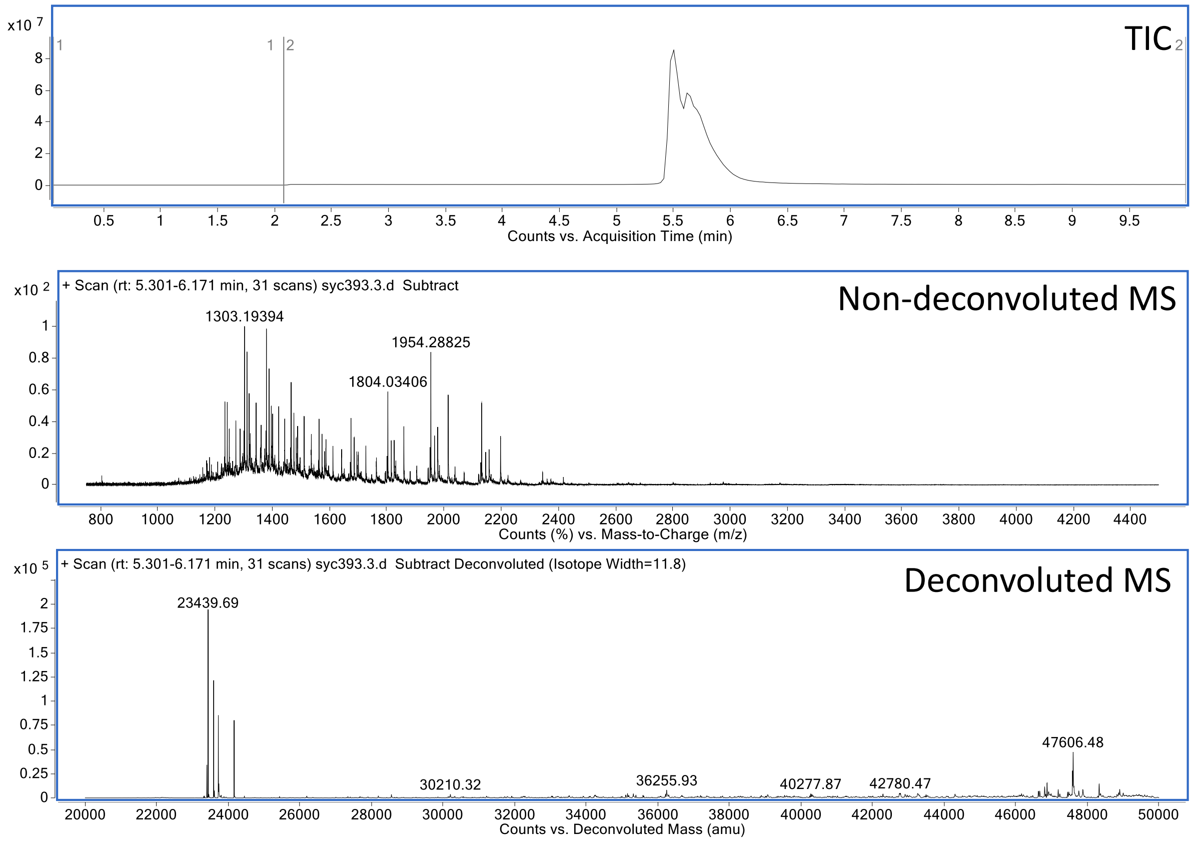

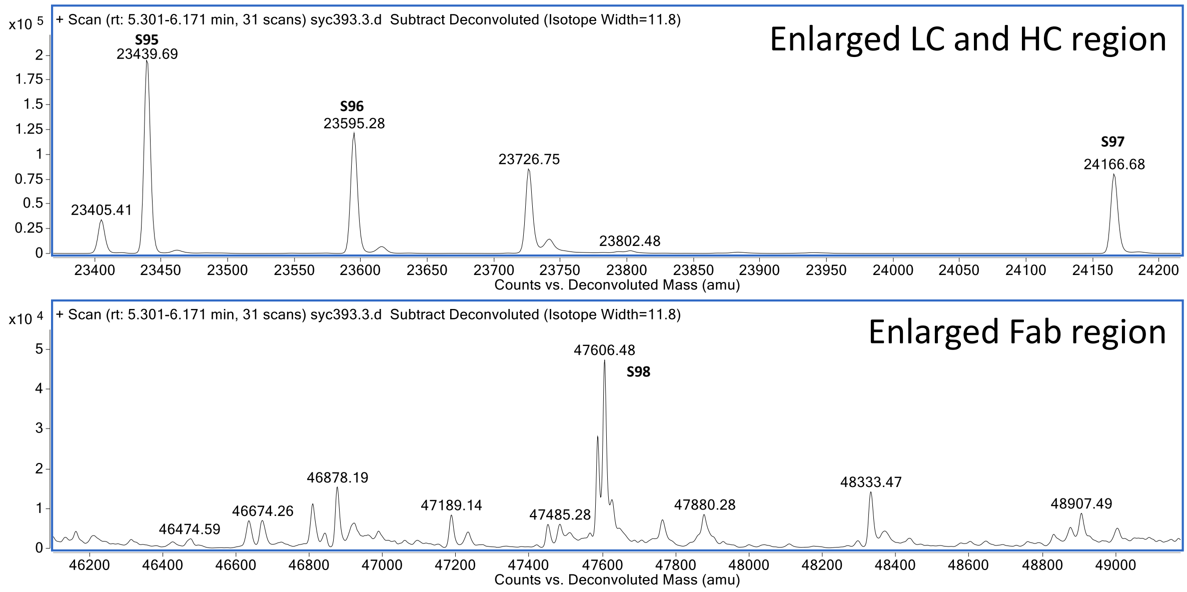

Figure S 32 LCMS of reaction of reduced Fab, EDC **4**, and MPAA. **S95** expected mass: 23440 Da, observed mass: 23440 Da; **S96** expected mass: 23596 Da, observed mass: 23595 Da; **S97** expected mass: 24166 Da, observed mass: 24167 Da; **S98** expected mass: 47606 Da, observed mass: 47604 Da.

Dually modified Fab conjugate 11

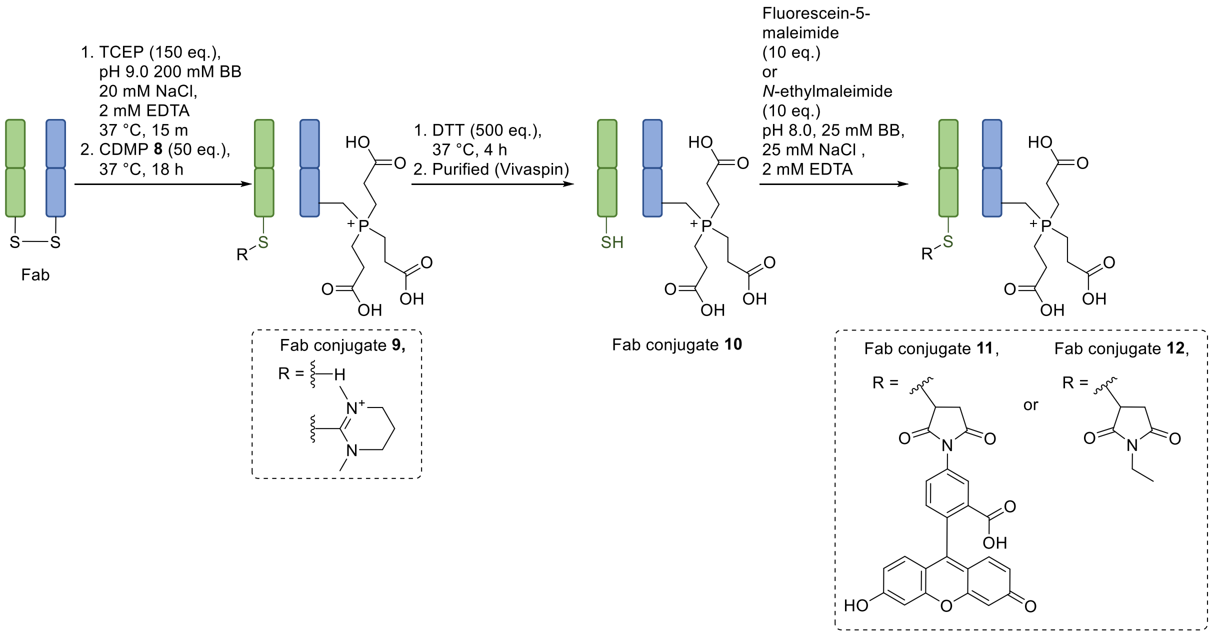

Method A: To Fab (80 µL, 20 µM) in pH 9.0 buffer (200 mM BB, 20 mM NaCl, 2 mM EDTA) was added TCEP (1.6 µL, 150 mM in H_2O, 150 eq.) and incubated at 37 °C for 15 min. To the reaction mixture was added CDMP **8** (1.6 µL, 50 mM in MeCN, 50 eq.) and incubated at 37 °C for 18 h. To the reaction mixture was added DTT (1.6 µL, 500 mM in MeCN, 500 eq.) and incubated at 37 °C for 4 h. Reaction mixture was purified (Vivaspin) into pH 8.0 buffer (25 mM BB, 25 mM NaCl, 2 mM EDTA) and was added maleimide (fluorescein-5-maleimide (1.6 µL, 10 mM in DMSO, 10 eq.) for Fab conjugate **11**, N-ethylmaleimide (3.2 µL, 5 mM in MeCN, 10 eq.) for Fab conjugate **12**) and allowed to stand at 22 °C for 15 m. Sample was desalted (ZebaSpin) and analysed using LCMS.

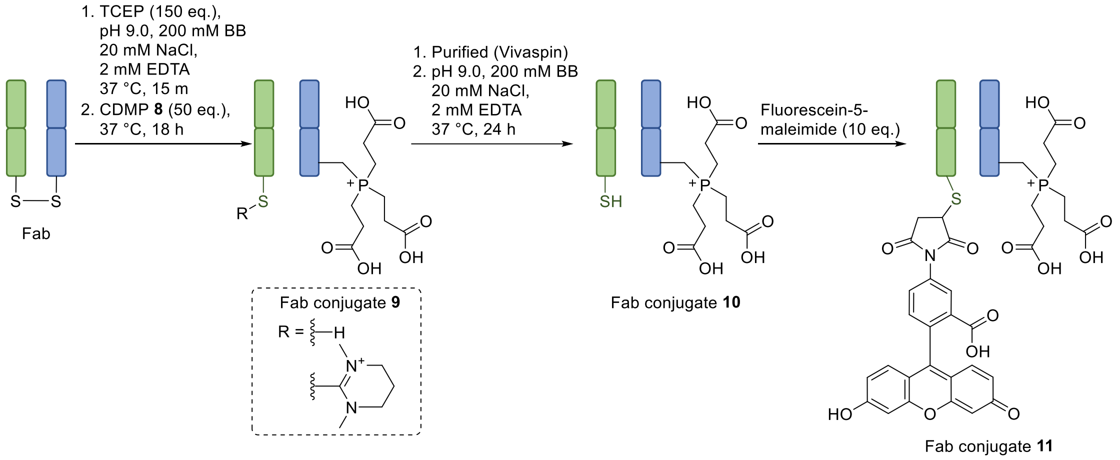

Method B: To Fab (120 µL, 20 µM) in pH 9.0 buffer (200 mM BB, 20 mM NaCl, 2 mM EDTA) was added TCEP (2.4 µL, 150 mM in H_2O, 150 eq.) and incubated at 37 °C for 15 min. To the reaction mixture was added CDMP **8** (2.4 µL, 50 mM in MeCN, 50 eq.) and incubated at 37 °C for 18 h. Reaction mixture was purified (Vivaspin) into pH 9.0 buffer (200 mM BB, 20 mM NaCl, 2 mM EDTA) and incubated at 37 °C for 24 h. To the reaction mixture was added fluorescein-5-maleimide (2.4 µL, 10 mM in DMSO, 10 eq.) and allowed to stand at 22 °C for 15 min, to generate dually modified Fab conjugate **11**. Sample was desalted (ZebaSpin) and analysed using LCMS.

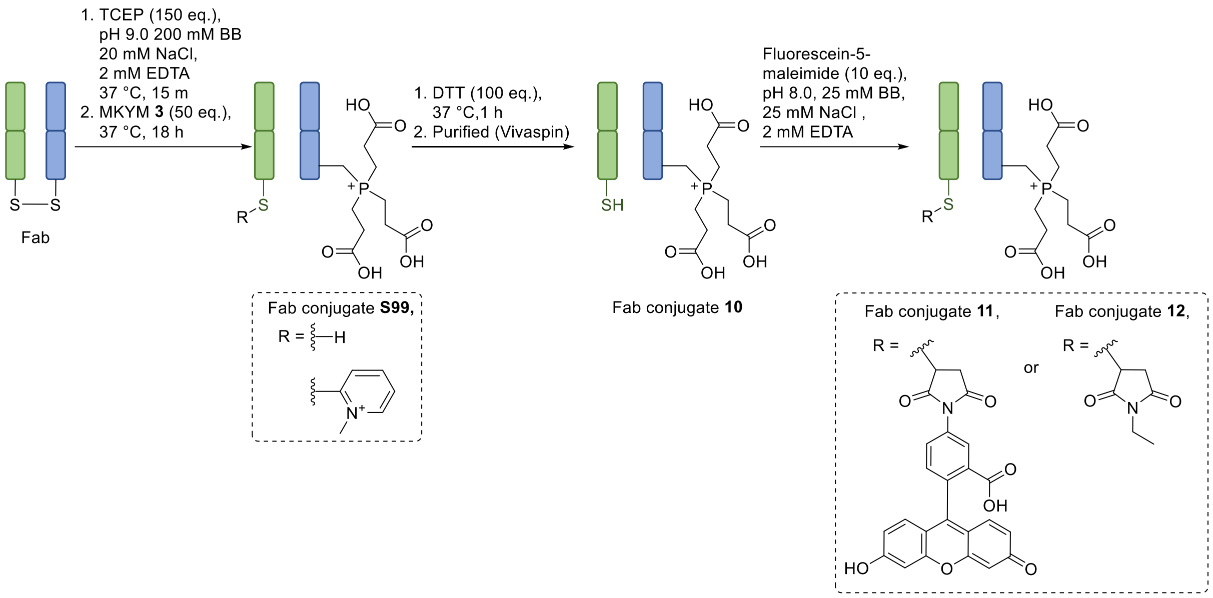

Method C: Fab (100 µL, 20 µM) in pH 9.0 buffer (200 mM BB, 20 mM NaCl, 2 mM EDTA) was added TCEP (2 µL, 150 mM in H_2O, 150 eq.) and incubated at 37 °C for 15 min. To the reaction mixture was added MKYM **3** (2 µL, 20 mM in MeCN, 20 eq.) and incubated at 37 °C for 18 h. To the reaction mixture was added DTT (2 µL, 100 mM in MeCN, 100 eq.) and incubated at 37 °C for 1 h. Reaction mixture was purified (Vivaspin) into pH 8.0 buffer (25 mM BB, 25 mM NaCl, 2 mM EDTA) and was added fluorescein-5-maleimide (2 µL, 10 mM in DMSO, 10 eq.) and allowed to stand at 22 °C for 15 min, to generate dually modified Fab conjugate **11**. Sample was desalted (ZebaSpin) and analysed using LCMS.

SDS-PAGE Fab conjugate 11

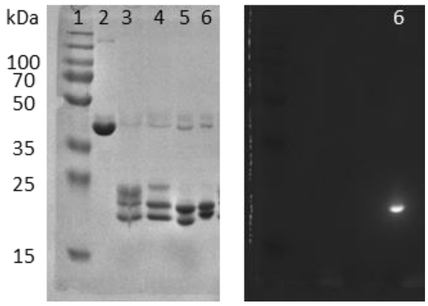

Figure S 33 SDS-PAGE of dually modified Fab conjugate **11** using method A. 1 = protein ladder, 2 = Fab **S9**, 3 = Reduced Fab **S10 S11**, 4 = Fab conjugate **9**, 5 = Fab conjugate **10**, 6 = Fab conjugate **11**.

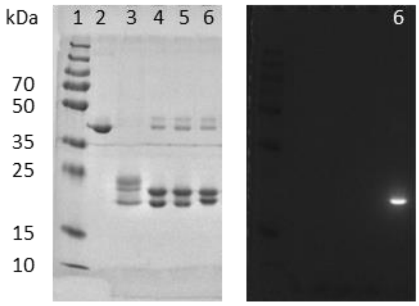

Figure S 34 SDS-PAGE of dually modified Fab conjugate **11** using method B. 1 = protein ladder, 2 = Fab **S9**, 3 = Reduced Fab **S10 S11**, 4 = Fab conjugate **9**, 5 = Fab conjugate **10**, 6 = Fab conjugate **11**.

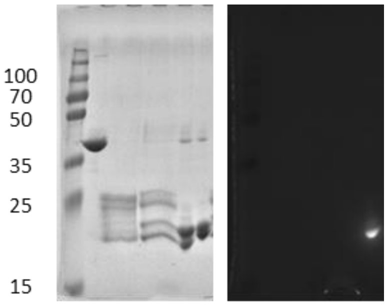

Figure S 35 SDS-PAGE of dually modified Fab conjugate **11** using method C. 1 = protein ladder, 2 = Fab **S9**, 3 = Reduced Fab **S10 S11**, 4 = Fab conjugate **S99**, 5 = Fab conjugate **10**, 6 = Fab conjugate **11**. Note: broadening of lane 3 and 4 due to high salt concentration from reaction buffer, i.e. 200 mM BB, 20 mM NaCl, 2 mM EDTA. This phenomenon was not observed in method A (Figure S 33) and method B (Figure S 34) as gel samples were desalted.

LCMS Fab conjugate 9

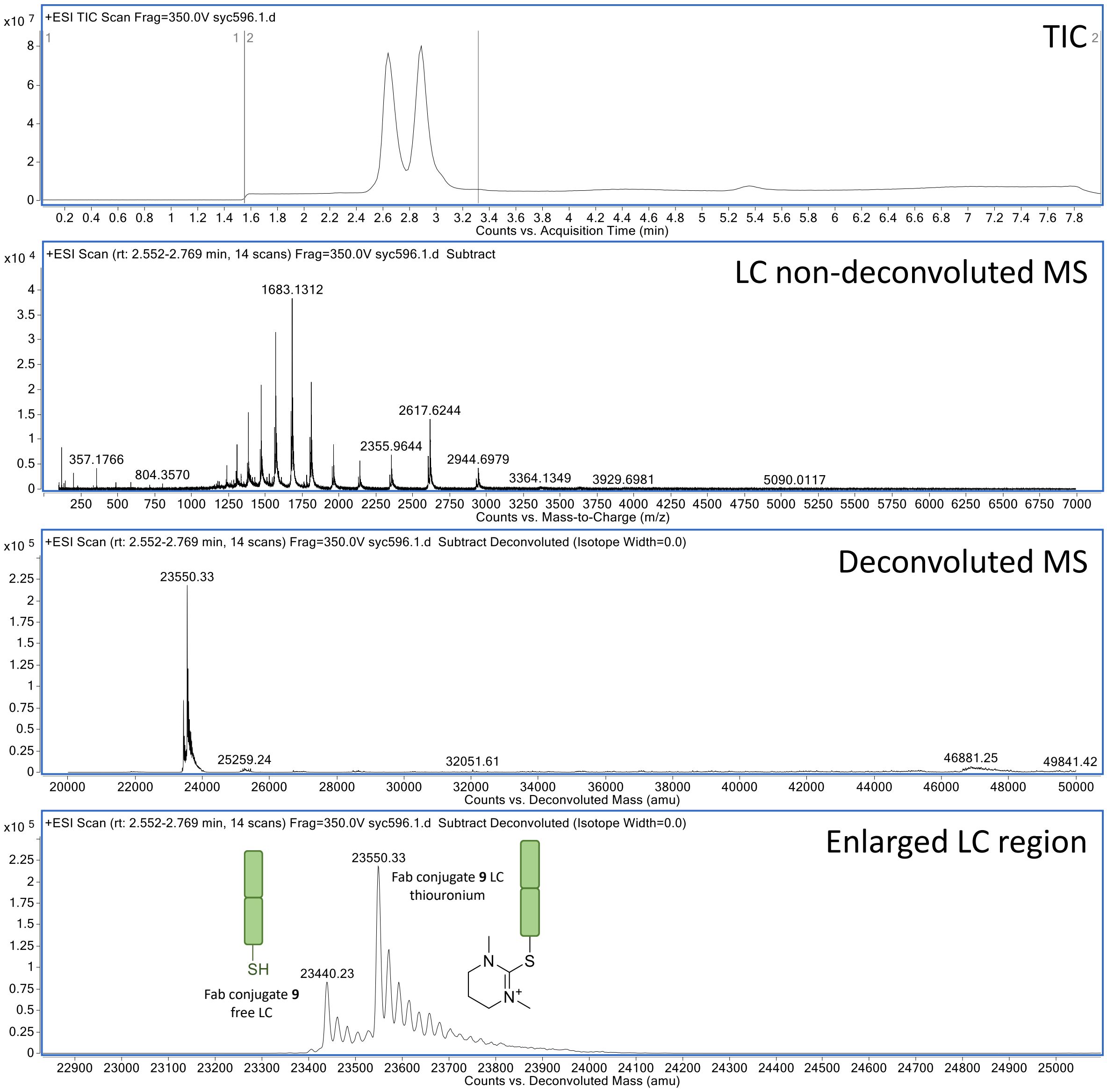

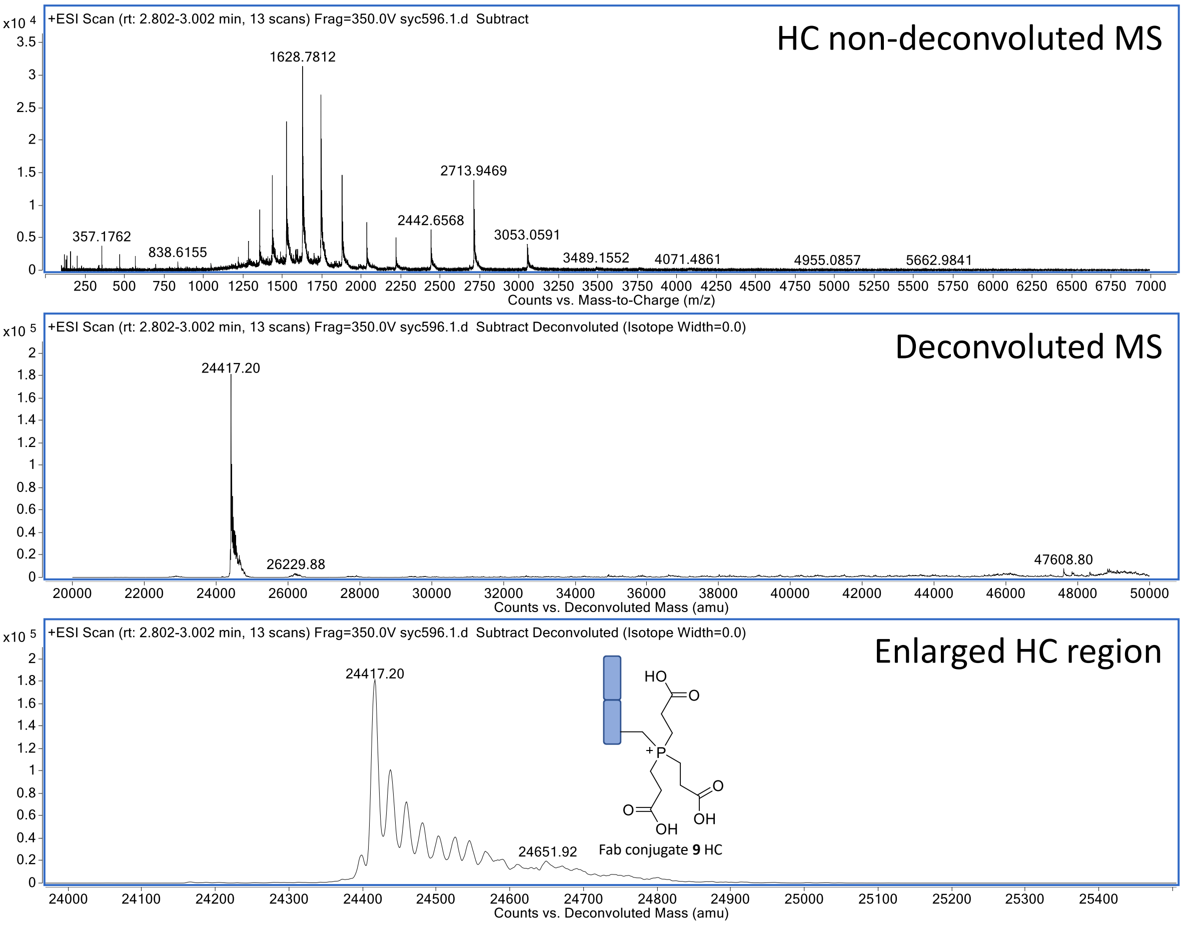

Figure S 36 LCMS Fab conjugate **9**. Fab conjugate **9** free LC, expected mass: 23440 Da, observed mass: 23440 Da; Fab conjugate **9** LC thiouronium, expected mass: 23551 Da, observed mass: 23550 Da; Fab conjugate **9** HC, expected mass: 24417 Da, observed mass: 24417 Da.

LCMS Fab conjugate S99

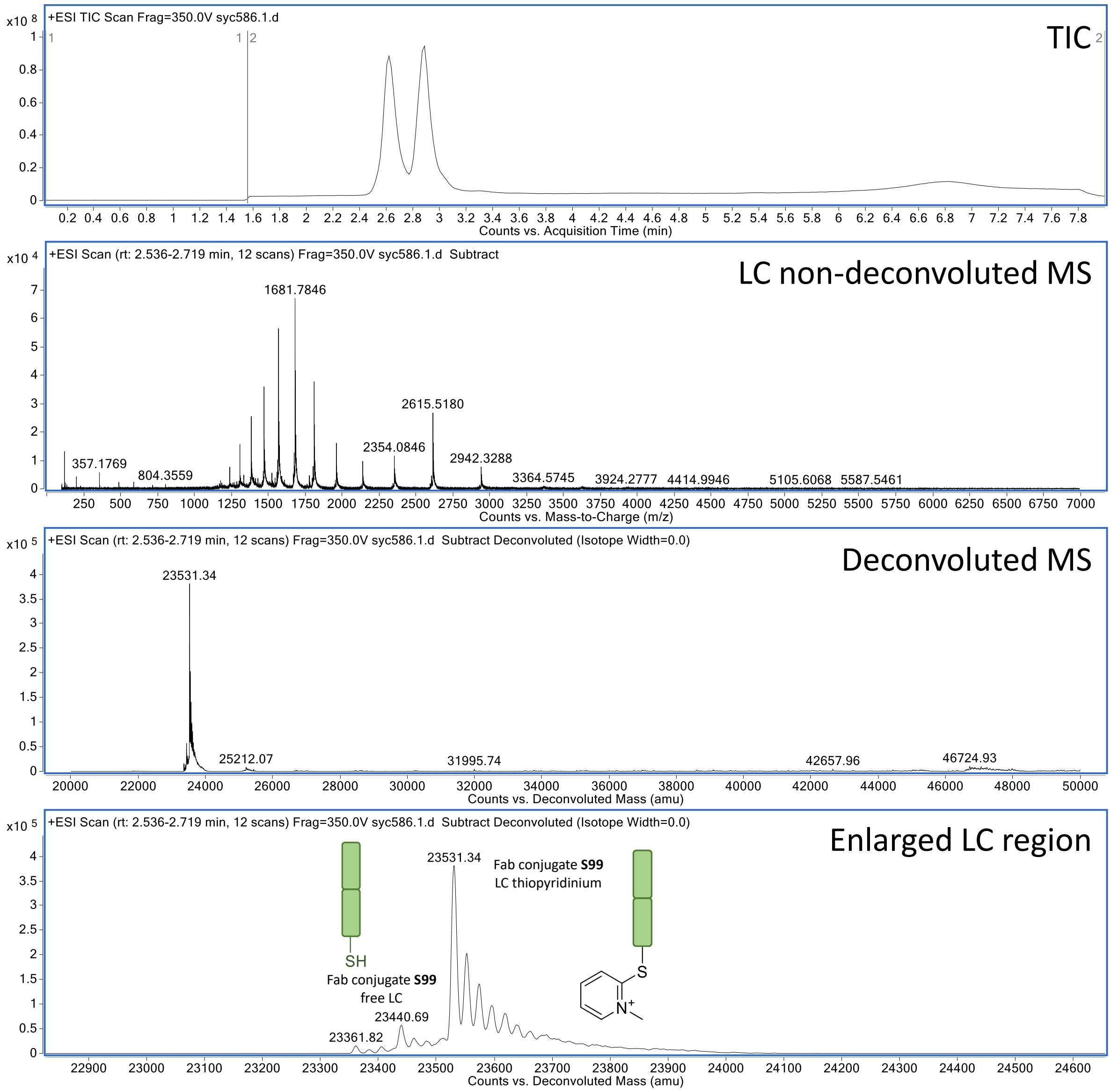

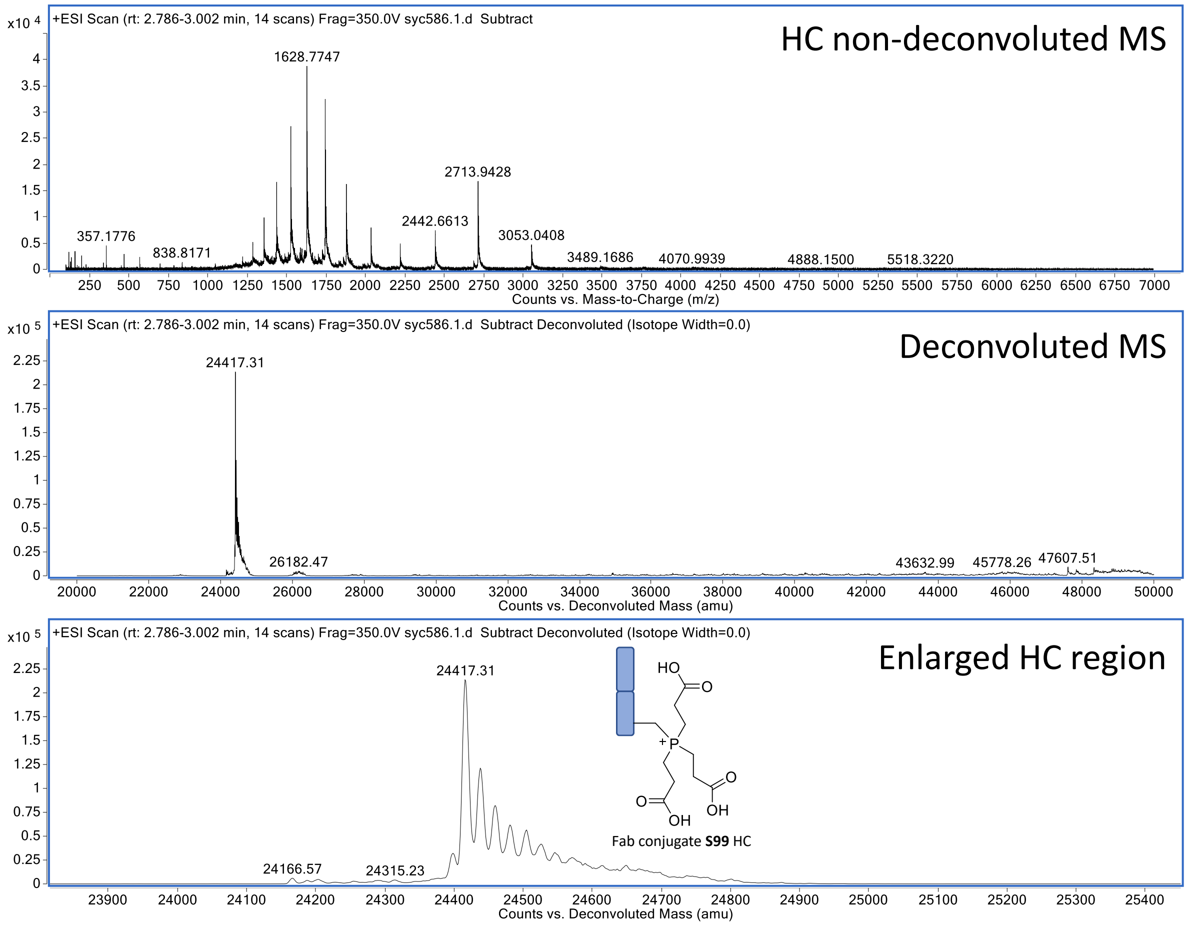

Figure S 37 LCMS Fab conjugate **S99**. Fab conjugate **S99** free LC, expected mass: 23440 Da, observed mass: 23441 Da; Fab conjugate **9** LC thiopyridinium, expected mass: 23532 Da, observed mass: 23531 Da; Fab conjugate **11** HC, expected mass: 24417 Da, observed mass: 24417 Da.

LCMS Fab conjugate 11

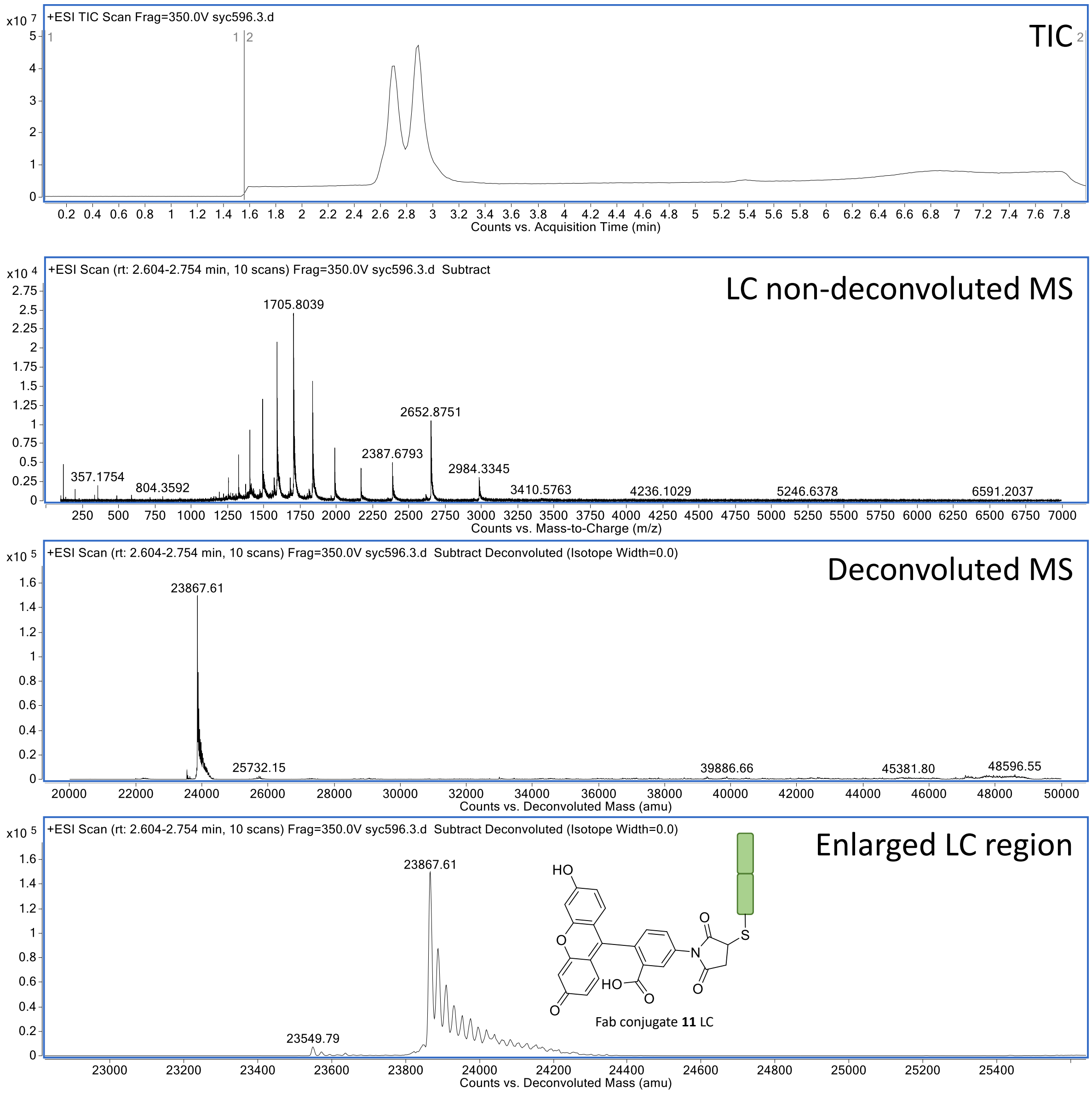

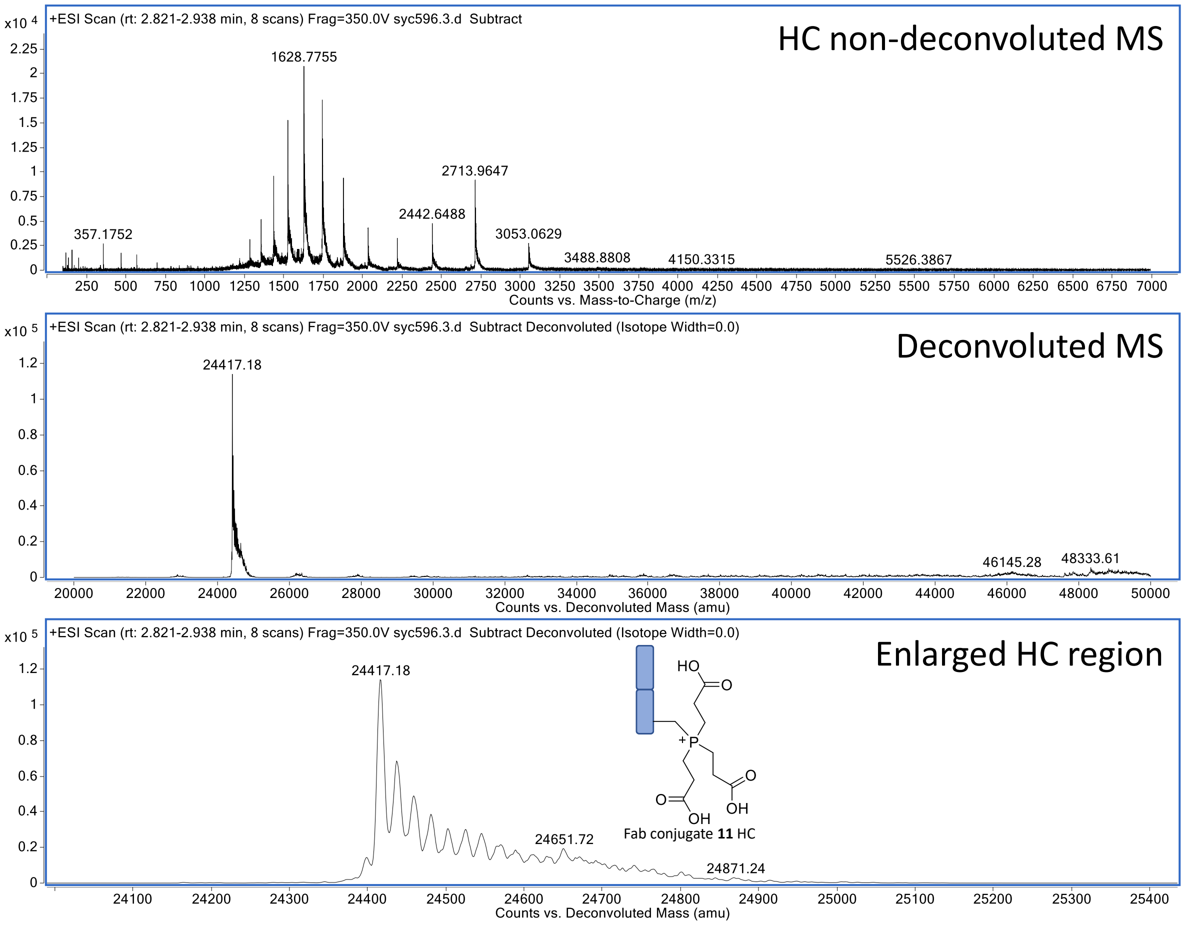

Figure S 38 LCMS Fab conjugate **11**. Fab conjugate **11** LC, expected mass: 23867 Da, observed mass: 23868 Da; Fab conjugate **11** HC, expected mass: 24417 Da, observed mass: 24417 Da.

LCMS Fab conjugate 12

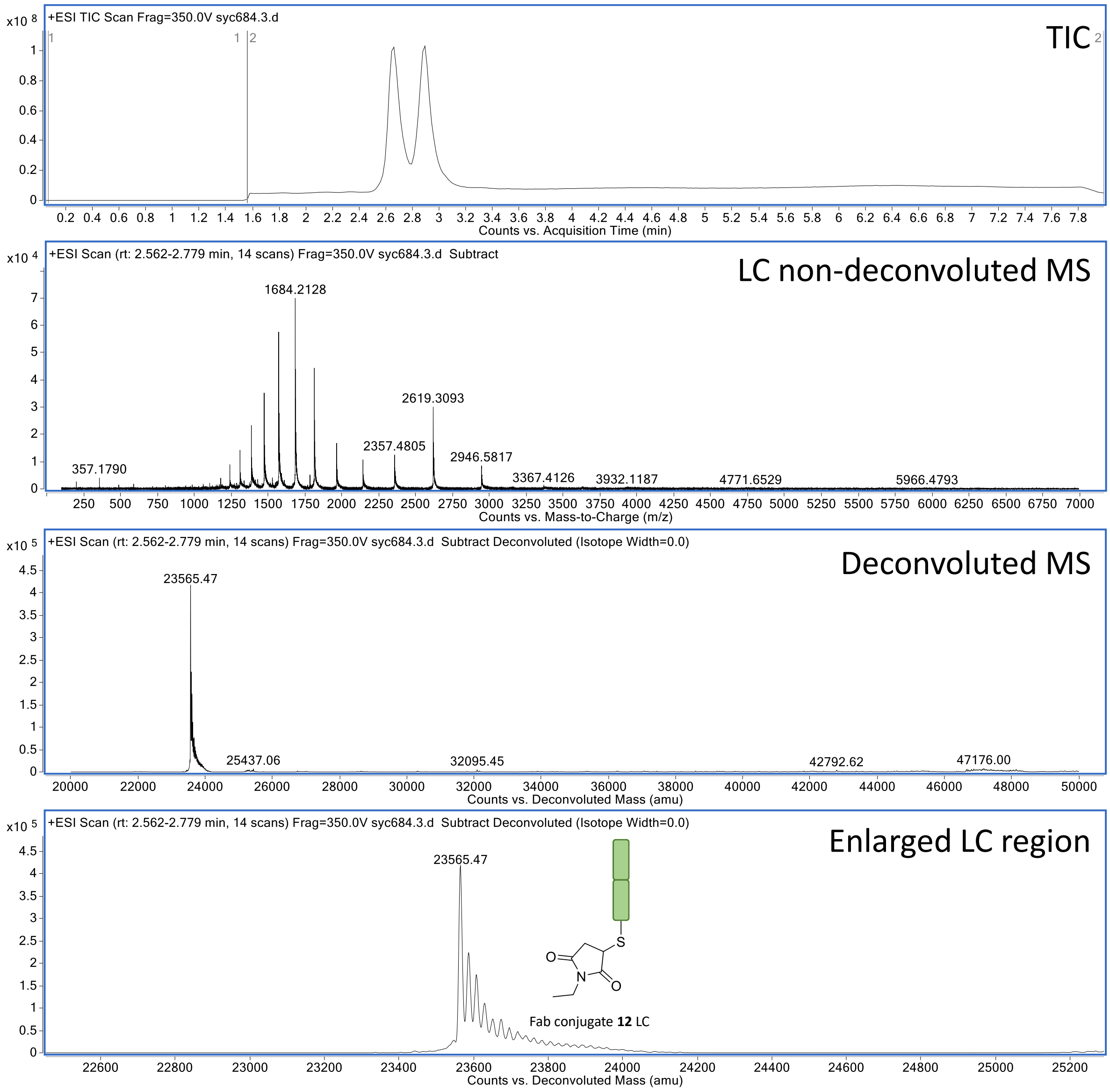

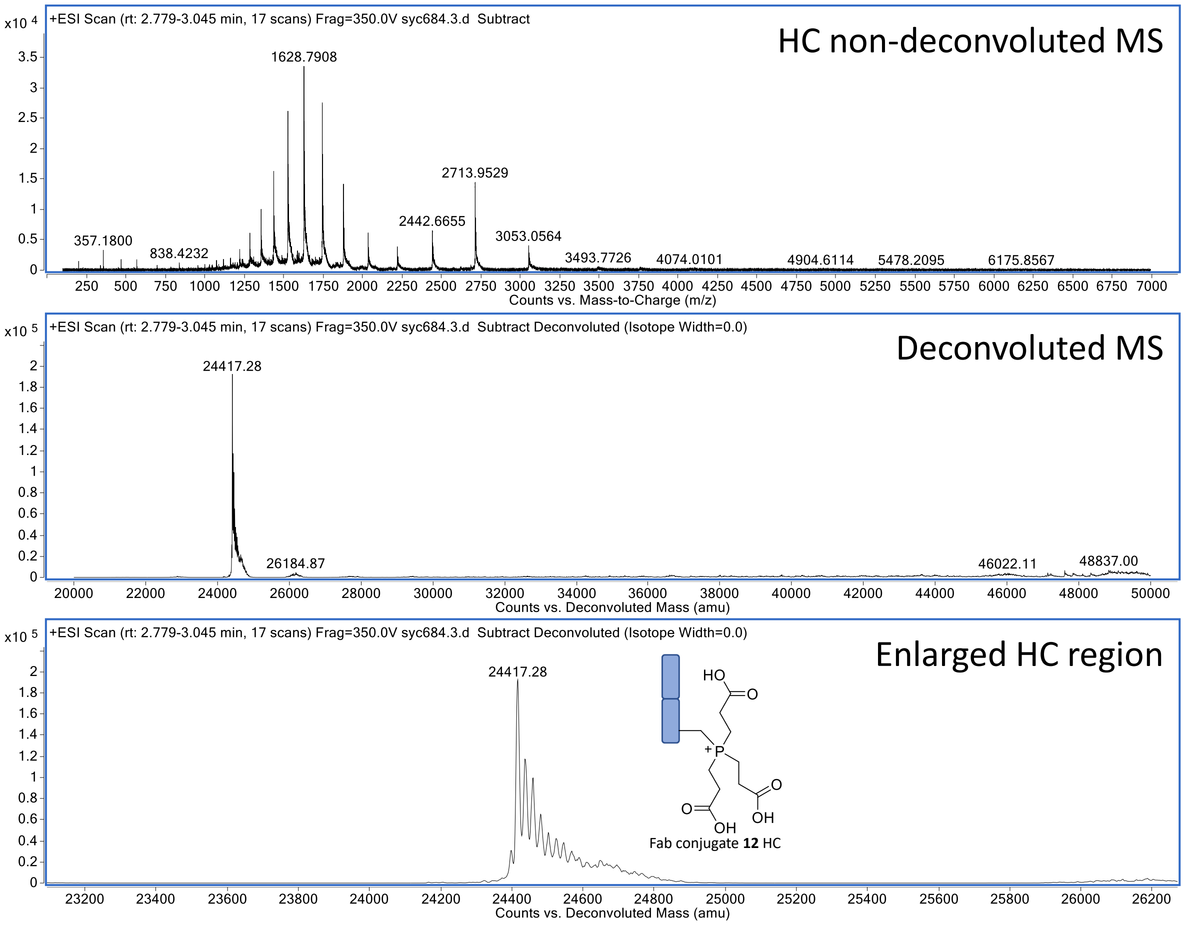

Figure S 39 LCMS Fab conjugate **12**. Fab conjugate **12** LC, expected mass: 23565 Da, observed mass: 23565 Da; Fab conjugate **12** HC, expected mass: 24417 Da, observed mass: 24417 Da.

Dually modified IgG

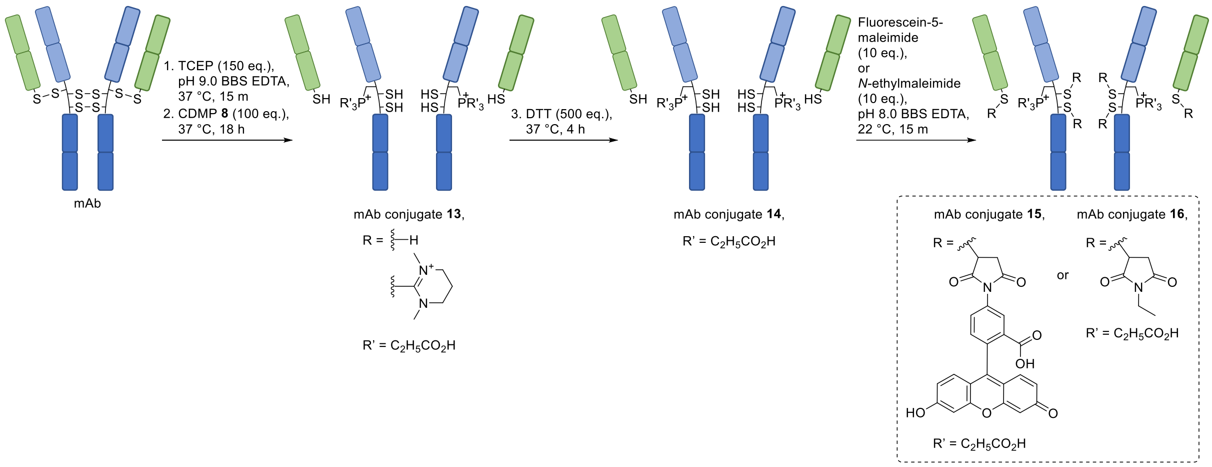

To mAb **S100** (200 µL, 20 µM) in pH 9.0 buffer (200 mM BB, 20 mM NaCl, 2 mM EDTA) was added TCEP (4 µL, 150 mM in H_2O, 150 eq.) and incubated at 37 °C for 15 m. To the reaction mixture was added CDMP **8** (4 µL, 100 mM in MeCN, 100 eq.) and incubated at 37 °C for 18 h. To the reaction mixture was added DTT (4 µL, 500 mM in MeCN, 500 eq.) and incubated at 37 °C for 4 h. Reaction mixture was purified (Vivaspin) into pH 8.0 buffer (25 mM BB, 25 mM NaCl, 2 mM EDTA) and was added (fluorescein-5-maleimide (4 µL, 10 mM in DMSO, 10 eq.) for mAb conjugate **15**, N-ethylmaleimide (8 µL, 5 mM in MeCN, 10 eq.) for mAb conjugate **16**) and allowed to stand at 22 °C for 15 min. Sample was desalted (ZebaSpin), added PNGase, and allowed to stand at 4 °C for 16 h before LCMS analysis.

SDS-PAGE mAb conjugate 15

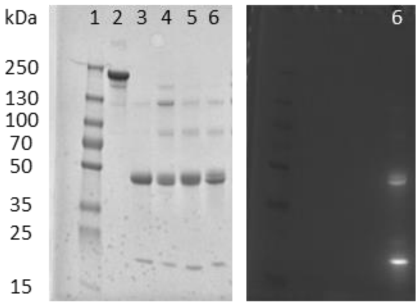

Figure S 40 SDS-PAGE of dually modified mAb conjugate **15** . 1 = protein ladder, 2 = mAb **S100**, 3 = Reduced mAb, 4 = mAb conjugate **13**, 5 = mAb conjugate **14**, 6 = Fab conjugate **15**.

LCMS Native Trastuzumab S100

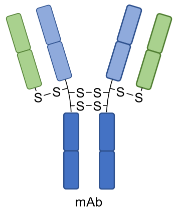

Trastuzumab **S100** was purchased from UCLH in its clinical formulation. Sample was desalted (ZebaSpin) and analysed using LCMS.

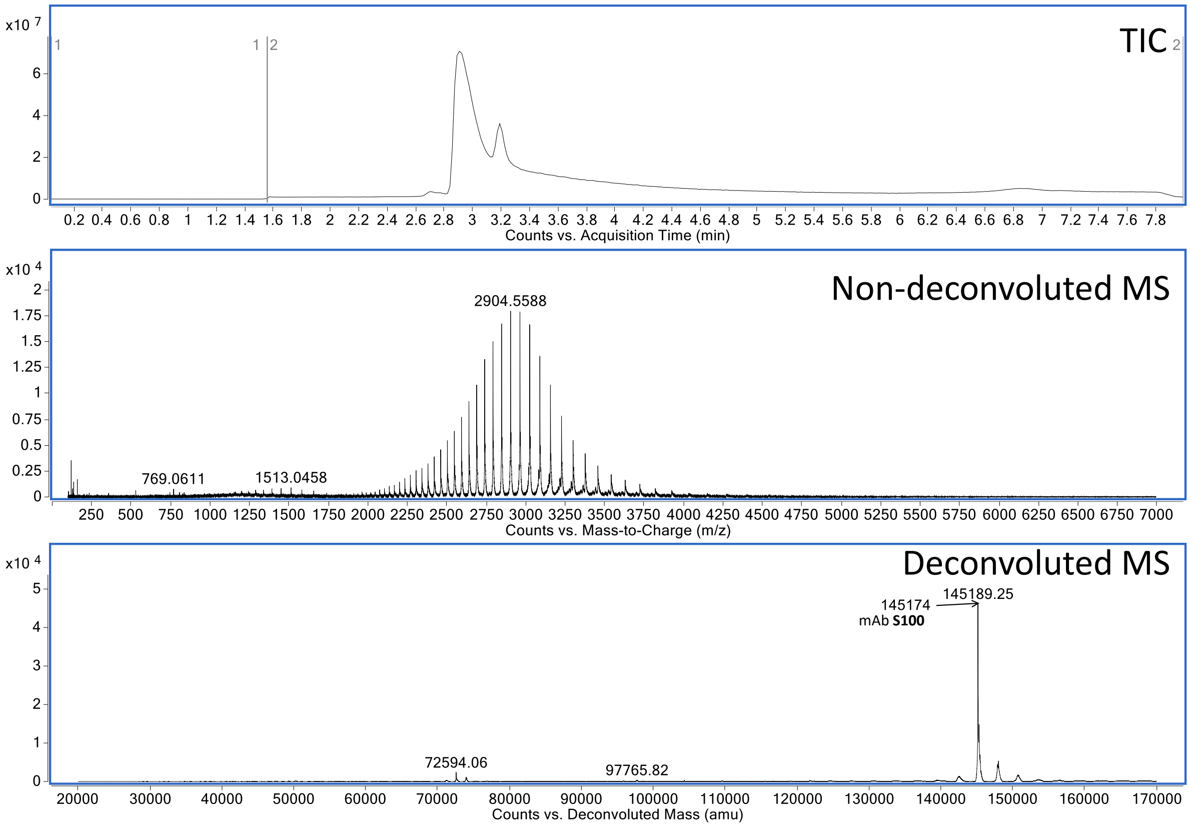

Figure S 41 LCMS of Trastuzumab. **mAb S100**, observed 145174 Da. Agilent software automatic peak picking was offset for unknown reasons. Peak maximum was subsequently manually selected as indicated by arrow.

LCMS of reduced Trastuzumab

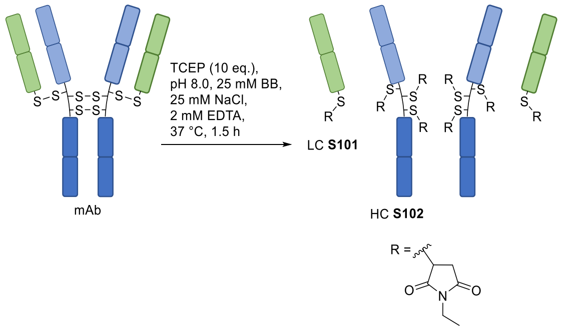

To trastuzumab **S100** (100 µL, 20 µM) in pH 8.0 buffer (25 mM BB, 25 mM NaCl, 2 mM EDTA) was added TCEP (1 µL, 20 mM in water, 10 eq.) and incubated at 37 °C for 1.5 h. Sample was desalted (ZebaSpin), added PNGase, and allowed to stand at 4°C for 16 h before LCMS analysis.

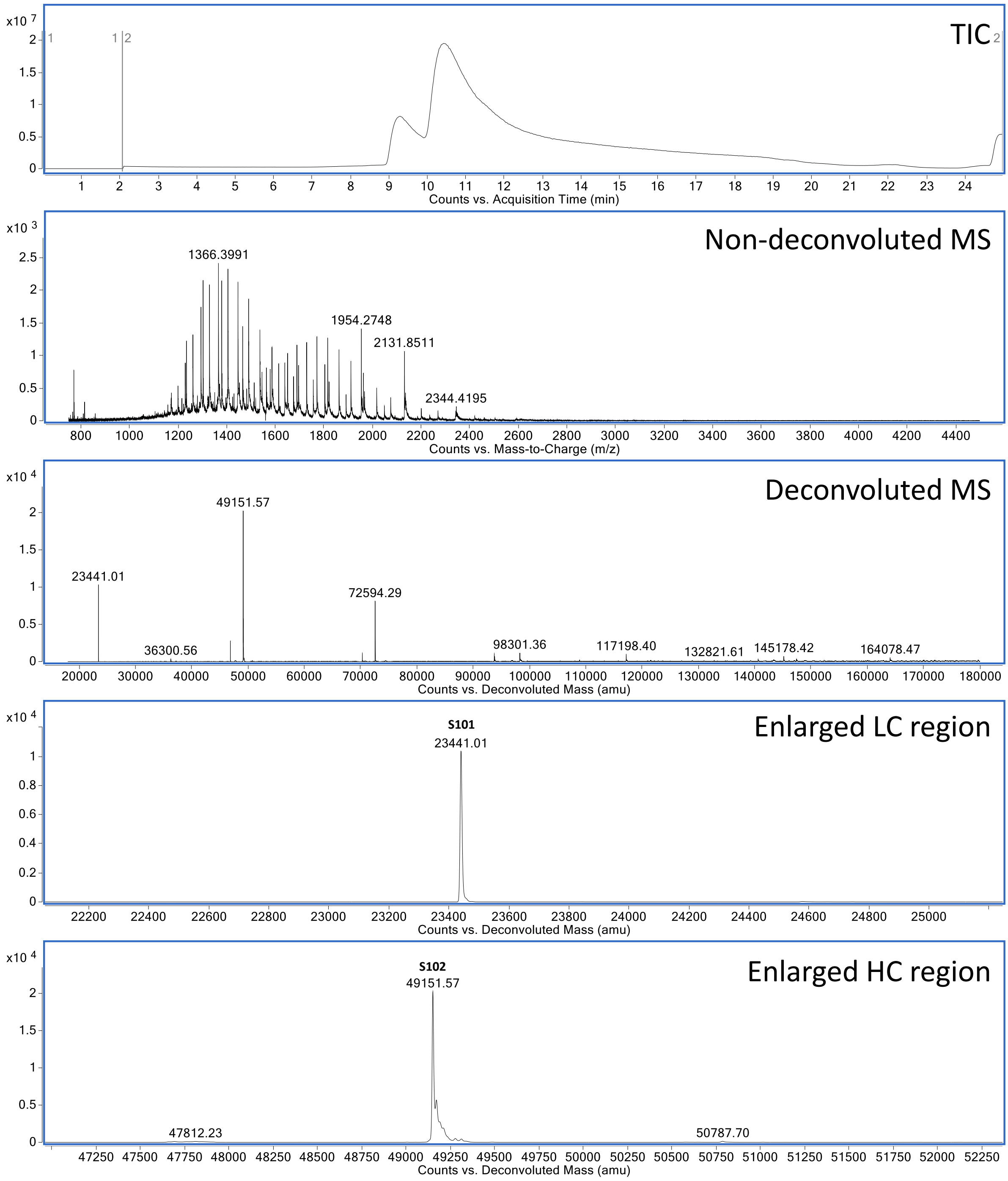

Figure S 42 LCMS of reduced Trastuzumab. **S101**, observed mass: 23441 Da; **S102**, observed mass: 49142 Da.

LCMS mAb conjugate 13

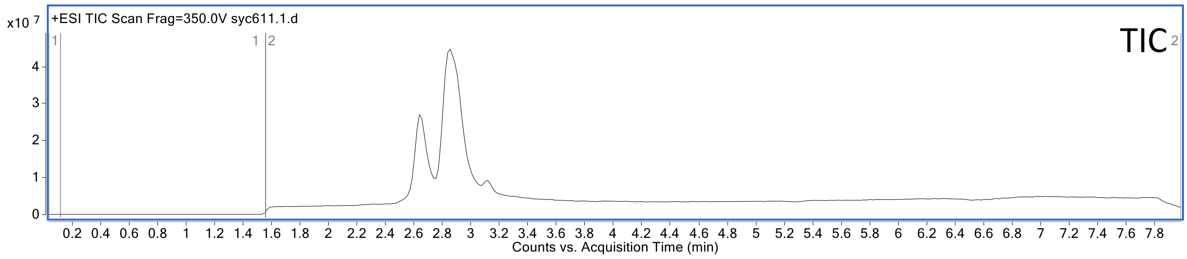

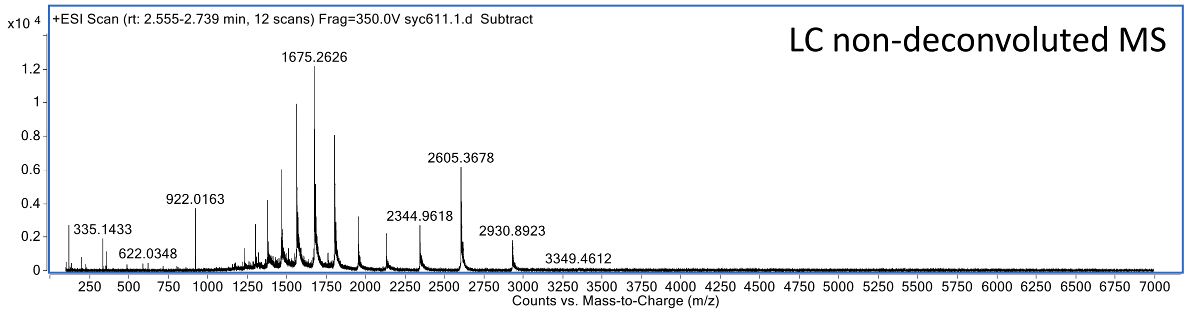

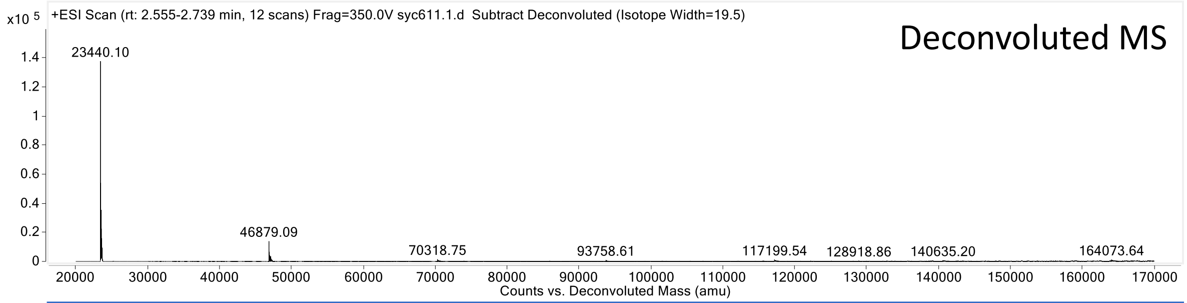

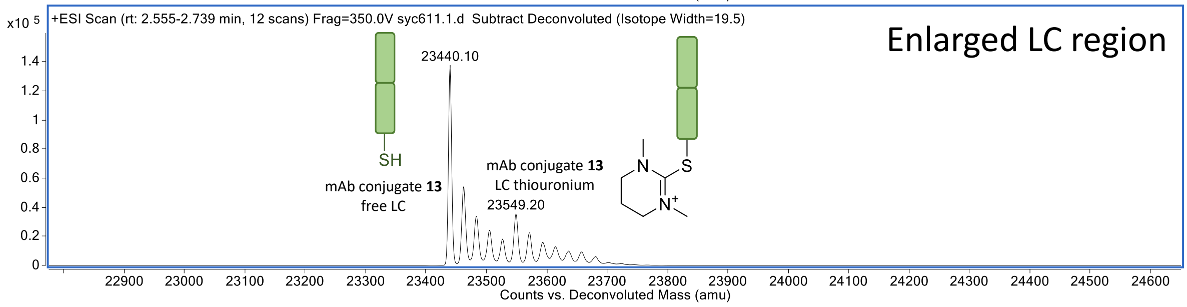

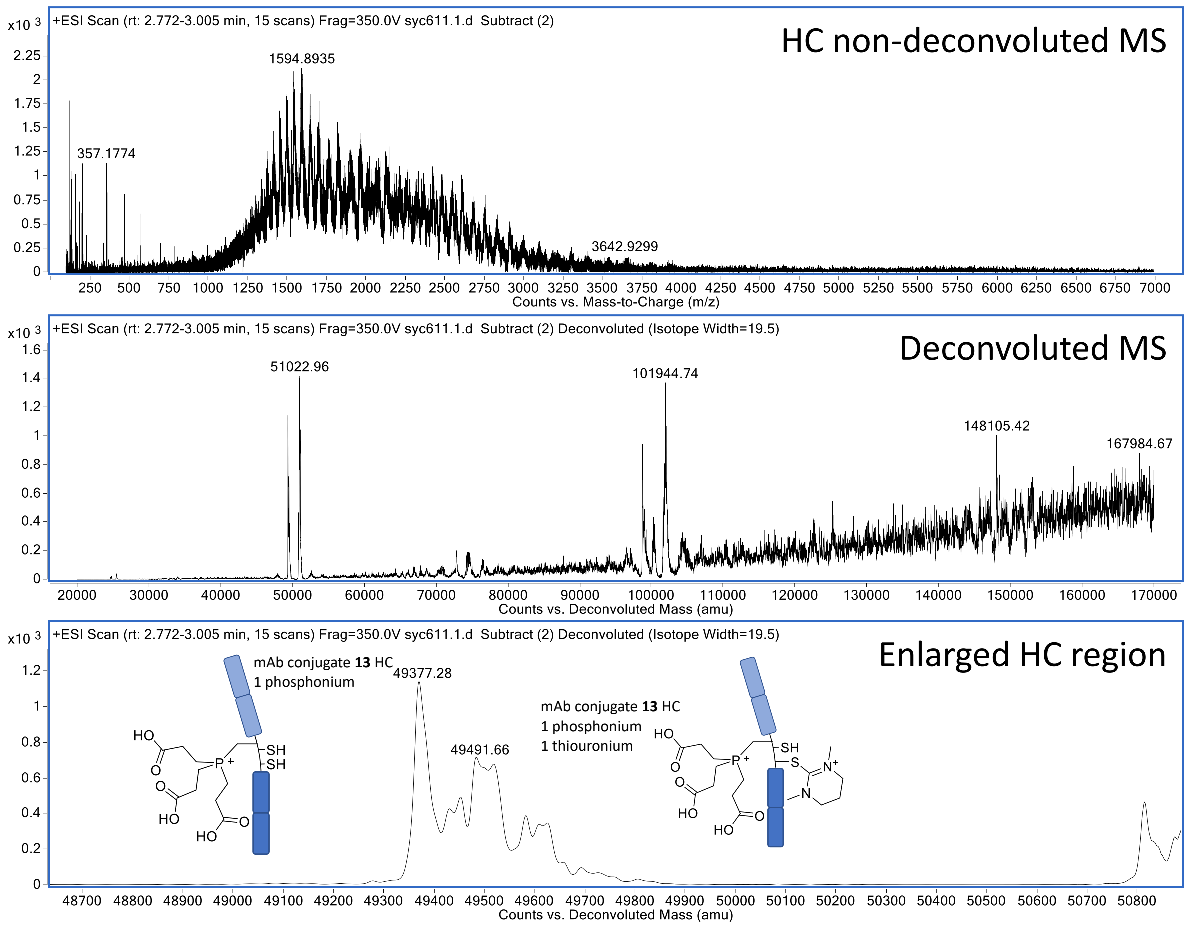

Figure S 43 LCMS mAb conjugate **13**. mAb conjugate **13** free LC, expected mass: 23440 Da, observed mass: 23440 Da; mAb conjugate **13** LC thiouronium, expected mass: 23551 Da, observed mass: 23549 Da; mAb conjugate **13** HC 1 phosphonium, expected mass: 49369 Da, observed mass: 49377 Da; mAb conjugate **13** HC 1 phosphonium 1 thiouronioum, expected mass: 49480 Da, observed mass: 49491 Da.

LCMS mAb conjugate 15

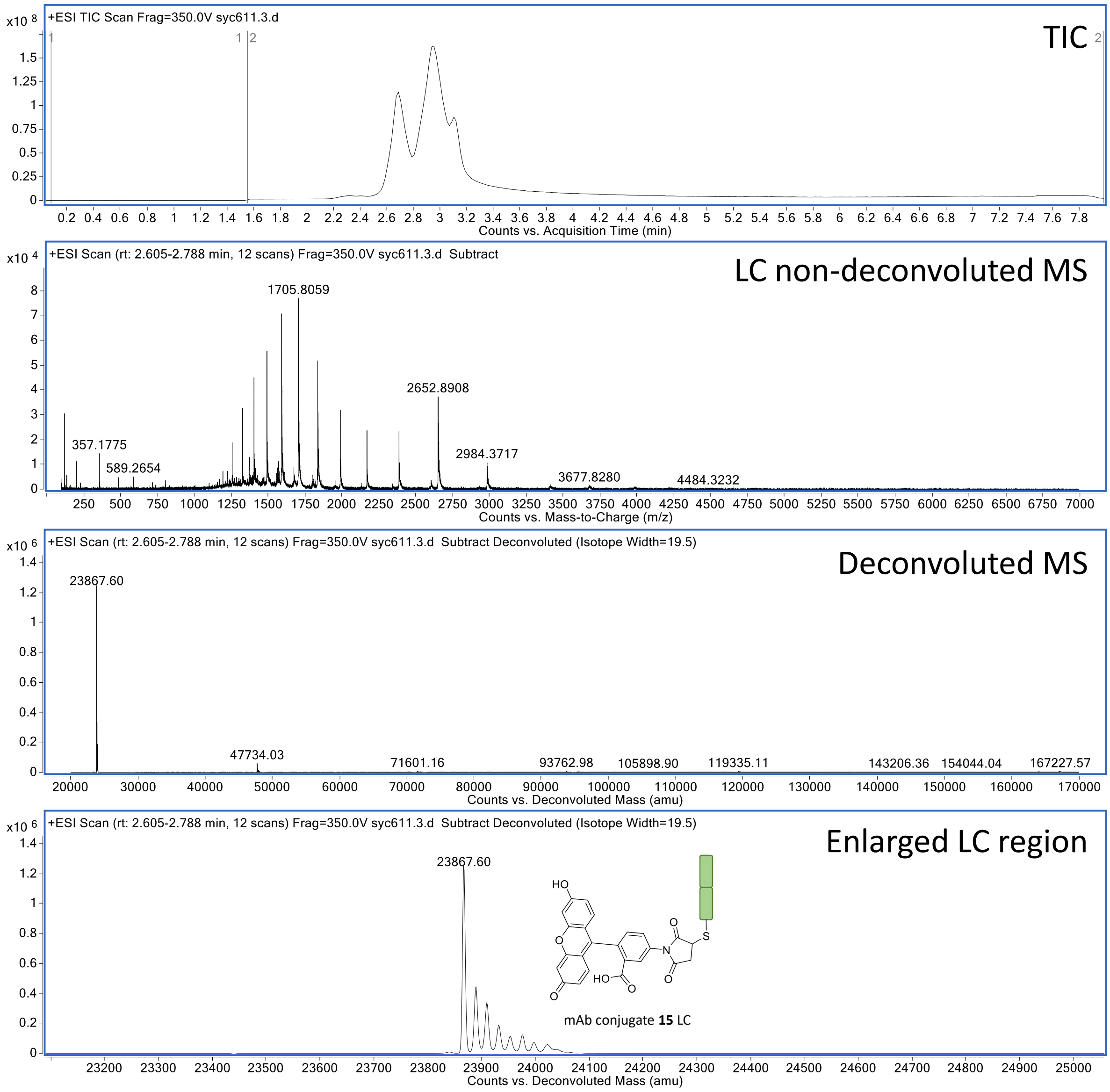

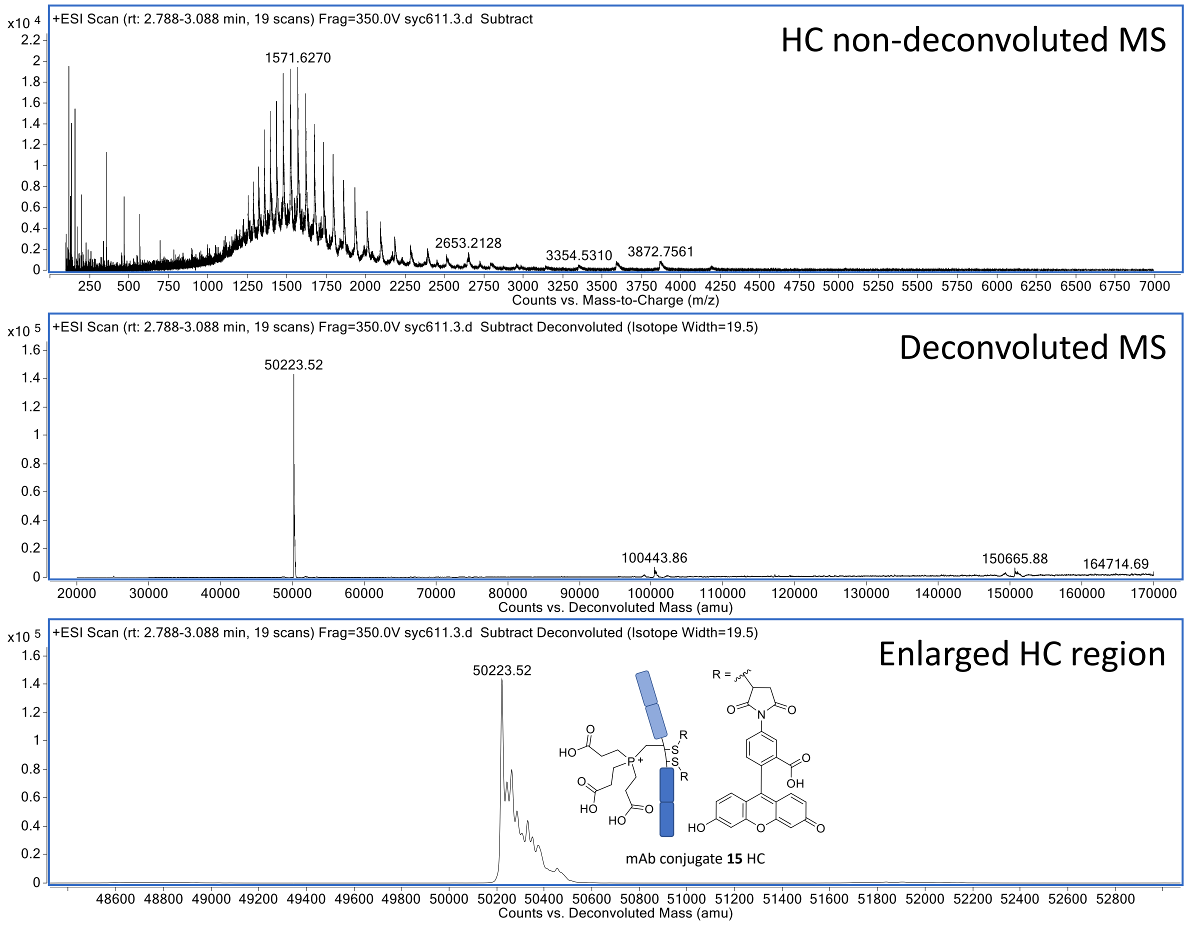

Figure S 44 LCMS of mAb conjugate **15**. mAb conjugate **15** LC, expected mass: 23867 Da, observed mass: 23868 Da; mAb conjugate **15** HC, expected mass: 50223 Da, observed mass: 50223 Da.

LCMS mAb conjugate 16

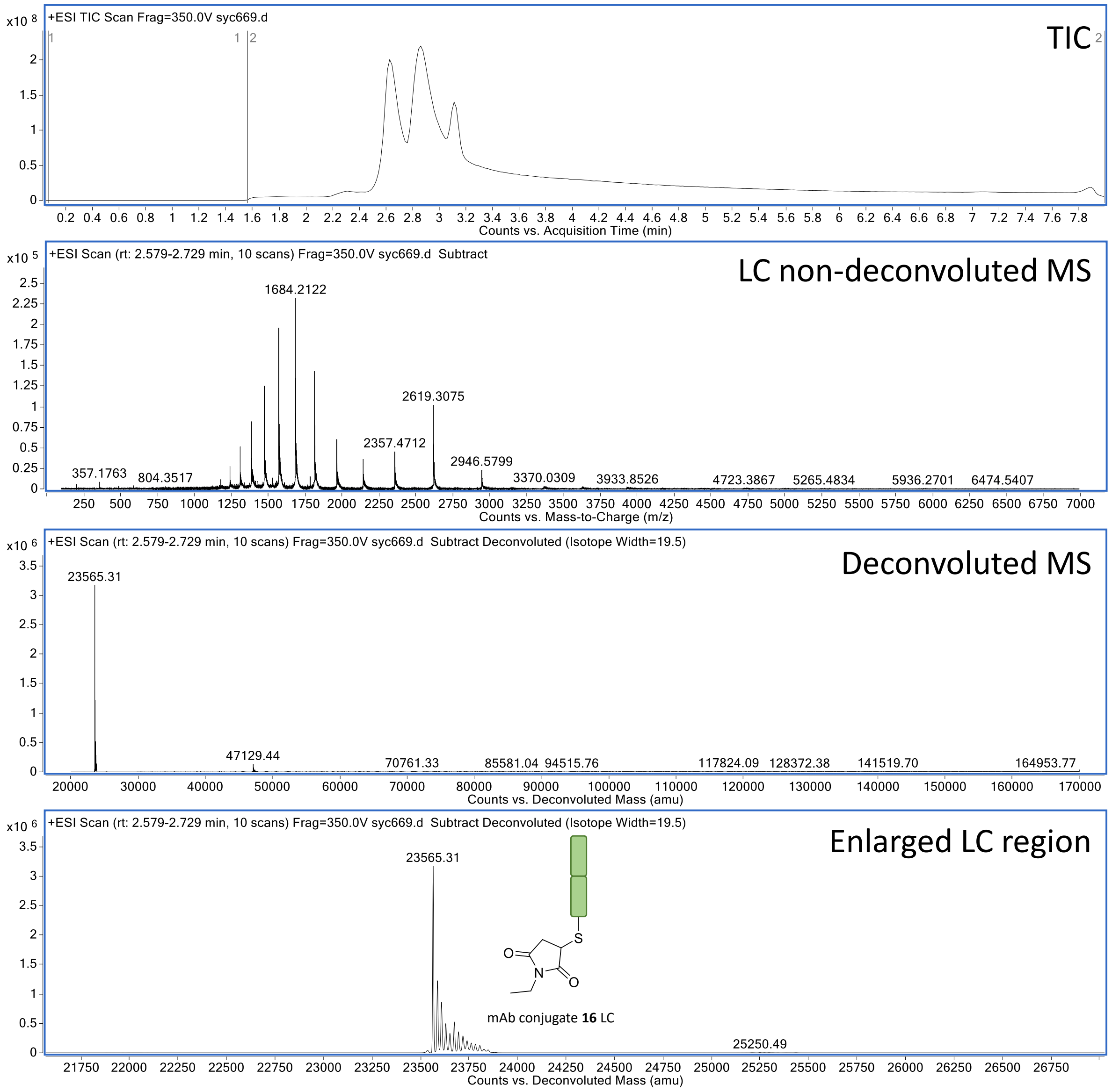

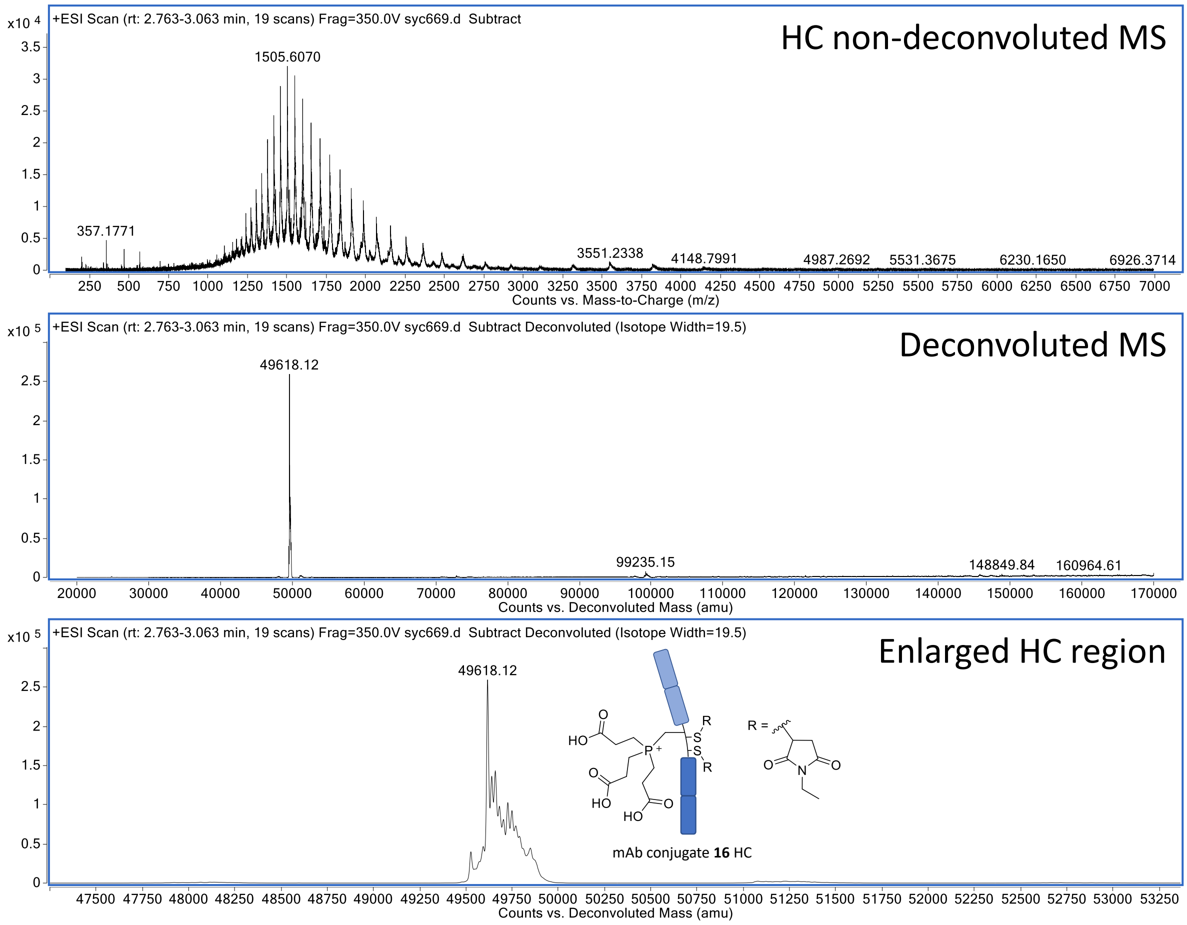

Figure S 45 LCMS of mAb conjugate **16**. mAb conjugate **16** LC, expected mass: 23565 Da, observed mass: 23565 Da; mAb conjugate **16** HC, expected mass: 49619 Da, observed mass: 49618 Da.

Papain digestion of mAb conjugate 16

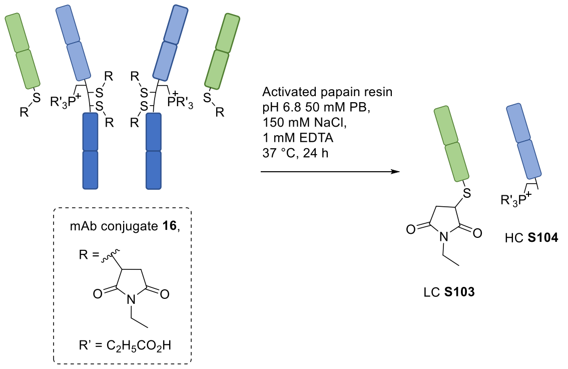

Immobilised papain (ThermoFischer) was activated. Briefly, immobilised papain (50 µL) was added to Pierce™ column (ThermoFischer) and storage buffer removed by centrifugation (5000 rpm, 1 min). Papain resin was washed with pH 6.8 buffer with DTT (50 mM PB, 150 mM NaCl, 1 mM EDTA, 10 mM DTT) (3×500 µL) and incubated with pH 6.8 buffer with DTT (50 mM PB, 150 mM NaCl, 1 mM EDTA, 10 mM DTT) at 37 °C, 1100 rpm for 1.5 h. Papain resin was filtered and washed with pH 6.8 buffer (6×500 µL, 50 mM PB, 150 mM NaCl, 1 mM EDTA) by centrifugation (5000 rpm, 1 min). mAb conjugate **16** (100 µL, 10 mM) in pH 6.8 buffer (50 mM PB, 150 mM NaCl, 1 mM EDTA) was added to Pierce™ column containing activated papain resin and was incubated at 37 °C, 1100 rpm for 24 h. Papain resin was filtered by centrifugation (5000 rpm, 1 min) and the filtrate desalted (ZebaSpin) and analysed using LCMS.

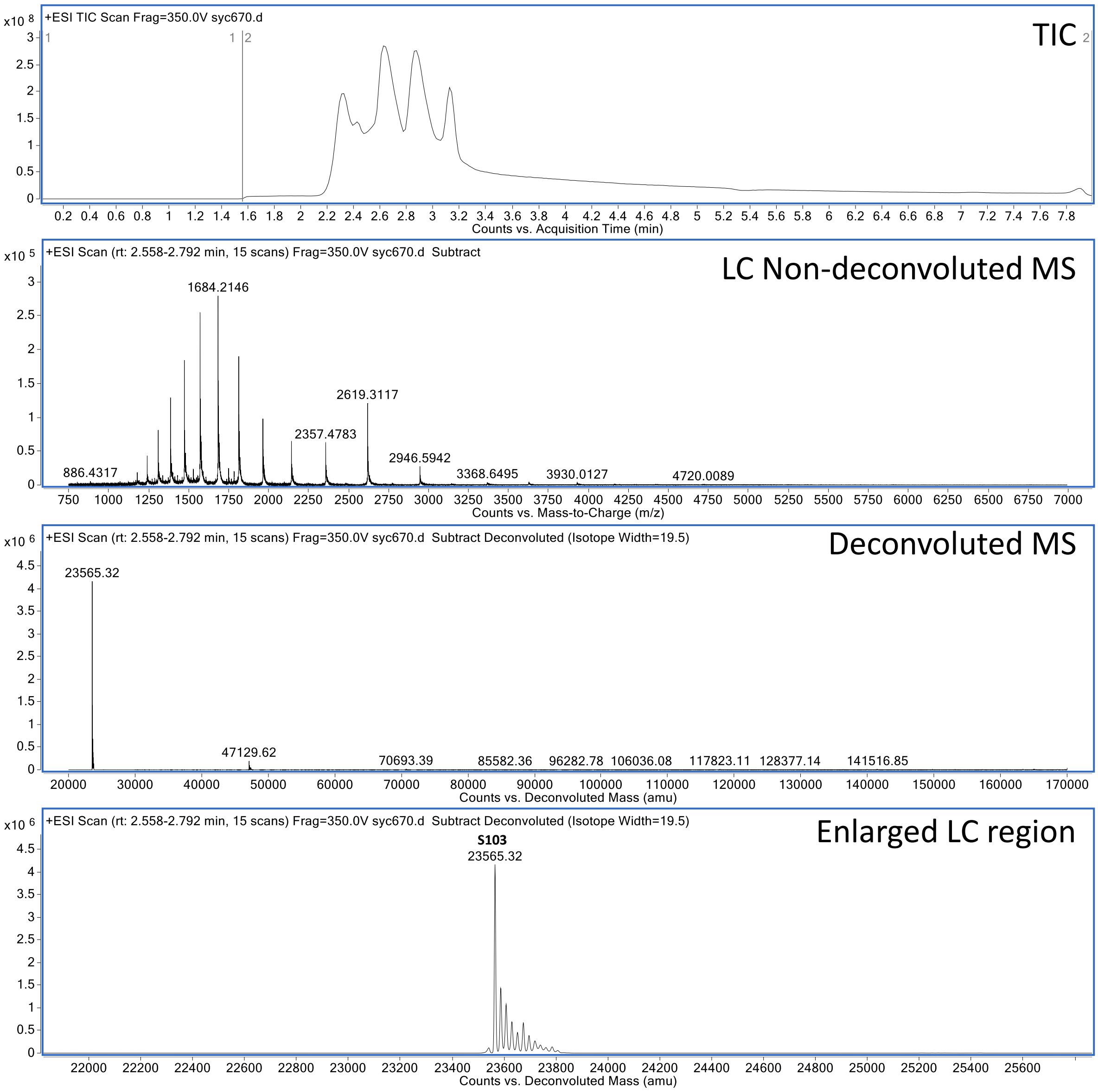
+ESI TIC Scan Frag=350.0V syc670.d
TIC
x10 8
Counts vs. Acquisition Time (min)
+ESI Scan (rt: 2.558-2.792 min, 15 scans) Frag=350.0V syc670.d Subtract
LC Non-deconvoluted MS
x10 5
1684.2146
2619.3117
2357.4783
2946.5942
886.4317
3368.6495
3930.0127
4720.0089
Counts vs. Mass-to-Charge (m/z)
+ESI Scan (rt: 2.558-2.792 min, 15 scans) Frag=350.0V syc670.d Subtract Deconvoluted (Isotope Width=19.5)
Deconvoluted MS
x10 6
23565.32
47129.62
70693.39
85582.36
96282.78
106036.08
117823.11
128377.14
141516.85
Counts vs. Deconvoluted Mass (amu)
+ESI Scan (rt: 2.558-2.792 min, 15 scans) Frag=350.0V syc670.d Subtract Deconvoluted (Isotope Width=19.5)
Enlarged LC region
x10 6
S103
23565.32
Counts vs. Deconvoluted Mass (amu)

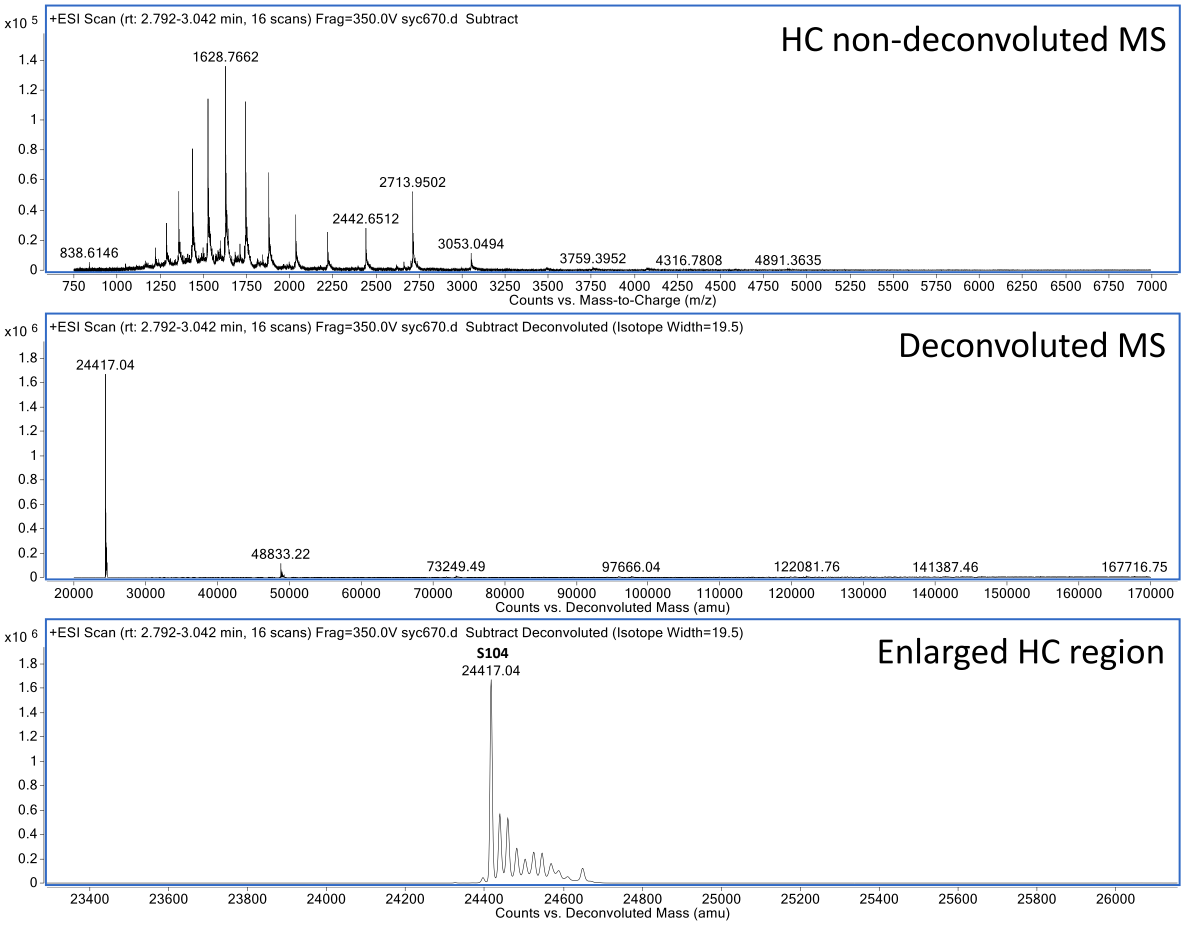

Figure S 46 LCMS of papain digested mAb conjugate **16**. **S103** expected mass: 23565 Da, observed mass: 23565 Da; **S104** expected mass: 24417 Da, observed mass: 24417 Da.

Small molecule experiments

General procedure: To small molecule (**S2**, **S4**, **S106** or *N*-acetyl-cysteine, final concentration of 0.5 mM) in buffer (25 mM) was added reagents (1.1 eq. HOTT **6**, 1.1 eq. EDC **4**, 2 eq. MKYM **3**, or 15 eq. CDMP **8**) and TCEP (1.1 eq., for EDC **4** MKYM **3**, CDMP **8** only). The reaction was incubated at 37 °C for 24 h. Reaction mixture was diluted 1:1 with water prior to LCMS analysis.

Reaction of HOTT 6 with S2

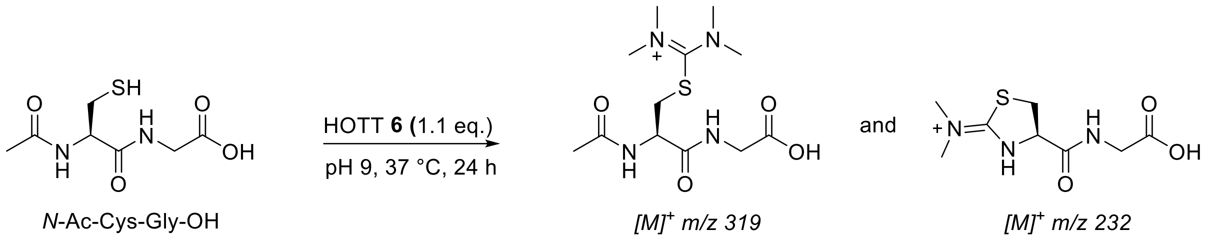

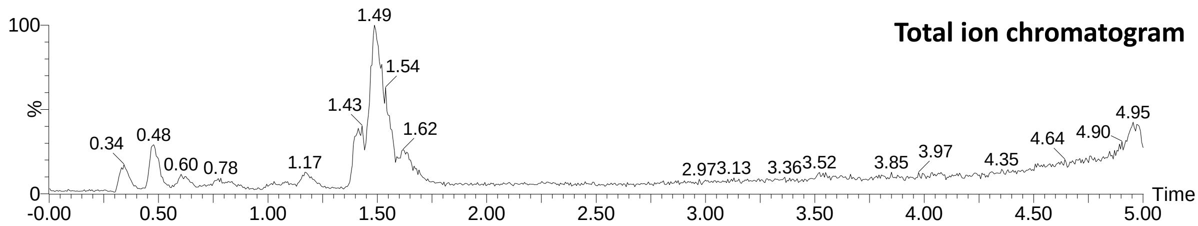

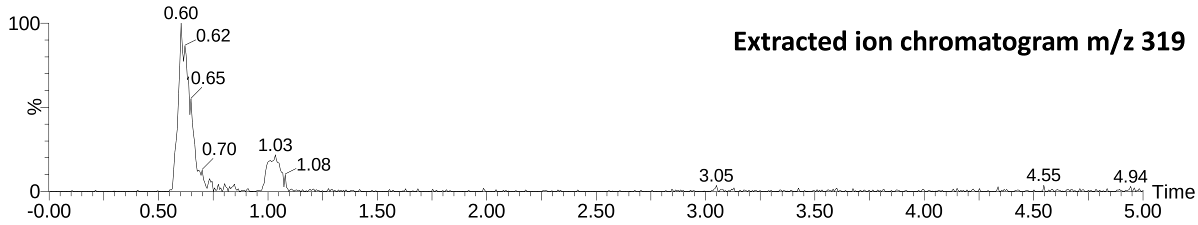

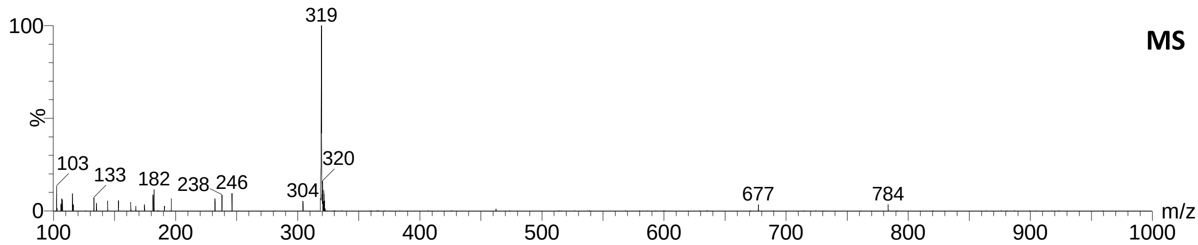

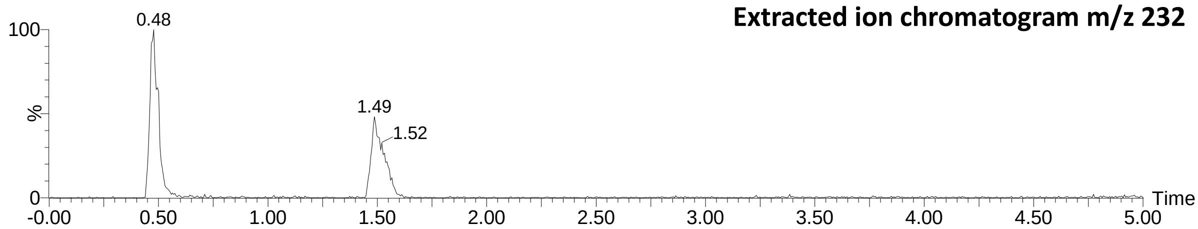

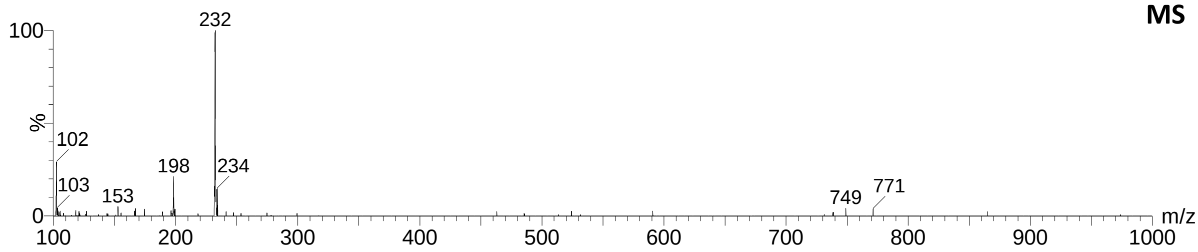

Reaction of MKYM 3 and TCEP with S2

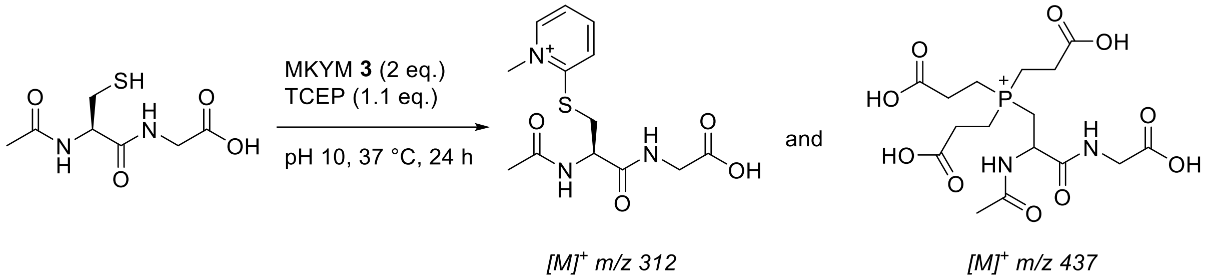

Total ion chromatogram

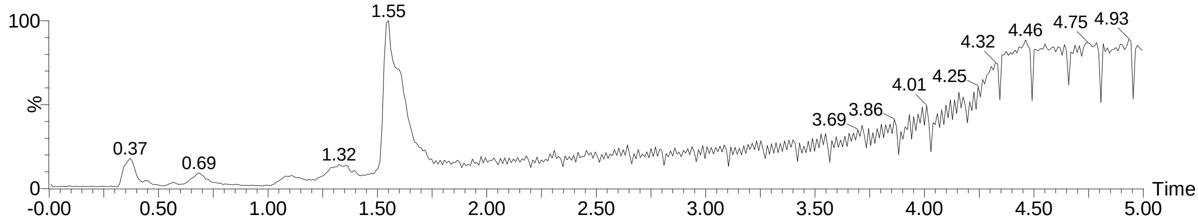

Extracted ion chromatogram m/z 312

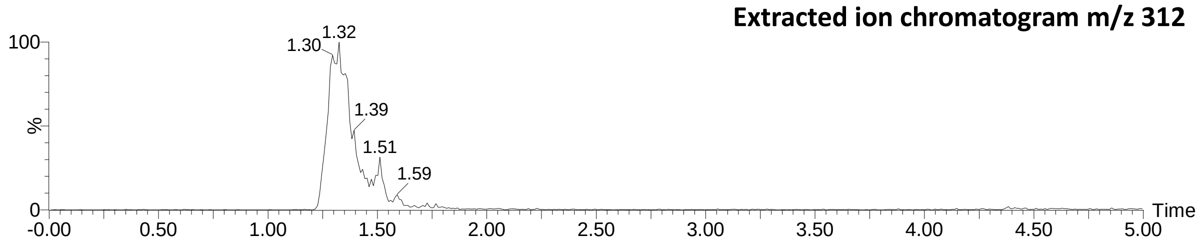

MS

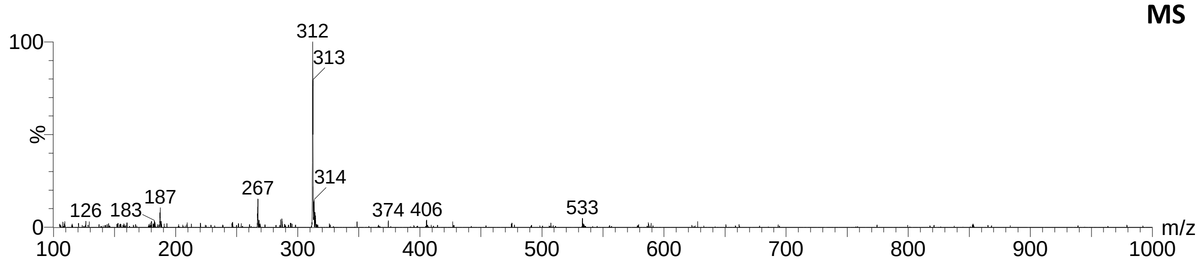

Extracted ion chromatogram m/z 437

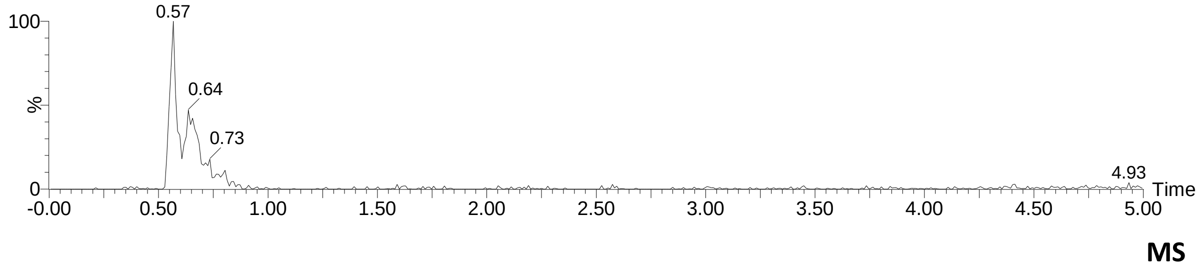

MS

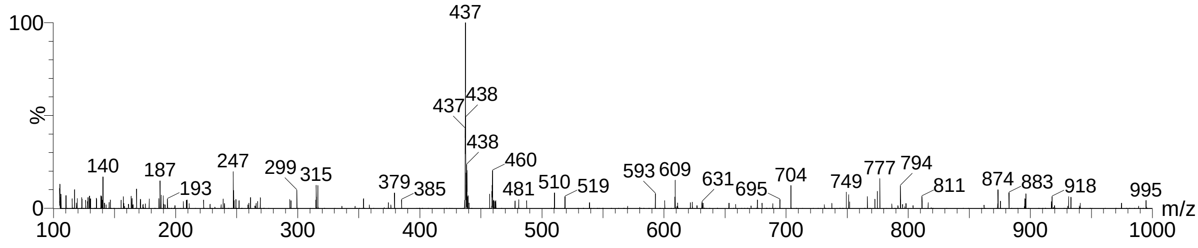

Reaction of MKYM 3 and TCEP with S4

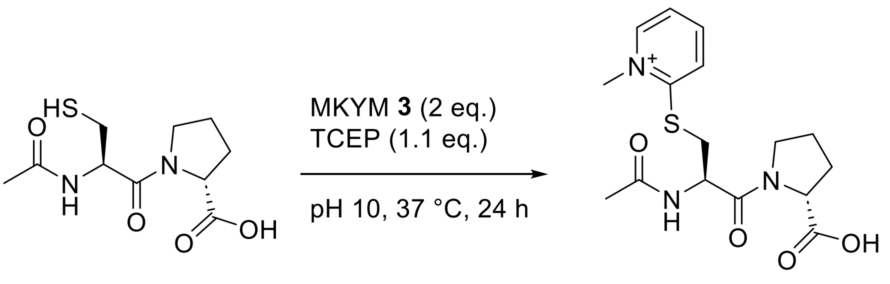

[M]+ m/z 352

Total ion chromatogram

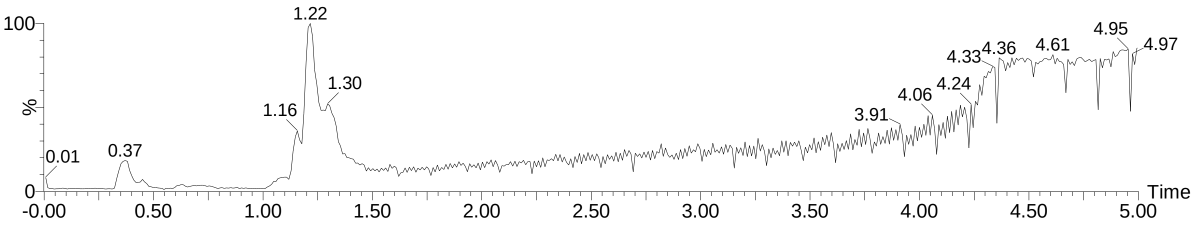

Extracted ion chromatogram m/z 352

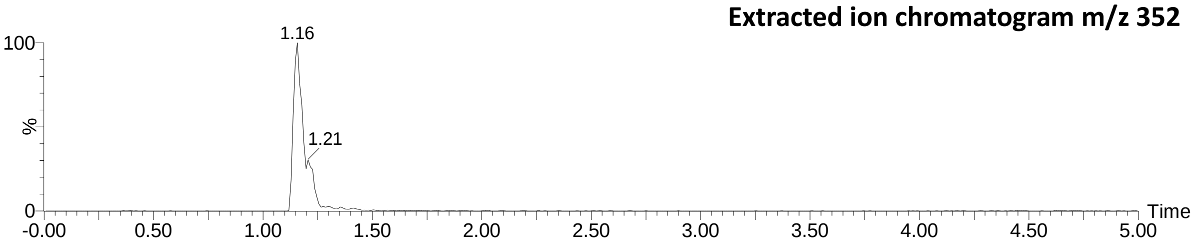

MS

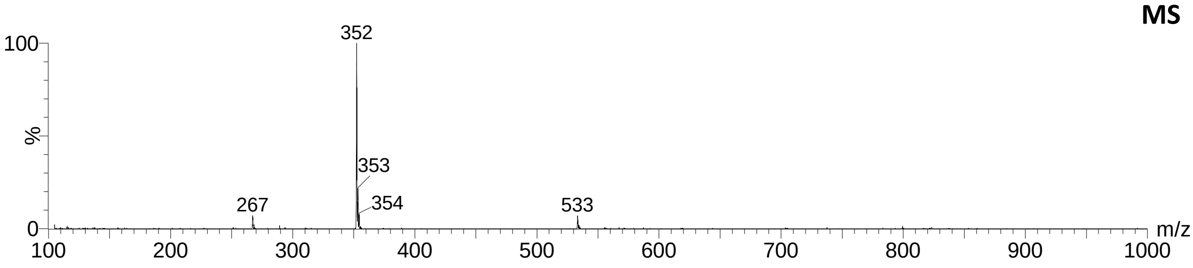

Reaction of MKYM 3 and TCEP with *N*-acetyl-cysteine

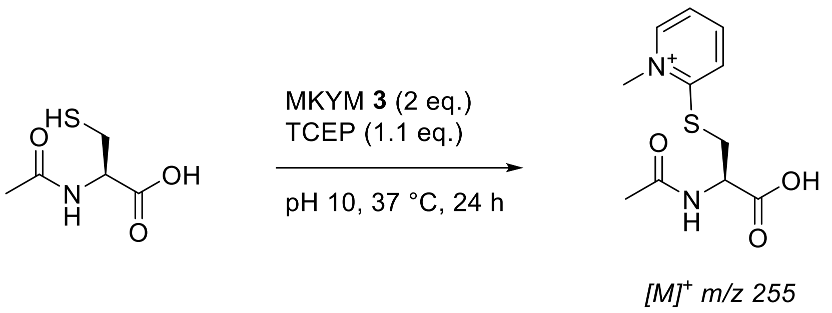

Total ion chromatogram

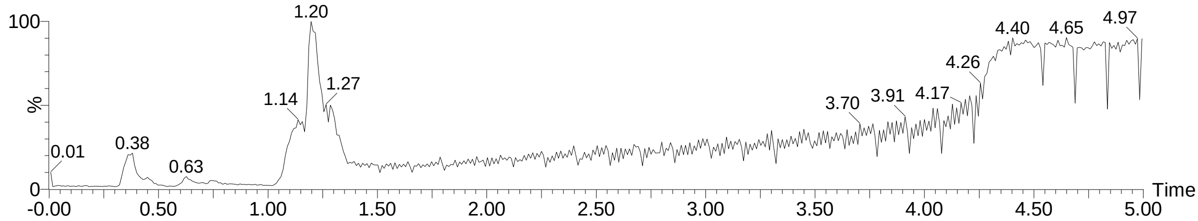

Extracted ion chromatogram m/z 255

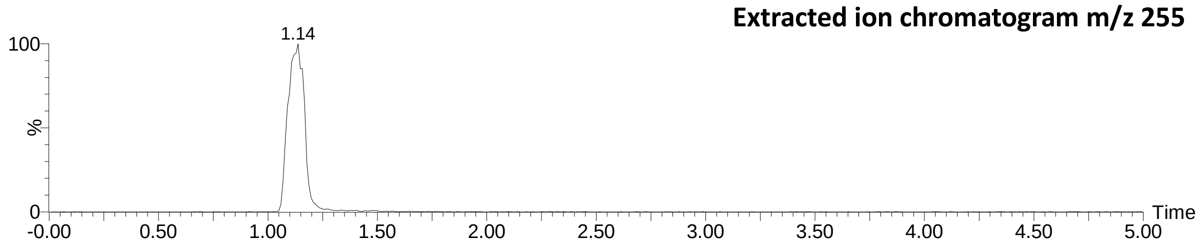

MS

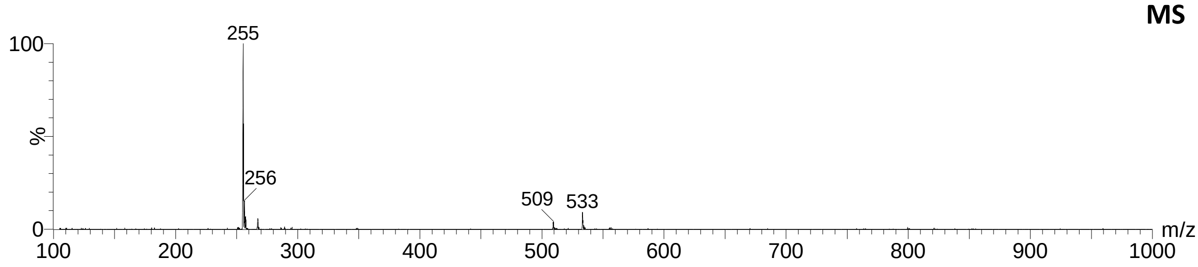

Reaction of MKYM 3 with N-Ac-Cys-Asp-OH S106

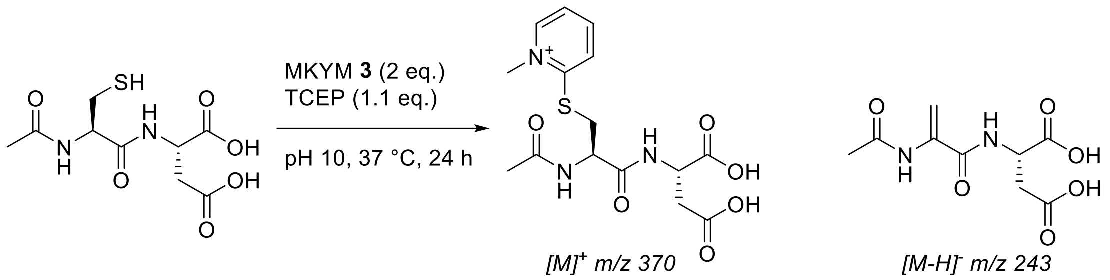

Positive ESI

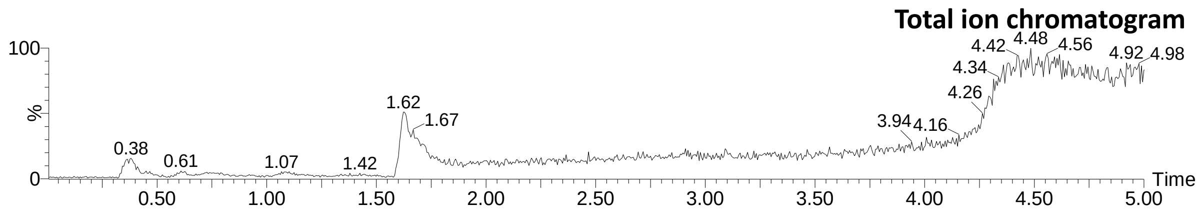

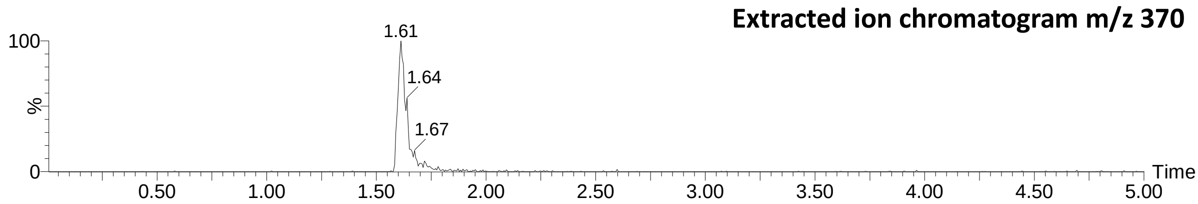

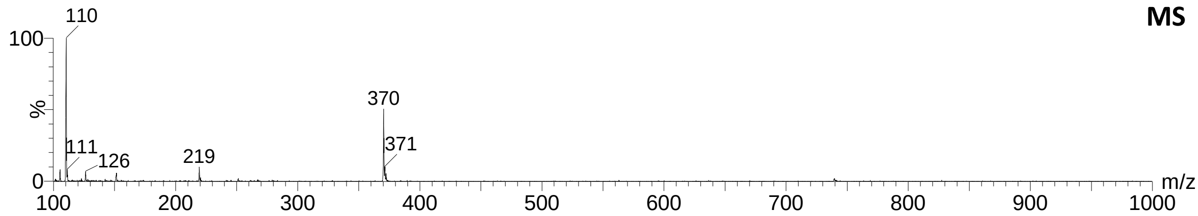

Negative ESI

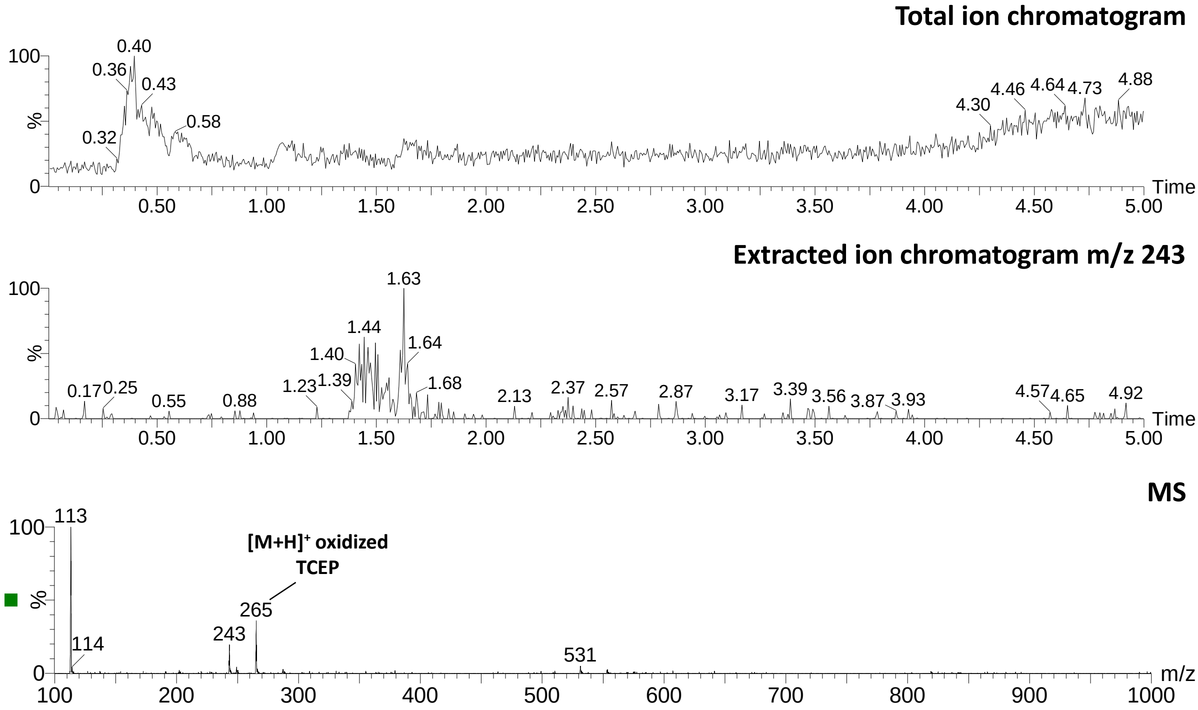

Reaction of EDC 4 with S2

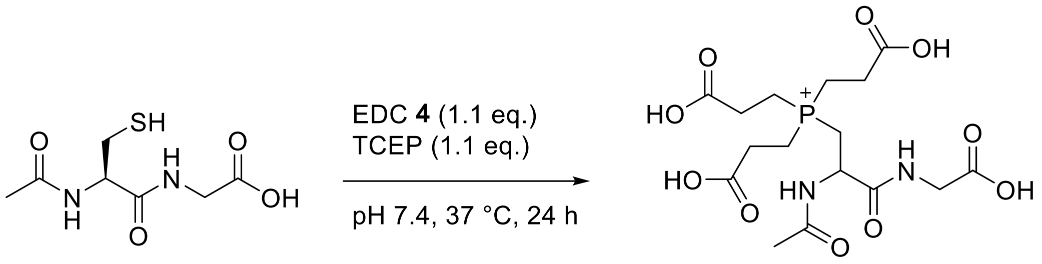

[M]+ *m/z 437*

Total ion chromatogram

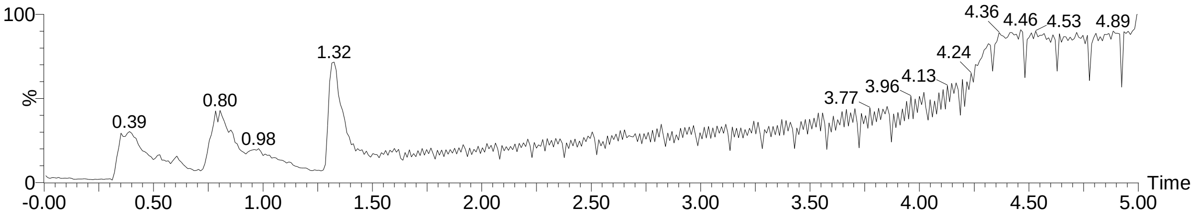

Extracted ion chromatogram m/z 437

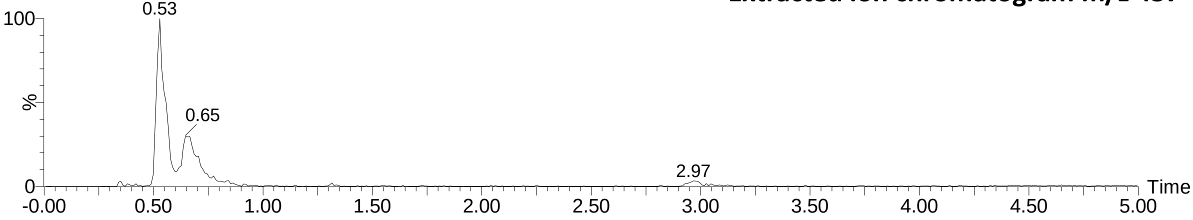

MS

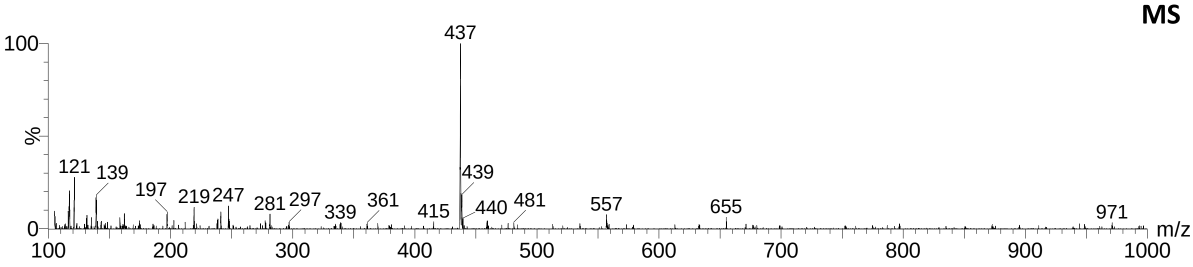

Reaction of EDC 4 and TCEP with S4

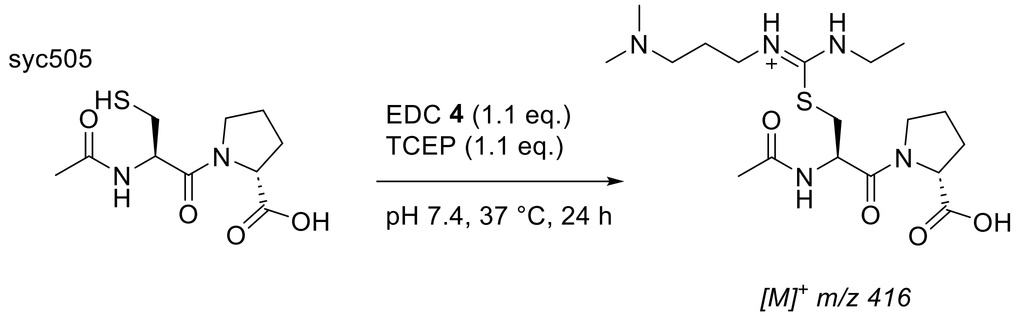

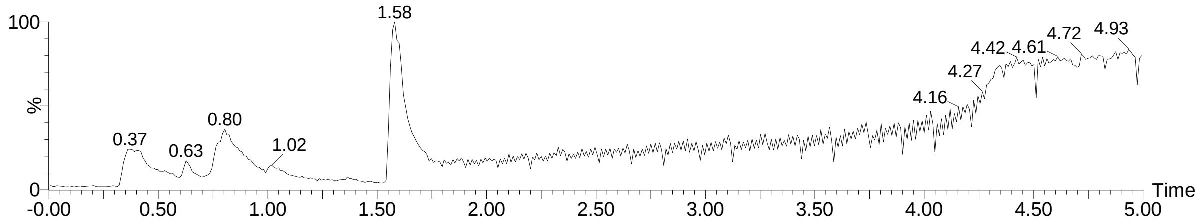

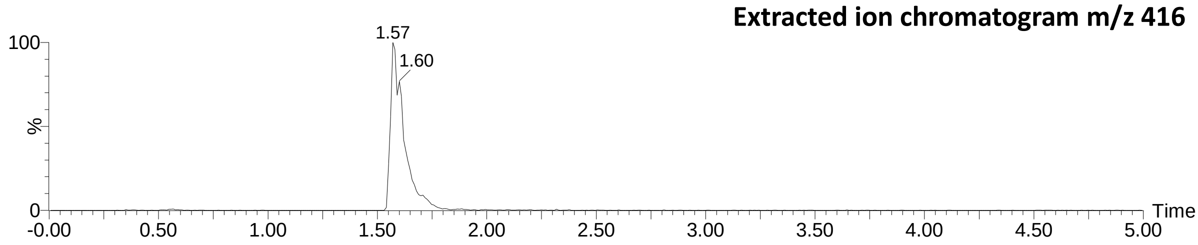

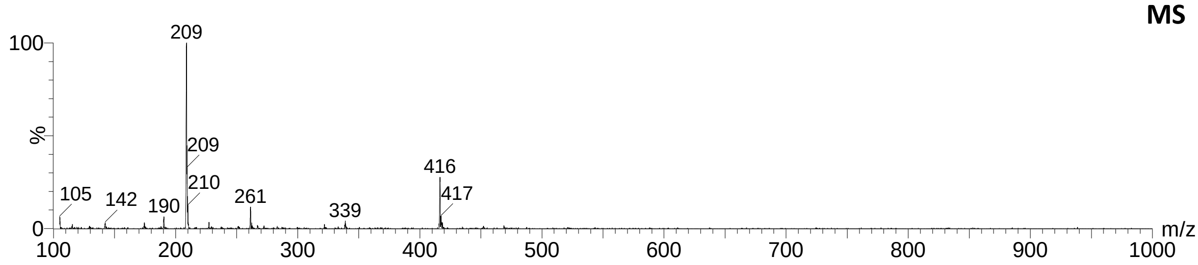

Reaction of EDC 4 and TCEP with *N*-acetyl-cysteine

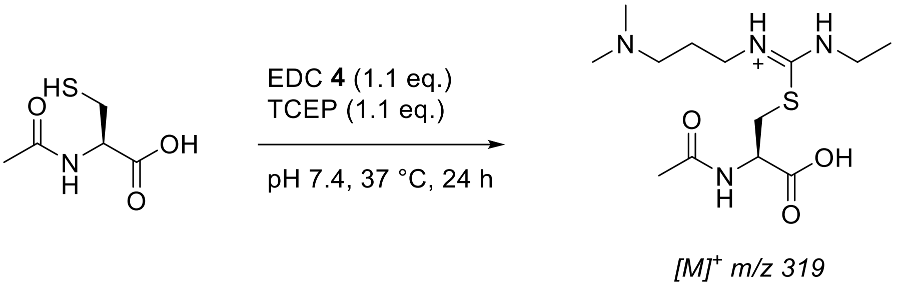

Total ion chromatogram

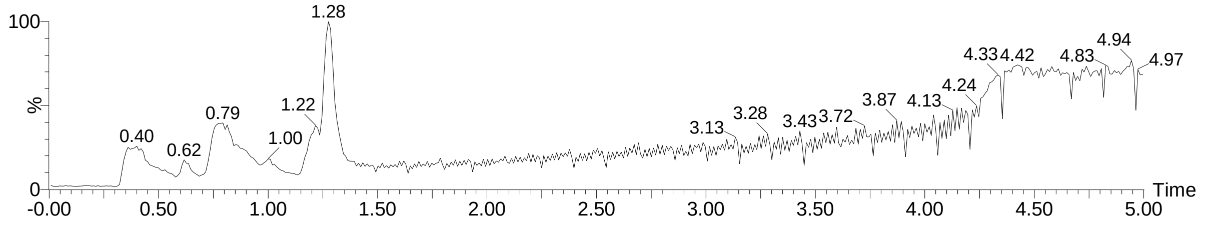

Extracted ion chromatogram m/z 319

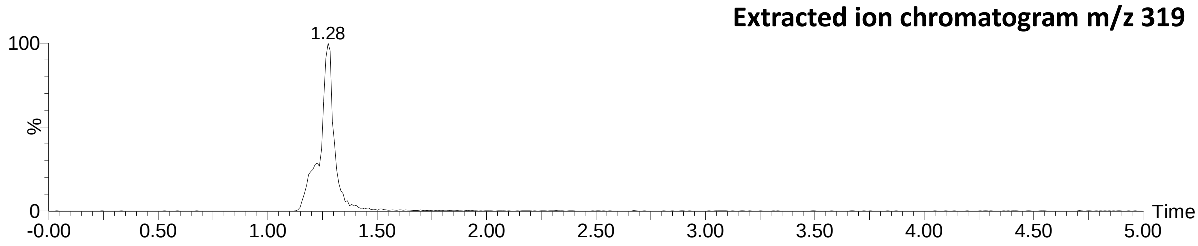

MS

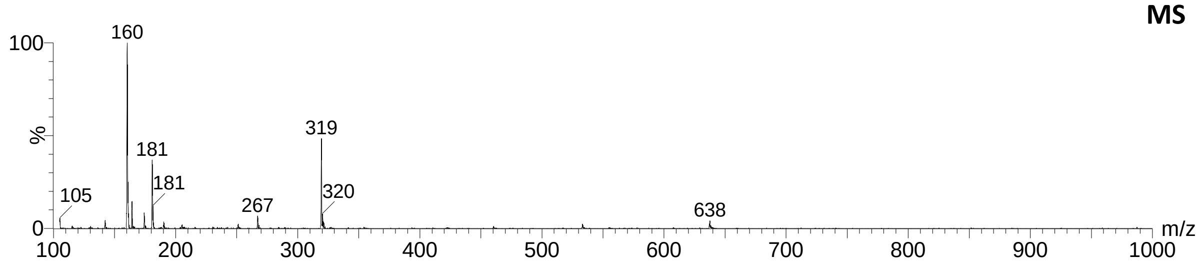

Reaction of EDC 4 with N-Ac-Cys-Asp-OH S106

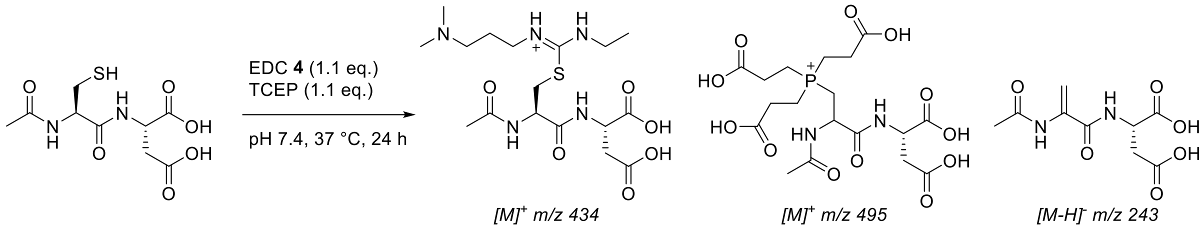

Positive ESI

Total ion chromatogram

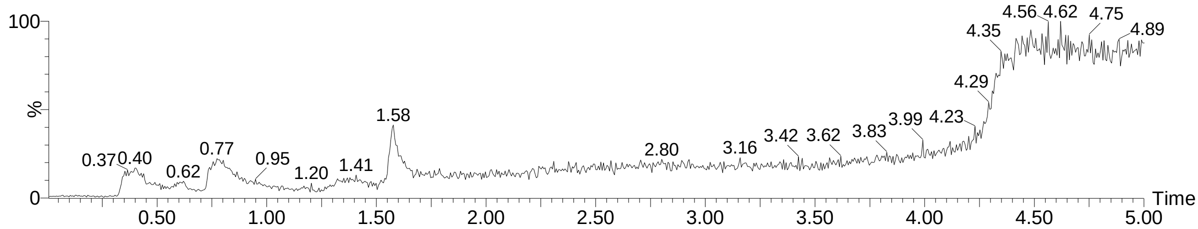

Extracted ion chromatogram m/z 434

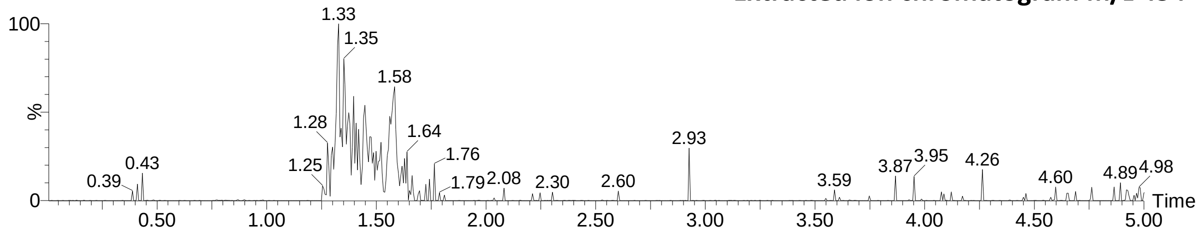

MS

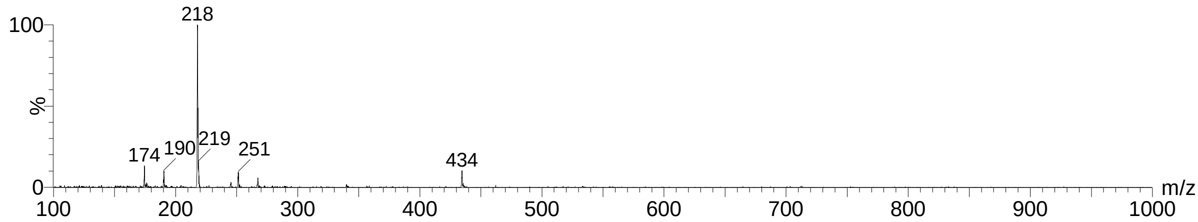

Extracted ion chromatogram m/z 495

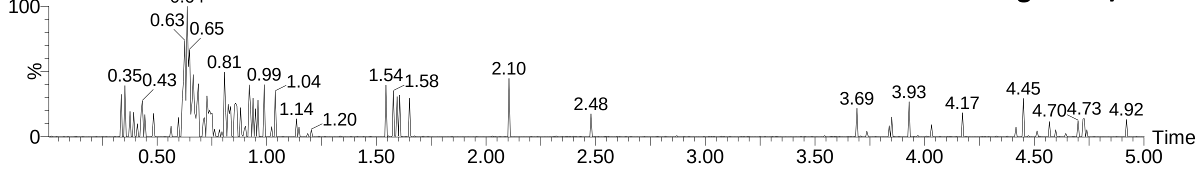

MS

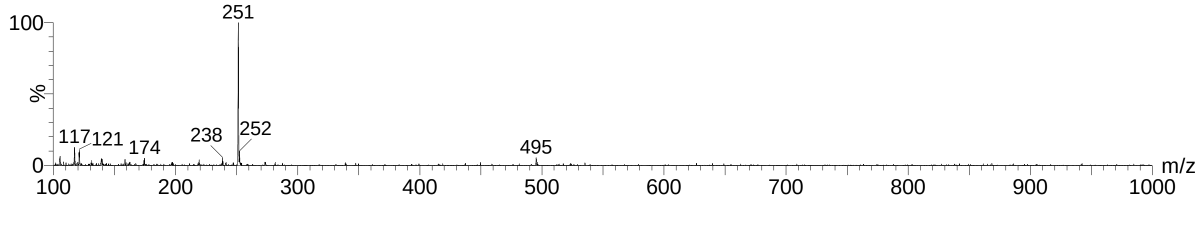

Negative ESI

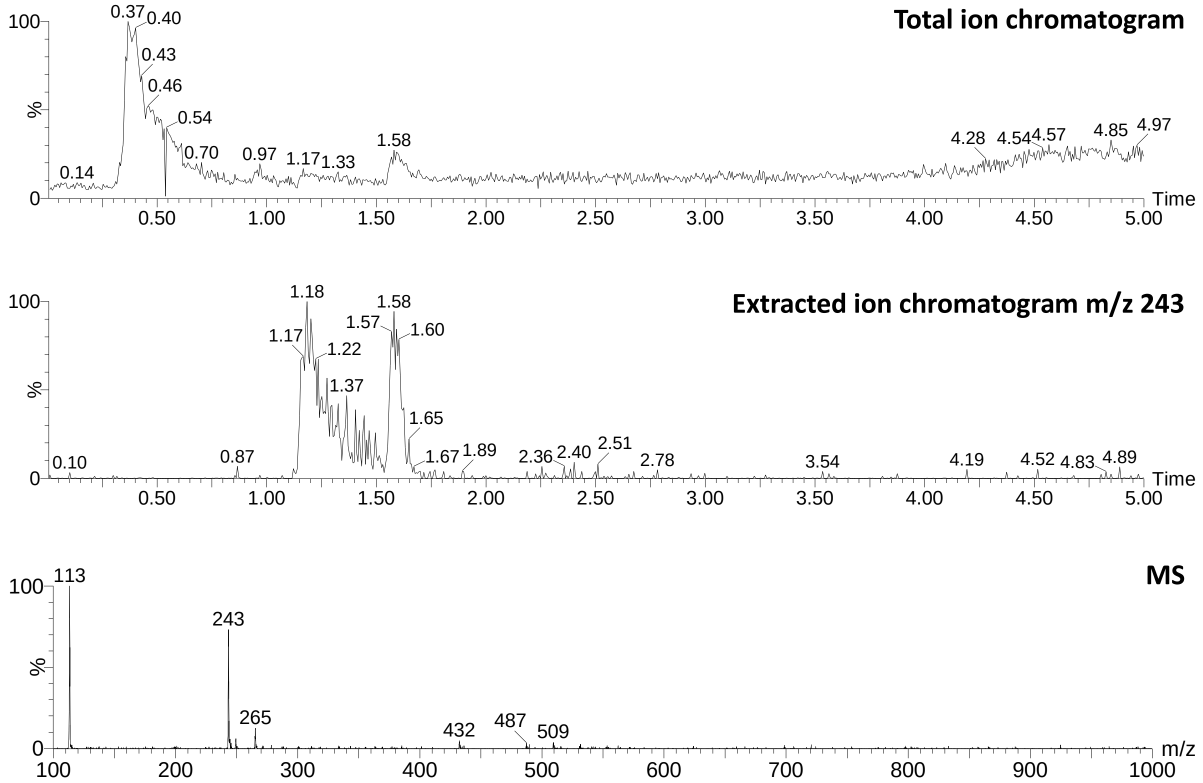

Reaction of CDMP 8 and TCEP with S2

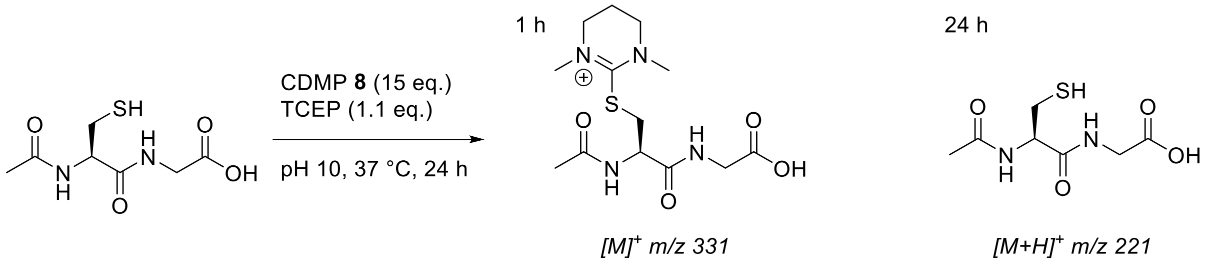

1 h timepoint

Total ion chromatogram

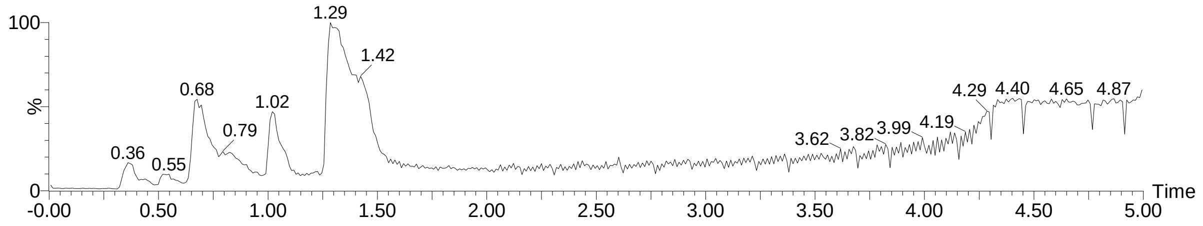

Extracted ion chromatogram m/z 331

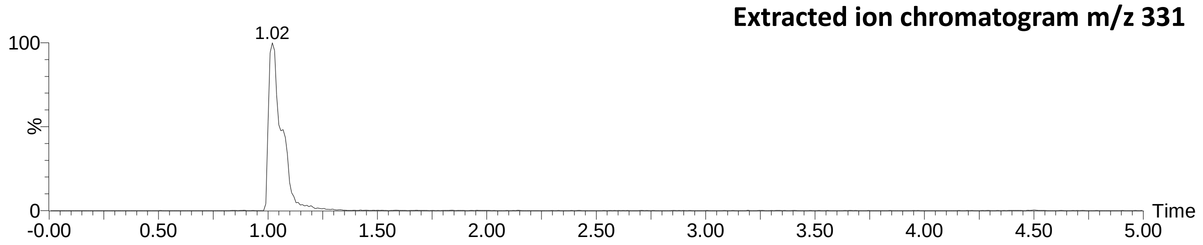

MS

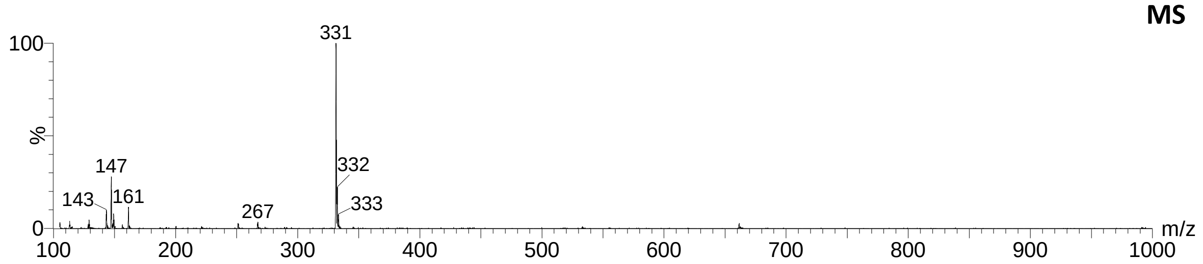

24 h timepoint

Total ion chromatogram

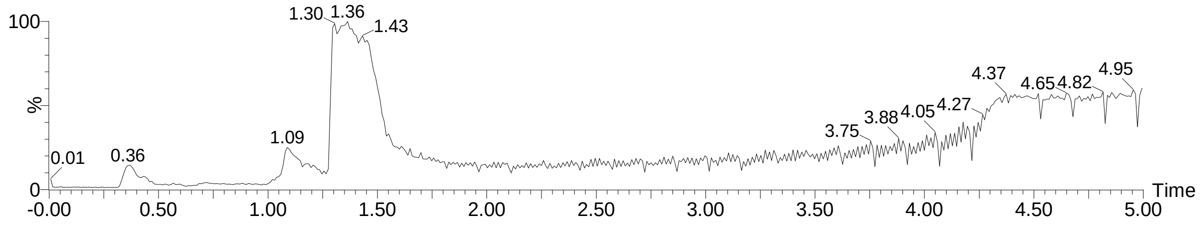

Extracted ion chromatogram m/z 221

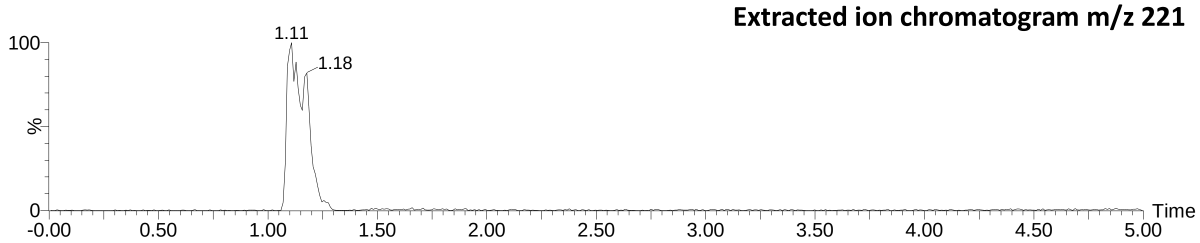

MS

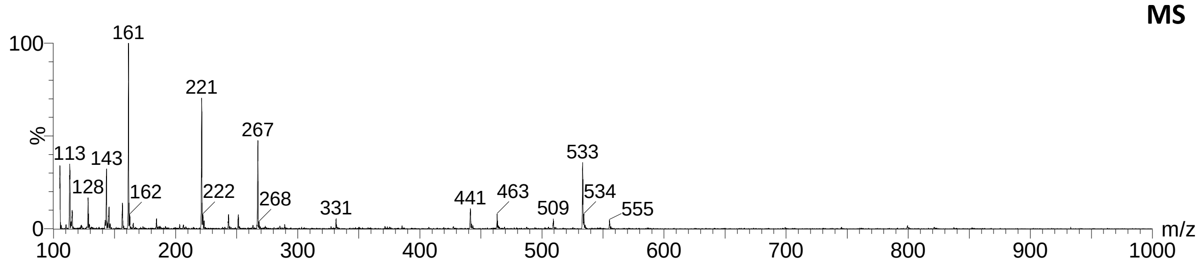

Reaction of CDMP 8 and TCEP with S4

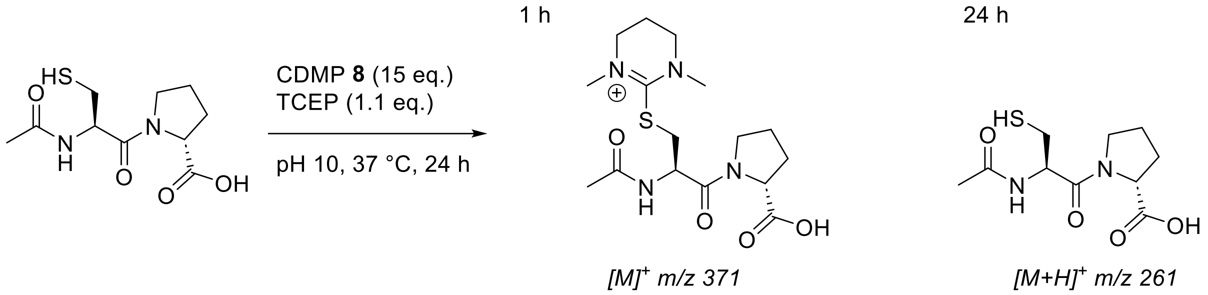

1 h timepoint

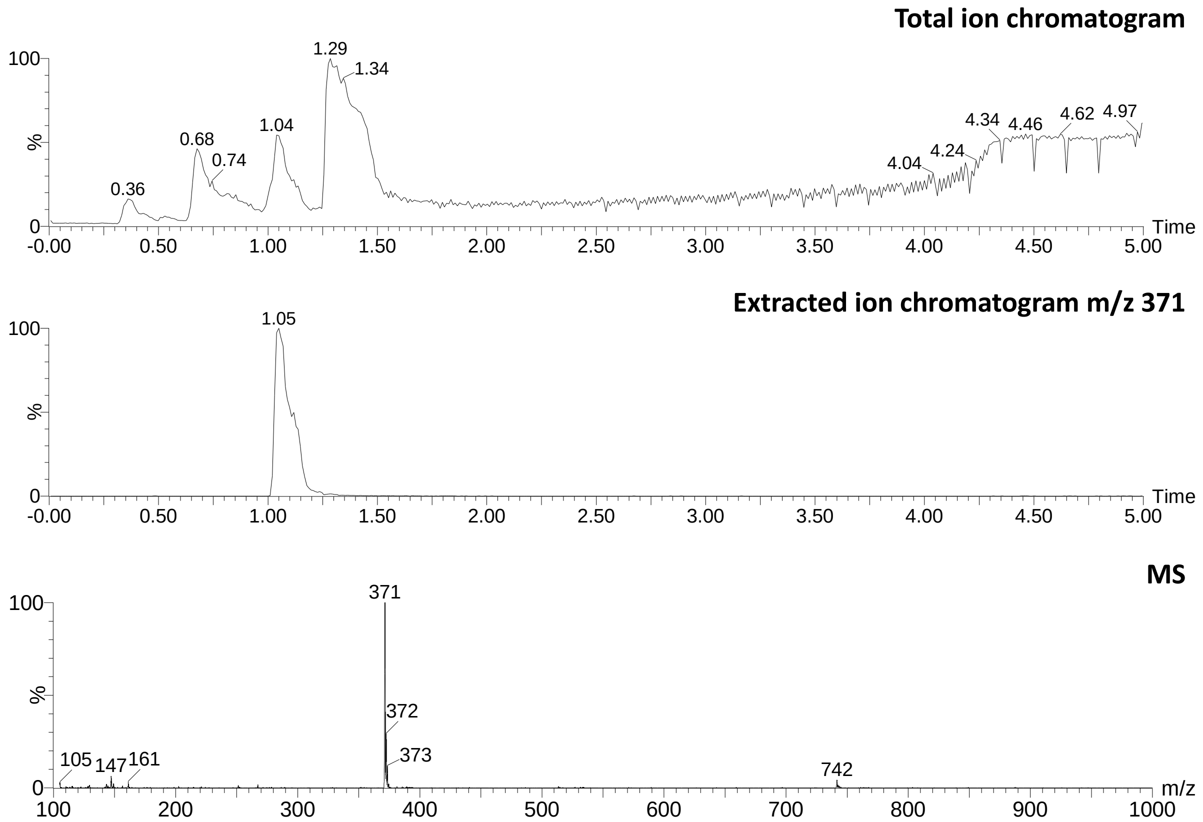

24 h timepoint

Total ion chromatogram

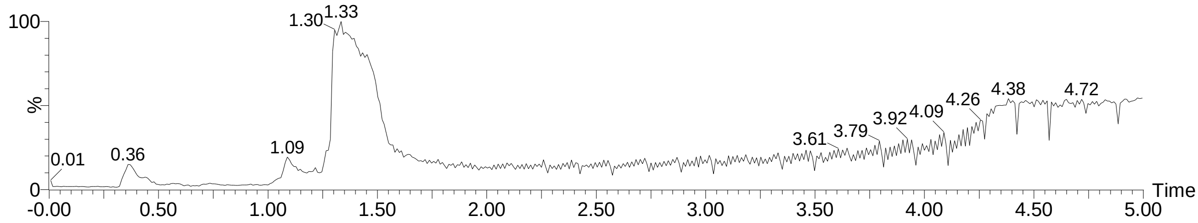

Extracted ion chromatogram m/z 261

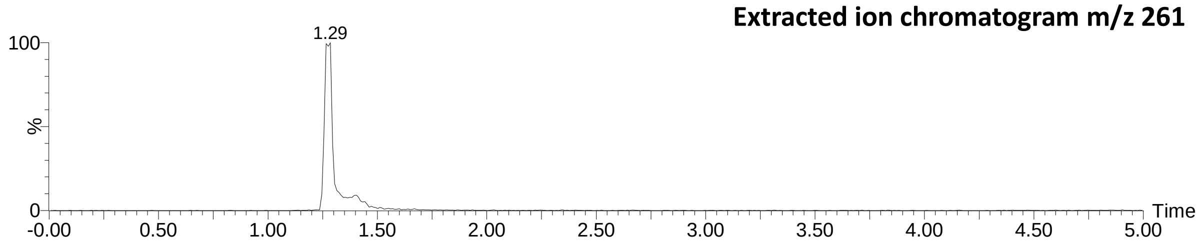

MS

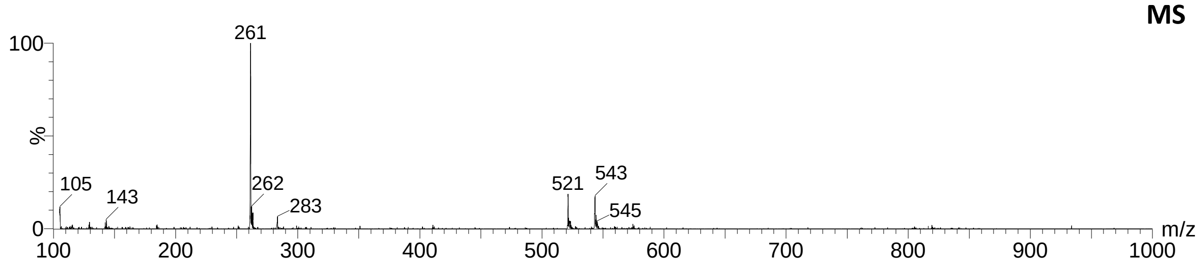

Reaction of CDMP 8 and TCEP with *N*-acetyl-cysteine

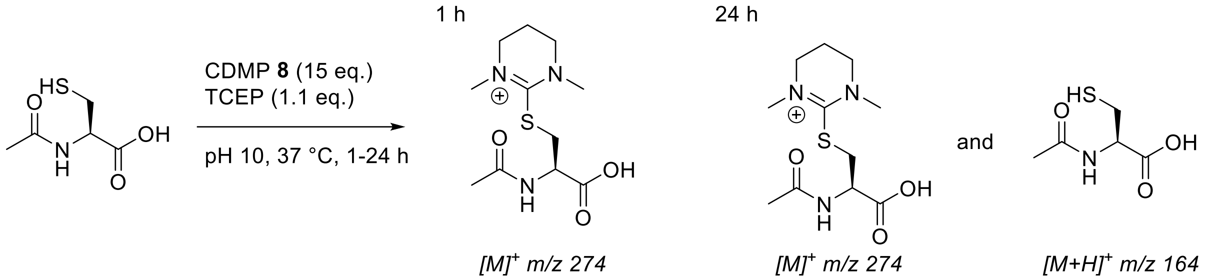

1 h timepoint

Total ion chromatogram

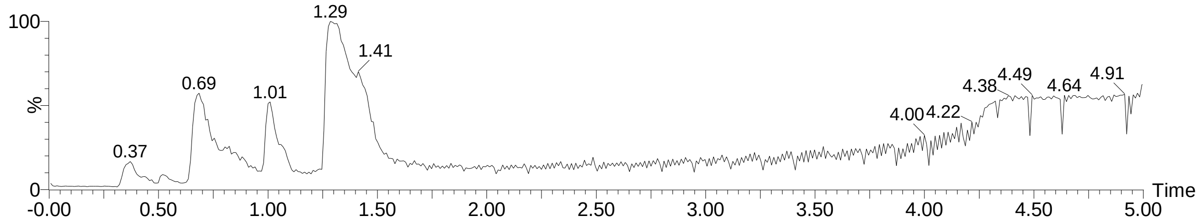

Extracted ion chromatogram m/z 274

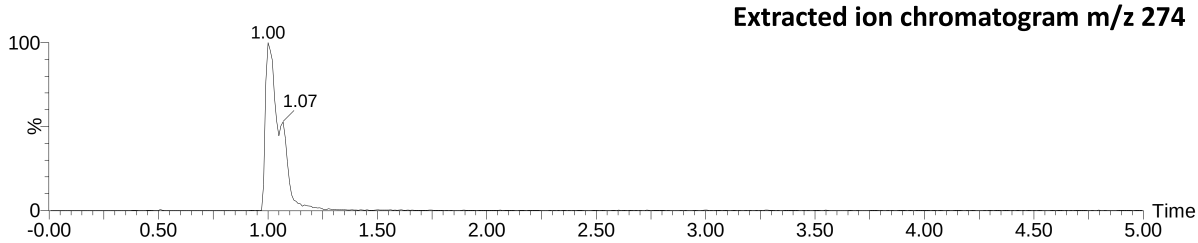

MS

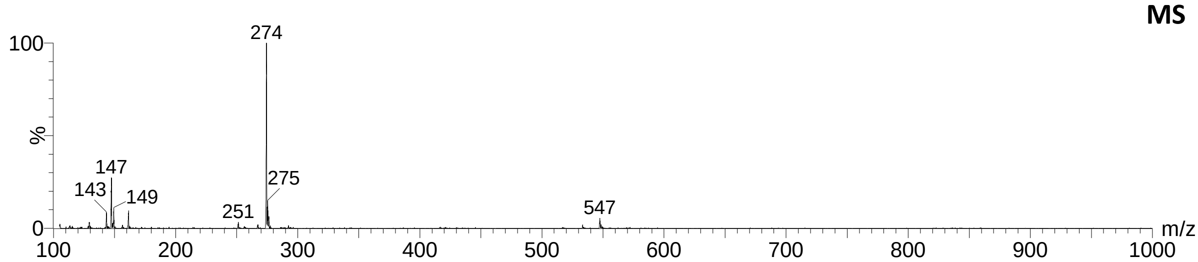

24 h timepoint

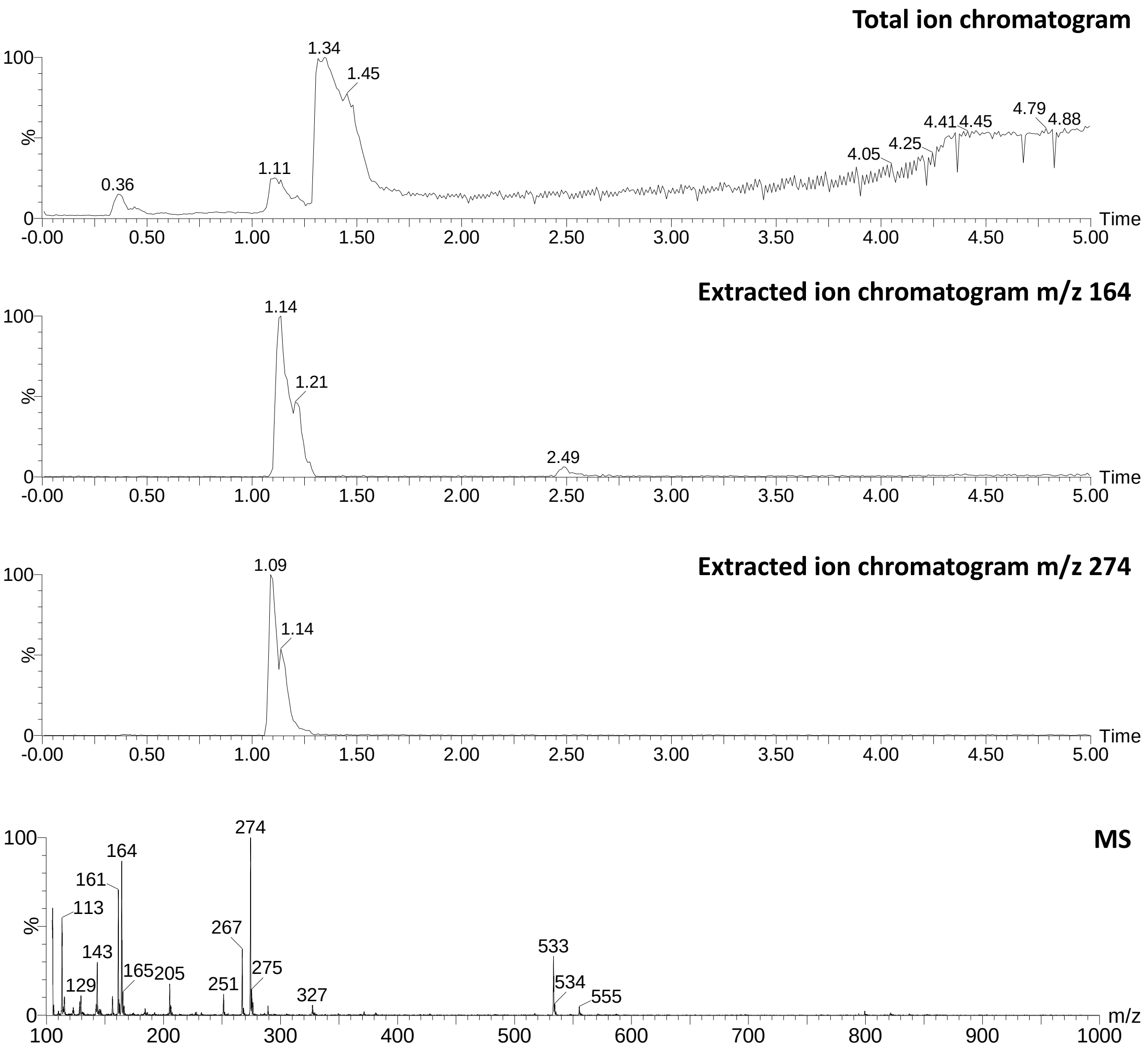

Reaction of CDMP 8 with N-Ac-Cys-Asp-OH S106

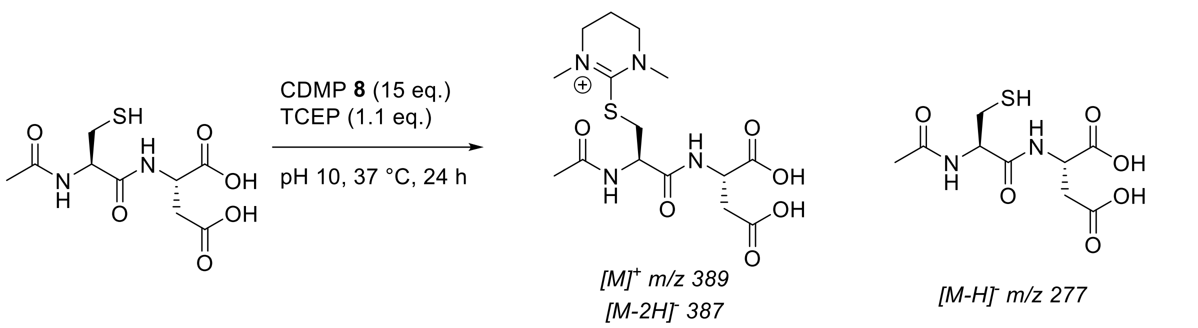

Positive ESI

Total ion chromatogram

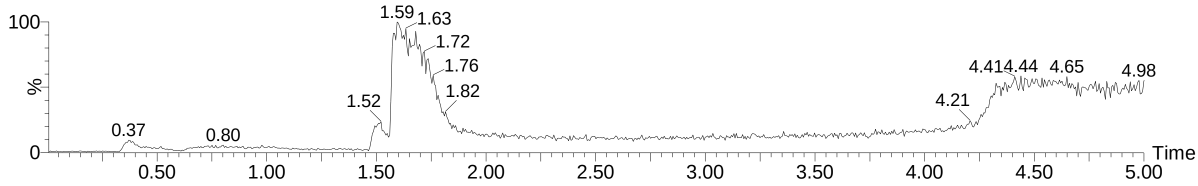

Extracted ion chromatogram m/z 389

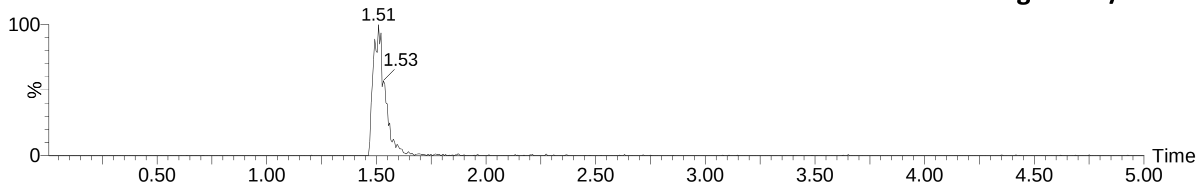

MS

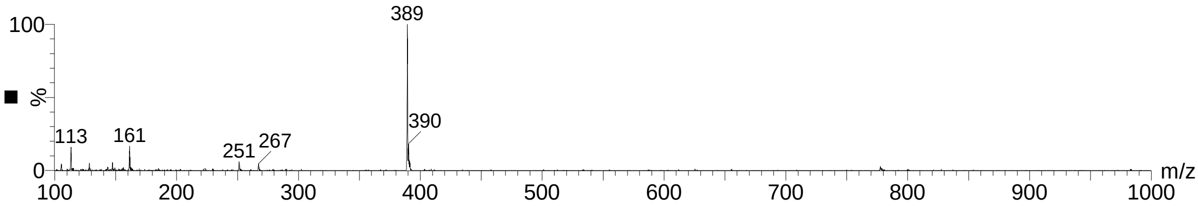

Negative ESI

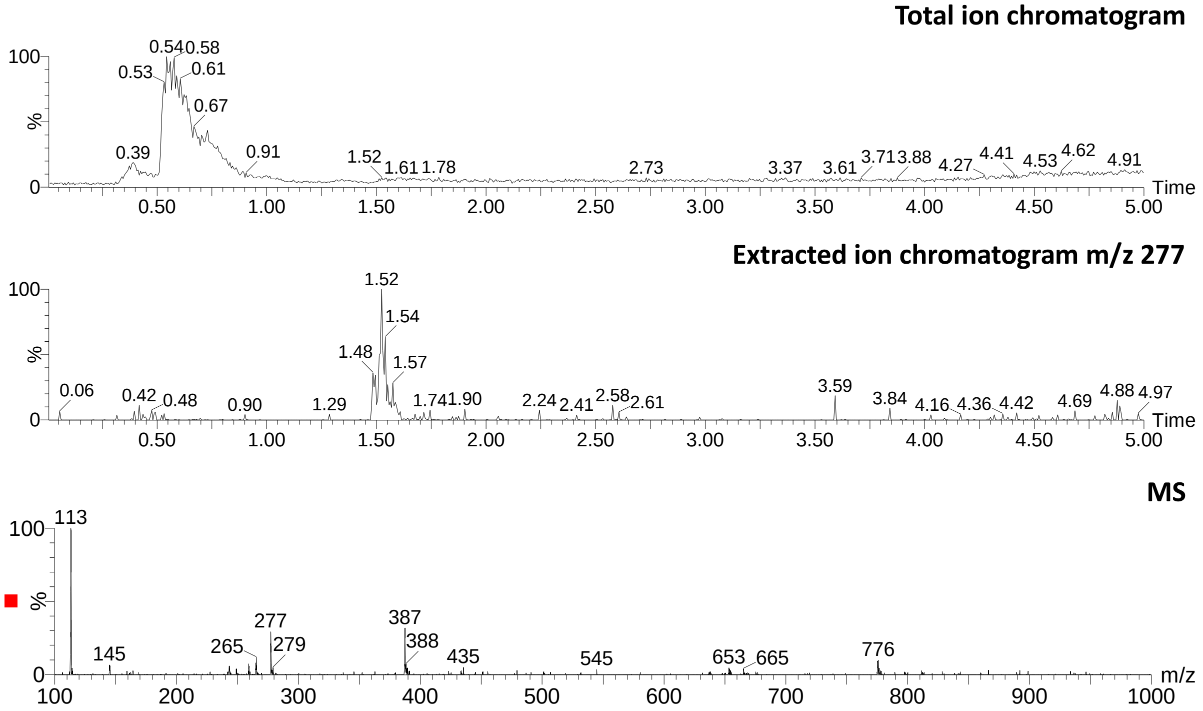

Peptide experiments

General procedure: To somatostatin (200µL, 150 µM) in pH 9.0 buffer (50 mM BB with 40% MeCN) was added TCEP (2 µL, 150 mM in water, 10 eq.) and reaction mixture was allowed to stand at 22 °C for 1 h. To the reaction mixture was added reagents (20 eq. EDC **4**, 10 eq. MKYM **3**, or 20 eq. CDMP **8**). The reaction was incubated at 37 °C for 1-24 h. Reaction mixture was analysed by LCMS. Regioselectivity assignment was inferred from the results above rather than by explicit MS-MS analysis.

Reaction of MKYM 3 and TCEP with somatostatin

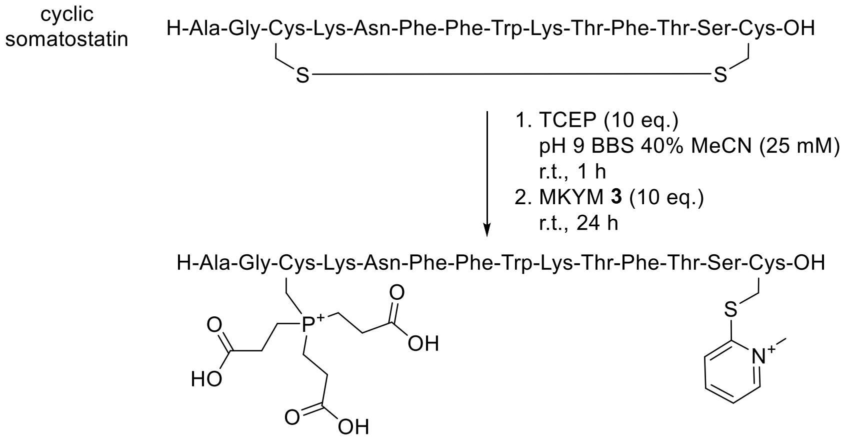

$[M]^{3+}$ m/z 650
$[M]^{4+}$ m/z 488

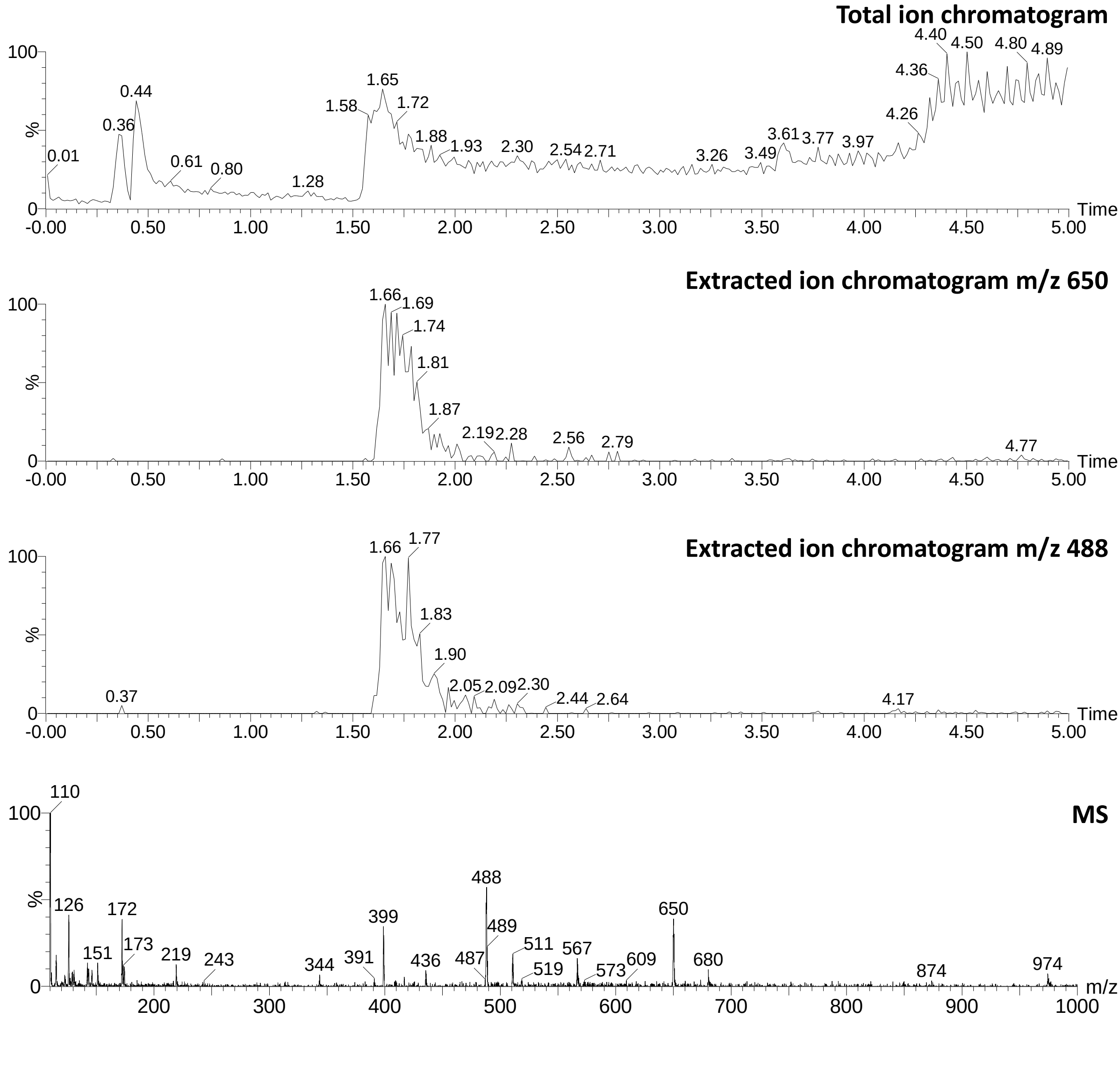

Reaction of EDC 4 and TCEP with somatostatin

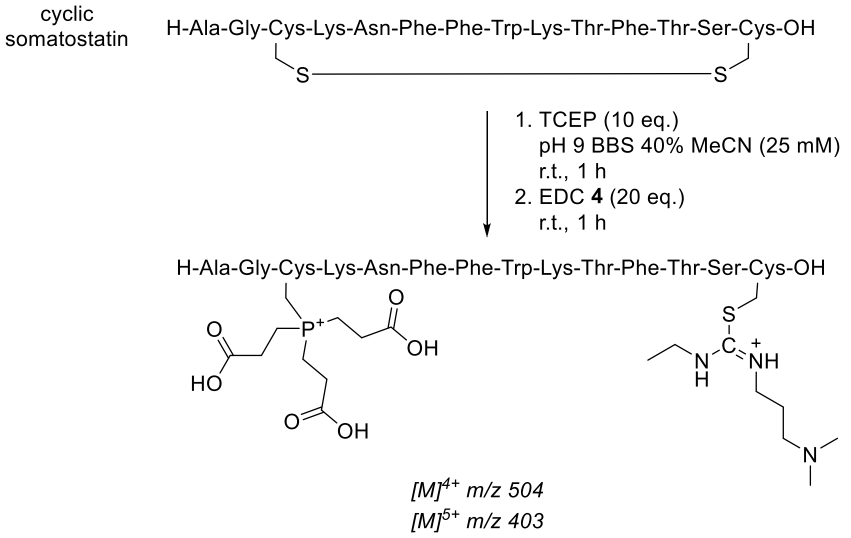

Total ion chromatogram

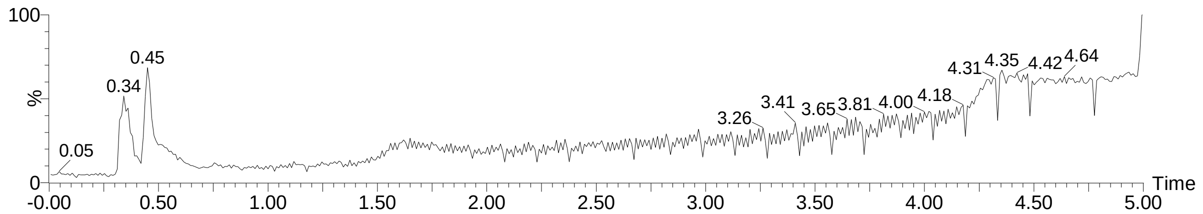

Extracted ion chromatogram m/z 504

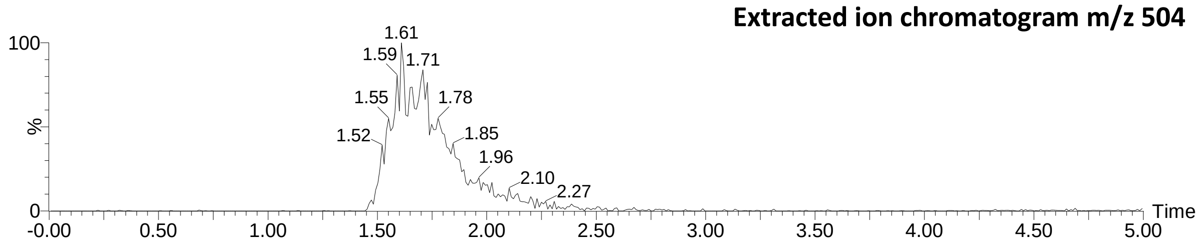

Extracted ion chromatogram m/z 403

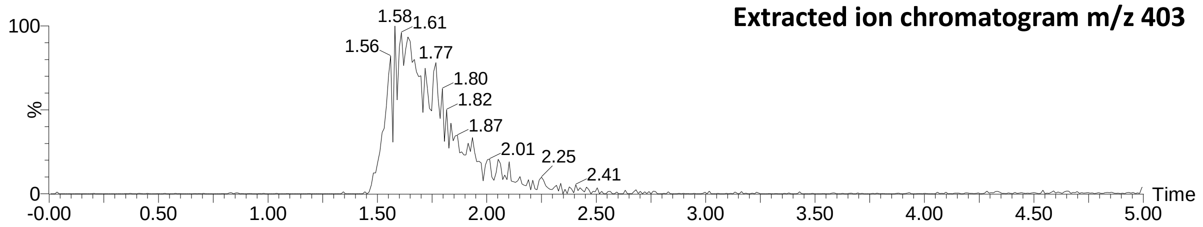

MS

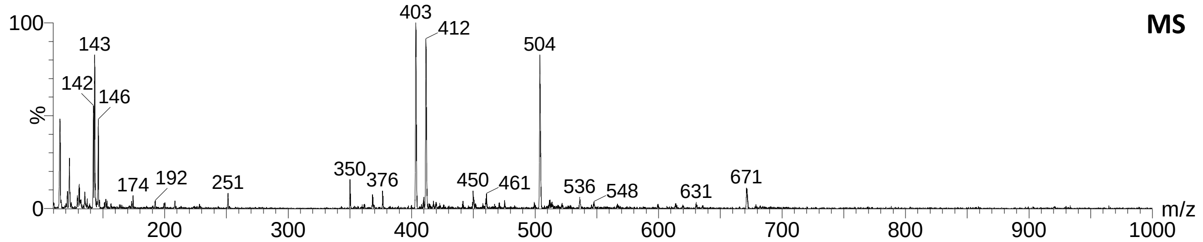

Reaction of CDMP 8 and TCEP with somatostatin

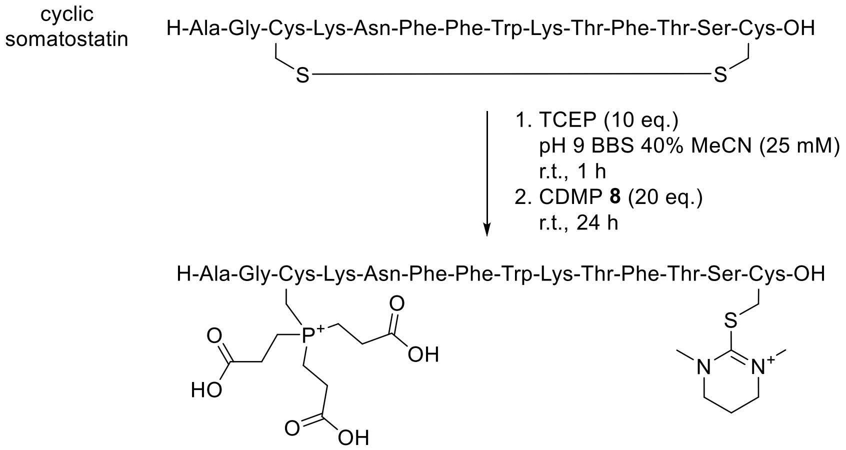

[M]$^{3+}$ m/z 657
[M]$^{4+}$ m/z 493

Total ion chromatogram

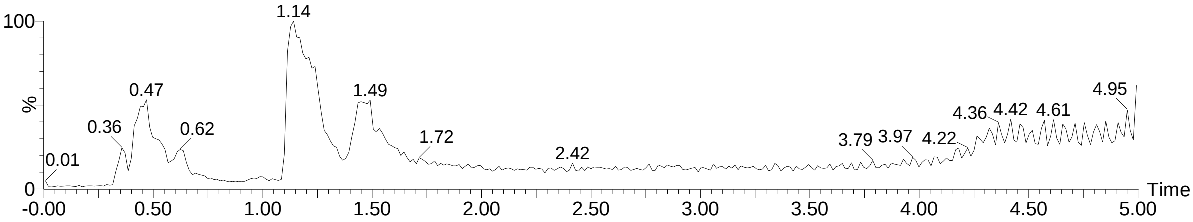

Extracted ion chromatogram m/z 657

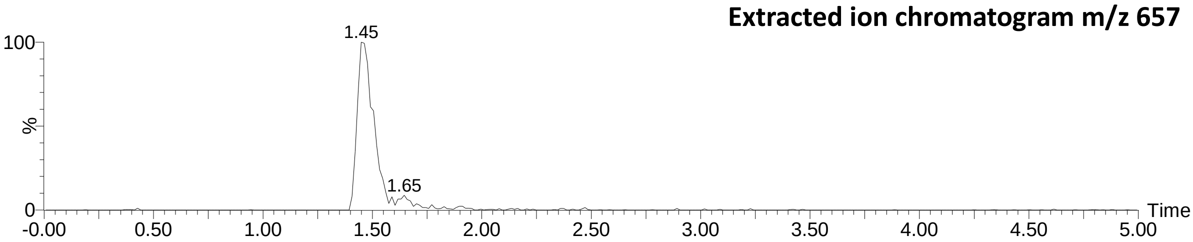

Extracted ion chromatogram m/z 493

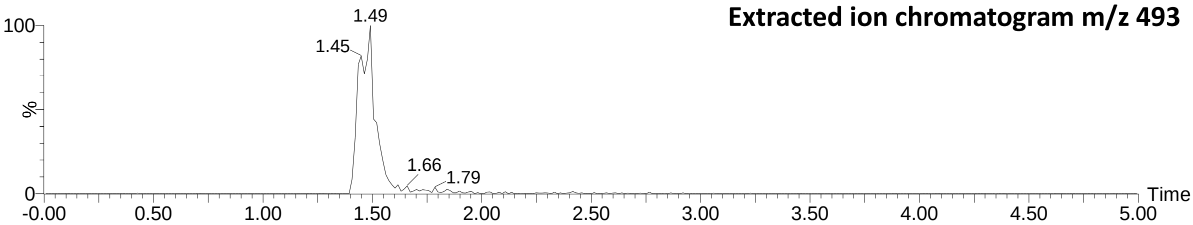

MS

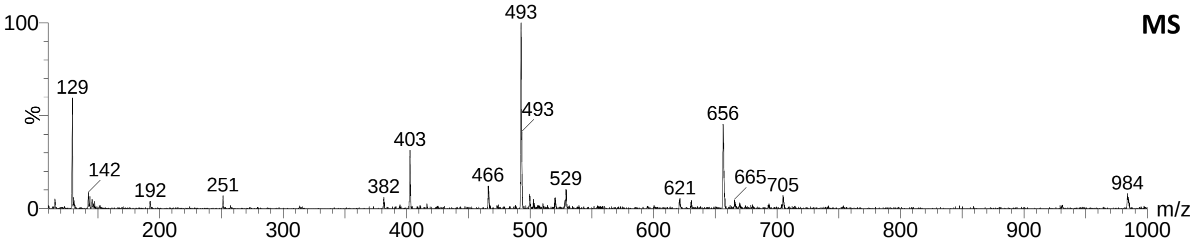

References

1 M. T. W. Lee, A. Maruani, J. R. Baker, S. Caddick and V. Chudasama, *Chem. Sci.*, 2015, **7**, 799–802.

2 P. Kandasamy, Y. Liu, V. Aduda, S. Akare, R. Alam, A. Andreucci, D. Boulay, K. Bowman, M. Byrne, M. Cannon, O. Chivatakarn, J. D. Shelke, N. Iwamoto, T. Kawamoto, J. Kumarasamy, S. Lamore, M. Lemaitre, X. Lin, K. Longo, R. Looby, S. Marappan, J. Metterville, S. Mohapatra, B. Newman, I.-H. Paik, S. Patil, E. Purcell-Estabrook, M. Shimizu, P. Shum, S. Standley, K. Taborn, S. Tripathi, H. Yang, Y. Yin, X. Zhao, E. Dale and C. Vargeese, *Nucleic Acids Res.*, 2022, **50**, 5401–5423.
